# An Analysis of Overstory Tree Canopy Cover in Sites Occupied by Native and Introduced Cottontails in the Northeastern United States with Recommendations for Habitat Management for New England Cottontail

**DOI:** 10.1371/journal.pone.0135067

**Published:** 2015-08-12

**Authors:** Bill Buffum, Thomas J. McGreevy, Amy E. Gottfried, Mary E. Sullivan, Thomas P. Husband

**Affiliations:** Department of Natural Resources Science, University of Rhode Island, Kingston, Rhode Island, United States of America; U.S. Geological Survey, UNITED STATES

## Abstract

The New England cottontail (*Sylvilagus transitionalis*) is a high conservation priority in the Northeastern United States and has been listed as a candidate species under the Endangered Species Act. Loss of early successional habitat is the most common explanation for the decline of the species, which is considered to require habitat with dense low vegetation and limited overstory tree canopy. Federal and state wildlife agencies actively encourage landowners to create this habitat type by clearcutting blocks of forest. However, there are recent indications that the species also occupies sites with moderate overstory tree canopy cover. This is important because many landowners have negative views about clearcutting and are more willing to adopt silvicultural approaches that retain some overstory trees. Furthermore, it is possible that clearcuts with no overstory canopy cover may attract the eastern cottontail (*S*. *floridanus*), an introduced species with an expanding range. The objective of our study was to provide guidance for future efforts to create habitat that would be more favorable for New England cottontail than eastern cottontail in areas where the two species are sympatric. We analyzed canopy cover at 336 cottontail locations in five states using maximum entropy modelling and other statistical methods. We found that New England cottontail occupied sites with a mean overstory tree canopy cover of 58% (*SE±1*.*36*), and was less likely than eastern cottontail to occupy sites with lower overstory canopy cover and more likely to occupy sites with higher overstory canopy cover. Our findings suggest that silvicultural approaches that retain some overstory canopy cover may be appropriate for creating habitat for New England cottontail. We believe that our results will help inform critical management decisions for the conservation of New England cottontail, and that our methodology can be applied to analyses of habitat use of other critical wildlife species.

## Introduction

Conservation of the New England cottontail (*Sylvilagus transitionalis*) is a high priority in the Northeastern United States (US). The range of the species decreased by more than 80% during the past fifty years [1], prompting the US Fish & Wildlife Service (USFWS) to nominate it as a candidate for threatened or endangered status under the Endangered Species Act [2]. A number of factors have contributed to the decline of the New England cottontail, including competition with eastern cottontail (*S*. *floridanus*), which was introduced from the Southern and Midwestern US to supplement cottontail populations for hunting between 1920 and 1960 [1, 3, 4]. The population of eastern cottontail has continued to expand while the New England cottontail has declined, likely due to the former’s adaptability to a wider variety of habitat types [4–7] and ability to avoid predators [7]. However, the loss of early successional habitat and habitat fragmentation are generally considered to be the most important reasons for the decline of New England cottontail [8, 9]. Early successional habitat was widely available in the region in the early 20^th^ century due to agricultural abandonment, but in recent decades most of this habitat has transitioned to mature forests with less understory cover and become more fragmented by development and infrastructure [10–12]. For example, a recent study in the state of Rhode Island estimated that the extent of upland shrubland forests in non-coastal areas was declining by at least 1.5% per year and that the average patch size was just 1.2 ha [13].

The New England Cottontail Technical Committee proposed an ambitious target of restoring 14,500 ha of habitat by 2020 [9]. The Natural Resources Conservation Service (NRCS) of the US Department of Agriculture encourages private landowners to create habitat for New England cottontail by offering technical and financial assistance [14]. The currently recommended approach to create habitat is clearcutting, which involves removing or killing by girdling all trees with diameter at breast height greater than 7.6 cm, except for a small number of apple trees, scrub oak, or low branching conifers left to provide cover and food sources [15, 16]. The patch size of clearcuts was recommended to be at least 10 ha because occupancy of small patches is believed to result in lower body weight, increased risky foraging behavior, and increased mortality from depredation [8, 9].

However, some aspects of the habitat use of New England cottontail are not well understood. For example, there are recent indications that the species occupies sites with a moderate amount of overstory tree canopy; research in Connecticut reported that New England cottontail occupied sites with a mean overstory canopy closure of 74% and a basal area of 12.3 m^2^/ha [17]. Monitoring of New England cottontail reintroduced to Patience Island, RI also found that New England cottontail was equally likely to occupy mixed forest sites (with mean basal area of 8 m per ha) as bramble-vine thicket sites (with mean basal area 3.8 m per ha) [18]. These findings imply that retention of a greater number of overstory trees may be an appropriate silvicultural alternative to clearcuts for creating habitat for New England cottontail. This is important because many landowners have negative views about clearcutting [19–22] and almost half of the habitat target of the New England Regional Technical Committee is planned to be achieved on private land [9].

Recent advances in conservation genetics permit reliable species identification of cottontails by analysis of fecal pellets and can provide valuable information about habitat use [23]. The University of Rhode Island Wildlife Genetics and Ecology Laboratory has analyzed more than 2,500 cottontail fecal samples collected in five states since 2008. This is the first study to use this important new source of data to better understand the habitat use of New England cottontail and eastern cottontail. This study also is the first to use new Geographic Information System (GIS) datasets from the Landscape Fire and Resource Management Planning Tools Project, managed by the US Department of Agriculture and the US Department of Interior [24] to conduct a landscape analysis of habitat use of New England cottontail and eastern cottontail.

**Fig 1:**
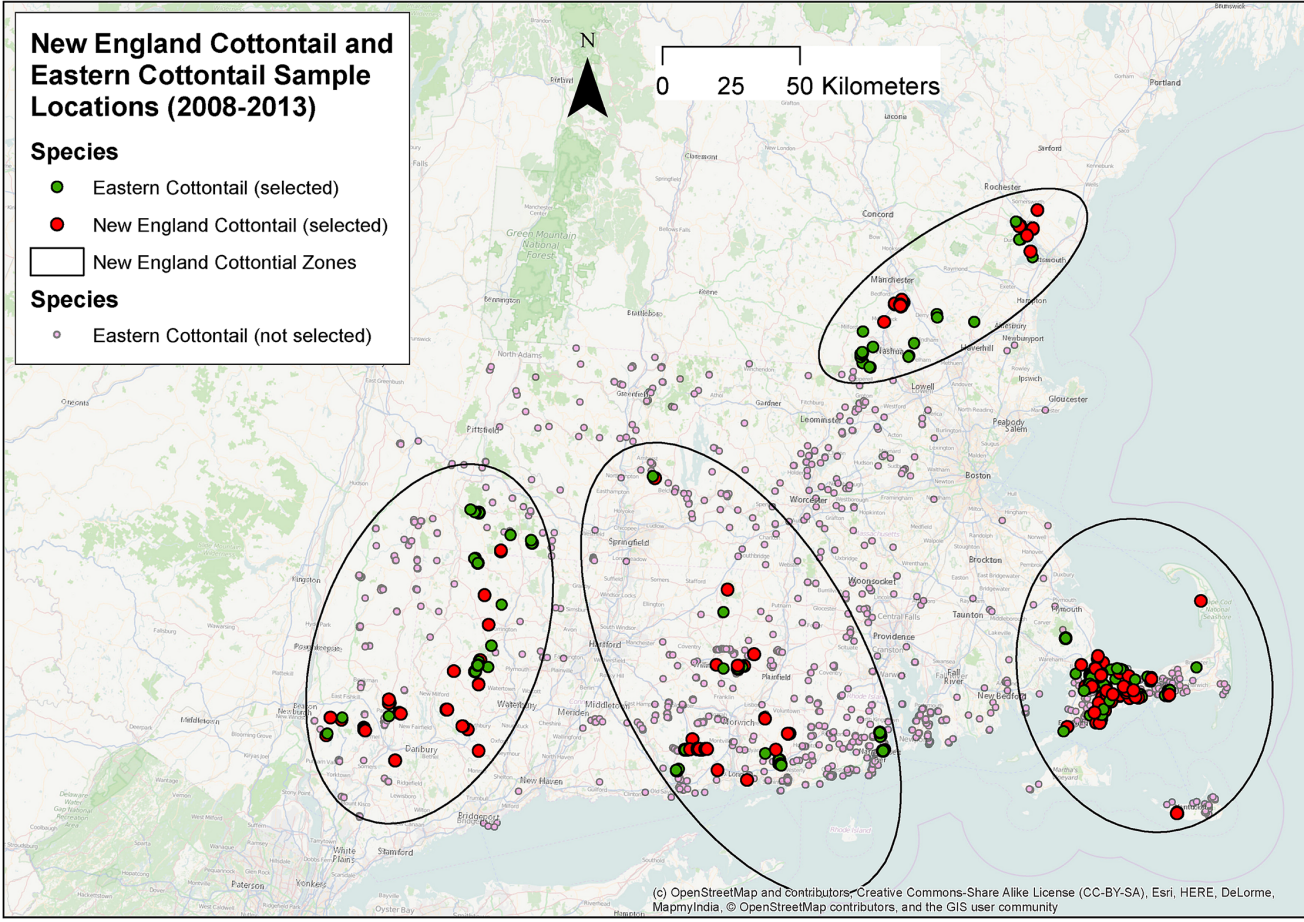
Locations of all cottontail samples identified from 2008 to 2013 and locations of New England cottontail (n = 168) and eastern cottontail (n = 168) selected for the current study, by zone. Notes: New England cottontail sites that were not selected are too close to selected samples to be visible in this map. License information about Open Street Map is available here.

The objective of our study was to provide guidance for future efforts to create habitat that will be more favorable for New England cottontail than eastern cottontail in areas where the two species are sympatric. We used the locations of New England cottontail and eastern cottontail sampled between 2008 and 2013 in five states in the Northeastern US where the two species are sympatric (Fig 1) in conjunction with existing GIS datasets to assess occupancy of sites with different amounts of overstory tree canopy cover. Our study addressed the following research question: are New England cottontail and eastern cottontail equally likely to occupy sites with high and low amounts of overstory tree canopy cover in areas where the two species are sympatric? We believe that the results have direct implications for efforts to create habitat to conserve the New England cottontail and that the same approach can be applied to analyze the occupancy habitat use of other critical wildlife species.

## Methods

### Overall Study Design

We selected an equal number of New England cottontail and eastern cottontail locations for the study. We assessed overstory tree canopy cover within 75 m of points where New England cottontail or eastern cottontail had been detected. This area (1.77 ha) exceeds most home range estimates for New England cottontail, although these are highly variable, ranging from 0.1 ha to 7.6 ha [8]. We assumed an average dispersal area of New England cottontail of 3 km based on previous studies [3, 25] and compared the habitat attributes of the occupied sites (within 75 m) to the available habitat within the dispersal area (within 3 km).

### GIS and Statistical Software

We conducted GIS analyses using ArcGIS 10.2 (Environmental Systems Research Institute, Redlands, CA) and the Geospatial Modelling Environment 0.7.2.1 (in conjunction with ArcGIS 10.1). We conducted statistical analyses using IBM SPSS Statistics v. 22 (International Business Machines Corp.) and SAS Software version 9.2 (SAS Institute, Inc., Cary, NC). We used Maximum Entropy Species Distribution Modelling (MaxEnt) Version 3.3.3k for modelling (available for download at https://www.cs.princeton.edu/~schapire/maxent/) [26, 27]. All reported results are for two-sided tests with an alpha level of 0.05 unless otherwise noted.

### Ethics Statement

The collection of fecal samples used in this study was approved by the University of Rhode Island Institutional Animal Care and Use Committee (IACUC) via Approval no. AN11-12-011, and was in accordance with the Guidelines of the American Society of Mammalogists for the Use of Wild Mammals in Research [28]. All sampling procedures were specifically approved as part of obtaining the IACUC approval. The study did not involve any federally endangered or protected species, but the US Fish & Wildlife Service has nominated the New England cottontail as a candidate for threatened or endangered status, and the states of New Hampshire and Maine have listed it as endangered. We were involved in both the collection and analysis of samples collected in the state of Rhode Island. We obtained approval from the Rhode Island Department of Environmental Management for the collection of fecal samples on state properties; from the US Fish and Wildlife Service for collection on federal properties; and from private landowners for collection on private land. State, federal, and university biologists collected additional fecal samples in Connecticut, Massachusetts, New Hampshire, New York, and Rhode Island and submitted to us for analysis. We conducted in-person trainings with the regional collectors in Connecticut, Massachusetts, New Hampshire and Rhode Island to ensure a consistent sample collection protocol. We are not able to release the exact locations of our samples for ethical and legal reasons, but we have included the data for our sample points (S1 Dataset) as Supporting Information.

### Collection of samples

We compiled the coordinates of all available New England cottontail and eastern cottontail samples collected between 2007 and 2013 from the five states where the two species are generally sympatric: Connecticut, Massachusetts, New Hampshire, New York, and Rhode Island. We did not include samples from Maine because there were no sites where New England cottontail and eastern cottontail samples were sympatric.

State, federal, and university biologists collected 2,543 fecal samples in Connecticut, Massachusetts, New Hampshire, New York, and Rhode Island and submitted to the University of Island Wildlife Genetics and Ecology Laboratory for analysis. Fecal samples were stored in sterile 15 mL tubes with either 100% ethanol or 5 mL of silica beads (Sigma Aldrich, St. Louis, MO) and a Kimwipe (Kinberly-Clark, Irving, TX) to prevent the fecal pellet from contacting the silica beads. Either a new tongue depressor or a natural object from the field (e.g., stick) was used to transfer the fecal pellet from the ground to the collection tube. Fecal samples collected with silica beads remained at ambient temperature for at least one hour to absorb moisture from the pellets and were subsequently stored in a -80°C freezer until DNA was extracted. The GPS locations of all samples collected were recorded; however, the time spent in each location was not recorded. We also requested the state wildlife offices to provide the locations of additional samples analyzed in other laboratories, and received data for 1,108 additional samples.

Most surveys took place 24 to 72 hours after a snowfall event and continued until temperatures rose above freezing or a rain event occurred. In locations with no snowfall during the field season, surveys took place when temperatures remained at or below freezing for at least 2 days. Detection of cottontail in the winter is much easier than in the summer: Brubaker et al [29] reported an overall winter detection rate for New England cottontail of 0.73, which they attributed to increased visibility in areas of dense vegetative cover and the accumulation of tracks and pellets on top of snow; they also noted that 82% of all detections occurred within the first 20 minutes of a survey, and that the stem density of the site did not affect detectability. In contrast, a study of live trapping of eastern cottontail reported detection rates of only 0.44 [30], confirming the detection advantages of pellet collection in the winter as mentioned above by Brubaker et al [29].

The selection of sites for the regional survey efforts was based on the individual needs of each state, but generally focused on areas where either (a) cottontails were known to occur; (b) the habitat appeared to be suitable for cottontails; or (c) the areas were of high priority for management. No attempt was made to search in all land use types; however, a post-sampling analysis found that the proportion of samples in most land use types matched the prevalence of the land use type in the New England cottontail range (Table 1). The exceptions were scrub/shrub, which was sampled more intensively (6% of samples) than its regional prevalence (2%), while evergreen forest and mixed forest were sampled less intensively (4% and 4% respectively) than their regional prevalence (11% and 10% respectively).

**Table 1:** Proportion of samples and New England cottontail (NEC) range by land use type.

Land use type*	All samples (n = 3649)	Random samples in study (n = 336)	NEC Range**
Bare Land	0.00	0.00	0.01
Cultivated Crops	0.04	0.02	0.02
Deciduous Forest	0.34	0.29	0.37
Developed, High Intensity	0.00	0.00	0.02
Developed, Low Intensity	0.08	0.11	0.06
Developed, Medium Intensity	0.03	0.07	0.04
Developed, Open Space	0.06	0.10	0.04
Estuarine Emergent Wetland	0.02	0.01	0.01
Estuarine Scrub/Shrub Wetland	0.00	0.00	0.00
Evergreen Forest	0.04	0.08	0.11
Grassland/Herbaceous	0.04	0.03	0.01
Mixed Forest	0.04	0.05	0.10
Palustrine Emergent Wetland	0.02	0.00	0.01
Palustrine Forested Wetland	0.07	0.06	0.08
Palustrine Scrub/Shrub Wetland	0.01	0.01	0.01
Pasture/Hay	0.14	0.08	0.08
Scrub/Shrub	0.06	0.08	0.02
Grand Total	1.00	1.00	1.00

Notes:

* Land use is based on the Coastal Change Analysis Program of the US National Oceanic and Atmospheric Administration, but does not include open water, aquatic bed or unconsolidated shore.

** This is a rough approximation of the NEC range based on a rectangle surrounding all known NEC sample locations.

### Selection of samples

Our total number of 3,649 cottontail locations (441 New England cottontail and 3,208 eastern cottontail) included 2,543 samples analyzed by the University of Rhode Island Wildlife Genetics and Ecology Laboratory and 1,106 additional samples from the state wildlife offices of Connecticut (792), Massachusetts (280), and New Hampshire (34) that were analyzed in other laboratories (Table 2, Fig 1). To avoid overlap between our primary sampling areas (within 75 m of cottontail locations) and at the same time maximize sample size, we used the ArcGIS RAN tool to randomly select New England cottontail locations with a minimum separation of 150 m, which generated 168 samples. We classified the samples into four zones corresponding to the four of the five currently distinct populations of New England cottontail identified in previous studies, the fifth population being in Maine [1, 3, 9].

**Table 2:** Selection of New England cottontail (NEC) and eastern cottontail (EC) points by zone.

Zones (NEC)	NEC Total Points	NEC Randomly selected points	EC Total Points	EC Randomly selected points	Proportion of selected NEC samples within 3 km of the nearest EC sample
Northeast	65	22	-	22	0.23
South	119	40	-	40	0.95
Southeast	135	69	-	69	0.90
Southwest	122	37	-	37	0.81
Total	441	168	3,208*	168	0.80

Notes:

* EC had a much wider distribution than NEC and could not be subdivided into NEC zones.

We selected a similar number of eastern cottontail locations in three steps. We randomly selected one eastern cottontail sample within the assumed dispersal distance (3 km) of each selected New England cottontail point using the ArcGIS RAN tool. Because eastern cottontail samples were not available within 3 km of 20% of our New England cottontail samples, we identified and selected the nearest eastern cottontail sample to these remaining New England cottontail samples using the ArcGIS NEAR tool. Finally, because some of the selected eastern cottontail samples were the nearest eastern cottontail sample to more than one New England cottontail sample, we identified additional eastern cottontail samples by taking random samples from expanding buffers around the remaining New England cottontail samples until we reached the target of 168 eastern cottontail samples, using the ArcGIS RAN tool.

### Assessment of overstory tree canopy cover

We assessed overstory tree canopy density using the 2010 Existing Vegetation Cover (EVC) dataset of the Landscape Fire and Resource Management Planning Tools Project, US Department of Agriculture and the US Department of Interior [24]. The EVC is the only currently available dataset of overstory tree canopy cover for the entire range of New England cottontail. Although this 30 m pixel dataset is generally recommended for regional analyses, we considered it to be appropriate for our analysis because our primary area of analysis (75 m radius) included approximately 20 pixels per site and our sample size was relatively high (N = 336). We checked the accuracy of EVC estimates of forest cover at our scale of analysis by comparing them to detailed field studies of 146 potential cottontail sites in Connecticut and Rhode Island [17]. The latter study used a convex spherical densiometer to estimate canopy closure (the proportion of sky hemisphere obscured by vegetation when viewed from a single point), while EVC used remote sensing to estimate canopy cover (the proportion of forest floor covered by the vertical projection of tree crowns). These two attributes are not always strongly correlated for a number of reasons; for example, tree canopy closure is affected by tree height while canopy cover is not [31]. However, we found a significant nonparametric Kendall’s tau correlation (τ = 0.30, p<0.001) and a stronger Pearson correlation (r = 0.50) between the densiometer estimates of canopy closure and the EVC estimates of canopy cover. The EVC tree canopy cover estimates were 18% less than the field estimates of canopy closure, which agreed with Paletto and Tosi [32] who reported that estimates of canopy closure were up to 20% higher than estimates of canopy cover in the same sites.

### Comparison of overstory tree canopy density in occupied sites to availability within dispersal area

We calculated the mean overstory tree canopy density (within 75 m) of each cottontail location using ArcGIS focal statistics. The area of a circle with a radius of 75 m (1.77 ha) exceeds most home range estimates for New England cottontail, although these are highly variable, ranging from 0.1 ha to 7.6 ha [8]. We considered 3 km to be the dispersal area of New England cottontail based on previous studies [3, 25]. In a 3 km radius circle around each cottontail location, we used the ArcGIS RAN tool to generate random pseudo-absence points, and calculated: (a) the mean overstory density (within 75 m) at each pseudo-absence point; and (b) the mean overstory tree canopy density (within 75 m) of all of the pseudo-absence points in each circle. We repeated the same process for two intermediate distances (150 m and 1 km).

In selecting the pseudo-absence points, we followed the guidance of Stokland et al [33]: (a) we used a random design which provides flexibility in generating different sample sizes; (b) we limited the spatial extent to conditions that were not far outside the ecological tolerance of the species (in our case we excluded open water); and (c) we did not exclude pseudo-absence points from where the species are known to occur, but rather generated points in the entire area of interest which included an occupied point in its center. We only selected pseudo-absence points inside of the 3 km dispersal areas, which followed the recommendation of Fourcade et al. [34] that the training area should be strictly relevant to the ecology of the species and should reflect the space accessible to the species. The numbers of random pseudo-absence points were as follows: within 150 m (10 random points for each occupied site, for a total of 3,600 points); within 1 km (100 random points per occupied site, for a total of 33,600 points); within 3 km (100 random points for each occupied site, for a total of 33,600 points).

Since all of the concentric circles were located around the same cottontail location points, we could compare these mean densities using One-Way Repeated-Measures Analysis of Variance (*F*). We also tested for significant linear trends. When Mauchly’s test indicated that the assumption of sphericity had been violated, the degrees of freedom were corrected by IBM SPSS Statistics using Greenhouse-Geisser estimates of sphericity [35].

We used a similar approach to compare the proportion of sites occupied by New England cottontail and eastern cottontail by canopy class (within 75 m) with the availability of sites in each canopy class within 150 m, 1 km, and 3 km. We classified the overstory tree canopy cover (within 75 m) of each cottontail location into one of six tree canopy cover classes (non-forest, 1–20% canopy cover, 21–40% canopy cover, 41–60% canopy cover, 61–80% canopy cover, and 81–100% canopy cover). We then classified all of the pseudo-absence points into the same tree canopy classes.

We tested for differences between New England cottontail and eastern cottontail in the use of overstory tree canopy cover in the occupied areas (within 75 m) with binary logistic regression tests, using SAS Software version 9.2, and a binary logit model with Fisher’s scoring as the optimization technique. We also conducted chi-squared tests (χ^2^) to compare the numbers of New England cottontail and eastern cottontail in each canopy class.

### Maximum entropy modelling of occupancy of sites with different overstory tree canopy density

We modelled the occupancy of the two cottontail species at sites with different mean overstory tree canopy densities within 77 m using Maximum Entropy Modelling (MaxEnt) Version 3.3.3k, which is designed for modeling species geographic distributions with presence-only data [26] and produces a number of outputs useful for our analysis including (a) an area under the receiver operating curve (AUC) for both the training and test data, (b) the significance of the prediction using a binomial test of omission, and (c) a response curve that shows how the predicted suitability relates to changes in an environmental variable. Traditionally, wildlife analyses have been conducted with both presence and-absence data; however, absence data can be difficult to verify as a species may been present at a site but not observed [36]. In our MaxEnt analyses we reserved 25% of the samples for testing the model performance, set the maximum number of background points (pseudo-absences) at 10,000, and ran each model ten times to measure the amount of variability in the model. We generally followed the Maxent default options: our only changes in addition to those mentioned above were: (a) using the random seed option, which results in a different set of pseudo-absences being selected for each replicated run; and (b) using a subsample replicated run type, as recommended when conducting replicated runs [37]. All of the settings used in the MaxEnt analyses can be seen in S1 Model Outputs, S2 Model Outputs, S3 Model Outputs and S4 Model Outputs.

## Results

The mean tree canopy cover within 75 m of sites occupied by New England cottontail was 58%, (*SE±1*.*36*), ranging from a high of 74% (*SE±1*.*32*) in the Southwest zone to 46% (*SE±4*.*44*) in the Northeast zone, and was significantly lower than the tree canopy cover of the surrounding areas in most zones (Table 3). We found a significant linear trend of decreasing tree canopy cover with increasing area of analysis and a significant difference between tree canopy cover within 75 m and tree canopy cover within 3 km in every zone except the southwest zone.

**Table 3:** Proportion of tree canopy cover at sites occupied by New England cottontail (NEC) and eastern cottontail (EC) by zone and scale of analysis.

Species and Zone	Proportion tree canopy within 75 m	Proportion tree canopy within 150 m *	Proportion tree canopy within 1 km **	Proportion tree canopy within 3 km **	Increasing linear trend of canopy cover with scale of analysis	Difference between tree canopy cover within 75 m and 3 km
NEC Northeast	0.46	0.46	0.60	0.63	*F* (1, 21) = 15.166, *p*<0.005	t(21) = -3.884, p<0.001
NEC South	0.59	0.64	0.71	0.73	*F* (1, 39) = 49.384, *p*<0.005	t(39) = -6.710, p<0.001
NEC Southeast	0.54	0.56	0.59	0.60	*F* (1, 68) = 12.596, *p*<0.005	t(68) = -3.497, p<0.001
NEC Southwest	0.74	0.73	0.76	0.76	NS	NS
NEC Total	0.58	0.61	0.64	0.67	*F* (1, 167) = 55.519, *p*<0.001	t(167) = -7.288, p<0.001
EC Northeast	0.49	0.58	0.65	0.69	F (1, 21) = 17.594, p<0.001	t(21) = -4.275, p<0.001
EC South	0.59	0.63	0.68	0.71	F (1, 39) = 433.486, p<0.001	t(39) = -5.898, p<0.001
EC Southeast	0.43	0.50	0.56	0.58	F (1, 68) = 34.714, p<0.001	t(68) = -6.049, p<0.001
EC Southwest	0.68	0.70	0.76	0.77	F (1, 21) = 15.166, p<0.001	t(36) = -4.250, p<0.001
EC Total	0.53	0.58	0.65	0.67	F (1, 167) = 97.308, p<0.001	t(167) = -9.891, p<0.001

Notes: NS = not significant

* based on average value for 10 random pseudo-absence points per cottontail location (total = 3,360)

** based on average value for 100 randomly generated pseudo-absence points per cottontail location (total = 36,000)

Different patterns emerged when we analyzed occupancy by tree canopy cover class. New England cottontail occupied sites in all of the six canopy cover classes except for the 1–20% class, but 79% of the occupied sites were in just two tree canopy classes (41–60% and 61–80%), with the highest percentage of sites in the 61%-80% canopy class (Table 4). The percentage of sites occupied by New England cottontail was significantly higher than the percentage of available sites within 3 km for the 41–60% canopy cover class, and higher, though not significantly so, for the 61–80% canopy cover class. Furthermore, we found decreasing trends in these percentages as the area of analysis increased, although not significant, for the 61–80% canopy cover class. In contrast, the percentage of sites occupied by New England cottontail in the lowest and highest tree canopy classes was significantly lower than the percentage of sites within 3 km, and we found significant increasing trends in these percentages as the area of analysis increased.

**Table 4:** Proportion of six overstory tree canopy site classes (based on mean tree canopy within 75 m) occupied by New England cottontail (NEC) and eastern cottontail (EC) availability at six site classes within 150 m, 1 km, and 3 km of occupied sites (based on the proportion of random sites having the appropriate mean overstory tree canopy within 75m).

Species and site class (mean overstory tree canopy cover within 75 m)	Proportion of sites occupied by cottontail	Availability of site classes within 150 m *	Availability of site classes within 1 km *	Availability of site classes within 3 km *	Linear trend: percent availability of site class with increasing area of analysis	Difference between proportion of sites occupied by cottontail and availability of site classes within 3 km
NEC Non-forest	0.04	0.05	0.07	0.08	Increasing: *F* (1, 167) = 12.794, *p*<0.001	t(167) = -3.586, p<0.001
NEC 1–20%	0.00	0.01	0.00	0.01	None: NS	t(167) = -7.133, p<0.001
NEC 21–40%	0.10	0.10	0.08	0.07	Decreasing: NS	NS
NEC 41–60%	0.34	0.34	0.28	0.27	Decreasing: *F* (1, 167) = 5.599, *p*<0.05	t(167) = 2.065, p<0.05
NEC 61–80%	0.45	0.43	0.41	0.41	Decreasing: NS	NS
NEC 81–100%	0.07	0.08	0.15	0.16	Increasing: *F* (1, 167) = 38.673, *p*<0.001	t(167) = -5.603, p<0.001
EC Non-forest	0.08	0.09	0.10	0.11	Increasing: *F* (1, 167) = 4.142, p<0.05	t(167) = -2.085, p<0.05
EC 1–20%	0.01	0.01	0.01	0.01	None: NS	NS
EC 21–40%	0.11	0.09	0.09	0.07	Decreasing: NS	NS
EC 41–60%	0.38	0.38	0.27	0.25	Decreasing: *F* (1, 167) = 2.106, p<0.001	t(167) = 3.611, p<0.001
EC 61–80%	0.35	0.34	0.38	0.38	Increasing: NS	NS
EC 81–100%	0.08	0.09	0.15	0.18	Increasing: *F* (1, 167) = 25.267, p<0.001	t(167) = -4.659, p<0.001

Notes: NS = not significant

* based on the proportion of pseudo absence points in each overstory tree canopy cover class (mean overstory tree canopy cover within 75m), with 10 random pseudo-absence points per cottontail location (total = 3,360) for analyses within 150m, and 100 randomly generated pseudo-absence points per cottontail location (total = 36,000) for analyses within 1km and 3km.

**Fig 2:**
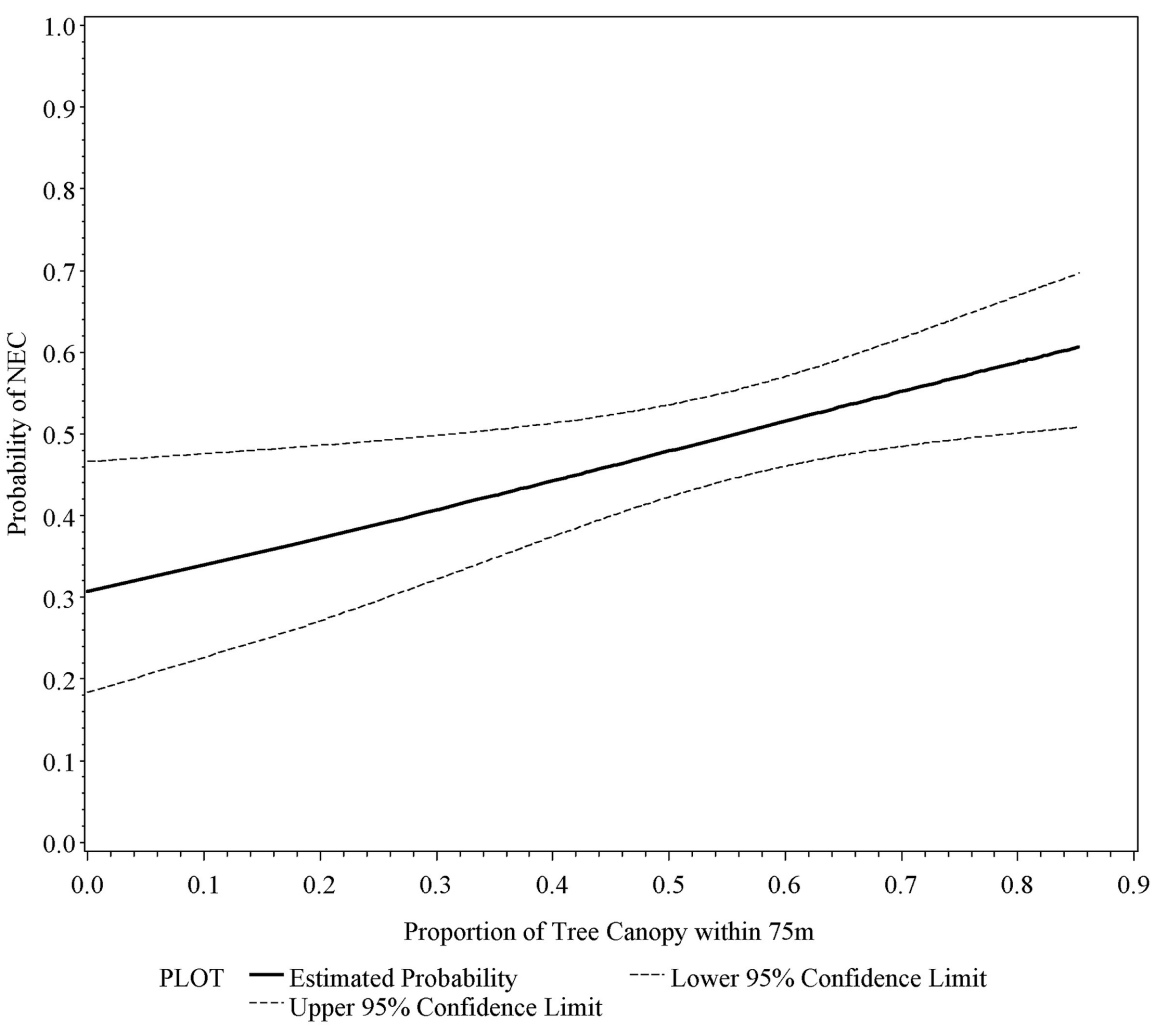
Logistic regression of the probability of New England cottontail (NEC) presence versus eastern cottontail presence based on average percent tree canopy closure within 75 m of detection location (Wald = 6.2230, p < 0.05). Notes: The frequencies of the two species in each canopy classes are provided in parentheses. New England cottontail: 0(6); 0.01–0.1 (0); 0.11–0.2 (4); 0.21–0.3 (13); 0.31–0.4 (19); 0.41–0.5 (38); 0.51-.06 (44); 0.61–0.7 (32); 0.71–0.8 (12); 0.81–1.0 (0). Eastern cottontail: 0 (13); 0.01–0.1 (1); 0.11–0.2 (2); 0.21–0.3 (17); 0.31–0.4 (35); 0.41–0.5 (29); 0.51-.06 (40); 0.61–0.7 (19); 0.71–0.8 (12); 0.81–1.0 (0).

A logistic regression revealed a significant positive relationship between the probability of occupancy by New England cottontail versus eastern cottontail and the proportion of tree canopy cover (within 75 m) in the entire study area (*Wald = 6*.*2230*, *p < 0*.*05*), where the probability of occupancy by New England cottontail ranged from 31% at 0% canopy cover to 60% at 85% canopy cover, with 50% probability at 55% canopy cover (Fig 2). We detected significant positive relationships in two of the four zones: Southeast (*Wald = 8*.*371*, *p < 0*.*01*) and Southwest (*Wald = 6*.*3998*, *p < 0*.*05*). A chi squared test confirmed that New England cottontail was more likely than eastern cottontail to occupy sites with 60 to 80% tree canopy (*χ* ^*2*^ *= 3*.*579*, *p < 0*.*05*, *one-sided*), and less likely to occupy non-forest sites, although this was not significant (*χ* ^*2*^ *= 2*.*734*, *p = 0*.*07*, *one-sided*).

The MaxEnt analysis revealed similar trends. The receiver operating curves (ROC) from running each model ten times with different sets of pseudo-absence points revealed that models based on canopy cover (within 75m) when the training area was limited to the 3 km dispersal distance resulted in a mean area under the curve (AUC) of 0.652 for New England cottontail with a standard deviation of 0.030, and an AUC of 0.651 for eastern cottontail with a standard deviation of 0.039. These AUC results for New England cottontail were significant (*p < 0*.*05*, *one sided)* for 95.45% of the 110 MaxEnt binomial tests (11 tests for each of the 10 replicates), and for eastern cottontail were significant for 82.73% of the 110 tests. See S1 Model Outputs and S2 Model Outputs for the full set of MaxEnt results when the training area was limited to the 3km dispersal distance.

Both of these mean AUC values were lower than the 0.70 threshold for classifying the usefulness of the model as “good”, but well over the 0.5 AUC value for being more useful than a “random model”[38], which we found encouraging for a model based on a single environmental attribute. Moreover, when we ran similar analyses with a training area that included the entire New England Cottontail range rather than just the 3km dispersal area, the mean AUC values for both New England cottontail and eastern cottontail exceeded the 0.7 threshold for model classification as “good”. These analyses resulted in a mean area under the curve (AUC) of 0.733 for New England cottontail with a standard deviation of 0.019, and an AUC of 0.727 for eastern cottontail with a standard deviation of 0.027. These AUC results were significant (*p < 0*.*05*, *one sided)* for 96.36% of the 110 MaxEnt binomial tests for New England cottontail, and significant for 88.18% of the 110 MaxEnt binomial tests for eastern cottontail. See S3 Model Outputs and S4 Model Outputs for the full set of MaxEnt results when the training area included the entire New England Cottontail range.

**Fig 3:**
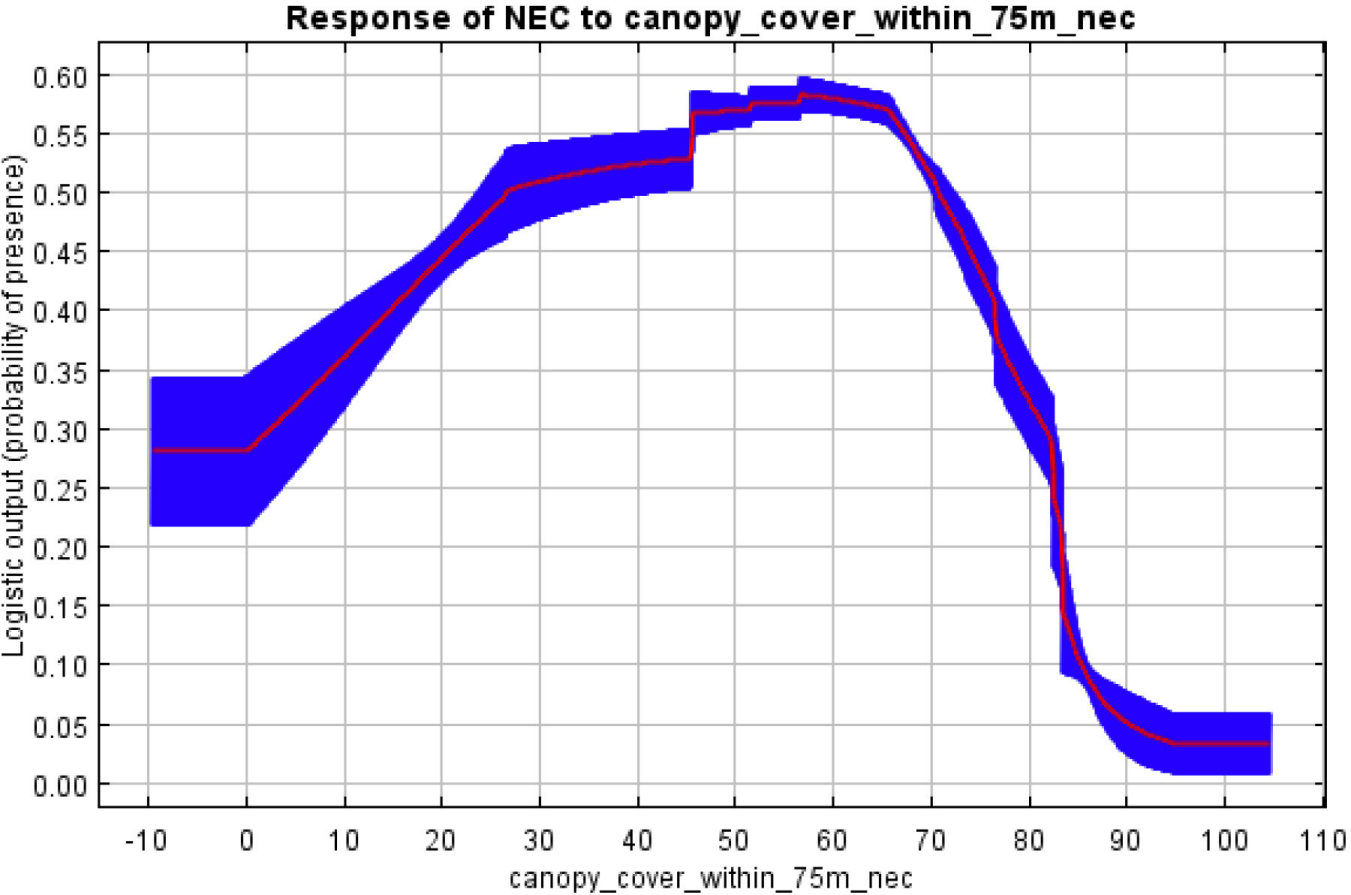
Response curve of New England cottontail (NEC): probability of presence and mean overstory tree canopy cover within 75m, generated by Maximum Entropy Modelling (MaxEnt) with training area limited to 3km dispersal areas.

**Fig 4:**
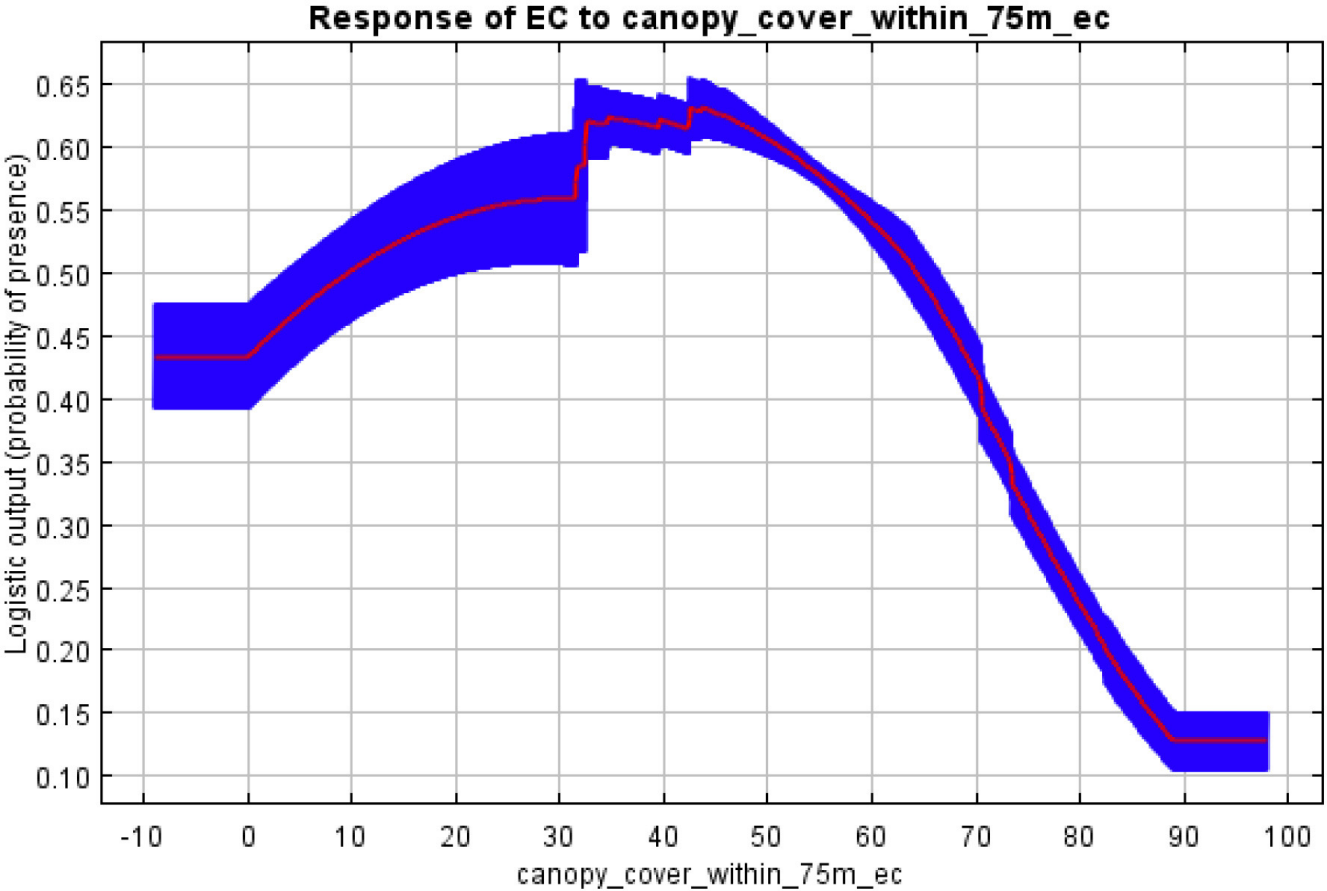
Response curve of eastern cottontail (EC): probability of presence and mean overstory tree canopy cover within 75m, generated by Maximum Entropy Modelling (MaxEnt) with training area limited to 3km dispersal areas.

The response curves for probability of presence to increasing canopy cover when the training area was limited to the 3 km dispersal distance were distinctly different for New England cottontail (Fig 3) than for eastern cottontail (Fig 4). New England cottontail had a lower mean probability of presence in 0% canopy cover than eastern cottontail (27% vs. 43%) and a higher mean probability of presence in 70% canopy cover (52% vs. 38%) and in 80% canopy cover (33% vs. 25%).

## Discussion

Achieving the target set by the New England Cottontail Technical Committee of restoring 14,500 ha of habitat by 2020 will be a major challenge, especially because almost half of the area is planned for implementation on private land [9]. The Natural Resources Conservation Service of the US Department of Agriculture actively encourages landowners to create habitat for New England cottontail by offering technical and financial assistance [14], but many landowners have negative views about forest clearcutting [19–22] the approach currently recommended for habitat creation [15, 16]. We believe that the findings of our study will support an expanded program of habitat creation by identifying an alternative silvicultural approach which will produce suitable habitat in a way that is more acceptable to landowners.

We found that both New England cottontail and eastern cottontail were generalists that occupied sites with a wide range of overstory tree canopy densities, but that New England cottontail was more likely than eastern cottontail to occupy areas with higher tree canopy cover and less likely to occupy areas with low tree canopy cover. The average tree canopy density for New England cottontail was 58% (*SE±1*.*36*). Most New England cottontail (79%) occupied sites with tree canopy between 41% and 80%, with more New England cottontail in the 61%-80% tree canopy class than any other canopy class (Table 4). New England cottontail was less likely to occupy sites in the highest canopy cover class (81–100%) and the lowest (non-forest), and occupancy in these classes was lower than the availability within their 3 km dispersal area.

Our findings agree with recent research based on telemetry data in Connecticut where sites occupied by New England cottontail had higher tree canopy closure than sites occupied by eastern cottontail [17] and in Rhode Island where released New England cottontail was equally likely to occupy mixed forest sites as bramble-vine thicket sites [18]. Our findings also agree with research conducted in Illinois where the presence of eastern cottontail was negatively associated with canopy closure [30].

We further investigated site occupancy by the two cottontail species using MaxEnt, which is designed for modeling species geographic distributions with presence-only data [26]. This analysis confirmed that overstory tree canopy density is an important environmental variable for both species, and that New England cottontail has a higher probability of presence in sites with more overstory tree canopy, while eastern cottontail has a higher probability of presence in sites with less overstory tree canopy.

MaxEnt is well suited for our analysis. Baasche et al [39] compared three modelling approaches and found that MaxEnt produced the most accurate and precise estimates of the known probability distribution when use occurs within a localized area, such as a home range, and availability was defined at a larger scale—both of which were the case in our study. Normally MaxEnt would be used to model species distributions based on a number of environmental factors, but we focused on one single factor: overstory tree canopy density. The more typical modelling approach is not feasible for the cottontail species that we studied due to a lack of spatial datasets on understory vegetation density, the key environmental factor for these two species. There has been at least one attempt to date to develop a habitat suitability index (HSI) for New England Cottontail using proxies for understory vegetation density [40]. However, when the resulting HSI was used to select sites for cottontail sampling in Rhode Island, it was found to be less effective at identifying sites with dense understory vegetation than a model based on a state-wide shrubland layer. New England Cottontail was not found in any of the sites identified by either model; however, 72% of sites identified by the shrubland model contained eastern cottontail as compared to only 19% of the sites identified by the HSI model [17]. There is currently no region-wide dataset on understory vegetation density, although Light Detection and Ranging (LiDAR) data is starting to become available in the region and has potential to generate such a dataset [41].

We recognize that occupancy of a habitat does not necessarily mean that the habitat is suitable for the concerned species. Gottfried [17] noted that the current patterns of site occupancy may be the result of New England cottontail having been excluded from more desirable patches due to competition with eastern cottontail. Van Horne [42] noted that a positive correlation between species abundance and habitat quality is often wrongly assumed, whereas other factors including individual survival probability and mean expectation of future offspring also affect habitat quality. Van Horne [38] pointed out that density may be a good indicator of habitat quality under certain conditions: (a) if the species of concern is rare; (b) if the occupied sites are identified during the season of highest mortality, and (c) if the habitat is not patchy. Our example of New England cottontail met two of these three conditions: the species is very rare in most parts of its range and the sampling was done during winter when mortality is highest [8], but not the third condition of occupying a non-patchy habitat. Therefore we share the concerns of Gottfried [17] and Van Horne [38], and agree that further research is required on immigration, survival, and reproduction patterns of New England cottontail in different site types. We plan to collaborate with the Rhode Island Department of Environmental Management in an expanded program of telemetry studies of New England cottontail during the next five years, which will address these issues.

Despite these limitations, our study provides evidence that New England cottontail is not limited to shrublands and young forests as other authors have suggested [3, 8, 15], but also occupies sites in maturing forests with overstory canopy cover of up to 80%. Accordingly, we believe that shelterwood cuts, which involve two or three harvests over a ten year period [43], may be a viable silvicultural approach to create habitat for New England cottontail. Shelterwood cuts retain 30% to 50% of the overstory canopy cover in the initial cut and create ideal conditions for regeneration of both shade tolerant and intolerant species in northeastern forests [43, 44]. Shelterwood cuts can be more effective than clearcuts in promoting regeneration in sites that are not already well stocked with seedlings and saplings [45], a condition common in the Northeast US due to heavy browsing by white-tailed deer (*Odocoileus virginianus*). Shelterwood cuts simultaneously provide habitat for both mature forest bird species and early successional bird species, resulting in greater diversity of bird species than either clearcuts or mature forests [46]. Furthermore, landowners are more likely to be willing to implement shelterwood cuts, whereas they often have negative views about clearcuts [19–21], the currently recommended approach to create habitat for New England cottontail [47, 48].

Shelterwood cuts have some disadvantages over clearcuts, however. For example, the persistence of the understory habitat in shelterwood cuts may be lower if a landowner implements the initial cut but omits the subsequent cuts. Understory vegetation generally declines after the overstory canopy closes [49], which is probably why the New England cottontail in our study avoided sites with 81–100% overstory canopy cover. Additionally, clearcuts provide better habitat than shelterwood cuts for some early successional specialist bird species [46]. Therefore, we recommend that both silvicultural approaches are promoted, dependent upon the objectives and concerns of individual landowners.

## Conclusions

This is the first study of New England cottontail to employ new data from the genetic analysis of more than 2,500 cottontail samples and new spatial datasets of the Landscape Fire and Resource Management Planning Tools Project. These datasets cover the entire United States, so our methodology can be used to support habitat studies for other critical wildlife species. Our findings have important implications for the selection of silvicultural approaches to create habitat for New England cottontail. We recommend that further research be conducted on the impact of shelterwood cuts and other silvicultural systems on occupancy, immigration, survival, and reproduction patterns by New England cottontail and eastern cottontail.

## Supporting Information

S1 Dataset Data for overstory tree canopy cover at sites occupied by New England cottontail (n = 168) and eastern cottontail ((n = 168). (XLSX)

S1 Model Outputs Maximum entropy modeling (MaxEnt) outputs for New England cottontail with training area limited to 3km dispersal area (10 replicates). (PDF)

S2 Model Outputs Maximum entropy modeling (MaxEnt) outputs for eastern cottontail with training area limited to 3km dispersal area (10 replicates). (PDF)

S3 Model Outputs Maximum entropy modeling (MaxEnt) outputs for New England cottontail with training area set for entire New England cottontail range (10 replicates). (PDF)

S4 Model Outputs Maximum entropy modeling (MaxEnt) outputs for eastern cottontail with training area set for entire New England cottontail range (10 replicates). (PDF)
