# Correction: An Analysis of Overstory Tree Canopy Cover in Sites Occupied by Native and Introduced Cottontails in the Northeastern United States with Recommendations for Habitat Management for New England Cottontail

**DOI:** 10.1371/journal.pone.0138741

**Published:** 2015-09-16

**Authors:** Bill Buffum, Thomas J. McGreevy, Amy E. Gottfried, Mary E. Sullivan, Thomas P. Husband

There is an error in the caption for Fig 2, “Logistic regression of the probability of New England cottontail (NEC) presence versus eastern cottontail presence based on average percent tree canopy closure within 75 m of detection location (Wald = 6.2230, p < 0.05).” Please see the corrected Fig 2 here.

**Fig 2:**
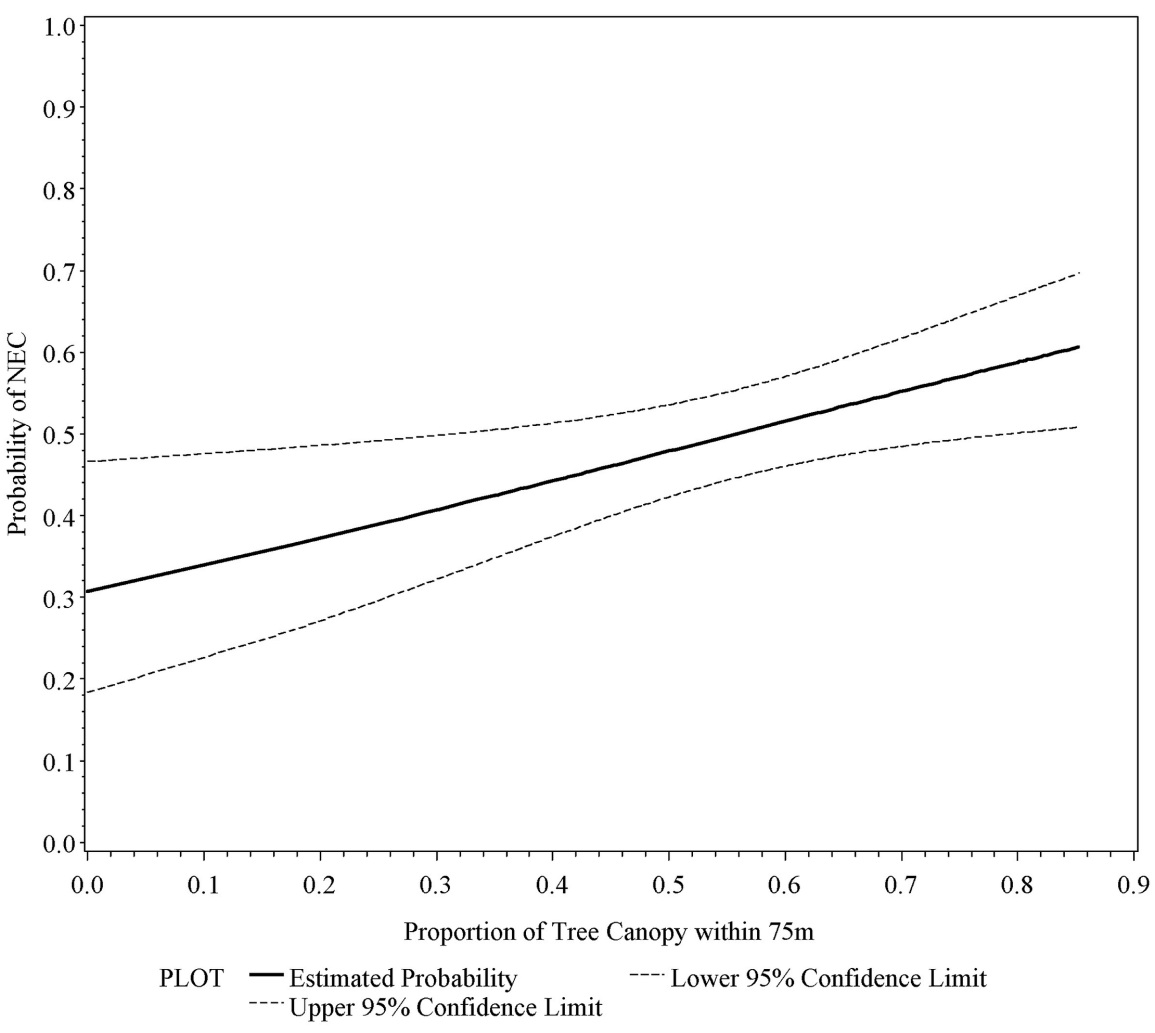
Logistic regression of the probability of New England cottontail (NEC) presence versus eastern cottontail presence based on average percent tree canopy cover within 75 m of detection location (Wald = 6.2230, p < 0.05). Notes: The frequencies of the two species in each canopy classes are provided in parentheses. New England cottontail: 0(6); 0.01–0.1 (0); 0.11–0.2 (4); 0.21–0.3 (13); 0.31–0.4 (19); 0.41–0.5 (38); 0.51-.06 (44); 0.61–0.7 (32); 0.71–0.8 (12); 0.81–1.0 (0). Eastern cottontail: 0 (13); 0.01–0.1 (1); 0.11–0.2 (2); 0.21–0.3 (17); 0.31–0.4 (35); 0.41–0.5 (29); 0.51-.06 (40); 0.61–0.7 (19); 0.71–0.8 (12); 0.81–1.0 (0).
