# Supplementary material for: An Analysis of Overstory Tree Canopy Cover in Sites Occupied by Native and Introduced Cottontails in the Northeastern United States with Recommendations for Habitat Management for New England Cottontail
Source: PLoS One. 2015 Aug 12;10(8):e0135067. doi: 10.1371/journal.pone.0135067 (PMC4534376; doi:10.1371/journal.pone.0135067)
Supplement: S1 Model Outputs — (PDF) [file pone.0135067.s002.pdf]

Replicated maxent model for NEC

This page summarizes the results of 10 split-sample models for NEC, created Wed Jul 08 19:02:21 EDT 2015 using Maxent version 3.3.3k. The individual models are here: [0] [1] [2] [3] [4] [5] [6] [7] [8] [9]

Analysis of omission/commission

The following picture shows the test omission rate and predicted area as a function of the cumulative threshold, averaged over the replicate runs. The omission rate should be close to the predicted omission, because of the definition of the cumulative threshold.

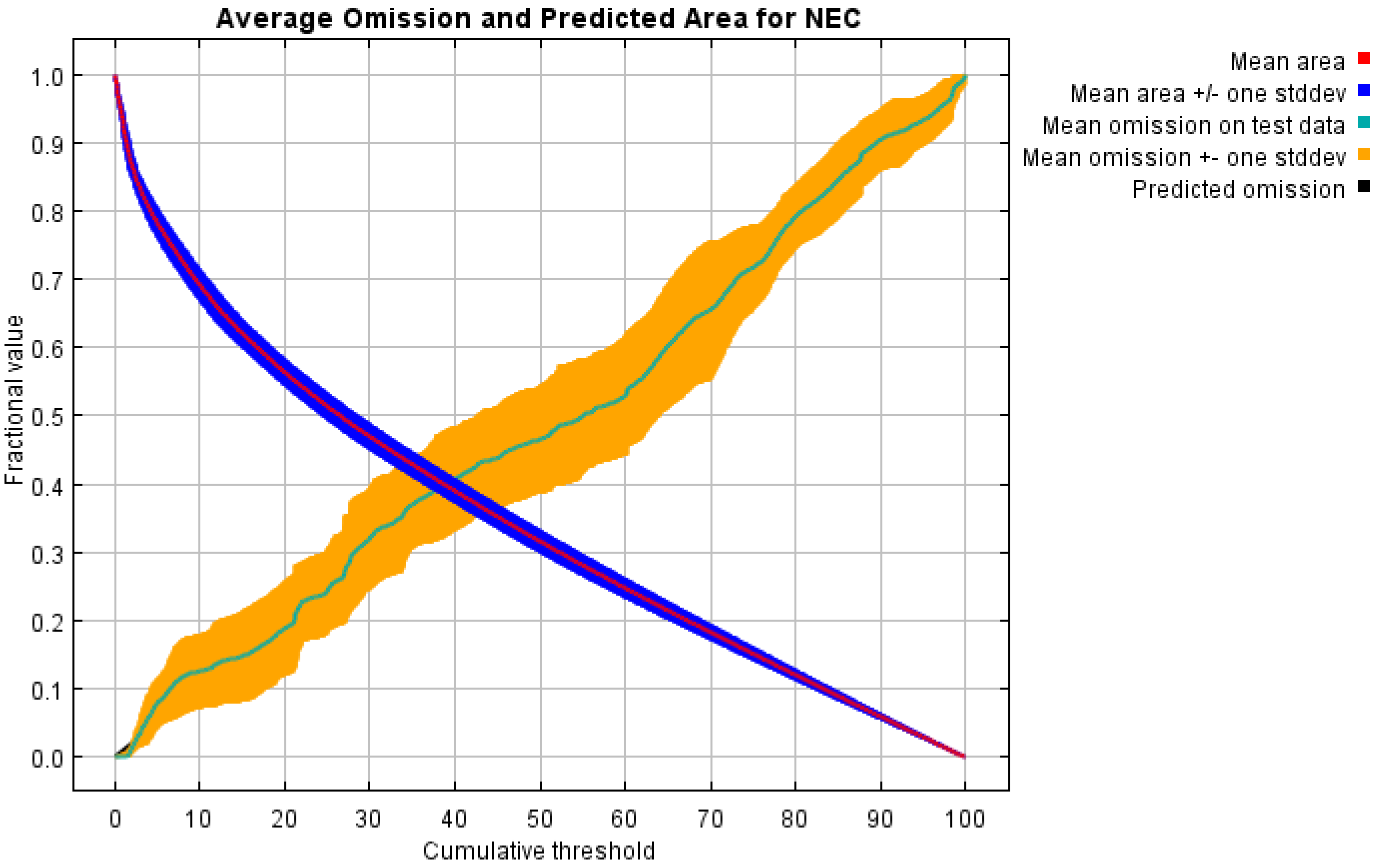

The next picture is the receiver operating characteristic (ROC) curve for the same data, again averaged over the replicate runs. Note that the specificity is defined using predicted area, rather than true commission (see the paper by Phillips, Anderson and Schapire cited on the help page for discussion of what this means). The average test AUC for the replicate runs is 0.652, and the standard deviation is 0.030.

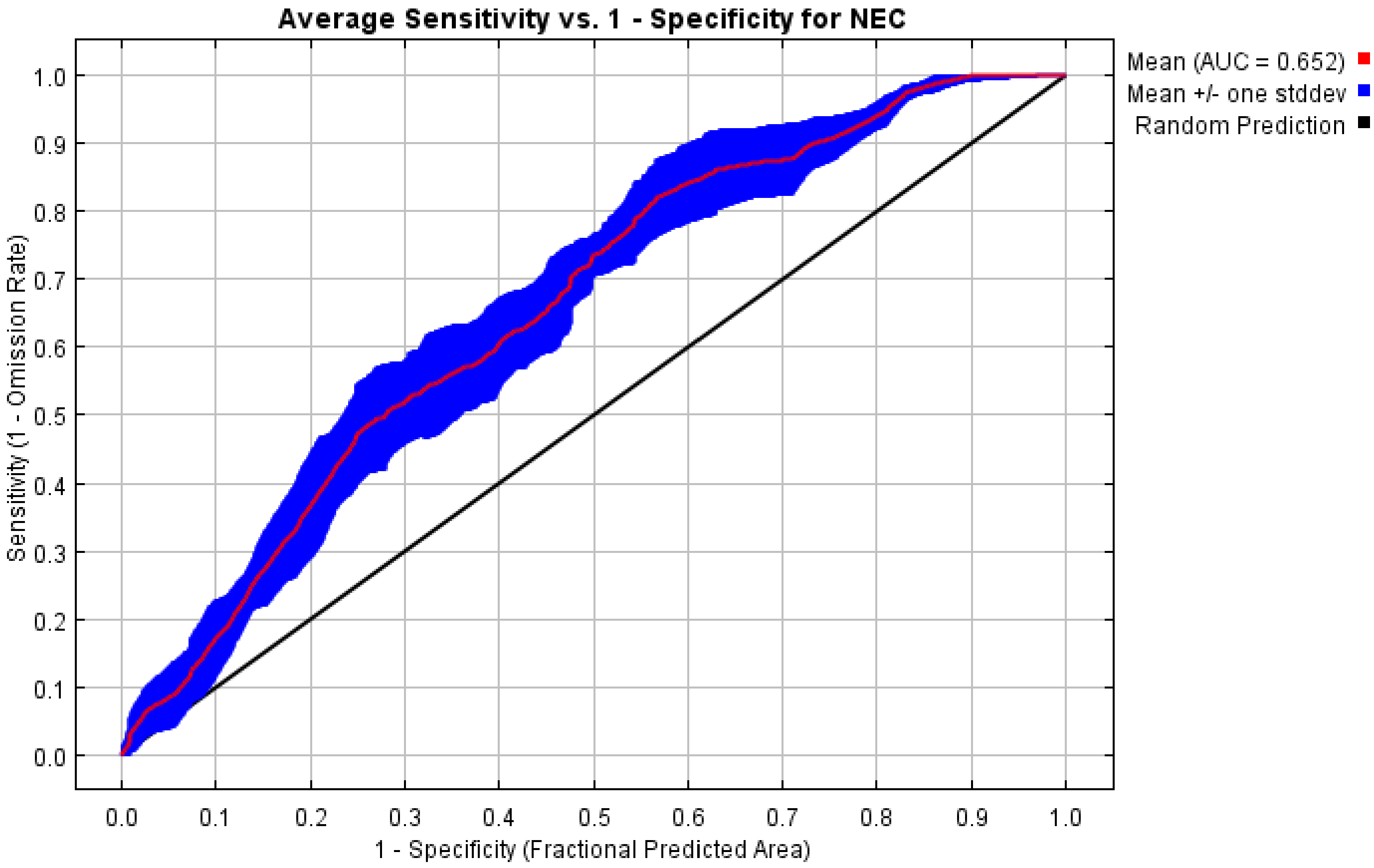

Response curves

These curves show how each environmental variable affects the Maxent prediction. The curves show how the logistic prediction changes as each environmental variable is varied, keeping all other environmental variables at their average sample value. Click on a response curve to see a larger version. Note that the curves can be hard to interpret if you have strongly correlated variables, as the model may depend on the correlations in ways that are not evident in the curves. In other words, the curves show the marginal effect of changing exactly one variable, whereas the model may take advantage of sets of variables changing together. The curves show the mean response of the 10 replicate Maxent runs (red) and and the mean +/- one standard deviation (blue, two shades for categorical variables).

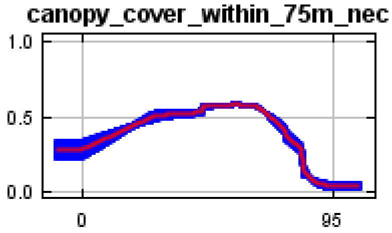

In contrast to the above marginal response curves, each of the following curves represents a different model, namely, a Maxent model created using only the corresponding variable. These plots reflect the dependence of predicted suitability both on the selected variable and on dependencies induced by correlations between the selected variable and other variables. They may be easier to interpret if there are strong correlations between variables.

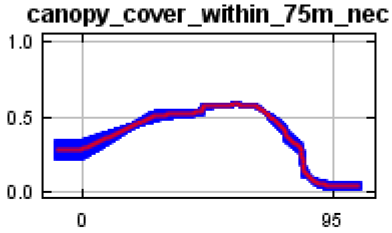

Analysis of variable contributions

The following table gives estimates of relative contributions of the environmental variables to the Maxent model. To determine the first estimate, in each iteration of the training algorithm, the increase in regularized gain is added to the contribution of the corresponding variable, or subtracted from it if the change to the absolute value of lambda is negative. For the second estimate, for each environmental variable in turn, the values of that variable on training presence and background data are randomly permuted. The model is reevaluated on the permuted data, and the resulting drop in training AUC is shown in the table, normalized to percentages. As with the variable jackknife, variable contributions should be interpreted with caution when the predictor variables are correlated. Values shown are averages over replicate runs.

Variable	Percent contribution	Permutation importance
canopy_cover_within_75m_nec	100	100

Command line to repeat this species model: java density.MaxEnt nowarnings noprefixes -E "" -E NEC responsecurves nopictures "outputdirectory=C:\Users\Bill\Desktop\Maxent Outputs NEC 7-8-15" samplesfile=C:\Users\Bill\Desktop\Maxent_samples\Compare_NEC_EC_150m_311_WGS_84_m.csv "environmentallayers=C:\Users\Bill\Desktop\Maxent Layers\maxent.cache" randomseed randomtestpoints=25 replicates=10 replicatetype=subsample -N canopy_cover_within_75m_ec -N cc10foc75_02_buff3km_nec_01_wgs84_01 -N cc10foc75_04_buff3km_nec_20_wgs84_20 -N cc10foc75_04_wgs84_02 -N cc10mfoc75_04_buff3km_ec_20_wgs84_20_01 -N cc_10foc75_d_buff3km_nec_01_wgs84_10 -N cc_10mfoc75_d_buff3km_ec_01_wgs84_10 -N vpd_b_wgs_buff10_ec_01 -N vpd_b_wgs_buff50_ec_01 -N vpd_b_wgs_ec_01 -N vpd_b_wgs_nec_01

Maxent model for NEC_0

This page contains some analysis of the Maxent model for NEC_0, created Wed Jul 08 17:34:57 EDT 2015 using Maxent version 3.3.3k. If you would like to do further analyses, the raw data used here is linked to at the end of this page.

Analysis of omission/commission

The following picture shows the omission rate and predicted area as a function of the cumulative threshold. The omission rate is is calculated both on the training presence records, and (if test data are used) on the test records. The omission rate should be close to the predicted omission, because of the definition of the cumulative threshold.

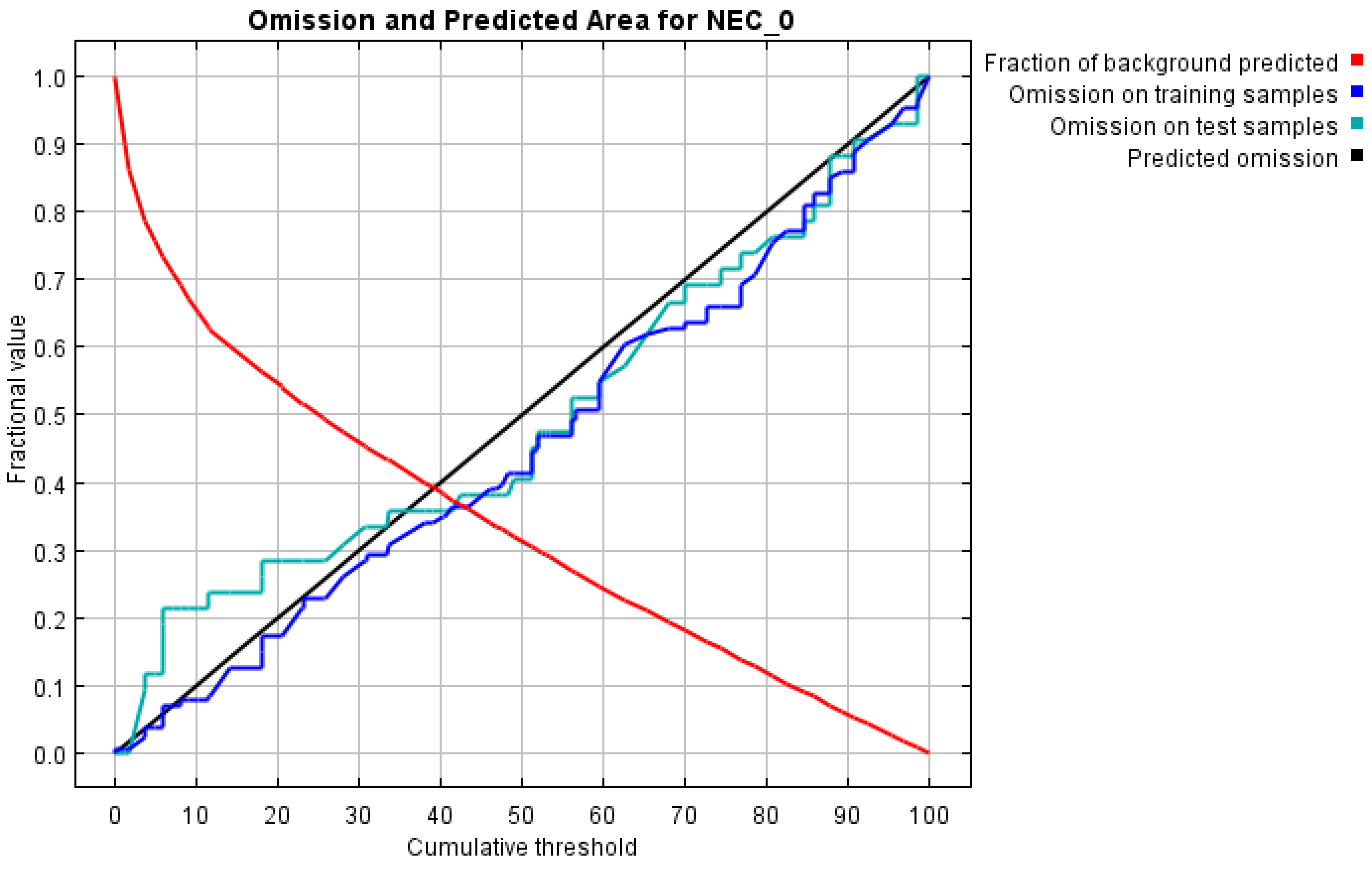

The next picture is the receiver operating characteristic (ROC) curve for the same data. Note that the specificity is defined using predicted area, rather than true commission (see the paper by Phillips, Anderson and Schapire cited on the help page for discussion of what this means). This implies that the maximum achievable AUC is less than 1. If test data is drawn from the Maxent distribution itself, then the maximum possible test AUC would be 0.660 rather than 1; in practice the test AUC may exceed this bound.

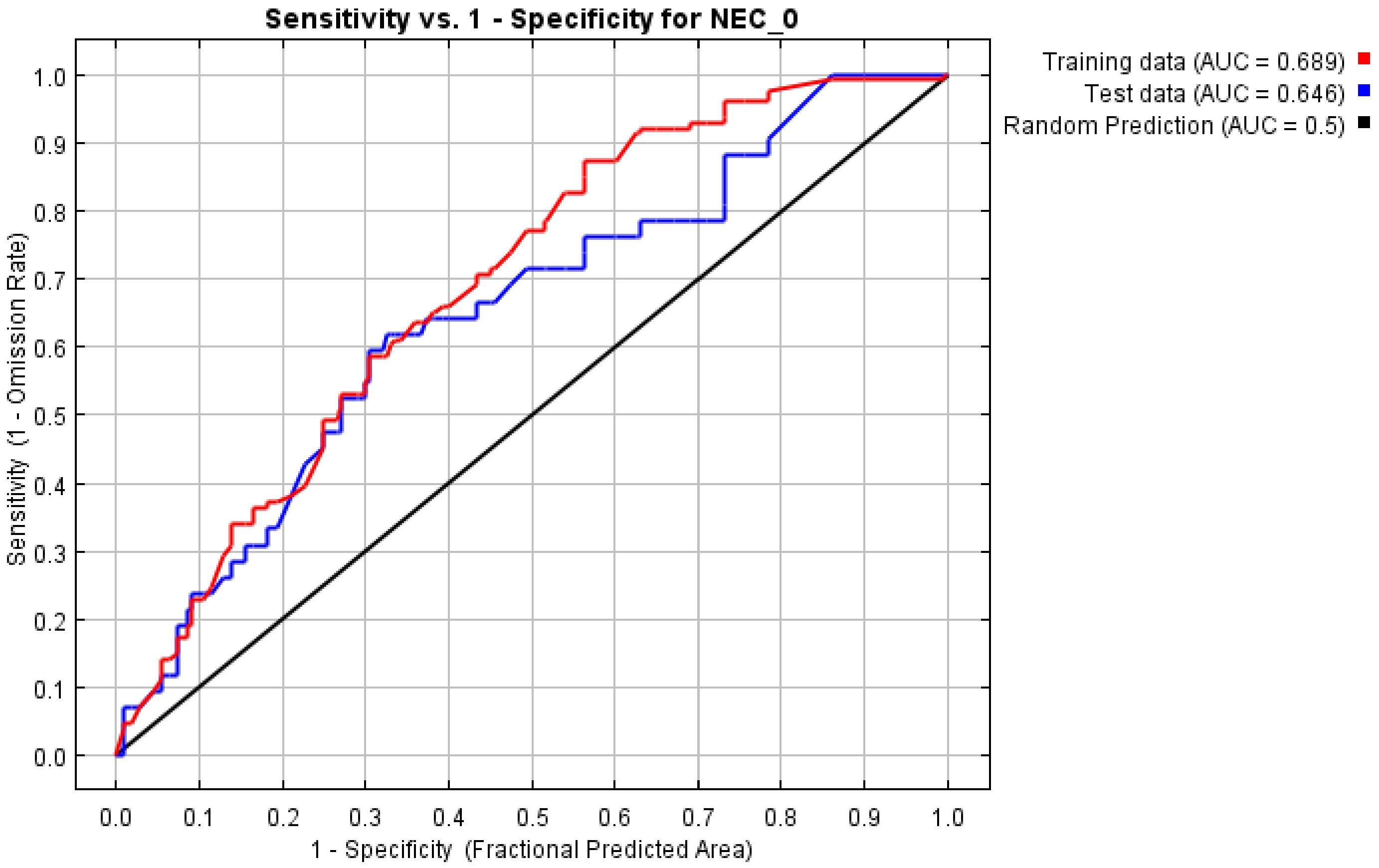

Some common thresholds and corresponding omission rates are as follows. If test data are available, binomial probabilities are calculated exactly if the number of test samples is at most 25, otherwise using a normal approximation to the binomial. These are 1-sided p-values for the null hypothesis that test points are predicted no better than by a random prediction with the same fractional predicted area. The "Balance" threshold minimizes 6 * training omission rate + .04 * cumulative threshold + 1.6 * fractional predicted area.

Cumulative threshold	Logistic threshold	Description	Fractional predicted area	Training omission rate	Test omission rate	P-value
1.000	0.088	Fixed cumulative value 1	0.899	0.008	0.000	1.483E-2
5.000	0.277	Fixed cumulative value 5	0.733	0.040	0.119	1.492E-2
10.000	0.325	Fixed cumulative value 10	0.649	0.079	0.214	3.214E-2
0.047	0.084	Minimum training presence	0.996	0.000	0.000	3.351E-1
11.806	0.444	10 percentile training presence	0.625	0.087	0.238	3.38E-2
42.514	0.532	Equal training sensitivity and specificity	0.367	0.365	0.381	3.507E-4
18.045	0.455	Maximum training sensitivity plus specificity	0.563	0.127	0.238	4.677E-3

42.514	0.532	Equal test sensitivity and specificity	0.367	0.365	0.381	3.507E-4
48.268	0.537	Maximum test sensitivity plus specificity	0.326	0.413	0.381	2.489E-5
1.593	0.176	Balance training omission, predicted area and threshold value	0.860	0.008	0.000	4.521E-3
3.579	0.267	Equate entropy of thresholded and original distributions	0.784	0.024	0.095	2.893E-2

Click here to interactively explore this prediction using the Explain tool. If clicking from your browser does not succeed in starting the tool, try running the script in C:\Users\Bill\Desktop\Maxent Outputs NEC 7-8-15\NEC_0_explain.bat directly. This tool requires the environmental grids to be small enough that they all fit in memory.

Response curves

These curves show how each environmental variable affects the Maxent prediction. The curves show how the logistic prediction changes as each environmental variable is varied, keeping all other environmental variables at their average sample value. Click on a response curve to see a larger version. Note that the curves can be hard to interpret if you have strongly correlated variables, as the model may depend on the correlations in ways that are not evident in the curves. In other words, the curves show the marginal effect of changing exactly one variable, whereas the model may take advantage of sets of variables changing together.

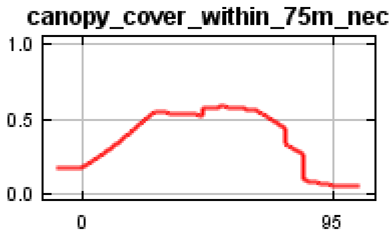

In contrast to the above marginal response curves, each of the following curves represents a different model, namely, a Maxent model created using only the corresponding variable. These plots reflect the dependence of predicted suitability both on the selected variable and on dependencies induced by correlations between the selected variable and other variables. They may be easier to interpret if there are strong correlations between variables.

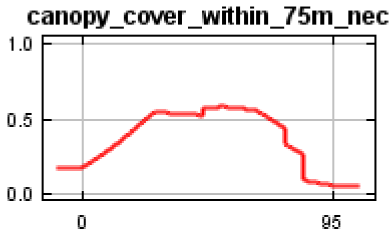

Analysis of variable contributions

The following table gives estimates of relative contributions of the environmental variables to the Maxent model. To determine the first estimate, in each iteration of the training algorithm, the increase in regularized gain is added to the contribution of the corresponding variable, or subtracted from it if the change to the absolute value of lambda is negative. For the second estimate, for each environmental variable in turn, the values of that variable on training presence and background data are randomly permuted. The model is reevaluated on the permuted data, and the resulting drop in training AUC is shown in the table, normalized to percentages. As with the variable jackknife, variable contributions should be interpreted with caution when the predictor variables are correlated.

Variable	Percent contribution	Permutation importance
canopy_cover_within_75m_nec	100	100

Raw data outputs and control parameters

The data used in the above analysis is contained in the next links. Please see the Help button for more information on these.
The model applied to the training environmental layers
The coefficients of the model
The omission and predicted area for varying cumulative and raw thresholds
The prediction strength at the training and (optionally) test presence sites
Results for all species modeled in the same Maxent run, with summary statistics and (optionally) jackknife results

Regularized training gain is 0.200, training AUC is 0.689, unregularized training gain is 0.249.
Unregularized test gain is 0.079.
Test AUC is 0.646, standard deviation is 0.042 (calculated as in DeLong, DeLong & Clarke-Pearson 1988, equation 2).
Algorithm converged after 280 iterations (1 seconds).

The follow settings were used during the run:
126 presence records used for training, 42 for testing.
10000 points used to determine the Maxent distribution (background points and presence points).
Environmental layers used (all continuous): canopy_cover_within_75m_nec
Regularization values: linear/quadratic/product: 0.050, categorical: 0.250, threshold: 1.000, hinge: 0.500
Feature types used: hinge linear threshold quadratic
responsecurves: true
pictures: false
outputdirectory: C:\Users\Bill\Desktop\Maxent Outputs NEC 7-8-15
samplesfile: C:\Users\Bill\Desktop\Maxent_samples\Compare_NEC_EC_150m_311_WGS_84_m.csv
environmentallayers: C:\Users\Bill\Desktop\Maxent Layers\maxent.cache
randomseed: true
randomtestpoints: 25
replicates: 10
replicatetype: subsample
Command line used:

Maxent model for NEC_1

This page contains some analysis of the Maxent model for NEC_1, created Wed Jul 08 17:41:15 EDT 2015 using Maxent version 3.3.3k. If you would like to do further analyses, the raw data used here is linked to at the end of this page.

Analysis of omission/commission

The following picture shows the omission rate and predicted area as a function of the cumulative threshold. The omission rate is is calculated both on the training presence records, and (if test data are used) on the test records. The omission rate should be close to the predicted omission, because of the definition of the cumulative threshold.

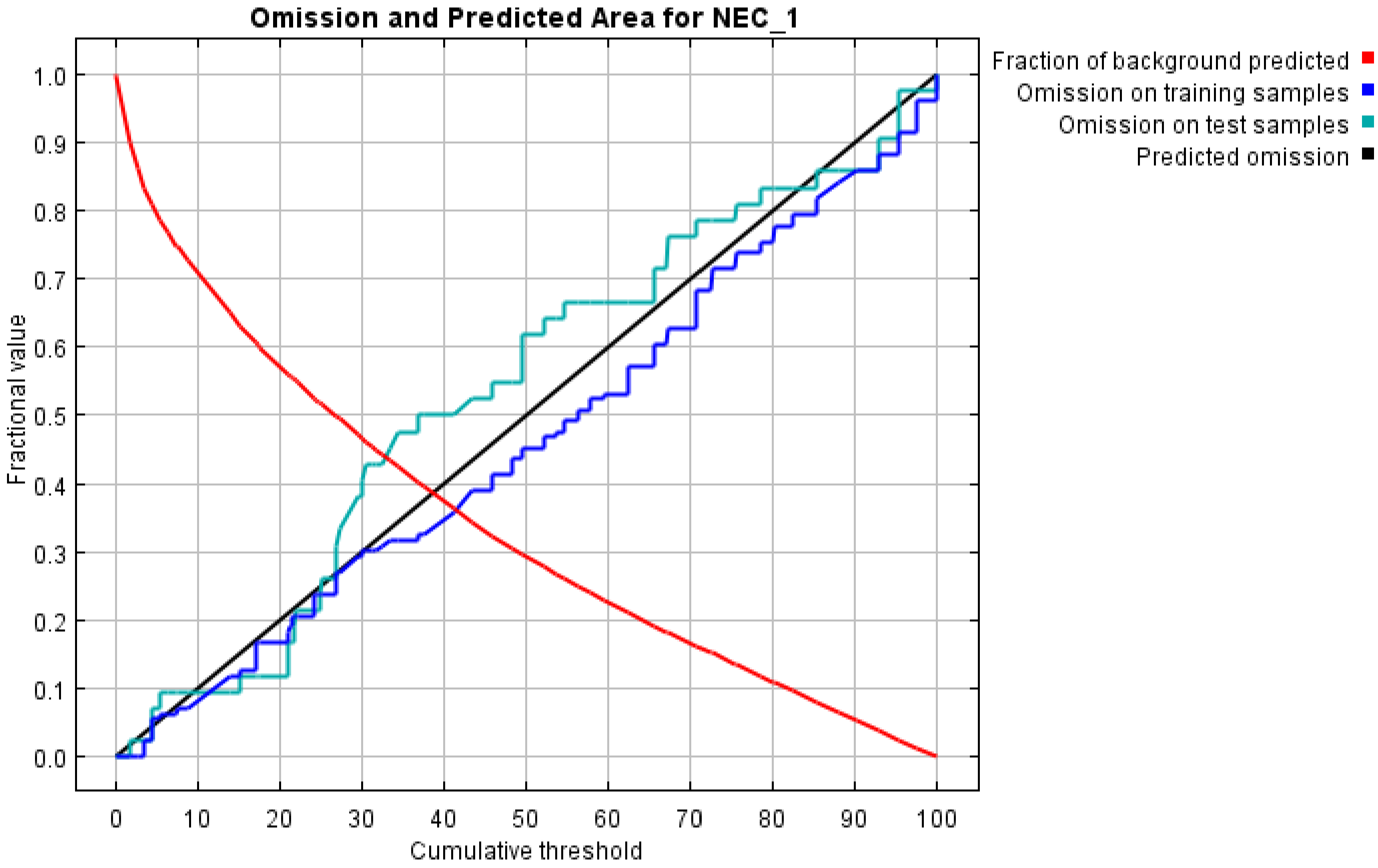

The next picture is the receiver operating characteristic (ROC) curve for the same data. Note that the specificity is defined using predicted area, rather than true commission (see the paper by Phillips, Anderson and Schapire cited on the help page for discussion of what this means). This implies that the maximum achievable AUC is less than 1. If test data is drawn from the Maxent distribution itself, then the maximum possible test AUC would be 0.658 rather than 1; in practice the test AUC may exceed this bound.

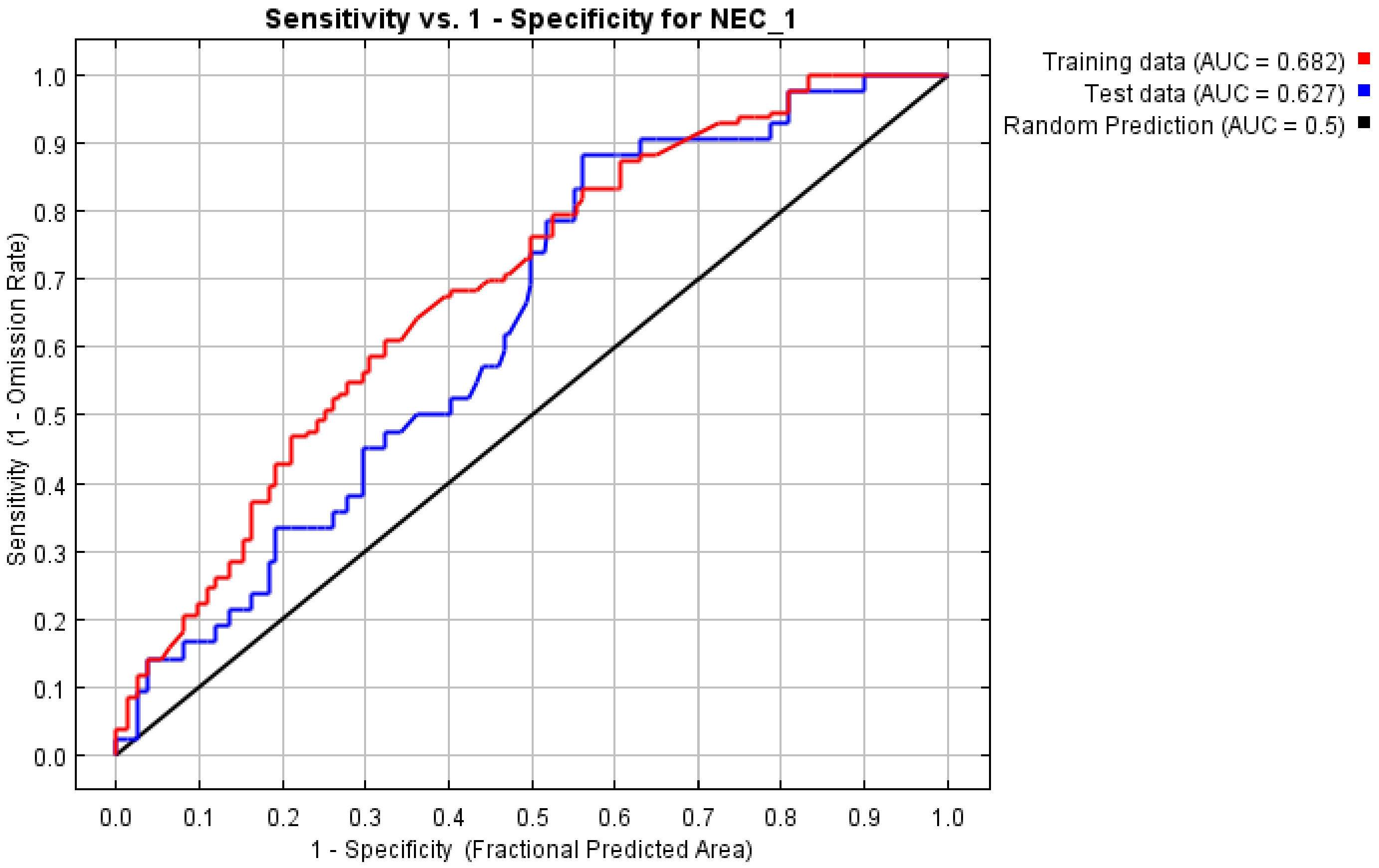

Some common thresholds and corresponding omission rates are as follows. If test data are available, binomial probabilities are calculated exactly if the number of test samples is at most 25, otherwise using a normal approximation to the binomial. These are 1-sided p-values for the null hypothesis that test points are predicted no better than by a random prediction with the same fractional predicted area. The "Balance" threshold minimizes 6 * training omission rate + .04 * cumulative threshold + 1.6 * fractional predicted area.

Cumulative threshold	Logistic threshold	Description	Fractional predicted area	Training omission rate	Test omission rate	P-value
1.000	0.127	Fixed cumulative value 1	0.899	0.000	0.000	1.483E-2
5.000	0.271	Fixed cumulative value 5	0.786	0.056	0.071	1.225E-2
10.000	0.371	Fixed cumulative value 10	0.649	0.119	0.095	2.63E-4
3.520	0.211	Minimum training presence	0.832	0.000	0.024	6.16E-3
8.805	0.359	10 percentile training presence	0.726	0.071	0.095	4.617E-3
41.132	0.495	Equal training sensitivity and specificity	0.363	0.357	0.500	3.231E-2
45.842	0.513	Maximum training sensitivity plus specificity	0.324	0.389	0.524	1.734E-2
		Equal test sensitivity and				4.548E-

32.554	0.473	specificity	0.442	0.310	0.429	2
20.875	0.421	Maximum test sensitivity plus specificity	0.561	0.167	0.119	1.5E-5
3.520	0.211	Balance training omission, predicted area and threshold value	0.832	0.000	0.024	6.16E-3
3.520	0.211	Equate entropy of thresholded and original distributions	0.832	0.000	0.024	6.16E-3

Click here to interactively explore this prediction using the Explain tool. If clicking from your browser does not succeed in starting the tool, try running the script in C:\Users\Bill\Desktop\Maxent Outputs NEC 7-8-15\NEC_1_explain.bat directly. This tool requires the environmental grids to be small enough that they all fit in memory.

Response curves

These curves show how each environmental variable affects the Maxent prediction. The curves show how the logistic prediction changes as each environmental variable is varied, keeping all other environmental variables at their average sample value. Click on a response curve to see a larger version. Note that the curves can be hard to interpret if you have strongly correlated variables, as the model may depend on the correlations in ways that are not evident in the curves. In other words, the curves show the marginal effect of changing exactly one variable, whereas the model may take advantage of sets of variables changing together.

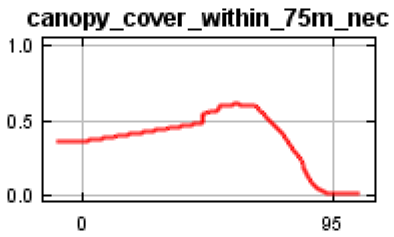

In contrast to the above marginal response curves, each of the following curves represents a different model, namely, a Maxent model created using only the corresponding variable. These plots reflect the dependence of predicted suitability both on the selected variable and on dependencies induced by correlations between the selected variable and other variables. They may be easier to interpret if there are strong correlations between variables.

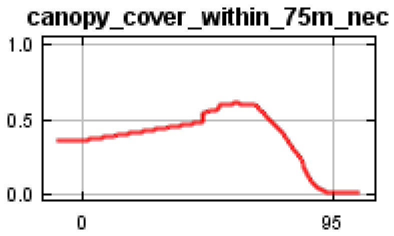

Analysis of variable contributions

The following table gives estimates of relative contributions of the environmental variables to the Maxent model. To determine the first estimate, in each iteration of the training algorithm, the increase in regularized gain is added to the contribution of the corresponding variable, or subtracted from it if the change to the absolute value of lambda is negative. For the second estimate, for each environmental variable in turn, the values of that variable on training presence and background data are randomly permuted. The model is reevaluated on the permuted data, and the resulting drop in training AUC is shown in the table, normalized to percentages. As with the variable jackknife, variable contributions should be interpreted with caution when the predictor variables are correlated.

Variable	Percent contribution	Permutation importance
canopy_cover_within_75m_nec	100	100

Raw data outputs and control parameters

The data used in the above analysis is contained in the next links. Please see the Help button for more information on these.
The model applied to the training environmental layers
The coefficients of the model
The omission and predicted area for varying cumulative and raw thresholds
The prediction strength at the training and (optionally) test presence sites
Results for all species modeled in the same Maxent run, with summary statistics and (optionally) jackknife results

Regularized training gain is 0.168, training AUC is 0.682, unregularized training gain is 0.212.
Unregularized test gain is 0.121.
Test AUC is 0.627, standard deviation is 0.037 (calculated as in DeLong, DeLong & Clarke-Pearson 1988, equation 2).
Algorithm converged after 260 iterations (1 seconds).

The follow settings were used during the run:
126 presence records used for training, 42 for testing.
10000 points used to determine the Maxent distribution (background points and presence points).
Environmental layers used (all continuous): canopy_cover_within_75m_nec
Regularization values: linear/quadratic/product: 0.050, categorical: 0.250, threshold: 1.000, hinge: 0.500
Feature types used: hinge linear threshold quadratic
responsecurves: true
pictures: false
outputdirectory: C:\Users\Bill\Desktop\Maxent Outputs NEC 7-8-15
samplesfile: C:\Users\Bill\Desktop\Maxent_samples\Compare_NEC_EC_150m_311_WGS_84_m.csv
environmentallayers: C:\Users\Bill\Desktop\Maxent Layers\maxent.cache
randomseed: true
randomtestpoints: 25
replicates: 10
replicatetype: subsample
Command line used:

Maxent model for NEC_2

This page contains some analysis of the Maxent model for NEC_2, created Wed Jul 08 17:47:10 EDT 2015 using Maxent version 3.3.3k. If you would like to do further analyses, the raw data used here is linked to at the end of this page.

Analysis of omission/commission

The following picture shows the omission rate and predicted area as a function of the cumulative threshold. The omission rate is is calculated both on the training presence records, and (if test data are used) on the test records. The omission rate should be close to the predicted omission, because of the definition of the cumulative threshold.

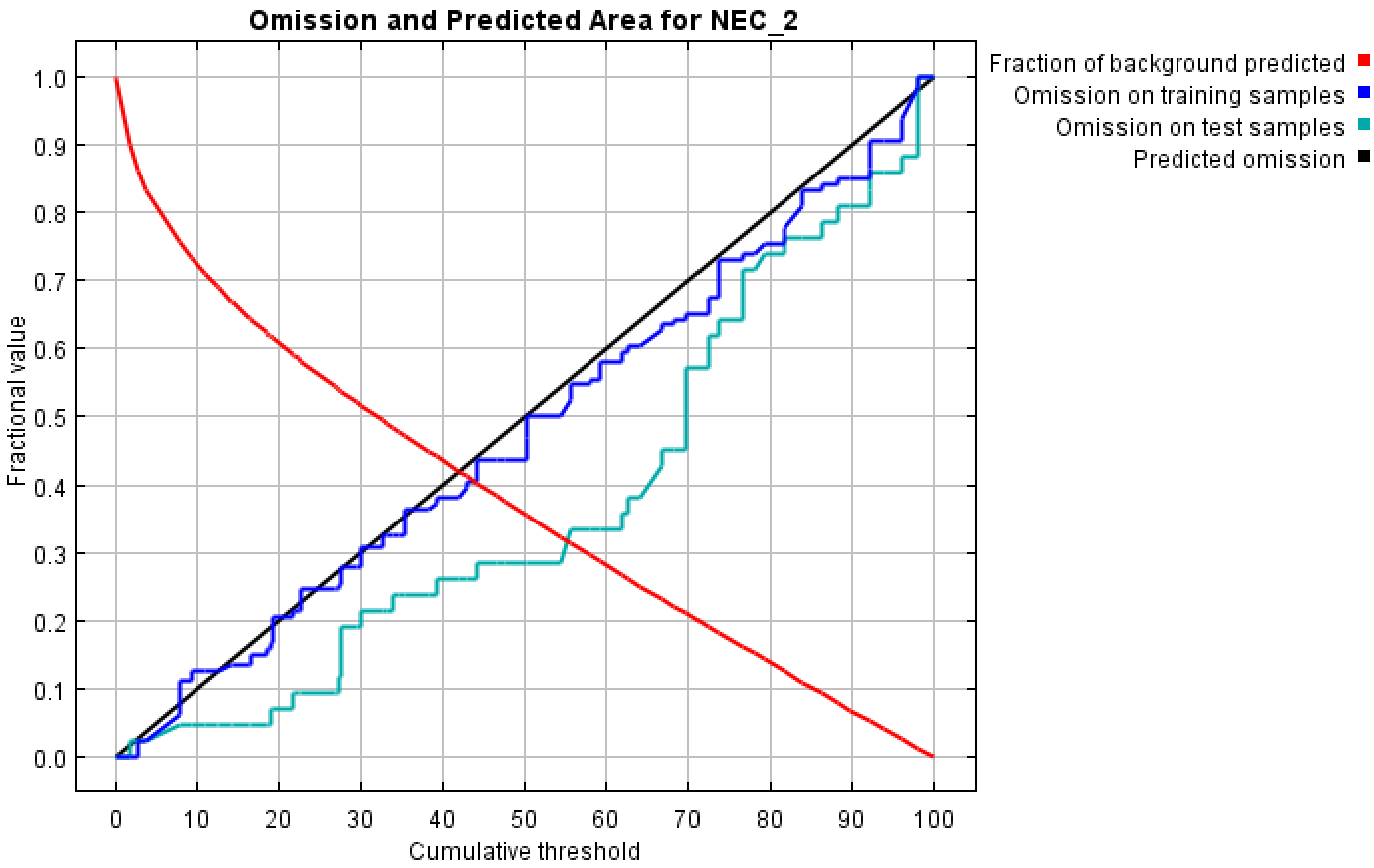

The next picture is the receiver operating characteristic (ROC) curve for the same data. Note that the specificity is defined using predicted area, rather than true commission (see the paper by Phillips, Anderson and Schapire cited on the help page for discussion of what this means). This implies that the maximum achievable AUC is less than 1. If test data is drawn from the Maxent distribution itself, then the maximum possible test AUC would be 0.621 rather than 1; in practice the test AUC may exceed this bound.

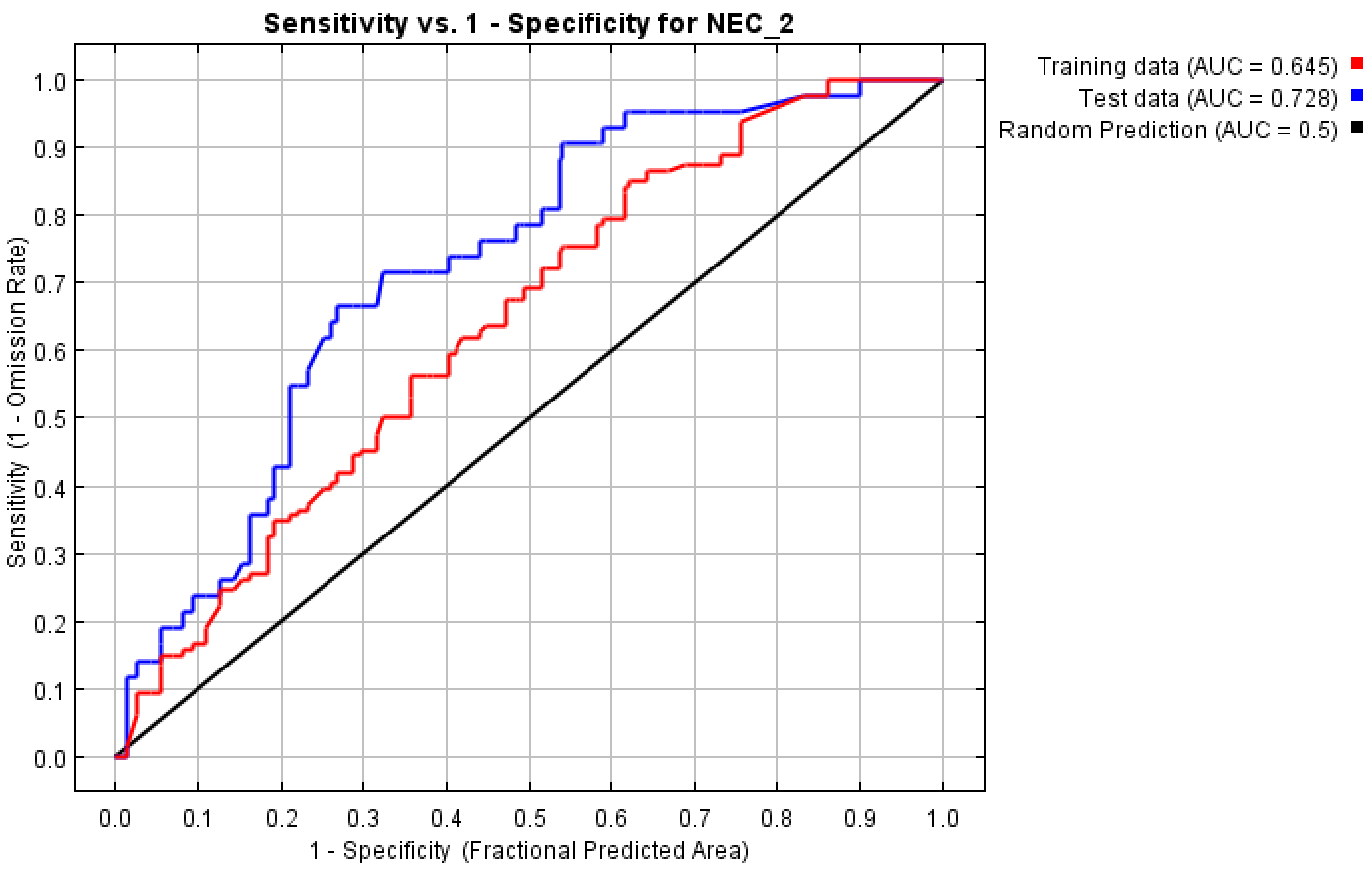

Some common thresholds and corresponding omission rates are as follows. If test data are available, binomial probabilities are calculated exactly if the number of test samples is at most 25, otherwise using a normal approximation to the binomial. These are 1-sided p-values for the null hypothesis that test points are predicted no better than by a random prediction with the same fractional predicted area. The "Balance" threshold minimizes 6 * training omission rate + .04 * cumulative threshold + 1.6 * fractional predicted area.

Cumulative threshold	Logistic threshold	Description	Fractional predicted area	Training omission rate	Test omission rate	P-value
1.000	0.127	Fixed cumulative value 1	0.899	0.000	0.000	1.483E-2
5.000	0.369	Fixed cumulative value 5	0.756	0.063	0.048	1.506E-3
10.000	0.404	Fixed cumulative value 10	0.710	0.127	0.048	2.716E-4
2.617	0.259	Minimum training presence	0.860	0.000	0.024	1.518E-2
7.824	0.369	10 percentile training presence	0.756	0.063	0.048	1.506E-3
44.108	0.533	Equal training sensitivity and specificity	0.403	0.405	0.262	4.768E-6
18.516	0.464	Maximum training sensitivity plus	0.622	0.151	0.048	5.112E-

		specificity				6
55.576	0.541	Equal test sensitivity and specificity	0.315	0.524	0.333	4.815E-7
61.846	0.546	Maximum test sensitivity plus specificity	0.269	0.579	0.333	2.974E-9
2.617	0.259	Balance training omission, predicted area and threshold value	0.860	0.000	0.024	1.518E-2
2.617	0.259	Equate entropy of thresholded and original distributions	0.860	0.000	0.024	1.518E-2

Click here to interactively explore this prediction using the Explain tool. If clicking from your browser does not succeed in starting the tool, try running the script in C:\Users\Bill\Desktop\Maxent Outputs NEC 7-8-15\NEC_2_explain.bat directly. This tool requires the environmental grids to be small enough that they all fit in memory.

Response curves

These curves show how each environmental variable affects the Maxent prediction. The curves show how the logistic prediction changes as each environmental variable is varied, keeping all other environmental variables at their average sample value. Click on a response curve to see a larger version. Note that the curves can be hard to interpret if you have strongly correlated variables, as the model may depend on the correlations in ways that are not evident in the curves. In other words, the curves show the marginal effect of changing exactly one variable, whereas the model may take advantage of sets of variables changing together.

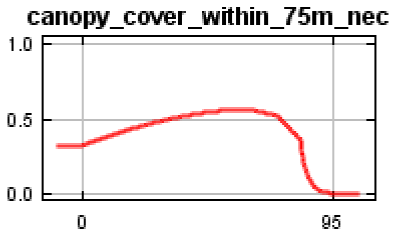

In contrast to the above marginal response curves, each of the following curves represents a different model, namely, a Maxent model created using only the corresponding variable. These plots reflect the dependence of predicted suitability both on the selected variable and on dependencies induced by correlations between the selected variable and other variables. They may be easier to interpret if there are strong correlations between variables.

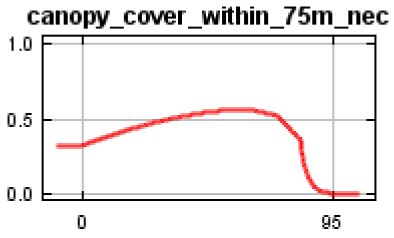

Analysis of variable contributions

The following table gives estimates of relative contributions of the environmental variables to the Maxent model. To determine the first estimate, in each iteration of the training algorithm, the increase in regularized gain is added to the contribution of the corresponding variable, or subtracted from it if the change to the absolute value of lambda is negative. For the second estimate, for each environmental variable in turn, the values of that variable on training presence and background data are randomly permuted. The model is reevaluated on the permuted data, and the resulting drop in training AUC is shown in the table, normalized to percentages. As with the variable jackknife, variable contributions should be interpreted with caution when the predictor variables are correlated.

Variable	Percent contribution	Permutation importance
canopy_cover_within_75m_nec	100	100

Raw data outputs and control parameters

The data used in the above analysis is contained in the next links. Please see the Help button for more information on these.
The model applied to the training environmental layers
The coefficients of the model
The omission and predicted area for varying cumulative and raw thresholds
The prediction strength at the training and (optionally) test presence sites
Results for all species modeled in the same Maxent run, with summary statistics and (optionally) jackknife results

Regularized training gain is 0.122, training AUC is 0.645, unregularized training gain is 0.152.
Unregularized test gain is 0.223.
Test AUC is 0.728, standard deviation is 0.034 (calculated as in DeLong, DeLong & Clarke-Pearson 1988, equation 2).
Algorithm converged after 380 iterations (1 seconds).

The follow settings were used during the run:
126 presence records used for training, 42 for testing.
10000 points used to determine the Maxent distribution (background points and presence points).
Environmental layers used (all continuous): canopy_cover_within_75m_nec
Regularization values: linear/quadratic/product: 0.050, categorical: 0.250, threshold: 1.000, hinge: 0.500
Feature types used: hinge linear threshold quadratic
responsecurves: true
pictures: false
outputdirectory: C:\Users\Bill\Desktop\Maxent Outputs NEC 7-8-15
samplesfile: C:\Users\Bill\Desktop\Maxent_samples\Compare_NEC_EC_150m_311_WGS_84_m.csv
environmentallayers: C:\Users\Bill\Desktop\Maxent Layers\maxent.cache
randomseed: true
randomtestpoints: 25
replicates: 10
replicatetype: subsample
Command line used:

Maxent model for NEC_3

This page contains some analysis of the Maxent model for NEC_3, created Wed Jul 08 17:52:58 EDT 2015 using Maxent version 3.3.3k. If you would like to do further analyses, the raw data used here is linked to at the end of this page.

Analysis of omission/commission

The following picture shows the omission rate and predicted area as a function of the cumulative threshold. The omission rate is is calculated both on the training presence records, and (if test data are used) on the test records. The omission rate should be close to the predicted omission, because of the definition of the cumulative threshold.

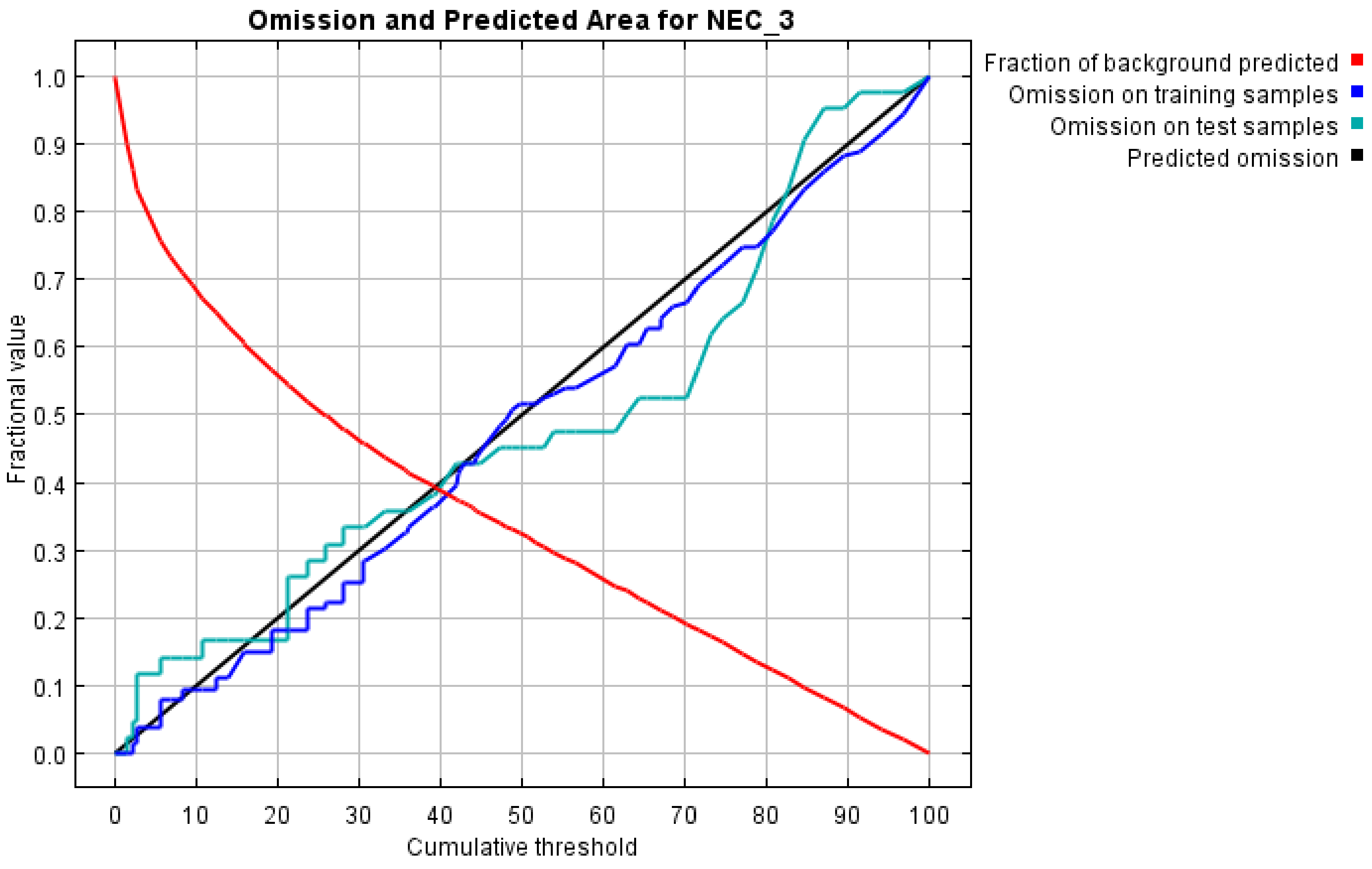

The next picture is the receiver operating characteristic (ROC) curve for the same data. Note that the specificity is defined using predicted area, rather than true commission (see the paper by Phillips, Anderson and Schapire cited on the help page for discussion of what this means). This implies that the maximum achievable AUC is less than 1. If test data is drawn from the Maxent distribution itself, then the maximum possible test AUC would be 0.650 rather than 1; in practice the test AUC may exceed this bound.

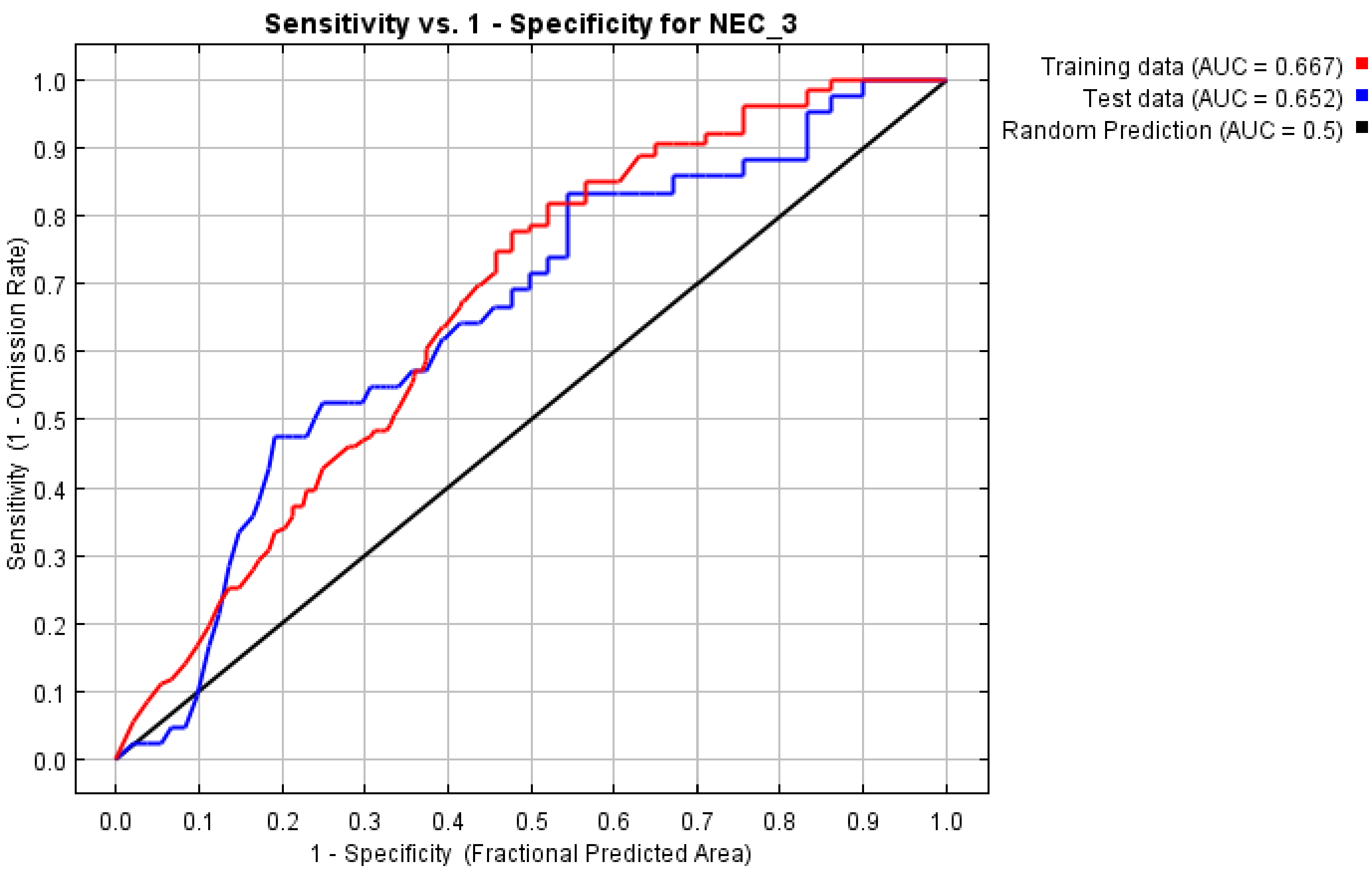

Some common thresholds and corresponding omission rates are as follows. If test data are available, binomial probabilities are calculated exactly if the number of test samples is at most 25, otherwise using a normal approximation to the binomial. These are 1-sided p-values for the null hypothesis that test points are predicted no better than by a random prediction with the same fractional predicted area. The "Balance" threshold minimizes 6 * training omission rate + .04 * cumulative threshold + 1.6 * fractional predicted area.

**Cumulative threshold**	**Logistic threshold**	**Description**	**Fractional predicted area**	**Training omission rate**	**Test omission rate**	**P-value**
1.000	0.115	Fixed cumulative value 1	0.899	0.000	0.000	1.483E-2
5.000	0.319	Fixed cumulative value 5	0.756	0.040	0.119	2.943E-2
10.000	0.362	Fixed cumulative value 10	0.671	0.095	0.143	5.192E-3
2.198	0.128	Minimum training presence	0.860	0.000	0.024	1.518E-2
12.320	0.391	10 percentile training presence	0.649	0.095	0.167	6.263E-3
42.071	0.564	Equal training sensitivity and specificity	0.375	0.397	0.429	4.202E-3
28.139	0.483	Maximum training sensitivity plus	0.478	0.222	0.310	2.908E-

		specificity				3
39.256	0.559	Equal test sensitivity and specificity	0.393	0.365	0.381	1.366E-3
21.249	0.436	Maximum test sensitivity plus specificity	0.544	0.183	0.167	8.334E-5
2.198	0.128	Balance training omission, predicted area and threshold value	0.860	0.000	0.024	1.518E-2
2.704	0.241	Equate entropy of thresholded and original distributions	0.832	0.016	0.048	1.829E-2

Click here to interactively explore this prediction using the Explain tool. If clicking from your browser does not succeed in starting the tool, try running the script in C:\Users\Bill\Desktop\Maxent Outputs NEC 7-8-15\NEC_3_explain.bat directly. This tool requires the environmental grids to be small enough that they all fit in memory.

Response curves

These curves show how each environmental variable affects the Maxent prediction. The curves show how the logistic prediction changes as each environmental variable is varied, keeping all other environmental variables at their average sample value. Click on a response curve to see a larger version. Note that the curves can be hard to interpret if you have strongly correlated variables, as the model may depend on the correlations in ways that are not evident in the curves. In other words, the curves show the marginal effect of changing exactly one variable, whereas the model may take advantage of sets of variables changing together.

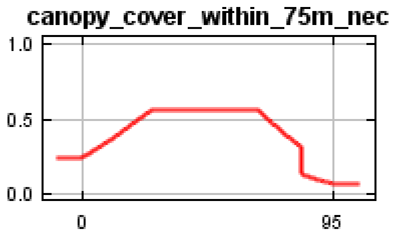

In contrast to the above marginal response curves, each of the following curves represents a different model, namely, a Maxent model created using only the corresponding variable. These plots reflect the dependence of predicted suitability both on the selected variable and on dependencies induced by correlations between the selected variable and other variables. They may be easier to interpret if there are strong correlations between variables.

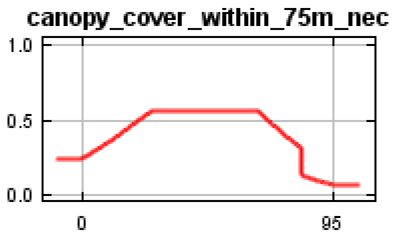

Analysis of variable contributions

The following table gives estimates of relative contributions of the environmental variables to the Maxent model. To determine the first estimate, in each iteration of the training algorithm, the increase in regularized gain is added to the contribution of the corresponding variable, or subtracted from it if the change to the absolute value of lambda is negative. For the second estimate, for each environmental variable in turn, the values of that variable on training presence and background data are randomly permuted. The model is reevaluated on the permuted data, and the resulting drop in training AUC is shown in the table, normalized to percentages. As with the variable jackknife, variable contributions should be interpreted with caution when the predictor variables are correlated.

Variable	Percent contribution	Permutation importance
canopy_cover_within_75m_nec	100	100

Raw data outputs and control parameters

The data used in the above analysis is contained in the next links. Please see the Help button for more information on these.
The model applied to the training environmental layers
The coefficients of the model
The omission and predicted area for varying cumulative and raw thresholds
The prediction strength at the training and (optionally) test presence sites
Results for all species modeled in the same Maxent run, with summary statistics and (optionally) jackknife results

Regularized training gain is 0.175, training AUC is 0.667, unregularized training gain is 0.211.
Unregularized test gain is 0.101.
Test AUC is 0.652, standard deviation is 0.040 (calculated as in DeLong, DeLong & Clarke-Pearson 1988, equation 2).
Algorithm converged after 160 iterations (0 seconds).

The follow settings were used during the run:
126 presence records used for training, 42 for testing.
10000 points used to determine the Maxent distribution (background points and presence points).
Environmental layers used (all continuous): canopy_cover_within_75m_nec
Regularization values: linear/quadratic/product: 0.050, categorical: 0.250, threshold: 1.000, hinge: 0.500
Feature types used: hinge linear threshold quadratic
responsecurves: true
pictures: false
outputdirectory: C:\Users\Bill\Desktop\Maxent Outputs NEC 7-8-15
samplesfile: C:\Users\Bill\Desktop\Maxent_samples\Compare_NEC_EC_150m_311_WGS_84_m.csv
environmentallayers: C:\Users\Bill\Desktop\Maxent Layers\maxent.cache
randomseed: true
randomtestpoints: 25
replicates: 10
replicatetype: subsample
Command line used:

Maxent model for NEC_4

This page contains some analysis of the Maxent model for NEC_4, created Wed Jul 08 17:58:44 EDT 2015 using Maxent version 3.3.3k. If you would like to do further analyses, the raw data used here is linked to at the end of this page.

Analysis of omission/commission

The following picture shows the omission rate and predicted area as a function of the cumulative threshold. The omission rate is is calculated both on the training presence records, and (if test data are used) on the test records. The omission rate should be close to the predicted omission, because of the definition of the cumulative threshold.

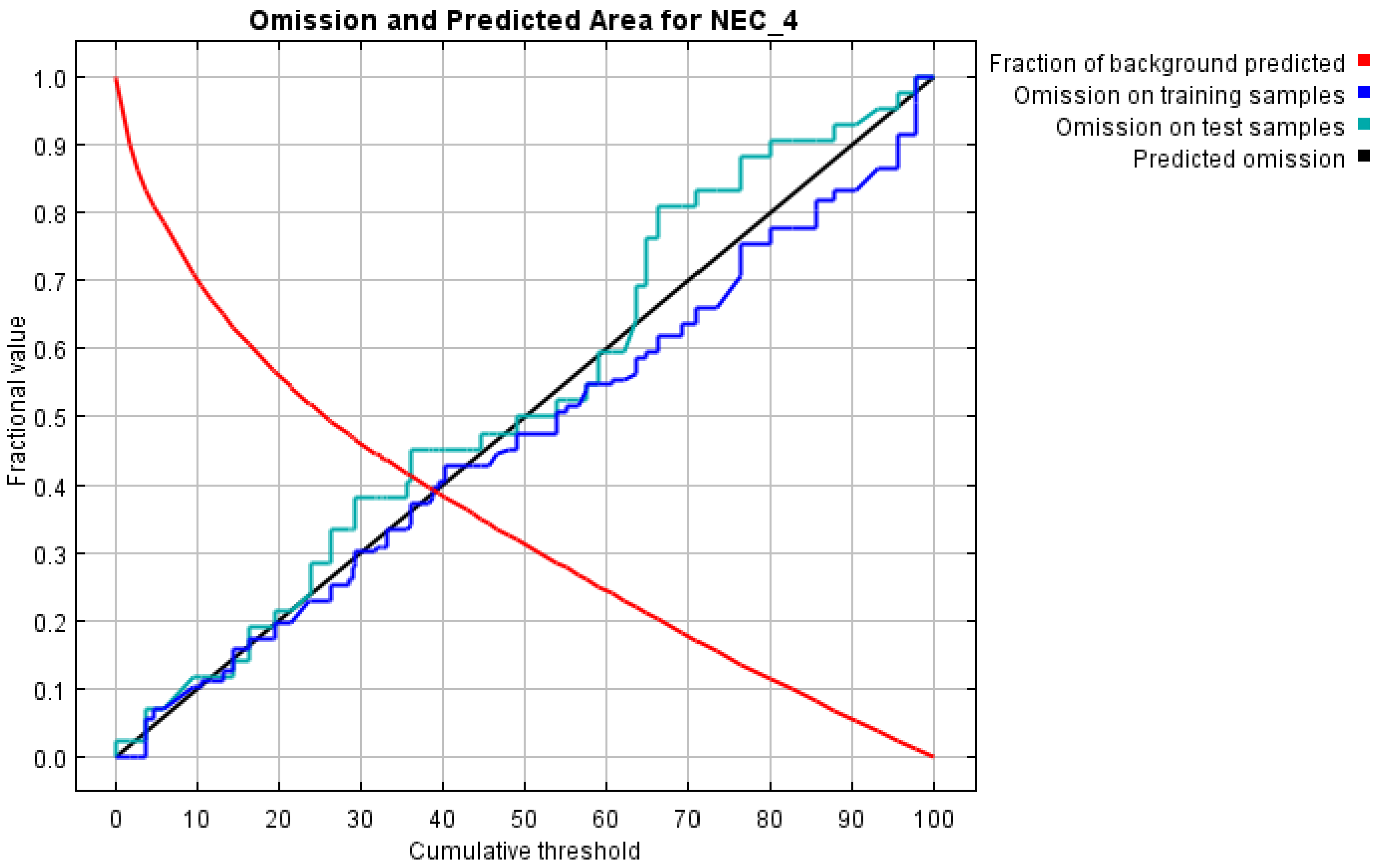

The next picture is the receiver operating characteristic (ROC) curve for the same data. Note that the specificity is defined using predicted area, rather than true commission (see the paper by Phillips, Anderson and Schapire cited on the help page for discussion of what this means). This implies that the maximum achievable AUC is less than 1. If test data is drawn from the Maxent distribution itself, then the maximum possible test AUC would be 0.653 rather than 1; in practice the test AUC may exceed this bound.

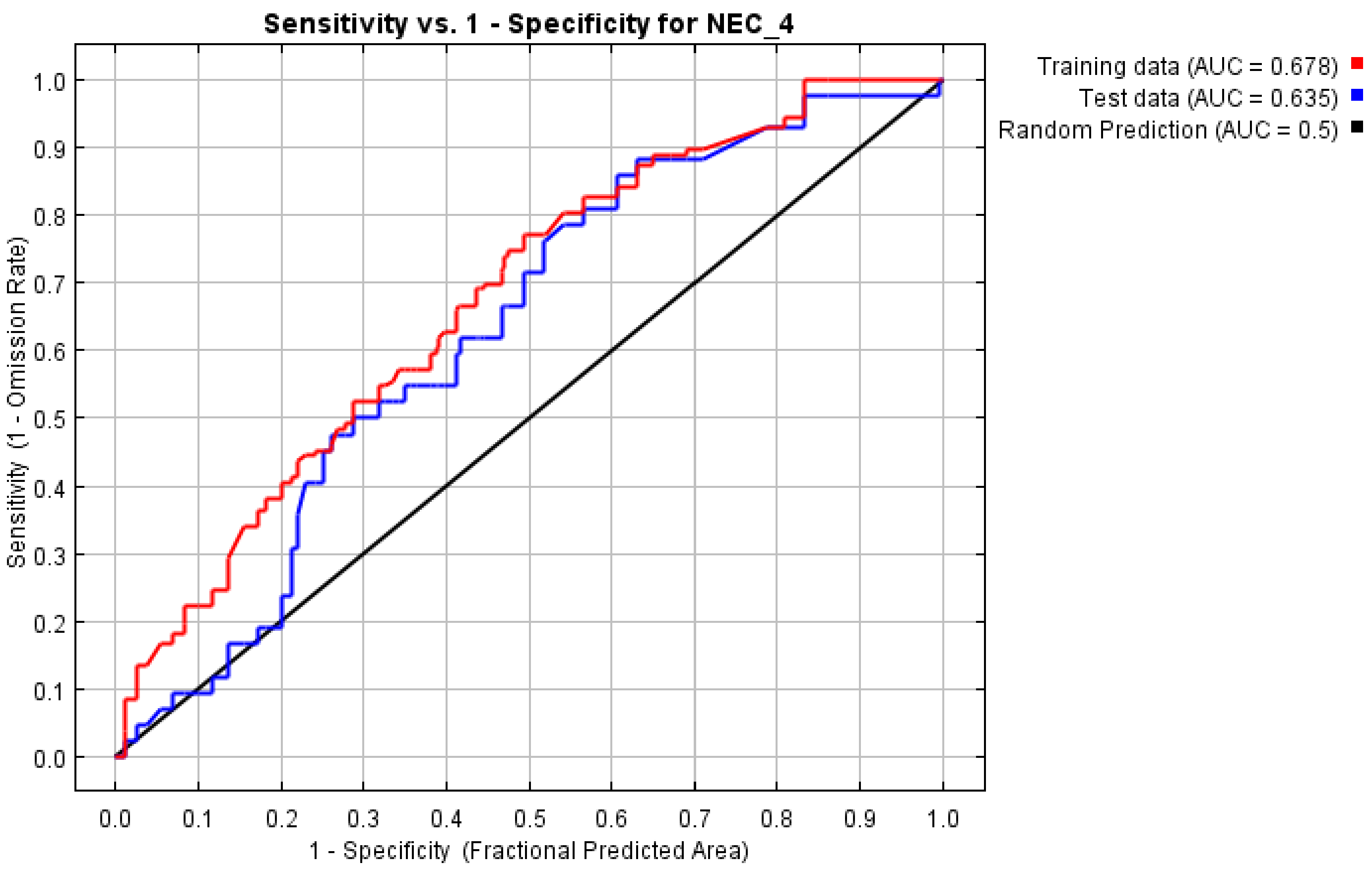

Some common thresholds and corresponding omission rates are as follows. If test data are available, binomial probabilities are calculated exactly if the number of test samples is at most 25, otherwise using a normal approximation to the binomial. These are 1-sided p-values for the null hypothesis that test points are predicted no better than by a random prediction with the same fractional predicted area. The "Balance" threshold minimizes 6 * training omission rate + .04 * cumulative threshold + 1.6 * fractional predicted area.

Cumulative threshold	Logistic threshold	Description	Fractional predicted area	Training omission rate	Test omission rate	P-value
1.000	0.172	Fixed cumulative value 1	0.899	0.000	0.024	4.816E-2
5.000	0.298	Fixed cumulative value 5	0.786	0.071	0.071	1.225E-2
10.000	0.330	Fixed cumulative value 10	0.691	0.103	0.119	3.833E-3
3.625	0.232	Minimum training presence	0.832	0.000	0.024	6.16E-3
5.764	0.298	10 percentile training presence	0.786	0.071	0.071	1.225E-2
38.845	0.536	Equal training sensitivity and specificity	0.392	0.397	0.452	1.936E-2
26.316	0.484	Maximum training sensitivity plus specificity	0.494	0.230	0.286	2.149E-3

36.040	0.529	Equal test sensitivity and specificity	0.413	0.341	0.405	8.227E-3
16.249	0.406	Maximum test sensitivity plus specificity	0.606	0.159	0.143	4.292E-4
3.625	0.232	Balance training omission, predicted area and threshold value	0.832	0.000	0.024	6.16E-3
3.625	0.232	Equate entropy of thresholded and original distributions	0.832	0.000	0.024	6.16E-3

Click here to interactively explore this prediction using the Explain tool. If clicking from your browser does not succeed in starting the tool, try running the script in C:\Users\Bill\Desktop\Maxent Outputs NEC 7-8-15\NEC_4_explain.bat directly. This tool requires the environmental grids to be small enough that they all fit in memory.

Response curves

These curves show how each environmental variable affects the Maxent prediction. The curves show how the logistic prediction changes as each environmental variable is varied, keeping all other environmental variables at their average sample value. Click on a response curve to see a larger version. Note that the curves can be hard to interpret if you have strongly correlated variables, as the model may depend on the correlations in ways that are not evident in the curves. In other words, the curves show the marginal effect of changing exactly one variable, whereas the model may take advantage of sets of variables changing together.

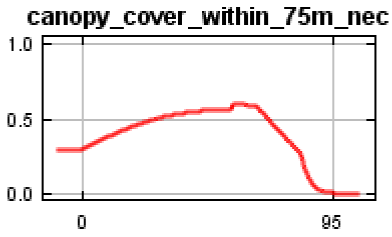

In contrast to the above marginal response curves, each of the following curves represents a different model, namely, a Maxent model created using only the corresponding variable. These plots reflect the dependence of predicted suitability both on the selected variable and on dependencies induced by correlations between the selected variable and other variables. They may be easier to interpret if there are strong correlations between variables.

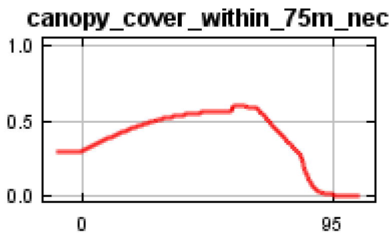

Analysis of variable contributions

The following table gives estimates of relative contributions of the environmental variables to the Maxent model. To determine the first estimate, in each iteration of the training algorithm, the increase in regularized gain is added to the contribution of the corresponding variable, or subtracted from it if the change to the absolute value of lambda is negative. For the second estimate, for each environmental variable in turn, the values of that variable on training presence and background data are randomly permuted. The model is reevaluated on the permuted data, and the resulting drop in training AUC is shown in the table, normalized to percentages. As with the variable jackknife, variable contributions should be interpreted with caution when the predictor variables are correlated.

Variable	Percent contribution	Permutation importance
canopy_cover_within_75m_nec	100	100

Raw data outputs and control parameters

The data used in the above analysis is contained in the next links. Please see the Help button for more information on these.
The model applied to the training environmental layers
The coefficients of the model
The omission and predicted area for varying cumulative and raw thresholds
The prediction strength at the training and (optionally) test presence sites
Results for all species modeled in the same Maxent run, with summary statistics and (optionally) jackknife results

Regularized training gain is 0.165, training AUC is 0.678, unregularized training gain is 0.203.
Unregularized test gain is 0.139.
Test AUC is 0.635, standard deviation is 0.036 (calculated as in DeLong, DeLong & Clarke-Pearson 1988, equation 2).
Algorithm converged after 420 iterations (1 seconds).

The follow settings were used during the run:
126 presence records used for training, 42 for testing.
10000 points used to determine the Maxent distribution (background points and presence points).
Environmental layers used (all continuous): canopy_cover_within_75m_nec
Regularization values: linear/quadratic/product: 0.050, categorical: 0.250, threshold: 1.000, hinge: 0.500
Feature types used: hinge linear threshold quadratic
responsecurves: true
pictures: false
outputdirectory: C:\Users\Bill\Desktop\Maxent Outputs NEC 7-8-15
samplesfile: C:\Users\Bill\Desktop\Maxent_samples\Compare_NEC_EC_150m_311_WGS_84_m.csv
environmentallayers: C:\Users\Bill\Desktop\Maxent Layers\maxent.cache
randomseed: true
randomtestpoints: 25
replicates: 10
replicatetype: subsample
Command line used:

Maxent model for NEC_5

This page contains some analysis of the Maxent model for NEC_5, created Wed Jul 08 18:04:37 EDT 2015 using Maxent version 3.3.3k. If you would like to do further analyses, the raw data used here is linked to at the end of this page.

Analysis of omission/commission

The following picture shows the omission rate and predicted area as a function of the cumulative threshold. The omission rate is is calculated both on the training presence records, and (if test data are used) on the test records. The omission rate should be close to the predicted omission, because of the definition of the cumulative threshold.

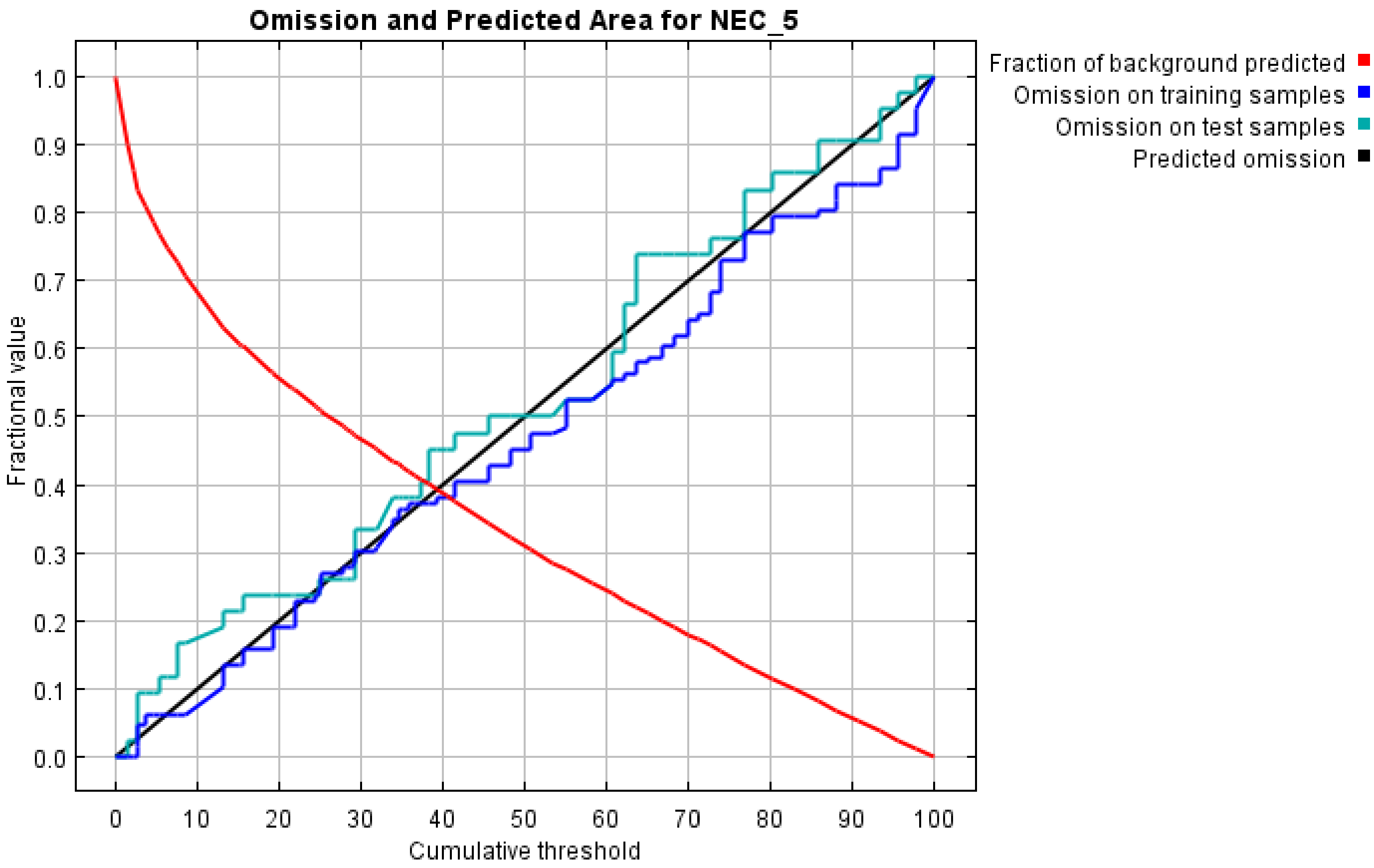

The next picture is the receiver operating characteristic (ROC) curve for the same data. Note that the specificity is defined using predicted area, rather than true commission (see the paper by Phillips, Anderson and Schapire cited on the help page for discussion of what this means). This implies that the maximum achievable AUC is less than 1. If test data is drawn from the Maxent distribution itself, then the maximum possible test AUC would be 0.655 rather than 1; in practice the test AUC may exceed this bound.

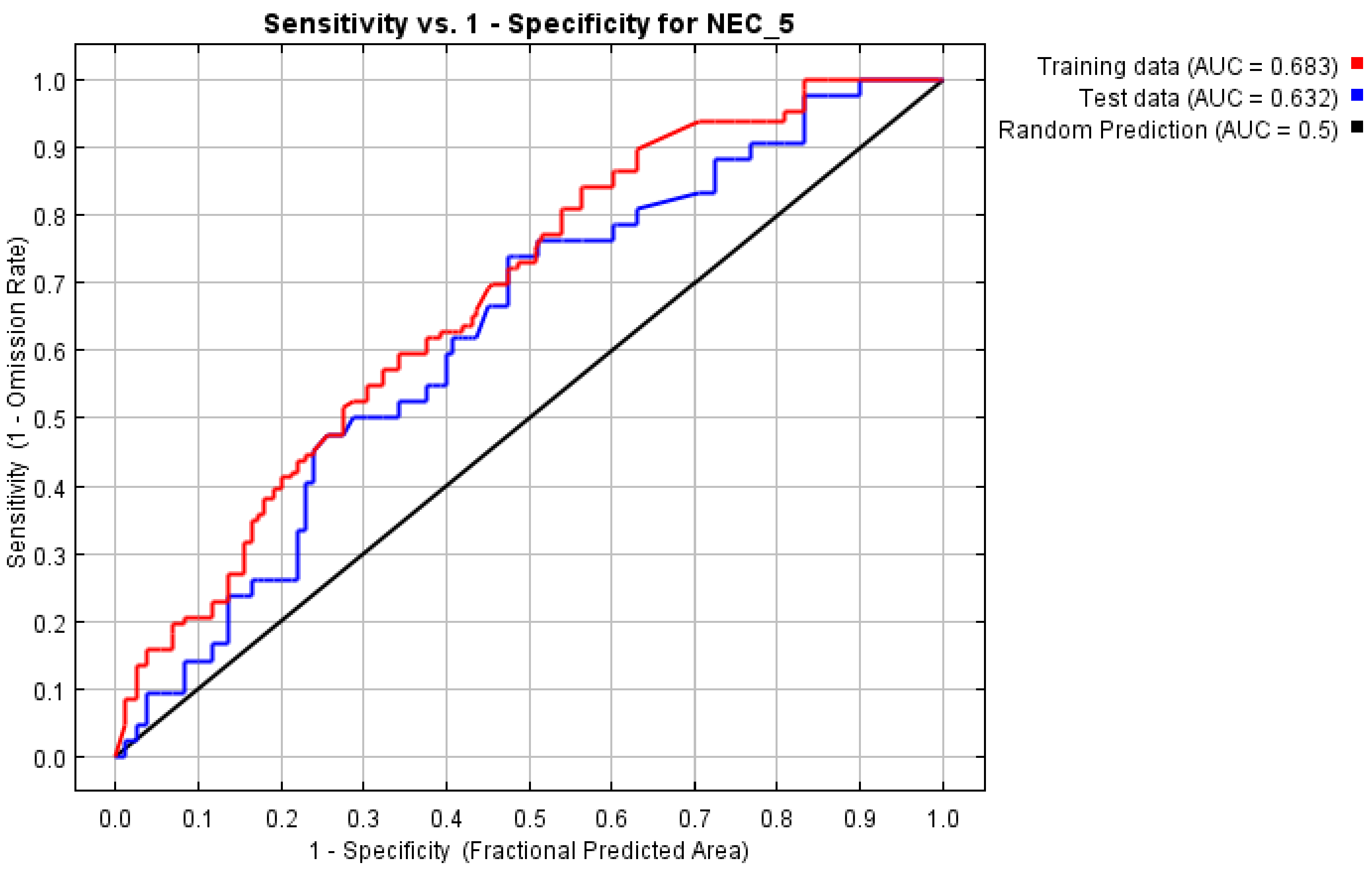

Some common thresholds and corresponding omission rates are as follows. If test data are available, binomial probabilities are calculated exactly if the number of test samples is at most 25, otherwise using a normal approximation to the binomial. These are 1-sided p-values for the null hypothesis that test points are predicted no better than by a random prediction with the same fractional predicted area. The "Balance" threshold minimizes 6 * training omission rate + .04 * cumulative threshold + 1.6 * fractional predicted area.

**Cumulative threshold**	**Logistic threshold**	**Description**	**Fractional predicted area**	**Training omission rate**	**Test omission rate**	**P-value**
1.000	0.115	Fixed cumulative value 1	0.899	0.000	0.000	1.483E-2
5.000	0.286	Fixed cumulative value 5	0.767	0.063	0.095	1.724E-2
10.000	0.431	Fixed cumulative value 10	0.630	0.103	0.190	7.955E-3
2.707	0.129	Minimum training presence	0.832	0.000	0.024	6.16E-3
8.536	0.332	10 percentile training presence	0.706	0.063	0.167	3.495E-2
40.504	0.518	Equal training sensitivity and specificity	0.384	0.381	0.452	1.451E-2
19.302	0.462	Maximum training sensitivity plus specificity	0.564	0.159	0.238	4.78E-3

37.423	0.516	Equal test sensitivity and specificity	0.408	0.373	0.405	6.747E-3
29.247	0.506	Maximum test sensitivity plus specificity	0.474	0.286	0.262	2.984E-4
2.707	0.129	Balance training omission, predicted area and threshold value	0.832	0.000	0.024	6.16E-3
2.707	0.129	Equate entropy of thresholded and original distributions	0.832	0.000	0.024	6.16E-3

Click here to interactively explore this prediction using the Explain tool. If clicking from your browser does not succeed in starting the tool, try running the script in C:\Users\Bill\Desktop\Maxent Outputs NEC 7-8-15\NEC_5_explain.bat directly. This tool requires the environmental grids to be small enough that they all fit in memory.

Response curves

These curves show how each environmental variable affects the Maxent prediction. The curves show how the logistic prediction changes as each environmental variable is varied, keeping all other environmental variables at their average sample value. Click on a response curve to see a larger version. Note that the curves can be hard to interpret if you have strongly correlated variables, as the model may depend on the correlations in ways that are not evident in the curves. In other words, the curves show the marginal effect of changing exactly one variable, whereas the model may take advantage of sets of variables changing together.

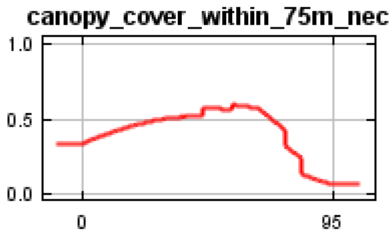

In contrast to the above marginal response curves, each of the following curves represents a different model, namely, a Maxent model created using only the corresponding variable. These plots reflect the dependence of predicted suitability both on the selected variable and on dependencies induced by correlations between the selected variable and other variables. They may be easier to interpret if there are strong correlations between variables.

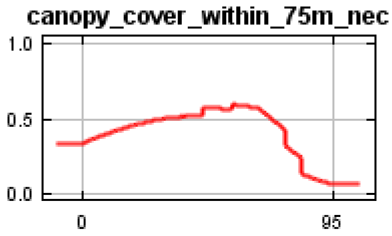

Analysis of variable contributions

The following table gives estimates of relative contributions of the environmental variables to the Maxent model. To determine the first estimate, in each iteration of the training algorithm, the increase in regularized gain is added to the contribution of the corresponding variable, or subtracted from it if the change to the absolute value of lambda is negative. For the second estimate, for each environmental variable in turn, the values of that variable on training presence and background data are randomly permuted. The model is reevaluated on the permuted data, and the resulting drop in training AUC is shown in the table, normalized to percentages. As with the variable jackknife, variable contributions should be interpreted with caution when the predictor variables are correlated.

Variable	Percent contribution	Permutation importance
canopy_cover_within_75m_nec	100	100

Raw data outputs and control parameters

The data used in the above analysis is contained in the next links. Please see the Help button for more information on these.
The model applied to the training environmental layers
The coefficients of the model
The omission and predicted area for varying cumulative and raw thresholds
The prediction strength at the training and (optionally) test presence sites
Results for all species modeled in the same Maxent run, with summary statistics and (optionally) jackknife results

Regularized training gain is 0.178, training AUC is 0.683, unregularized training gain is 0.224.
Unregularized test gain is 0.103.
Test AUC is 0.632, standard deviation is 0.039 (calculated as in DeLong, DeLong & Clarke-Pearson 1988, equation 2).
Algorithm converged after 400 iterations (1 seconds).

The follow settings were used during the run:
126 presence records used for training, 42 for testing.
10000 points used to determine the Maxent distribution (background points and presence points).
Environmental layers used (all continuous): canopy_cover_within_75m_nec
Regularization values: linear/quadratic/product: 0.050, categorical: 0.250, threshold: 1.000, hinge: 0.500
Feature types used: hinge linear threshold quadratic
responsecurves: true
pictures: false
outputdirectory: C:\Users\Bill\Desktop\Maxent Outputs NEC 7-8-15
samplesfile: C:\Users\Bill\Desktop\Maxent_samples\Compare_NEC_EC_150m_311_WGS_84_m.csv
environmentallayers: C:\Users\Bill\Desktop\Maxent Layers\maxent.cache
randomseed: true
randomtestpoints: 25
replicates: 10
replicatetype: subsample
Command line used:

Maxent model for NEC_6

This page contains some analysis of the Maxent model for NEC_6, created Wed Jul 08 18:38:41 EDT 2015 using Maxent version 3.3.3k. If you would like to do further analyses, the raw data used here is linked to at the end of this page.

Analysis of omission/commission

The following picture shows the omission rate and predicted area as a function of the cumulative threshold. The omission rate is is calculated both on the training presence records, and (if test data are used) on the test records. The omission rate should be close to the predicted omission, because of the definition of the cumulative threshold.

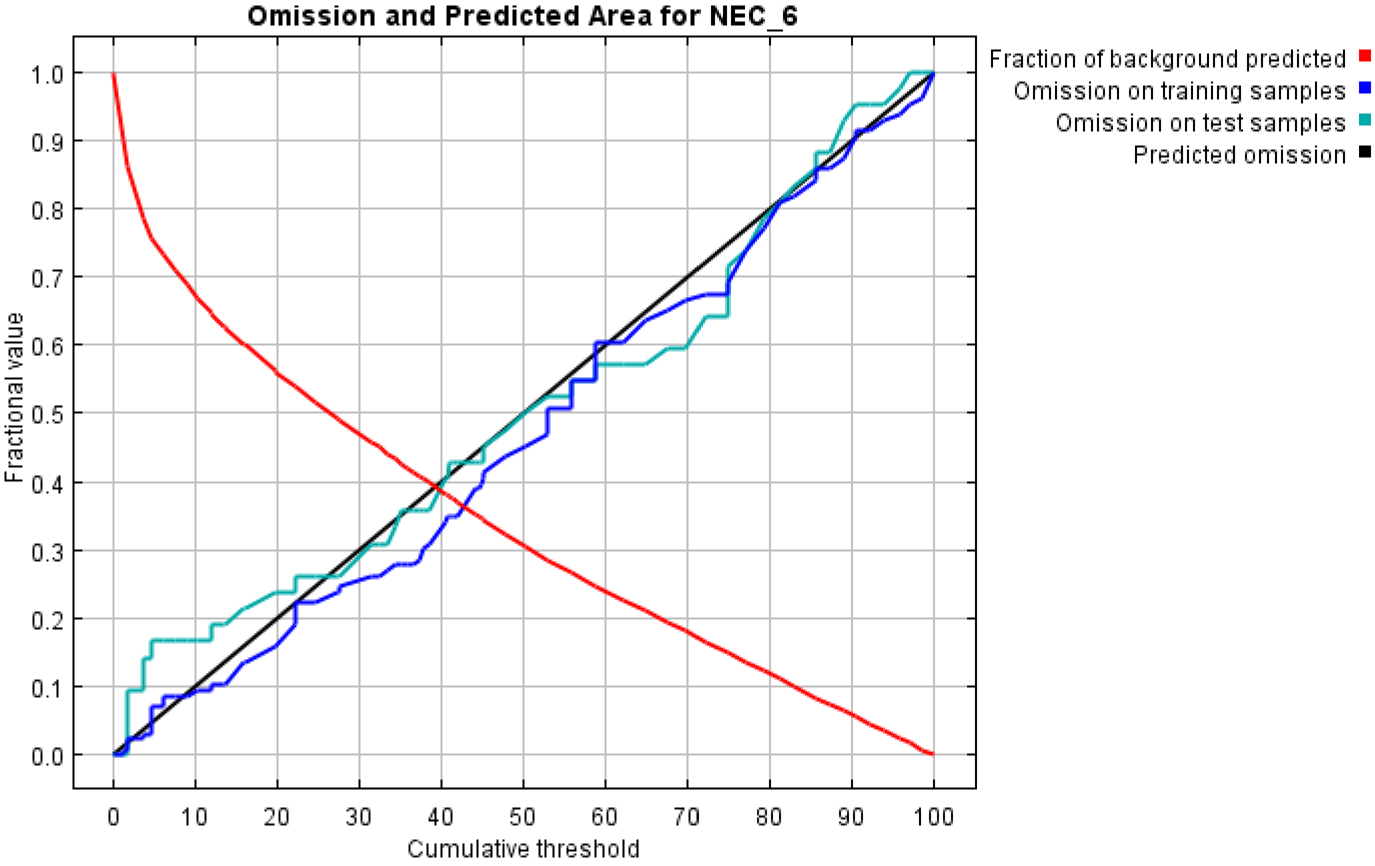

The next picture is the receiver operating characteristic (ROC) curve for the same data. Note that the specificity is defined using predicted area, rather than true commission (see the paper by Phillips, Anderson and Schapire cited on the help page for discussion of what this means). This implies that the maximum achievable AUC is less than 1. If test data is drawn from the Maxent distribution itself, then the maximum possible test AUC would be 0.657 rather than 1; in practice the test AUC may exceed this bound.

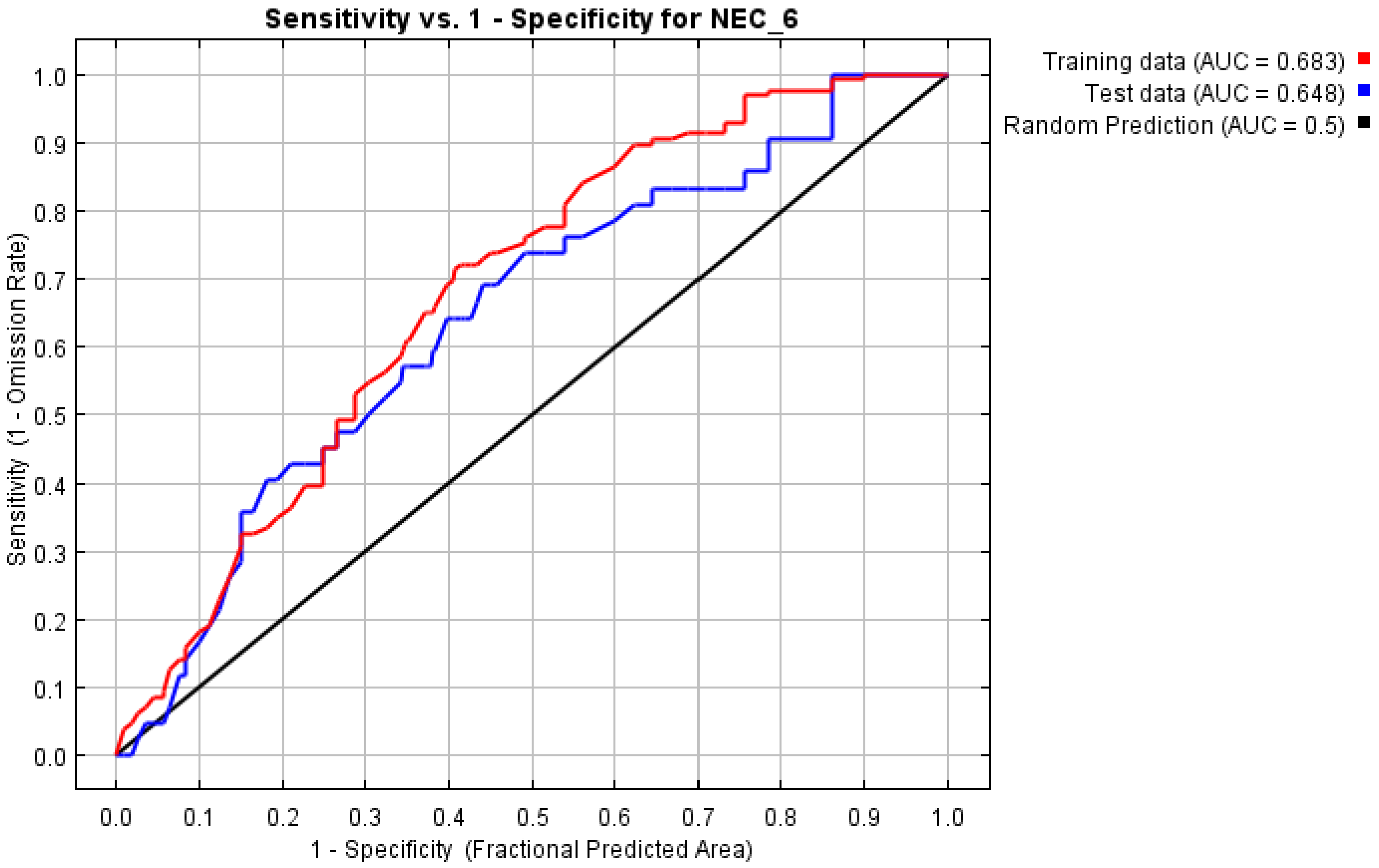

Some common thresholds and corresponding omission rates are as follows. If test data are available, binomial probabilities are calculated exactly if the number of test samples is at most 25, otherwise using a normal approximation to the binomial. These are 1-sided p-values for the null hypothesis that test points are predicted no better than by a random prediction with the same fractional predicted area. The "Balance" threshold minimizes 6 * training omission rate + .04 * cumulative threshold + 1.6 * fractional predicted area.

Cumulative threshold	Logistic threshold	Description	Fractional predicted area	Training omission rate	Test omission rate	P-value
1.000	0.085	Fixed cumulative value 1	0.899	0.000	0.000	1.483E-2
5.000	0.342	Fixed cumulative value 5	0.733	0.071	0.167	7.011E-2
10.000	0.376	Fixed cumulative value 10	0.669	0.095	0.167	1.192E-2
1.136	0.085	Minimum training presence	0.899	0.000	0.000	1.483E-2
11.980	0.390	10 percentile training presence	0.646	0.095	0.167	5.6E-3
41.868	0.502	Equal training sensitivity and specificity	0.371	0.349	0.429	3.617E-3
36.691	0.498	Maximum training sensitivity plus specificity	0.414	0.278	0.357	1.307E-3

40.448	0.500	Equal test sensitivity and specificity	0.383	0.341	0.405	2.321E-3
33.451	0.496	Maximum test sensitivity plus specificity	0.441	0.270	0.310	5.726E-4
1.136	0.085	Balance training omission, predicted area and threshold value	0.899	0.000	0.000	1.483E-2
3.600	0.243	Equate entropy of thresholded and original distributions	0.784	0.024	0.095	2.893E-2

Click here to interactively explore this prediction using the Explain tool. If clicking from your browser does not succeed in starting the tool, try running the script in C:\Users\Bill\Desktop\Maxent Outputs NEC 7-8-15\NEC_6_explain.bat directly. This tool requires the environmental grids to be small enough that they all fit in memory.

Response curves

These curves show how each environmental variable affects the Maxent prediction. The curves show how the logistic prediction changes as each environmental variable is varied, keeping all other environmental variables at their average sample value. Click on a response curve to see a larger version. Note that the curves can be hard to interpret if you have strongly correlated variables, as the model may depend on the correlations in ways that are not evident in the curves. In other words, the curves show the marginal effect of changing exactly one variable, whereas the model may take advantage of sets of variables changing together.

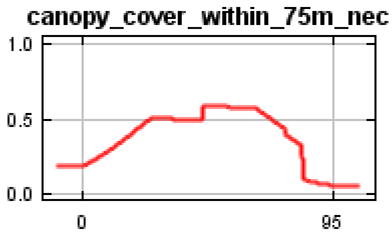

In contrast to the above marginal response curves, each of the following curves represents a different model, namely, a Maxent model created using only the corresponding variable. These plots reflect the dependence of predicted suitability both on the selected variable and on dependencies induced by correlations between the selected variable and other variables. They may be easier to interpret if there are strong correlations between variables.

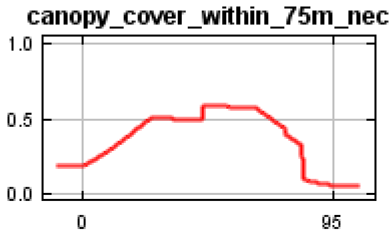

Analysis of variable contributions

The following table gives estimates of relative contributions of the environmental variables to the Maxent model. To determine the first estimate, in each iteration of the training algorithm, the increase in regularized gain is added to the contribution of the corresponding variable, or subtracted from it if the change to the absolute value of lambda is negative. For the second estimate, for each environmental variable in turn, the values of that variable on training presence and background data are randomly permuted. The model is reevaluated on the permuted data, and the resulting drop in training AUC is shown in the table, normalized to percentages. As with the variable jackknife, variable contributions should be interpreted with caution when the predictor variables are correlated.

Variable	Percent contribution	Permutation importance
canopy_cover_within_75m_nec	100	100

Raw data outputs and control parameters

The data used in the above analysis is contained in the next links. Please see the Help button for more information on these.
The model applied to the training environmental layers
The coefficients of the model
The omission and predicted area for varying cumulative and raw thresholds
The prediction strength at the training and (optionally) test presence sites
Results for all species modeled in the same Maxent run, with summary statistics and (optionally) jackknife results

Regularized training gain is 0.189, training AUC is 0.683, unregularized training gain is 0.238.
Unregularized test gain is 0.106.
Test AUC is 0.648, standard deviation is 0.040 (calculated as in DeLong, DeLong & Clarke-Pearson 1988, equation 2).
Algorithm converged after 140 iterations (0 seconds).

The follow settings were used during the run:
126 presence records used for training, 42 for testing.
10000 points used to determine the Maxent distribution (background points and presence points).
Environmental layers used (all continuous): canopy_cover_within_75m_nec
Regularization values: linear/quadratic/product: 0.050, categorical: 0.250, threshold: 1.000, hinge: 0.500
Feature types used: hinge linear threshold quadratic
responsecurves: true
pictures: false
outputdirectory: C:\Users\Bill\Desktop\Maxent Outputs NEC 7-8-15
samplesfile: C:\Users\Bill\Desktop\Maxent_samples\Compare_NEC_EC_150m_311_WGS_84_m.csv
environmentallayers: C:\Users\Bill\Desktop\Maxent Layers\maxent.cache
randomseed: true
randomtestpoints: 25
replicates: 10
replicatetype: subsample
Command line used:

Maxent model for NEC_7

This page contains some analysis of the Maxent model for NEC_7, created Wed Jul 08 18:44:44 EDT 2015 using Maxent version 3.3.3k. If you would like to do further analyses, the raw data used here is linked to at the end of this page.

Analysis of omission/commission

The following picture shows the omission rate and predicted area as a function of the cumulative threshold. The omission rate is is calculated both on the training presence records, and (if test data are used) on the test records. The omission rate should be close to the predicted omission, because of the definition of the cumulative threshold.

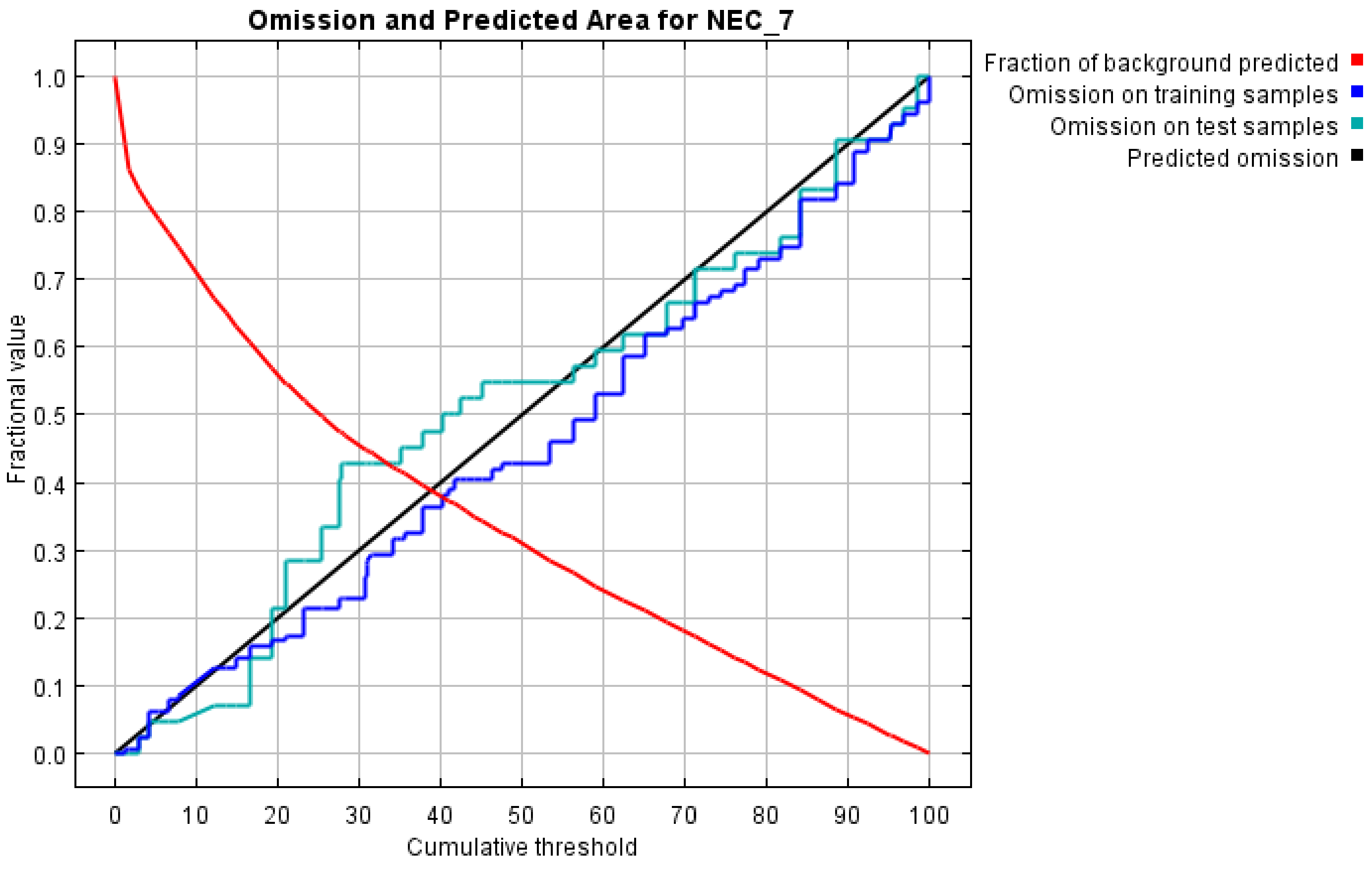

The next picture is the receiver operating characteristic (ROC) curve for the same data. Note that the specificity is defined using predicted area, rather than true commission (see the paper by Phillips, Anderson and Schapire cited on the help page for discussion of what this means). This implies that the maximum achievable AUC is less than 1. If test data is drawn from the Maxent distribution itself, then the maximum possible test AUC would be 0.654 rather than 1; in practice the test AUC may exceed this bound.

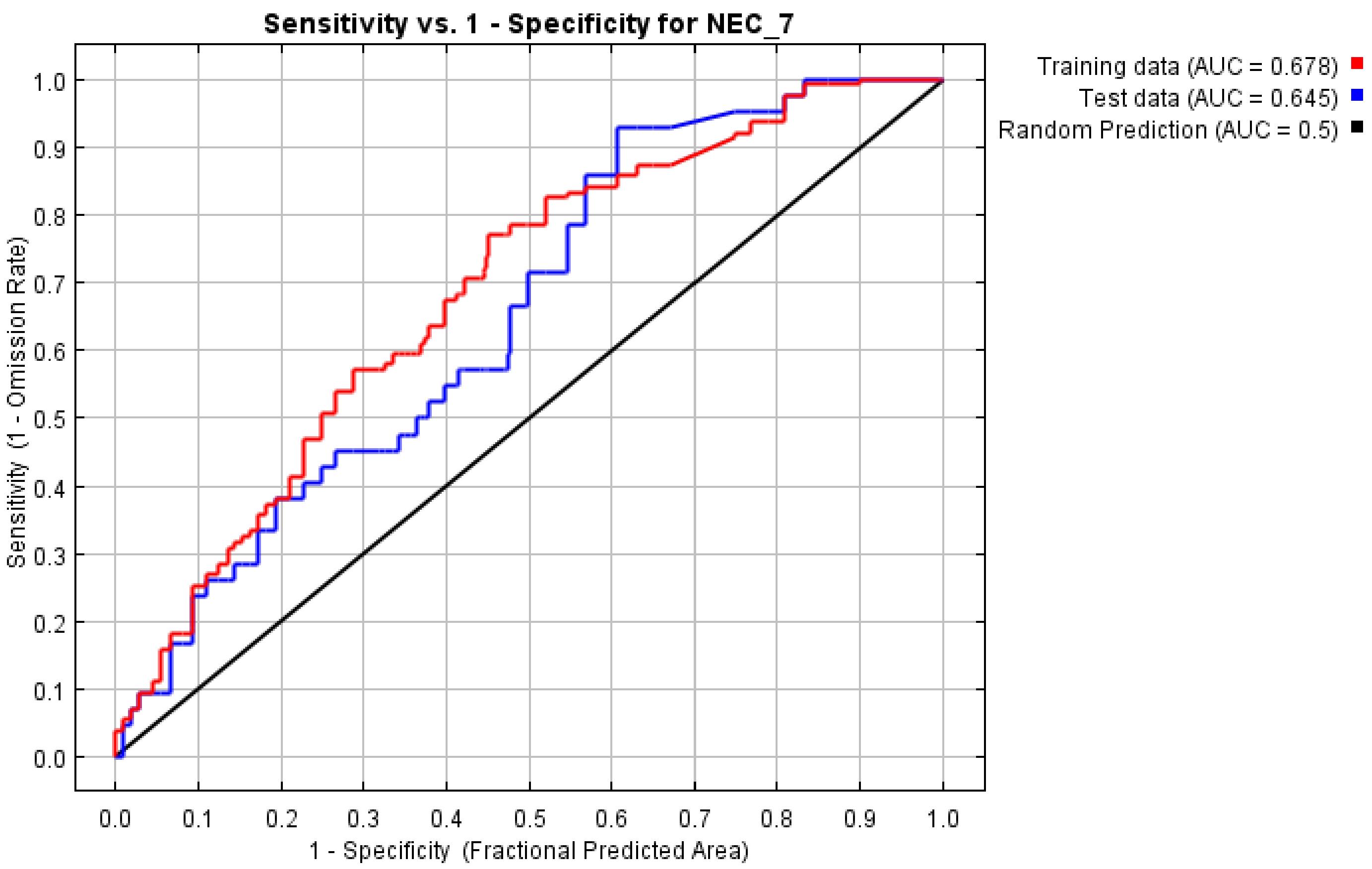

Some common thresholds and corresponding omission rates are as follows. If test data are available, binomial probabilities are calculated exactly if the number of test samples is at most 25, otherwise using a normal approximation to the binomial. These are 1-sided p-values for the null hypothesis that test points are predicted no better than by a random prediction with the same fractional predicted area. The "Balance" threshold minimizes 6 * training omission rate + .04 * cumulative threshold + 1.6 * fractional predicted area.

Cumulative threshold	Logistic threshold	Description	Fractional predicted area	Training omission rate	Test omission rate	P-value
1.000	0.086	Fixed cumulative value 1	0.899	0.000	0.000	1.483E-2
5.000	0.322	Fixed cumulative value 5	0.789	0.063	0.048	4.744E-3
10.000	0.341	Fixed cumulative value 10	0.671	0.127	0.071	1.938E-4
1.129	0.086	Minimum training presence	0.899	0.000	0.000	1.483E-2
7.716	0.331	10 percentile training presence	0.747	0.087	0.048	1.117E-3
40.319	0.536	Equal training sensitivity and specificity	0.378	0.381	0.500	5.194E-2
30.731	0.508	Maximum training sensitivity plus	0.450	0.230	0.429	5.669E-

		specificity				2
34.228	0.522	Equal test sensitivity and specificity	0.423	0.294	0.429	2.568E-2
16.509	0.369	Maximum test sensitivity plus specificity	0.606	0.143	0.071	9.323E-6
1.129	0.086	Balance training omission, predicted area and threshold value	0.899	0.000	0.000	1.483E-2
3.022	0.295	Equate entropy of thresholded and original distributions	0.832	0.008	0.000	1.777E-3

Click here to interactively explore this prediction using the Explain tool. If clicking from your browser does not succeed in starting the tool, try running the script in C:\Users\Bill\Desktop\Maxent Outputs NEC 7-8-15\NEC_7_explain.bat directly. This tool requires the environmental grids to be small enough that they all fit in memory.

Response curves

These curves show how each environmental variable affects the Maxent prediction. The curves show how the logistic prediction changes as each environmental variable is varied, keeping all other environmental variables at their average sample value. Click on a response curve to see a larger version. Note that the curves can be hard to interpret if you have strongly correlated variables, as the model may depend on the correlations in ways that are not evident in the curves. In other words, the curves show the marginal effect of changing exactly one variable, whereas the model may take advantage of sets of variables changing together.

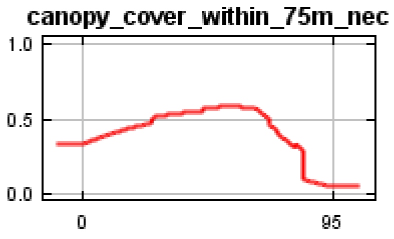

In contrast to the above marginal response curves, each of the following curves represents a different model, namely, a Maxent model created using only the corresponding variable. These plots reflect the dependence of predicted suitability both on the selected variable and on dependencies induced by correlations between the selected variable and other variables. They may be easier to interpret if there are strong correlations between variables.

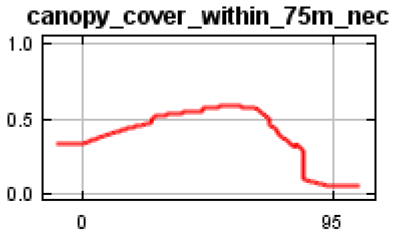

Analysis of variable contributions

The following table gives estimates of relative contributions of the environmental variables to the Maxent model. To determine the first estimate, in each iteration of the training algorithm, the increase in regularized gain is added to the contribution of the corresponding variable, or subtracted from it if the change to the absolute value of lambda is negative. For the second estimate, for each environmental variable in turn, the values of that variable on training presence and background data are randomly permuted. The model is reevaluated on the permuted data, and the resulting drop in training AUC is shown in the table, normalized to percentages. As with the variable jackknife, variable contributions should be interpreted with caution when the predictor variables are correlated.

Variable	Percent contribution	Permutation importance
canopy_cover_within_75m_nec	100	100

Raw data outputs and control parameters

The data used in the above analysis is contained in the next links. Please see the Help button for more information on these.
The model applied to the training environmental layers
The coefficients of the model
The omission and predicted area for varying cumulative and raw thresholds
The prediction strength at the training and (optionally) test presence sites
Results for all species modeled in the same Maxent run, with summary statistics and (optionally) jackknife results

Regularized training gain is 0.178, training AUC is 0.678, unregularized training gain is 0.223.
Unregularized test gain is 0.151.
Test AUC is 0.645, standard deviation is 0.036 (calculated as in DeLong, DeLong & Clarke-Pearson 1988, equation 2).
Algorithm terminated after 500 iterations (1 seconds).

The follow settings were used during the run:
126 presence records used for training, 42 for testing.
10000 points used to determine the Maxent distribution (background points and presence points).
Environmental layers used (all continuous): canopy_cover_within_75m_nec
Regularization values: linear/quadratic/product: 0.050, categorical: 0.250, threshold: 1.000, hinge: 0.500
Feature types used: hinge linear threshold quadratic
responsecurves: true
pictures: false
outputdirectory: C:\Users\Bill\Desktop\Maxent Outputs NEC 7-8-15
samplesfile: C:\Users\Bill\Desktop\Maxent_samples\Compare_NEC_EC_150m_311_WGS_84_m.csv
environmentallayers: C:\Users\Bill\Desktop\Maxent Layers\maxent.cache
randomseed: true
randomtestpoints: 25
replicates: 10
replicatetype: subsample
Command line used:

Maxent model for NEC_8

This page contains some analysis of the Maxent model for NEC_8, created Wed Jul 08 18:50:37 EDT 2015 using Maxent version 3.3.3k. If you would like to do further analyses, the raw data used here is linked to at the end of this page.

Analysis of omission/commission

The following picture shows the omission rate and predicted area as a function of the cumulative threshold. The omission rate is is calculated both on the training presence records, and (if test data are used) on the test records. The omission rate should be close to the predicted omission, because of the definition of the cumulative threshold.

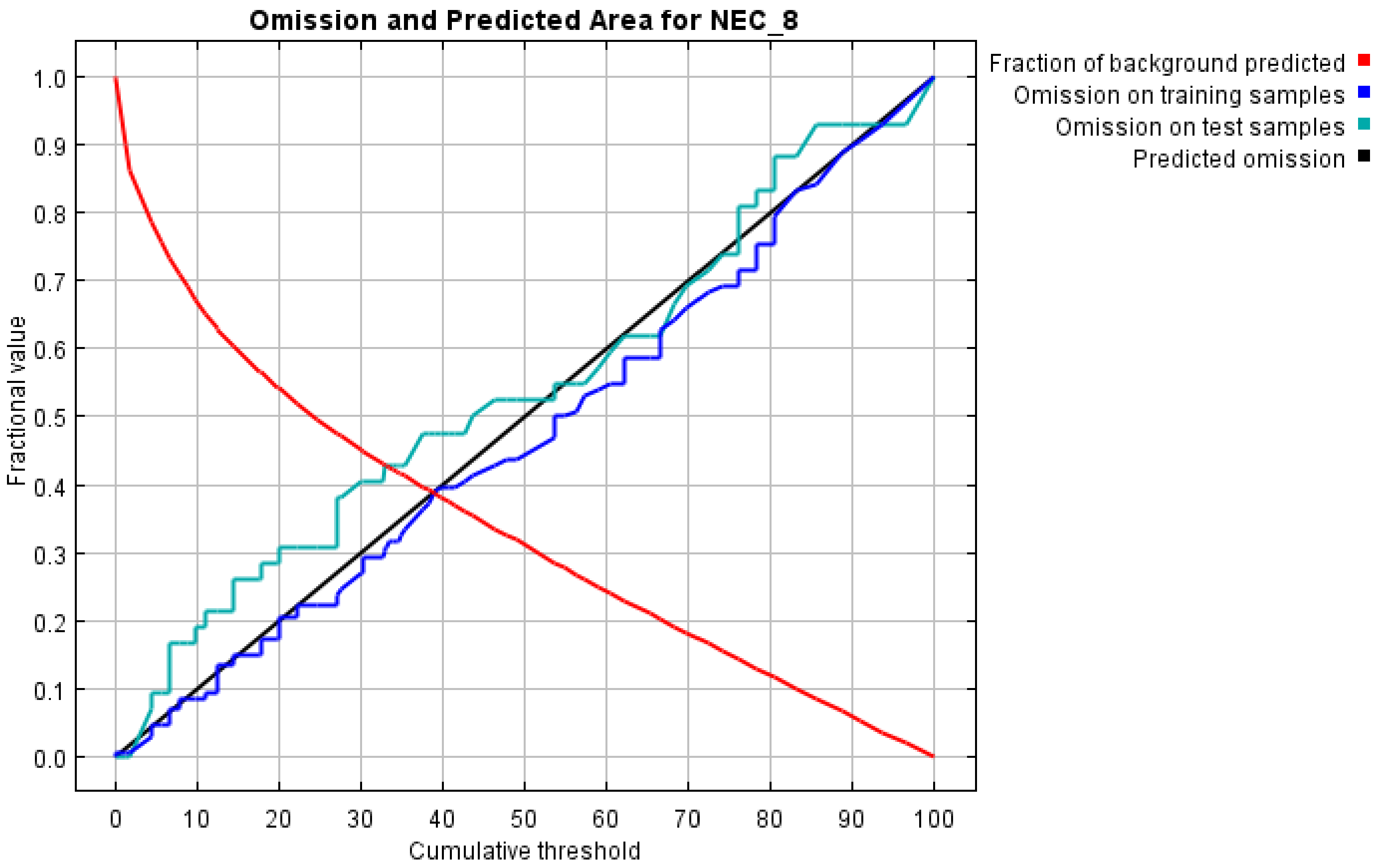

The next picture is the receiver operating characteristic (ROC) curve for the same data. Note that the specificity is defined using predicted area, rather than true commission (see the paper by Phillips, Anderson and Schapire cited on the help page for discussion of what this means). This implies that the maximum achievable AUC is less than 1. If test data is drawn from the Maxent distribution itself, then the maximum possible test AUC would be 0.660 rather than 1; in practice the test AUC may exceed this bound.

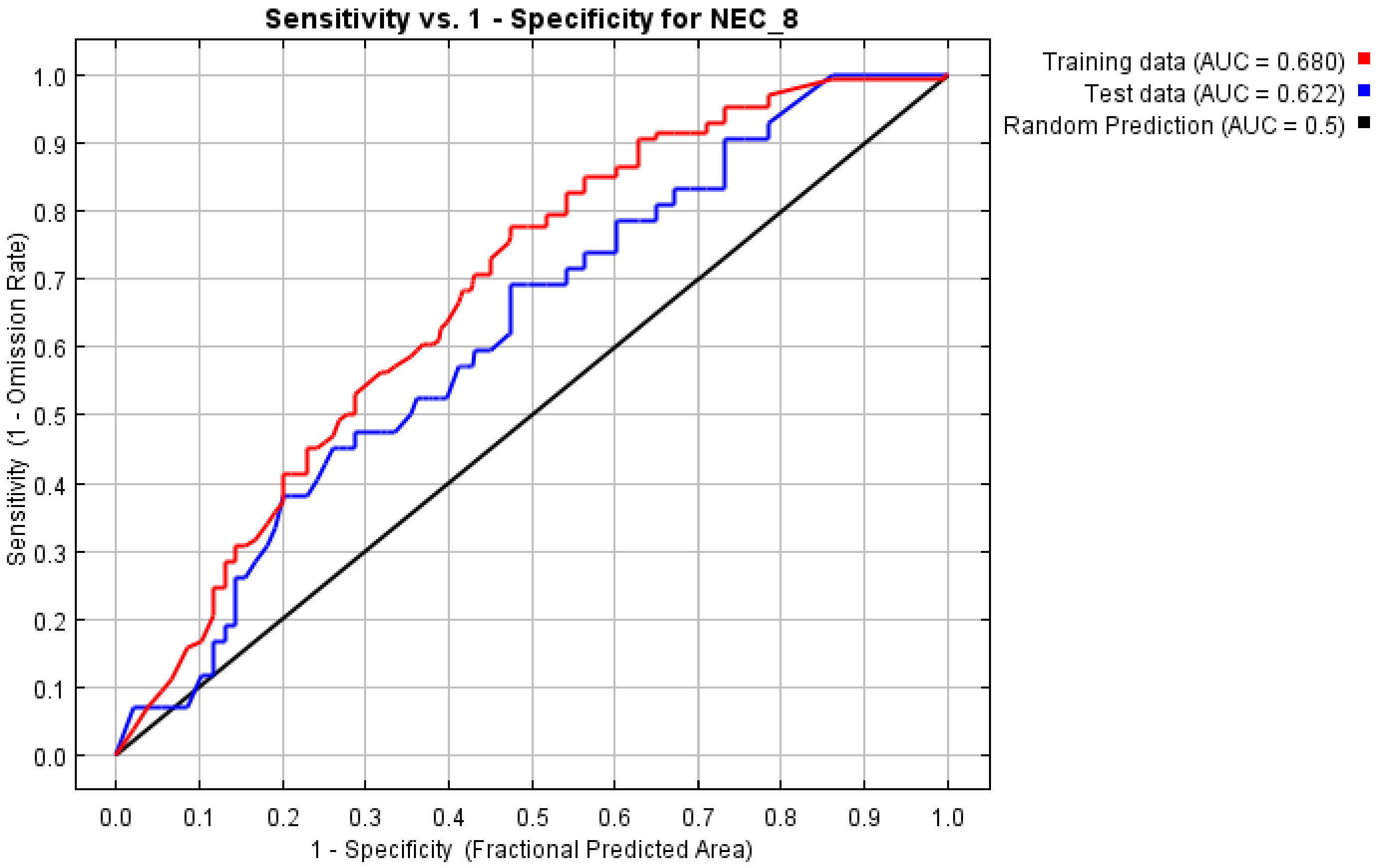

Some common thresholds and corresponding omission rates are as follows. If test data are available, binomial probabilities are calculated exactly if the number of test samples is at most 25, otherwise using a normal approximation to the binomial. These are 1-sided p-values for the null hypothesis that test points are predicted no better than by a random prediction with the same fractional predicted area. The "Balance" threshold minimizes 6 * training omission rate + .04 * cumulative threshold + 1.6 * fractional predicted area.

Cumulative threshold	Logistic threshold	Description	Fractional predicted area	Training omission rate	Test omission rate	P-value
1.000	0.090	Fixed cumulative value 1	0.899	0.008	0.000	1.483E-2
5.000	0.265	Fixed cumulative value 5	0.756	0.048	0.095	1.228E-2
10.000	0.346	Fixed cumulative value 10	0.649	0.087	0.190	1.487E-2
0.044	0.084	Minimum training presence	0.996	0.000	0.000	3.351E-1
12.474	0.395	10 percentile training presence	0.628	0.095	0.214	1.723E-2
38.982	0.542	Equal training sensitivity and specificity	0.387	0.389	0.476	3.457E-2
26.957	0.489	Maximum training sensitivity plus	0.476	0.222	0.310	2.659E-

		specificity				3
33.137	0.534	Equal test sensitivity and specificity	0.428	0.310	0.429	3.052E-2
26.957	0.489	Maximum test sensitivity plus specificity	0.476	0.222	0.310	2.659E-3
1.588	0.238	Balance training omission, predicted area and threshold value	0.860	0.008	0.000	4.521E-3
4.472	0.250	Equate entropy of thresholded and original distributions	0.784	0.032	0.071	1.154E-2

Click here to interactively explore this prediction using the Explain tool. If clicking from your browser does not succeed in starting the tool, try running the script in C:\Users\Bill\Desktop\Maxent Outputs NEC 7-8-15\NEC_8_explain.bat directly. This tool requires the environmental grids to be small enough that they all fit in memory.

Response curves

These curves show how each environmental variable affects the Maxent prediction. The curves show how the logistic prediction changes as each environmental variable is varied, keeping all other environmental variables at their average sample value. Click on a response curve to see a larger version. Note that the curves can be hard to interpret if you have strongly correlated variables, as the model may depend on the correlations in ways that are not evident in the curves. In other words, the curves show the marginal effect of changing exactly one variable, whereas the model may take advantage of sets of variables changing together.

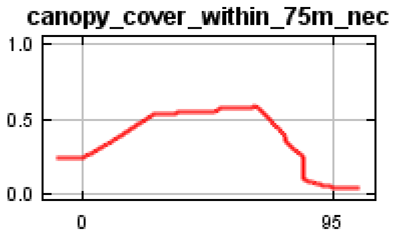

In contrast to the above marginal response curves, each of the following curves represents a different model, namely, a Maxent model created using only the corresponding variable. These plots reflect the dependence of predicted suitability both on the selected variable and on dependencies induced by correlations between the selected variable and other variables. They may be easier to interpret if there are strong correlations between variables.

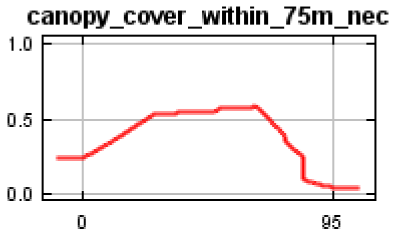

Analysis of variable contributions

The following table gives estimates of relative contributions of the environmental variables to the Maxent model. To determine the first estimate, in each iteration of the training algorithm, the increase in regularized gain is added to the contribution of the corresponding variable, or subtracted from it if the change to the absolute value of lambda is negative. For the second estimate, for each environmental variable in turn, the values of that variable on training presence and background data are randomly permuted. The model is reevaluated on the permuted data, and the resulting drop in training AUC is shown in the table, normalized to percentages. As with the variable jackknife, variable contributions should be interpreted with caution when the predictor variables are correlated.

Variable	Percent contribution	Permutation importance
canopy_cover_within_75m_nec	100	100

Raw data outputs and control parameters

The data used in the above analysis is contained in the next links. Please see the Help button for more information on these.
The model applied to the training environmental layers
The coefficients of the model
The omission and predicted area for varying cumulative and raw thresholds
The prediction strength at the training and (optionally) test presence sites
Results for all species modeled in the same Maxent run, with summary statistics and (optionally) jackknife results

Regularized training gain is 0.194, training AUC is 0.680, unregularized training gain is 0.234.
Unregularized test gain is 0.103.
Test AUC is 0.622, standard deviation is 0.039 (calculated as in DeLong, DeLong & Clarke-Pearson 1988, equation 2).
Algorithm converged after 240 iterations (0 seconds).

The follow settings were used during the run:
126 presence records used for training, 42 for testing.
10000 points used to determine the Maxent distribution (background points and presence points).
Environmental layers used (all continuous): canopy_cover_within_75m_nec
Regularization values: linear/quadratic/product: 0.050, categorical: 0.250, threshold: 1.000, hinge: 0.500
Feature types used: hinge linear threshold quadratic
responsecurves: true
pictures: false
outputdirectory: C:\Users\Bill\Desktop\Maxent Outputs NEC 7-8-15
samplesfile: C:\Users\Bill\Desktop\Maxent_samples\Compare_NEC_EC_150m_311_WGS_84_m.csv
environmentallayers: C:\Users\Bill\Desktop\Maxent Layers\maxent.cache
randomseed: true
randomtestpoints: 25
replicates: 10
replicatetype: subsample
Command line used:

Maxent model for NEC_9

This page contains some analysis of the Maxent model for NEC_9, created Wed Jul 08 18:56:34 EDT 2015 using Maxent version 3.3.3k. If you would like to do further analyses, the raw data used here is linked to at the end of this page.

Analysis of omission/commission

The following picture shows the omission rate and predicted area as a function of the cumulative threshold. The omission rate is is calculated both on the training presence records, and (if test data are used) on the test records. The omission rate should be close to the predicted omission, because of the definition of the cumulative threshold.

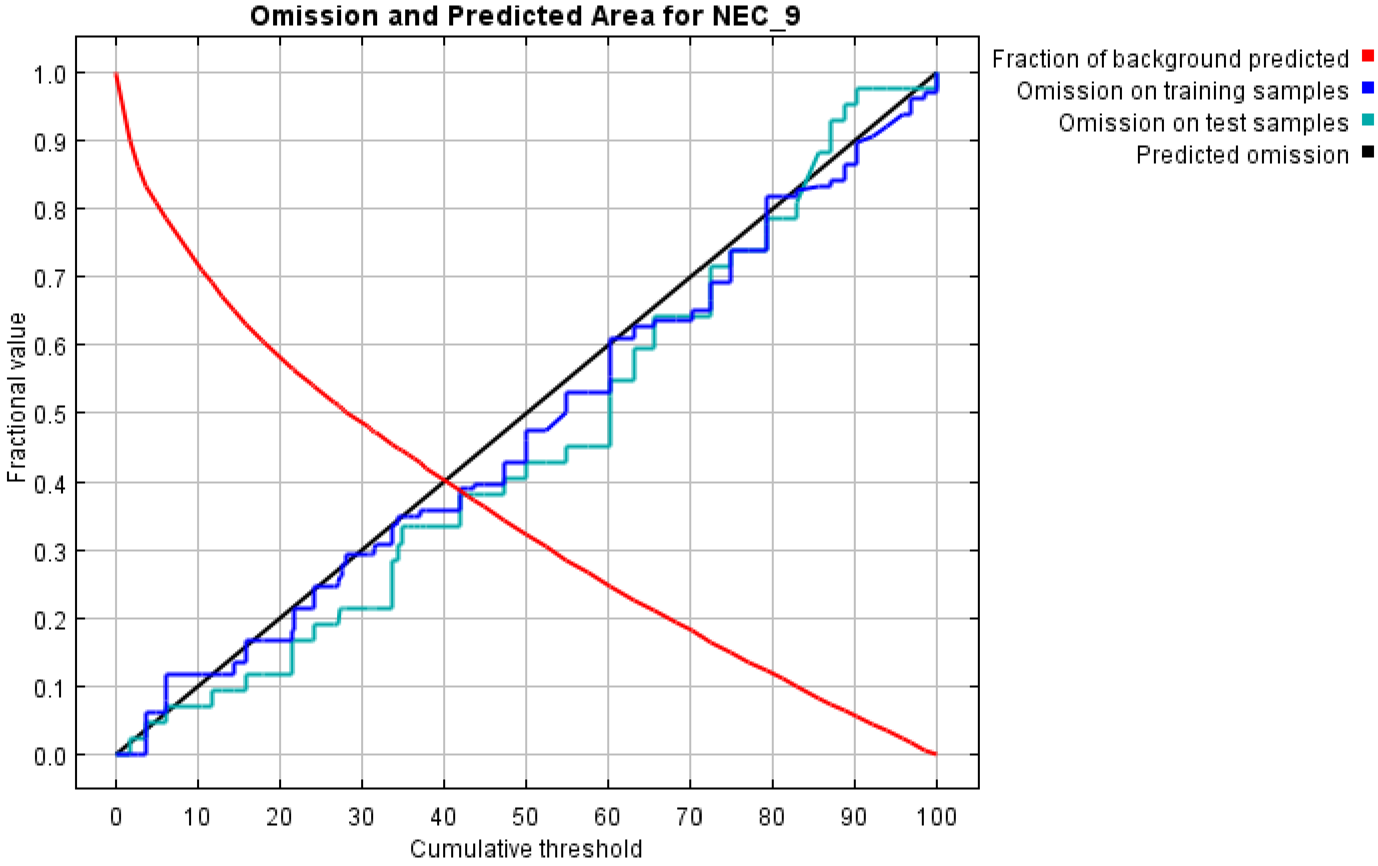

The next picture is the receiver operating characteristic (ROC) curve for the same data. Note that the specificity is defined using predicted area, rather than true commission (see the paper by Phillips, Anderson and Schapire cited on the help page for discussion of what this means). This implies that the maximum achievable AUC is less than 1. If test data is drawn from the Maxent distribution itself, then the maximum possible test AUC would be 0.643 rather than 1; in practice the test AUC may exceed this bound.

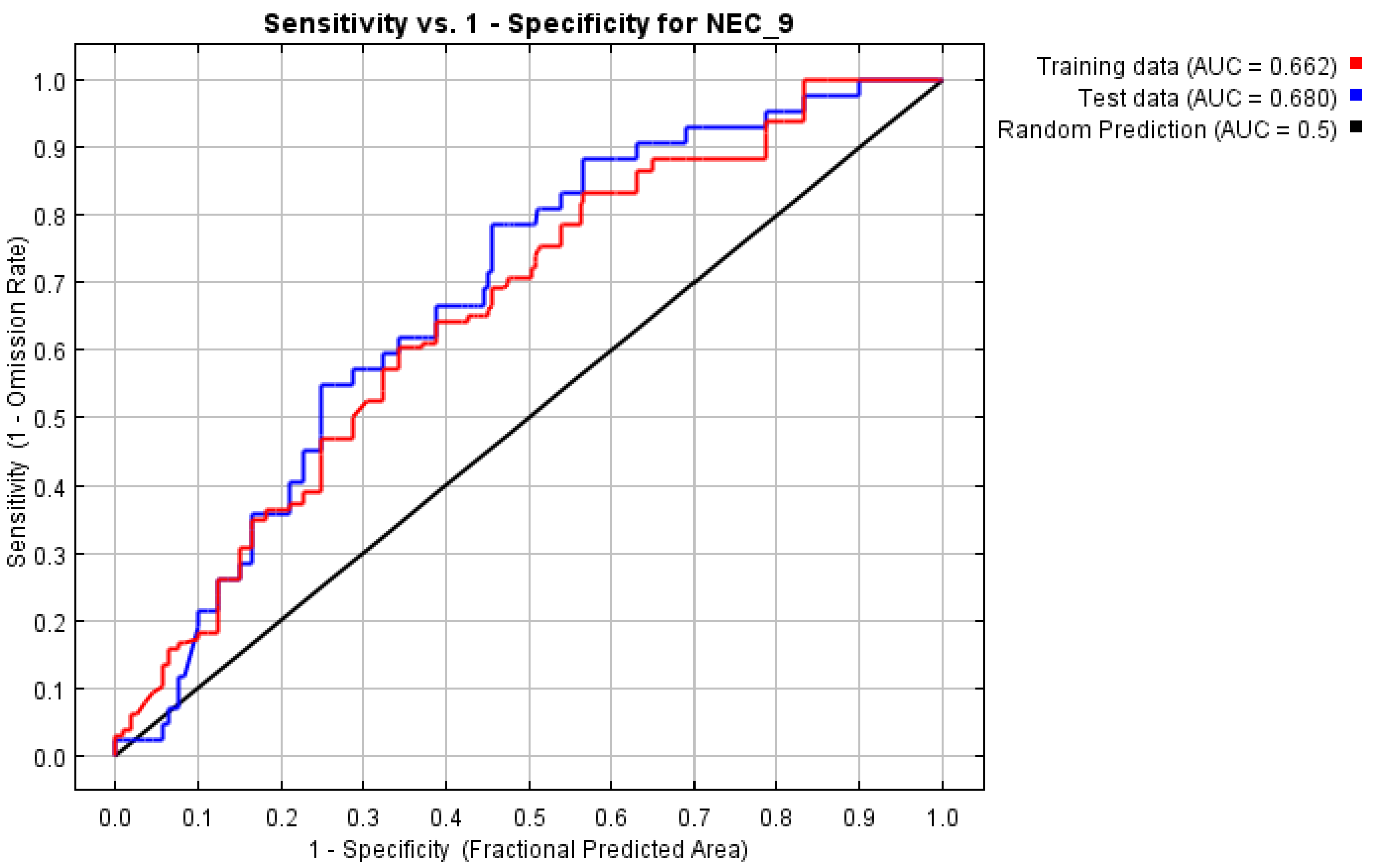

Some common thresholds and corresponding omission rates are as follows. If test data are available, binomial probabilities are calculated exactly if the number of test samples is at most 25, otherwise using a normal approximation to the binomial. These are 1-sided p-values for the null hypothesis that test points are predicted no better than by a random prediction with the same fractional predicted area. The "Balance" threshold minimizes 6 * training omission rate + .04 * cumulative threshold + 1.6 * fractional predicted area.

Cumulative threshold	Logistic threshold	Description	Fractional predicted area	Training omission rate	Test omission rate	P-value
1.000	0.124	Fixed cumulative value 1	0.899	0.000	0.000	1.483E-2
5.000	0.320	Fixed cumulative value 5	0.786	0.063	0.048	4.323E-3
10.000	0.340	Fixed cumulative value 10	0.710	0.119	0.071	9.093E-4
3.707	0.250	Minimum training presence	0.832	0.000	0.024	6.16E-3
6.083	0.332	10 percentile training presence	0.786	0.079	0.048	4.323E-3
41.915	0.515	Equal training sensitivity and specificity	0.387	0.389	0.381	1.029E-3
21.489	0.439	Maximum training sensitivity plus specificity	0.566	0.167	0.119	1.928E-5

42.846	0.515	Equal test sensitivity and specificity	0.380	0.389	0.381	6.999E-4
33.680	0.508	Maximum test sensitivity plus specificity	0.455	0.310	0.214	8.283E-6
3.707	0.250	Balance training omission, predicted area and threshold value	0.832	0.000	0.024	6.16E-3
2.603	0.250	Equate entropy of thresholded and original distributions	0.860	0.000	0.024	1.518E-2

Click here to interactively explore this prediction using the Explain tool. If clicking from your browser does not succeed in starting the tool, try running the script in C:\Users\Bill\Desktop\Maxent Outputs NEC 7-8-15\NEC_9_explain.bat directly. This tool requires the environmental grids to be small enough that they all fit in memory.

Response curves

These curves show how each environmental variable affects the Maxent prediction. The curves show how the logistic prediction changes as each environmental variable is varied, keeping all other environmental variables at their average sample value. Click on a response curve to see a larger version. Note that the curves can be hard to interpret if you have strongly correlated variables, as the model may depend on the correlations in ways that are not evident in the curves. In other words, the curves show the marginal effect of changing exactly one variable, whereas the model may take advantage of sets of variables changing together.

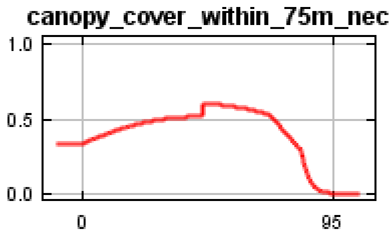

In contrast to the above marginal response curves, each of the following curves represents a different model, namely, a Maxent model created using only the corresponding variable. These plots reflect the dependence of predicted suitability both on the selected variable and on dependencies induced by correlations between the selected variable and other variables. They may be easier to interpret if there are strong correlations between variables.

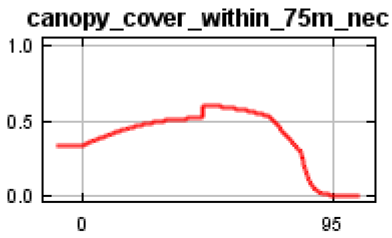

Analysis of variable contributions

The following table gives estimates of relative contributions of the environmental variables to the Maxent model. To determine the first estimate, in each iteration of the training algorithm, the increase in regularized gain is added to the contribution of the corresponding variable, or subtracted from it if the change to the absolute value of lambda is negative. For the second estimate, for each environmental variable in turn, the values of that variable on training presence and background data are randomly permuted. The model is reevaluated on the permuted data, and the resulting drop in training AUC is shown in the table, normalized to percentages. As with the variable jackknife, variable contributions should be interpreted with caution when the predictor variables are correlated.

Variable	Percent contribution	Permutation importance
canopy_cover_within_75m_nec	100	100

Raw data outputs and control parameters

The data used in the above analysis is contained in the next links. Please see the Help button for more information on these.
The model applied to the training environmental layers
The coefficients of the model
The omission and predicted area for varying cumulative and raw thresholds
The prediction strength at the training and (optionally) test presence sites
Results for all species modeled in the same Maxent run, with summary statistics and (optionally) jackknife results

Regularized training gain is 0.145, training AUC is 0.662, unregularized training gain is 0.183.
Unregularized test gain is 0.208.
Test AUC is 0.680, standard deviation is 0.035 (calculated as in DeLong, DeLong & Clarke-Pearson 1988, equation 2).
Algorithm converged after 400 iterations (1 seconds).

The follow settings were used during the run:
126 presence records used for training, 42 for testing.
10000 points used to determine the Maxent distribution (background points and presence points).
Environmental layers used (all continuous): canopy_cover_within_75m_nec
Regularization values: linear/quadratic/product: 0.050, categorical: 0.250, threshold: 1.000, hinge: 0.500
Feature types used: hinge linear threshold quadratic
responsecurves: true
pictures: false
outputdirectory: C:\Users\Bill\Desktop\Maxent Outputs NEC 7-8-15
samplesfile: C:\Users\Bill\Desktop\Maxent_samples\Compare_NEC_EC_150m_311_WGS_84_m.csv
environmentallayers: C:\Users\Bill\Desktop\Maxent Layers\maxent.cache
randomseed: true
randomtestpoints: 25
replicates: 10
replicatetype: subsample
Command line used:
