# Supplementary material for: An Analysis of Overstory Tree Canopy Cover in Sites Occupied by Native and Introduced Cottontails in the Northeastern United States with Recommendations for Habitat Management for New England Cottontail
Source: PLoS One. 2015 Aug 12;10(8):e0135067. doi: 10.1371/journal.pone.0135067 (PMC4534376; doi:10.1371/journal.pone.0135067)
Supplement: S2 Model Outputs — (PDF) [file pone.0135067.s003.pdf]

Replicated maxent model for EC

This page summarizes the results of 10 split-sample models for EC, created Wed Jul 08 11:48:54 EDT 2015 using Maxent version 3.3.3k. The individual models are here: [0] [1] [2] [3] [4] [5] [6] [7] [8] [9]

Analysis of omission/commission

The following picture shows the test omission rate and predicted area as a function of the cumulative threshold, averaged over the replicate runs. The omission rate should be close to the predicted omission, because of the definition of the cumulative threshold.

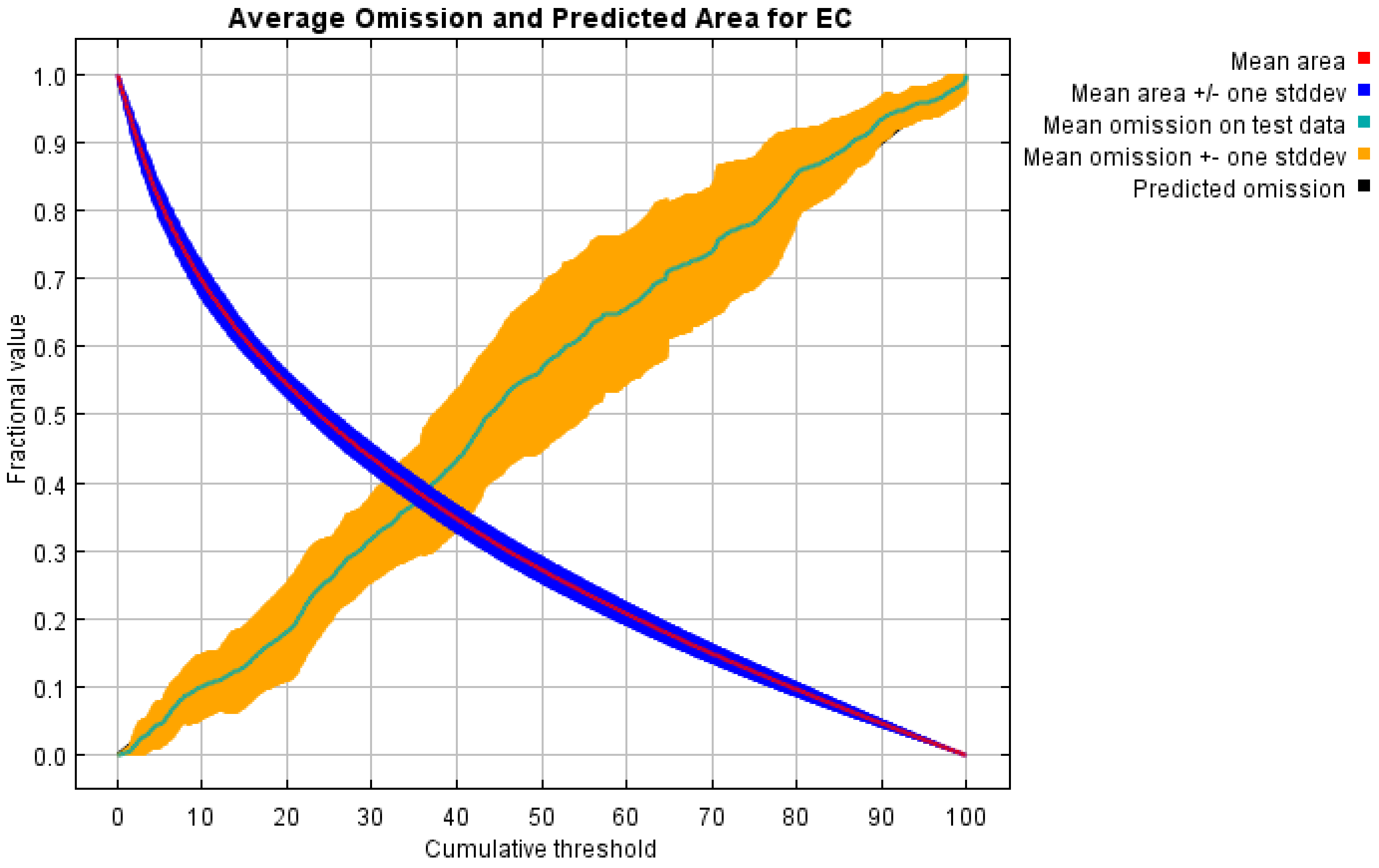

The next picture is the receiver operating characteristic (ROC) curve for the same data, again averaged over the replicate runs. Note that the specificity is defined using predicted area, rather than true commission (see the paper by Phillips, Anderson and Schapire cited on the help page for discussion of what this means). The average test AUC for the replicate runs is 0.651, and the standard deviation is 0.039.

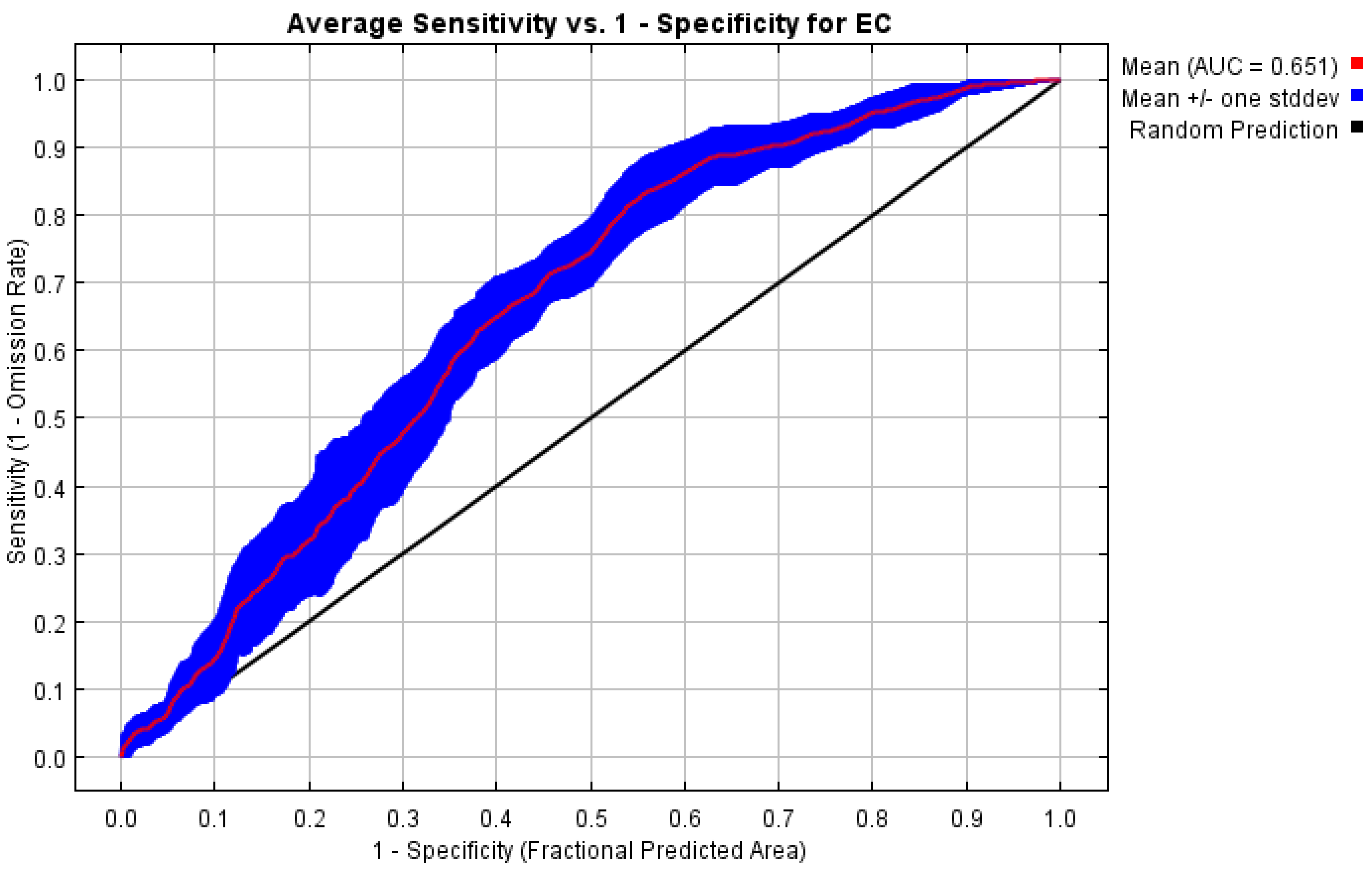

Response curves

These curves show how each environmental variable affects the Maxent prediction. The curves show how the logistic prediction changes as each environmental variable is varied, keeping all other environmental variables at their average sample value. Click on a response curve to see a larger version. Note that the curves can be hard to interpret if you have strongly correlated variables, as the model may depend on the correlations in ways that are not evident in the curves. In other words, the curves show the marginal effect of changing exactly one variable, whereas the model may take advantage of sets of variables changing together. The curves show the mean response of the 10 replicate Maxent runs (red) and and the mean +/- one standard deviation (blue, two shades for categorical variables).

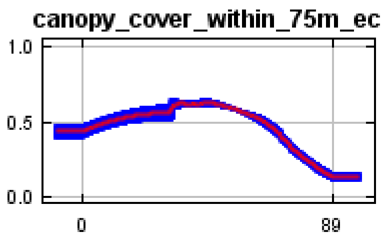

In contrast to the above marginal response curves, each of the following curves represents a different model, namely, a Maxent model created using only the corresponding variable. These plots reflect the dependence of predicted suitability both on the selected variable and on dependencies induced by correlations between the selected variable and other variables. They may be easier to interpret if there are strong correlations between variables.

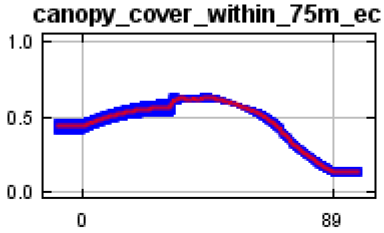

Analysis of variable contributions

The following table gives estimates of relative contributions of the environmental variables to the Maxent model. To determine the first estimate, in each iteration of the training algorithm, the increase in regularized gain is added to the contribution of the corresponding variable, or subtracted from it if the change to the absolute value of lambda is negative. For the second estimate, for each environmental variable in turn, the values of that variable on training presence and background data are randomly permuted. The model is reevaluated on the permuted data, and the resulting drop in training AUC is shown in the table, normalized to percentages. As with the variable jackknife, variable contributions should be interpreted with caution when the predictor variables are correlated. Values shown are averages over replicate runs.

Variable	Percent contribution	Permutation importance
canopy_cover_within_75m_ec	100	100

Command line to repeat this species model: java density.MaxEnt nowarnings noprefixes -E "" -E EC responsecurves nopictures "outputdirectory=C:\Users\Bill\Desktop\Maxent Outputs EC 7-8-15" samplesfile=C:\Users\Bill\Desktop\Maxent_samples\Compare_NEC_EC_150m_311_WGS_84_m.csv "environmentallayers=C:\Users\Bill\Desktop\Maxent Layers\maxent.cache" randomseed noaskoverwrite randomtestpoints=25 replicates=10 replicatetype=subsample -N canopy_cover_within_75m_nec -N cc10foc75_02_buff3km_nec_01_wgs84_01 -N cc10foc75_04_buff3km_nec_20_wgs84_20 -N cc10foc75_04_wgs84_02 -N cc10mfoc75_04_buff3km_ec_20_wgs84_20_01 -N cc_10foc75_d_buff3km_nec_01_wgs84_10 -N cc_10mfoc75_d_buff3km_ec_01_wgs84_10 -N vpd_b_wgs_buff10_ec_01 -N vpd_b_wgs_buff50_ec_01 -N vpd_b_wgs_ec_01 -N vpd_b_wgs_nec_01

Maxent model for EC_0

This page contains some analysis of the Maxent model for EC_0, created Wed Jul 08 10:46:35 EDT 2015 using Maxent version 3.3.3k. If you would like to do further analyses, the raw data used here is linked to at the end of this page.

Analysis of omission/commission

The following picture shows the omission rate and predicted area as a function of the cumulative threshold. The omission rate is is calculated both on the training presence records, and (if test data are used) on the test records. The omission rate should be close to the predicted omission, because of the definition of the cumulative threshold.

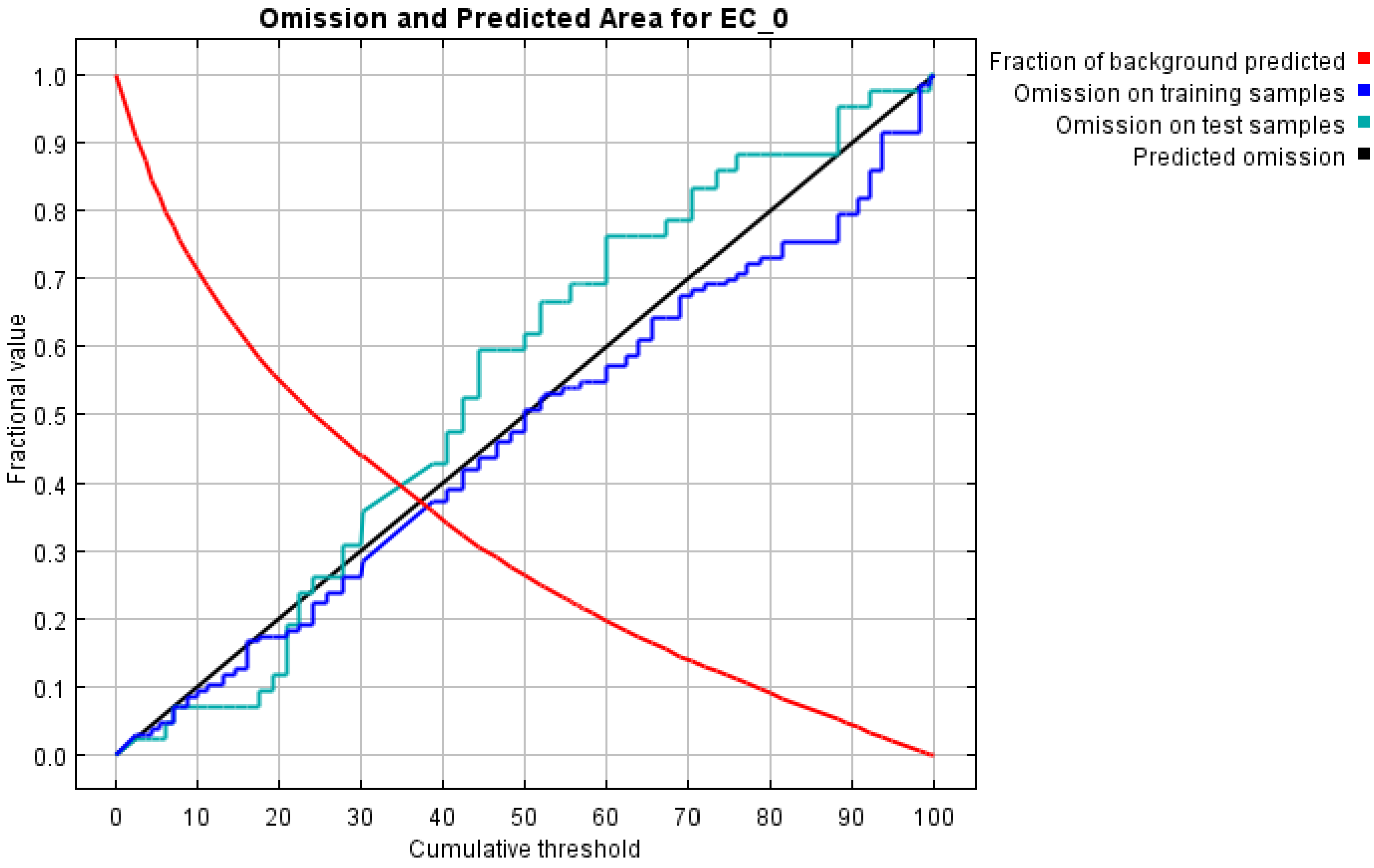

The next picture is the receiver operating characteristic (ROC) curve for the same data. Note that the specificity is defined using predicted area, rather than true commission (see the paper by Phillips, Anderson and Schapire cited on the help page for discussion of what this means). This implies that the maximum achievable AUC is less than 1. If test data is drawn from the Maxent distribution itself, then the maximum possible test AUC would be 0.674 rather than 1; in practice the test AUC may exceed this bound.

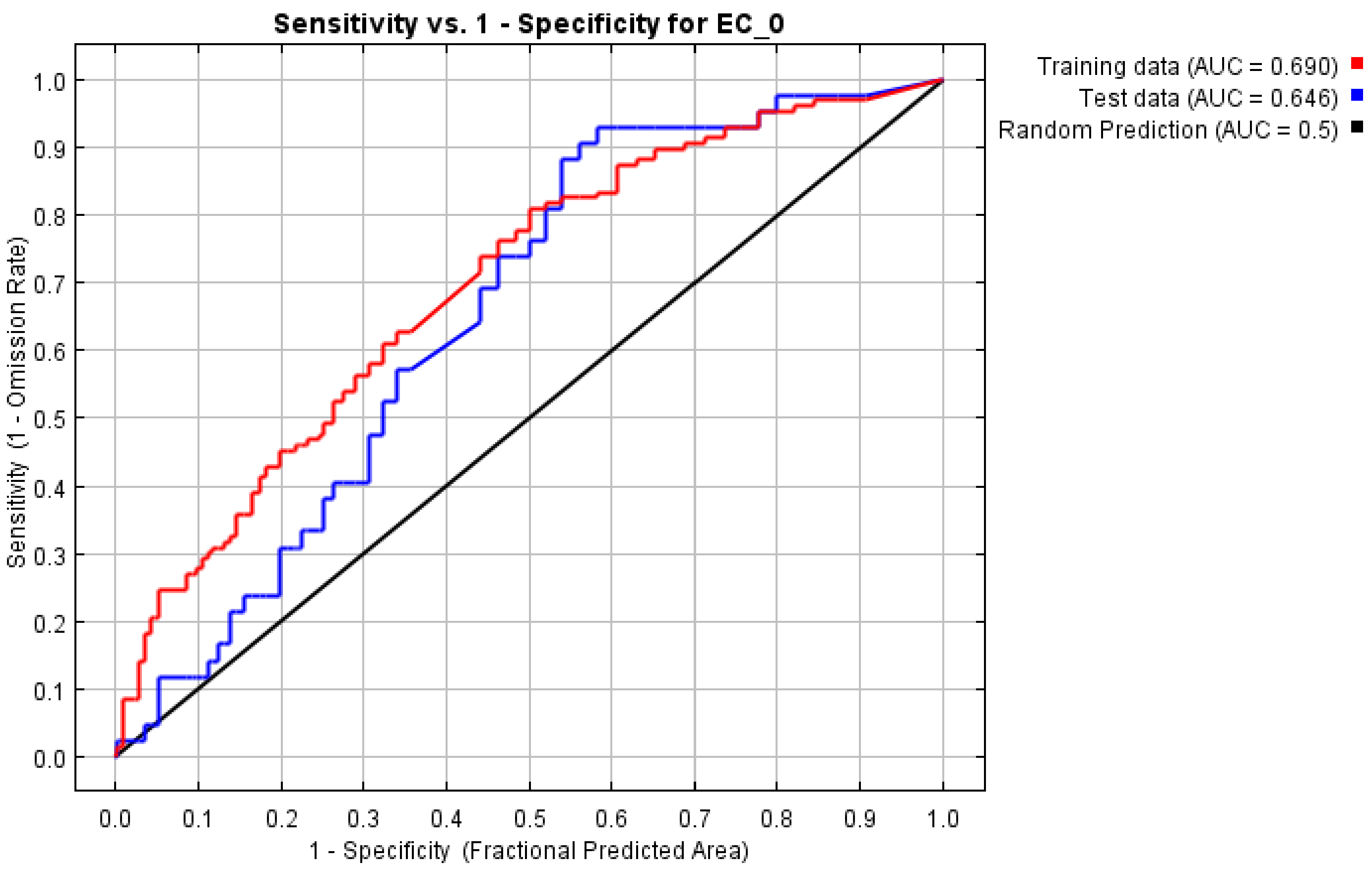

Some common thresholds and corresponding omission rates are as follows. If test data are available, binomial probabilities are calculated exactly if the number of test samples is at most 25, otherwise using a normal approximation to the binomial. These are 1-sided p-values for the null hypothesis that test points are predicted no better than by a random prediction with the same fractional predicted area. The "Balance" threshold minimizes 6 * training omission rate + .04 * cumulative threshold + 1.6 * fractional predicted area.

Cumulative threshold	Logistic threshold	Description	Fractional predicted area	Training omission rate	Test omission rate	P-value
1.000	0.197	Fixed cumulative value 1	0.908	0.032	0.024	6.295E-2
5.000	0.222	Fixed cumulative value 5	0.820	0.040	0.024	4.238E-3
10.000	0.304	Fixed cumulative value 10	0.688	0.095	0.071	3.78E-4
0.062	0.184	Minimum training presence	0.998	0.000	0.000	3.753E-1
11.251	0.304	10 percentile training presence	0.688	0.095	0.071	3.78E-4
38.776	0.471	Equal training sensitivity and specificity	0.357	0.373	0.429	1.845E-3
24.158	0.418	Maximum training sensitivity plus specificity	0.502	0.190	0.238	3.775E-4
		Equal test sensitivity and				1.845E-

38.776	0.471	specificity	0.357	0.373	0.429	3
17.653	0.361	Maximum test sensitivity plus specificity	0.584	0.167	0.071	2.942E-6
0.062	0.184	Balance training omission, predicted area and threshold value	0.998	0.000	0.000	3.753E-1
5.292	0.222	Equate entropy of thresholded and original distributions	0.820	0.040	0.024	4.238E-3

Click here to interactively explore this prediction using the Explain tool. If clicking from your browser does not succeed in starting the tool, try running the script in C:\Users\Bill\Desktop\Maxent Outputs EC 7-8-15\EC_0_explain.bat directly. This tool requires the environmental grids to be small enough that they all fit in memory.

Response curves

These curves show how each environmental variable affects the Maxent prediction. The curves show how the logistic prediction changes as each environmental variable is varied, keeping all other environmental variables at their average sample value. Click on a response curve to see a larger version. Note that the curves can be hard to interpret if you have strongly correlated variables, as the model may depend on the correlations in ways that are not evident in the curves. In other words, the curves show the marginal effect of changing exactly one variable, whereas the model may take advantage of sets of variables changing together.

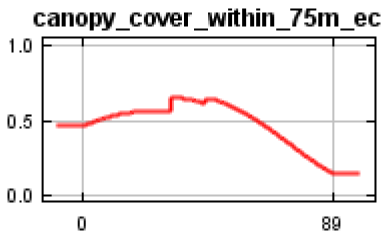

-

In contrast to the above marginal response curves, each of the following curves represents a different model, namely, a Maxent model created using only the corresponding variable. These plots reflect the dependence of predicted suitability both on the selected variable and on dependencies induced by correlations between the selected variable and other variables. They may be easier to interpret if there are strong correlations between variables.

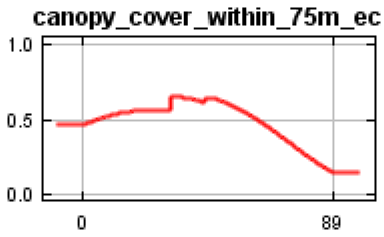

-

Analysis of variable contributions

The following table gives estimates of relative contributions of the environmental variables to the Maxent model. To determine the first estimate, in each iteration of the training algorithm, the increase in regularized gain is added to the contribution of the corresponding variable, or subtracted from it if the change to the absolute value of lambda is negative. For the second estimate, for each environmental variable in turn, the values of that variable on training presence and background data are randomly permuted. The model is reevaluated on the permuted data, and the resulting drop in training AUC is shown in the table, normalized to percentages. As with the variable jackknife, variable contributions should be interpreted with caution when the predictor variables are correlated.

Variable	Percent contribution	Permutation importance
canopy_cover_within_75m_ec	100	100

Raw data outputs and control parameters

The data used in the above analysis is contained in the next links. Please see the Help button for more information on these.
The model applied to the training environmental layers
The coefficients of the model
The omission and predicted area for varying cumulative and raw thresholds
The prediction strength at the training and (optionally) test presence sites
Results for all species modeled in the same Maxent run, with summary statistics and (optionally) jackknife results

Regularized training gain is 0.191, training AUC is 0.690, unregularized training gain is 0.219.
Unregularized test gain is 0.138.
Test AUC is 0.646, standard deviation is 0.033 (calculated as in DeLong, DeLong & Clarke-Pearson 1988, equation 2).
Algorithm converged after 420 iterations (1 seconds).

The follow settings were used during the run:
126 presence records used for training, 42 for testing.
10000 points used to determine the Maxent distribution (background points and presence points).
Environmental layers used (all continuous): canopy_cover_within_75m_ec
Regularization values: linear/quadratic/product: 0.050, categorical: 0.250, threshold: 1.000, hinge: 0.500
Feature types used: hinge linear threshold quadratic
responsecurves: true
pictures: false
outputdirectory: C:\Users\Bill\Desktop\Maxent Outputs EC 7-8-15
samplesfile: C:\Users\Bill\Desktop\Maxent_samples\Compare_NEC_EC_150m_311_WGS_84_m.csv
environmentallayers: C:\Users\Bill\Desktop\Maxent Layers\maxent.cache
randomseed: true
askoverwrite: false
randomtestpoints: 25
replicates: 10
replicatetype: subsample
Command line used:

Maxent model for EC_1

This page contains some analysis of the Maxent model for EC_1, created Wed Jul 08 10:52:49 EDT 2015 using Maxent version 3.3.3k. If you would like to do further analyses, the raw data used here is linked to at the end of this page.

Analysis of omission/commission

The following picture shows the omission rate and predicted area as a function of the cumulative threshold. The omission rate is is calculated both on the training presence records, and (if test data are used) on the test records. The omission rate should be close to the predicted omission, because of the definition of the cumulative threshold.

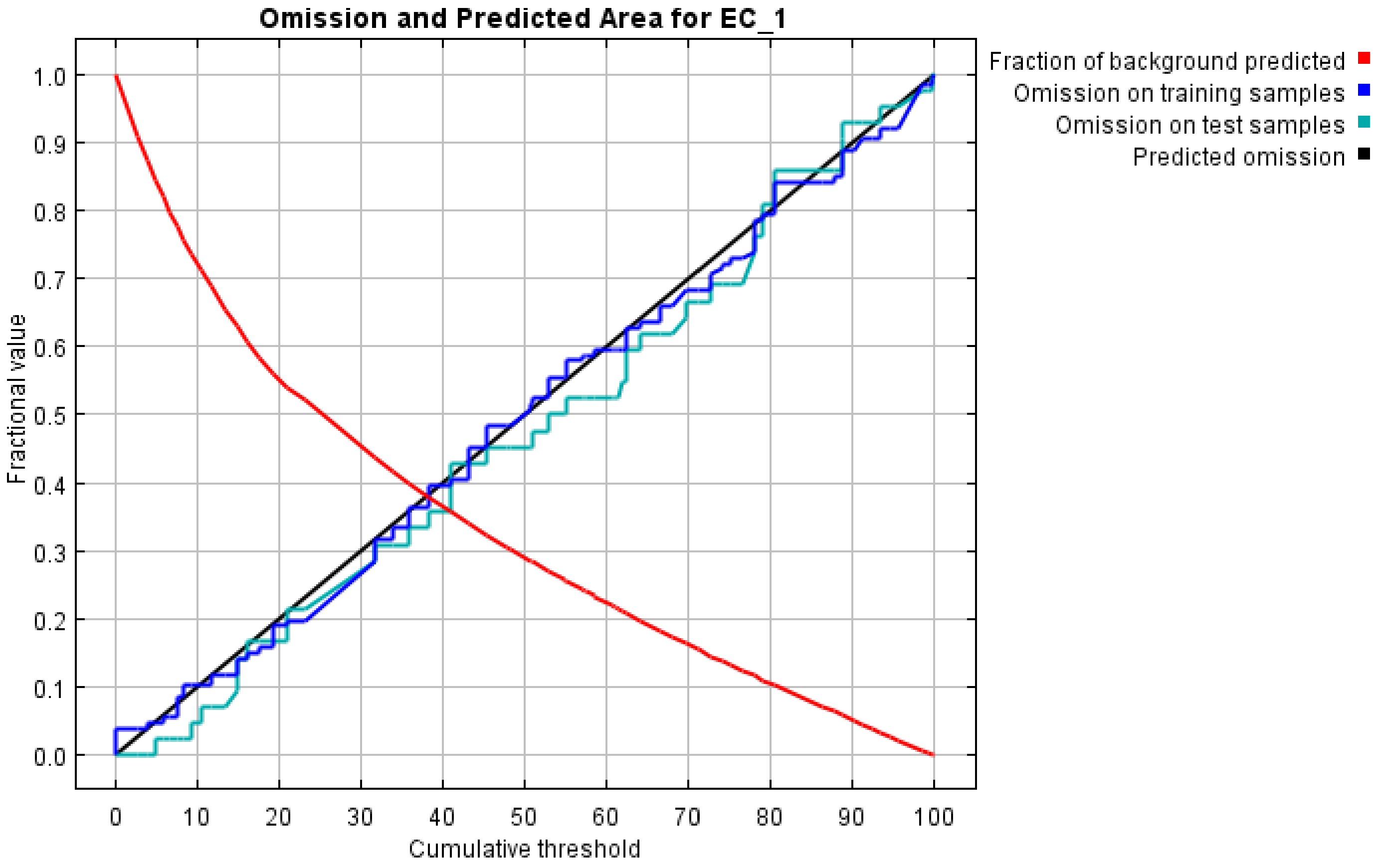

The next picture is the receiver operating characteristic (ROC) curve for the same data. Note that the specificity is defined using predicted area, rather than true commission (see the paper by Phillips, Anderson and Schapire cited on the help page for discussion of what this means). This implies that the maximum achievable AUC is less than 1. If test data is drawn from the Maxent distribution itself, then the maximum possible test AUC would be 0.660 rather than 1; in practice the test AUC may exceed this bound.

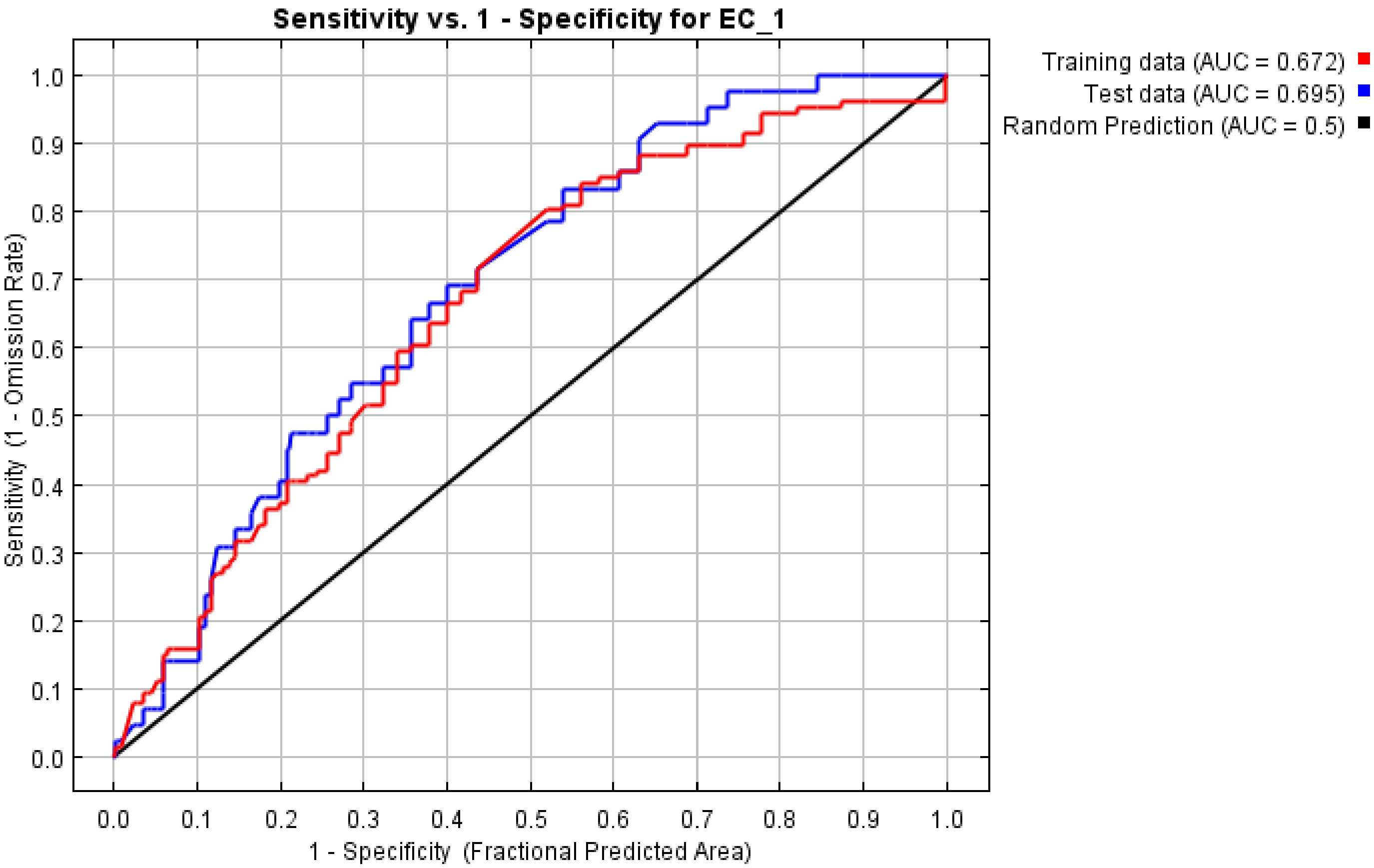

Some common thresholds and corresponding omission rates are as follows. If test data are available, binomial probabilities are calculated exactly if the number of test samples is at most 25, otherwise using a normal approximation to the binomial. These are 1-sided p-values for the null hypothesis that test points are predicted no better than by a random prediction with the same fractional predicted area. The "Balance" threshold minimizes 6 * training omission rate + .04 * cumulative threshold + 1.6 * fractional predicted area.

Cumulative threshold	Logistic threshold	Description	Fractional predicted area	Training omission rate	Test omission rate	P-value
1.000	0.215	Fixed cumulative value 1	0.908	0.040	0.000	1.95E-2
5.000	0.246	Fixed cumulative value 5	0.820	0.048	0.024	4.238E-3
10.000	0.300	Fixed cumulative value 10	0.713	0.103	0.048	2.988E-4
0.070	0.205	Minimum training presence	0.998	0.000	0.000	3.753E-1
8.369	0.278	10 percentile training presence	0.756	0.087	0.024	4.477E-4
38.297	0.511	Equal training sensitivity and specificity	0.379	0.365	0.333	6.235E-5
19.157	0.453	Maximum training sensitivity plus specificity	0.560	0.159	0.167	1.837E-4

41.078	0.522	Equal test sensitivity and specificity	0.357	0.397	0.357	5.446E-5
21.090	0.465	Maximum test sensitivity plus specificity	0.541	0.190	0.167	7.073E-5
0.070	0.205	Balance training omission, predicted area and threshold value	0.998	0.000	0.000	3.753E-1
4.900	0.235	Equate entropy of thresholded and original distributions	0.844	0.048	0.000	2.675E-3

Click here to interactively explore this prediction using the Explain tool. If clicking from your browser does not succeed in starting the tool, try running the script in C:\Users\Bill\Desktop\Maxent Outputs EC 7-8-15\EC_1_explain.bat directly. This tool requires the environmental grids to be small enough that they all fit in memory.

Response curves

These curves show how each environmental variable affects the Maxent prediction. The curves show how the logistic prediction changes as each environmental variable is varied, keeping all other environmental variables at their average sample value. Click on a response curve to see a larger version. Note that the curves can be hard to interpret if you have strongly correlated variables, as the model may depend on the correlations in ways that are not evident in the curves. In other words, the curves show the marginal effect of changing exactly one variable, whereas the model may take advantage of sets of variables changing together.

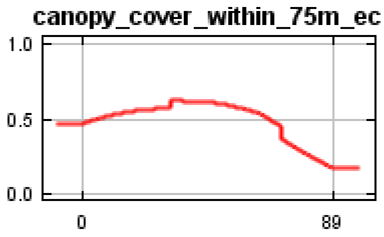

In contrast to the above marginal response curves, each of the following curves represents a different model, namely, a Maxent model created using only the corresponding variable. These plots reflect the dependence of predicted suitability both on the selected variable and on dependencies induced by correlations between the selected variable and other variables. They may be easier to interpret if there are strong correlations between variables.

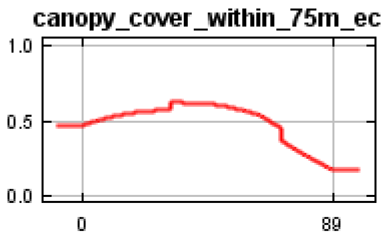

Analysis of variable contributions

The following table gives estimates of relative contributions of the environmental variables to the Maxent model. To determine the first estimate, in each iteration of the training algorithm, the increase in regularized gain is added to the contribution of the corresponding variable, or subtracted from it if the change to the absolute value of lambda is negative. For the second estimate, for each environmental variable in turn, the values of that variable on training presence and background data are randomly permuted. The model is reevaluated on the permuted data, and the resulting drop in training AUC is shown in the table, normalized to percentages. As with the variable jackknife, variable contributions should be interpreted with caution when the predictor variables are correlated.

Variable	Percent contribution	Permutation importance
canopy_cover_within_75m_ec	100	100

Raw data outputs and control parameters

The data used in the above analysis is contained in the next links. Please see the Help button for more information on these.
The model applied to the training environmental layers
The coefficients of the model
The omission and predicted area for varying cumulative and raw thresholds
The prediction strength at the training and (optionally) test presence sites
Results for all species modeled in the same Maxent run, with summary statistics and (optionally) jackknife results

Regularized training gain is 0.165, training AUC is 0.672, unregularized training gain is 0.194.
Unregularized test gain is 0.236.
Test AUC is 0.695, standard deviation is 0.034 (calculated as in DeLong, DeLong & Clarke-Pearson 1988, equation 2).
Algorithm converged after 320 iterations (1 seconds).

The follow settings were used during the run:
126 presence records used for training, 42 for testing.
10000 points used to determine the Maxent distribution (background points and presence points).
Environmental layers used (all continuous): canopy_cover_within_75m_ec
Regularization values: linear/quadratic/product: 0.050, categorical: 0.250, threshold: 1.000, hinge: 0.500
Feature types used: hinge linear threshold quadratic
responsecurves: true
pictures: false
outputdirectory: C:\Users\Bill\Desktop\Maxent Outputs EC 7-8-15
samplesfile: C:\Users\Bill\Desktop\Maxent_samples\Compare_NEC_EC_150m_311_WGS_84_m.csv
environmentallayers: C:\Users\Bill\Desktop\Maxent Layers\maxent.cache
randomseed: true
askoverwrite: false
randomtestpoints: 25
replicates: 10
replicatetype: subsample
Command line used:

Maxent model for EC_2

This page contains some analysis of the Maxent model for EC_2, created Wed Jul 08 10:59:03 EDT 2015 using Maxent version 3.3.3k. If you would like to do further analyses, the raw data used here is linked to at the end of this page.

Analysis of omission/commission

The following picture shows the omission rate and predicted area as a function of the cumulative threshold. The omission rate is is calculated both on the training presence records, and (if test data are used) on the test records. The omission rate should be close to the predicted omission, because of the definition of the cumulative threshold.

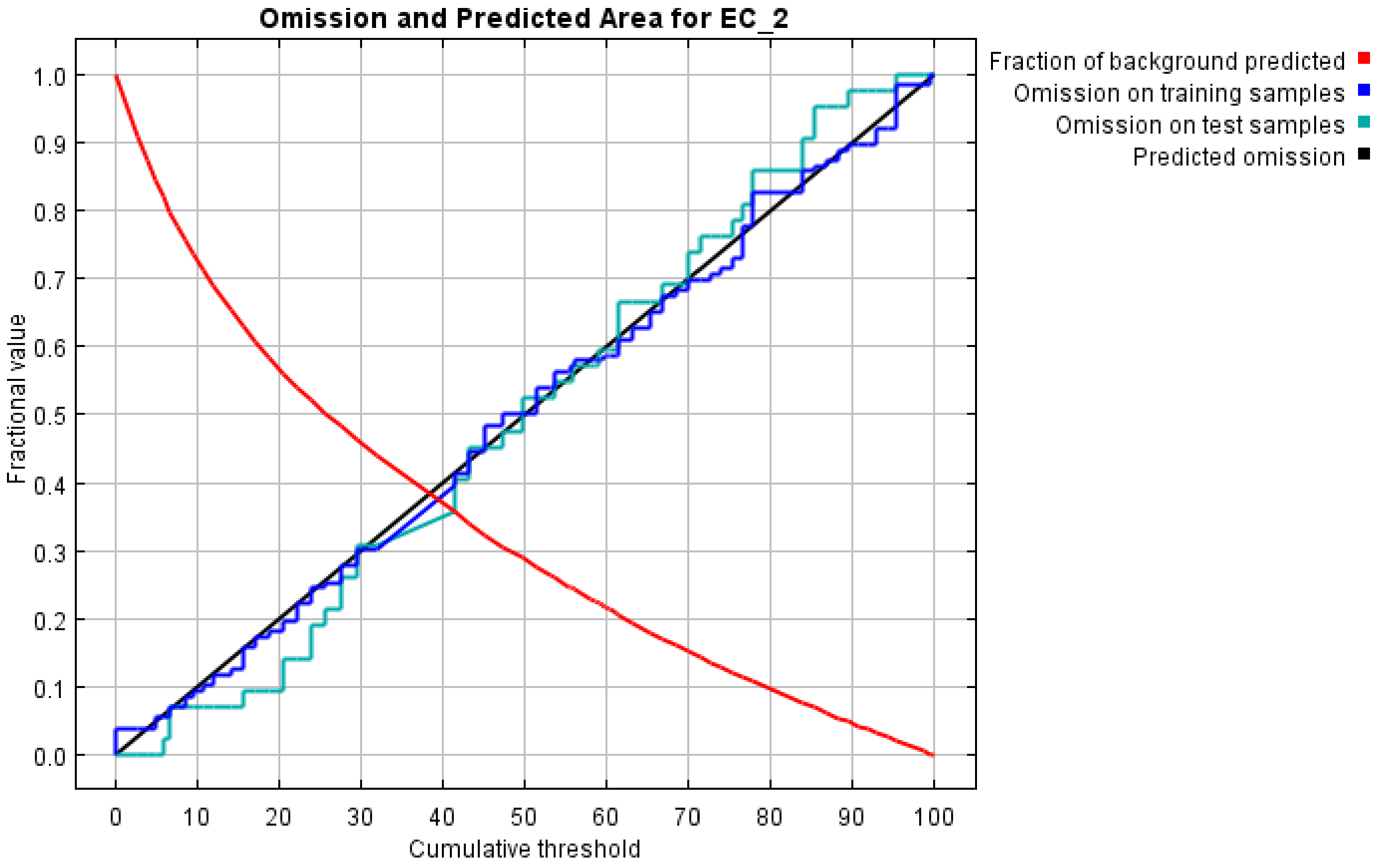

The next picture is the receiver operating characteristic (ROC) curve for the same data. Note that the specificity is defined using predicted area, rather than true commission (see the paper by Phillips, Anderson and Schapire cited on the help page for discussion of what this means). This implies that the maximum achievable AUC is less than 1. If test data is drawn from the Maxent distribution itself, then the maximum possible test AUC would be 0.660 rather than 1; in practice the test AUC may exceed this bound.

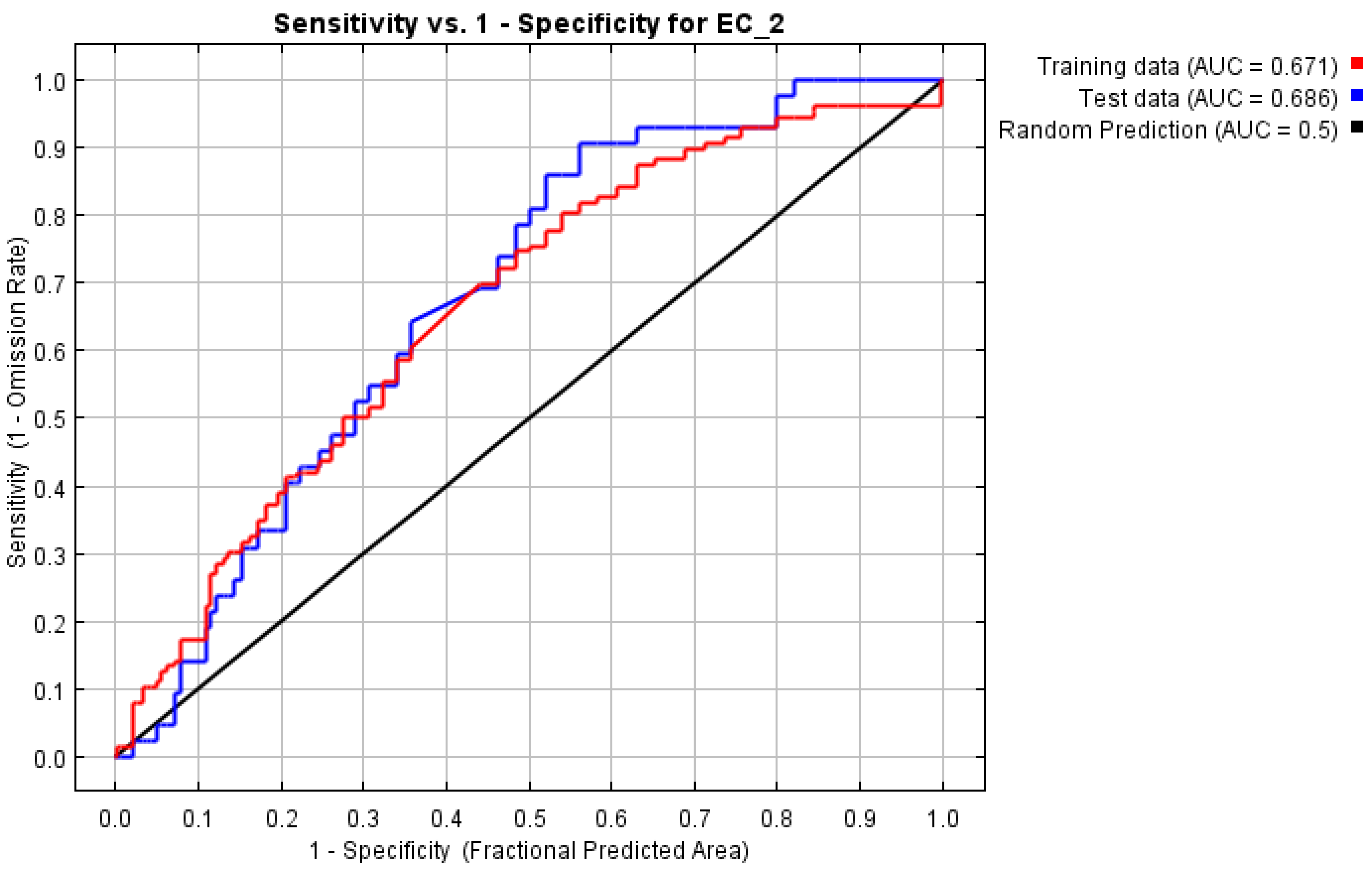

Some common thresholds and corresponding omission rates are as follows. If test data are available, binomial probabilities are calculated exactly if the number of test samples is at most 25, otherwise using a normal approximation to the binomial. These are 1-sided p-values for the null hypothesis that test points are predicted no better than by a random prediction with the same fractional predicted area. The "Balance" threshold minimizes 6 * training omission rate + .04 * cumulative threshold + 1.6 * fractional predicted area.

Cumulative threshold	Logistic threshold	Description	Fractional predicted area	Training omission rate	Test omission rate	P-value
1.000	0.214	Fixed cumulative value 1	0.908	0.040	0.000	1.95E-2
5.000	0.252	Fixed cumulative value 5	0.820	0.056	0.000	1.205E-3
10.000	0.321	Fixed cumulative value 10	0.713	0.095	0.071	9.95E-4
0.068	0.202	Minimum training presence	0.998	0.000	0.000	3.753E-1
10.665	0.321	10 percentile training presence	0.713	0.095	0.071	9.95E-4
41.357	0.494	Equal training sensitivity and specificity	0.357	0.397	0.357	5.446E-5
27.448	0.466	Maximum training sensitivity plus specificity	0.483	0.254	0.214	4.392E-5
41.357	0.494	Equal test sensitivity and specificity	0.357	0.397	0.357	5.446E-5

20.585	0.409	Maximum test sensitivity plus specificity	0.560	0.183	0.095	3.476E-6
0.068	0.202	Balance training omission, predicted area and threshold value	0.998	0.000	0.000	3.753E-1
4.853	0.227	Equate entropy of thresholded and original distributions	0.844	0.040	0.000	2.675E-3

Click here to interactively explore this prediction using the Explain tool. If clicking from your browser does not succeed in starting the tool, try running the script in C:\Users\Bill\Desktop\Maxent Outputs EC 7-8-15\EC_2_explain.bat directly. This tool requires the environmental grids to be small enough that they all fit in memory.

Response curves

These curves show how each environmental variable affects the Maxent prediction. The curves show how the logistic prediction changes as each environmental variable is varied, keeping all other environmental variables at their average sample value. Click on a response curve to see a larger version. Note that the curves can be hard to interpret if you have strongly correlated variables, as the model may depend on the correlations in ways that are not evident in the curves. In other words, the curves show the marginal effect of changing exactly one variable, whereas the model may take advantage of sets of variables changing together.

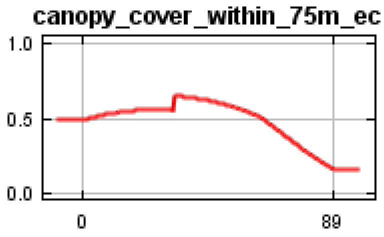

-

In contrast to the above marginal response curves, each of the following curves represents a different model, namely, a Maxent model created using only the corresponding variable. These plots reflect the dependence of predicted suitability both on the selected variable and on dependencies induced by correlations between the selected variable and other variables. They may be easier to interpret if there are strong correlations between variables.

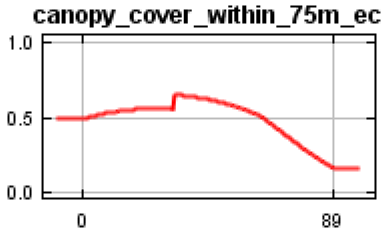

-

Analysis of variable contributions

The following table gives estimates of relative contributions of the environmental variables to the Maxent model. To determine the first estimate, in each iteration of the training algorithm, the increase in regularized gain is added to the contribution of the corresponding variable, or subtracted from it if the change to the absolute value of lambda is negative. For the second estimate, for each environmental variable in turn, the values of that variable on training presence and background data are randomly permuted. The model is reevaluated on the permuted data, and the resulting drop in training AUC is shown in the table, normalized to percentages. As with the variable jackknife, variable contributions should be interpreted with caution when the predictor variables are correlated.

Variable	Percent contribution	Permutation importance
canopy_cover_within_75m_ec	100	100

Raw data outputs and control parameters

The data used in the above analysis is contained in the next links. Please see the Help button for more information on these.
The model applied to the training environmental layers
The coefficients of the model
The omission and predicted area for varying cumulative and raw thresholds
The prediction strength at the training and (optionally) test presence sites
Results for all species modeled in the same Maxent run, with summary statistics and (optionally) jackknife results

Regularized training gain is 0.162, training AUC is 0.671, unregularized training gain is 0.186.
Unregularized test gain is 0.226.
Test AUC is 0.686, standard deviation is 0.033 (calculated as in DeLong, DeLong & Clarke-Pearson 1988, equation 2).
Algorithm converged after 300 iterations (0 seconds).

The follow settings were used during the run:
126 presence records used for training, 42 for testing.
10000 points used to determine the Maxent distribution (background points and presence points).
Environmental layers used (all continuous): canopy_cover_within_75m_ec
Regularization values: linear/quadratic/product: 0.050, categorical: 0.250, threshold: 1.000, hinge: 0.500
Feature types used: hinge linear threshold quadratic
responsecurves: true
pictures: false
outputdirectory: C:\Users\Bill\Desktop\Maxent Outputs EC 7-8-15
samplesfile: C:\Users\Bill\Desktop\Maxent_samples\Compare_NEC_EC_150m_311_WGS_84_m.csv
environmentallayers: C:\Users\Bill\Desktop\Maxent Layers\maxent.cache
randomseed: true
askoverwrite: false
randomtestpoints: 25
replicates: 10
replicatetype: subsample
Command line used:

Maxent model for EC_3

This page contains some analysis of the Maxent model for EC_3, created Wed Jul 08 11:05:11 EDT 2015 using Maxent version 3.3.3k. If you would like to do further analyses, the raw data used here is linked to at the end of this page.

Analysis of omission/commission

The following picture shows the omission rate and predicted area as a function of the cumulative threshold. The omission rate is is calculated both on the training presence records, and (if test data are used) on the test records. The omission rate should be close to the predicted omission, because of the definition of the cumulative threshold.

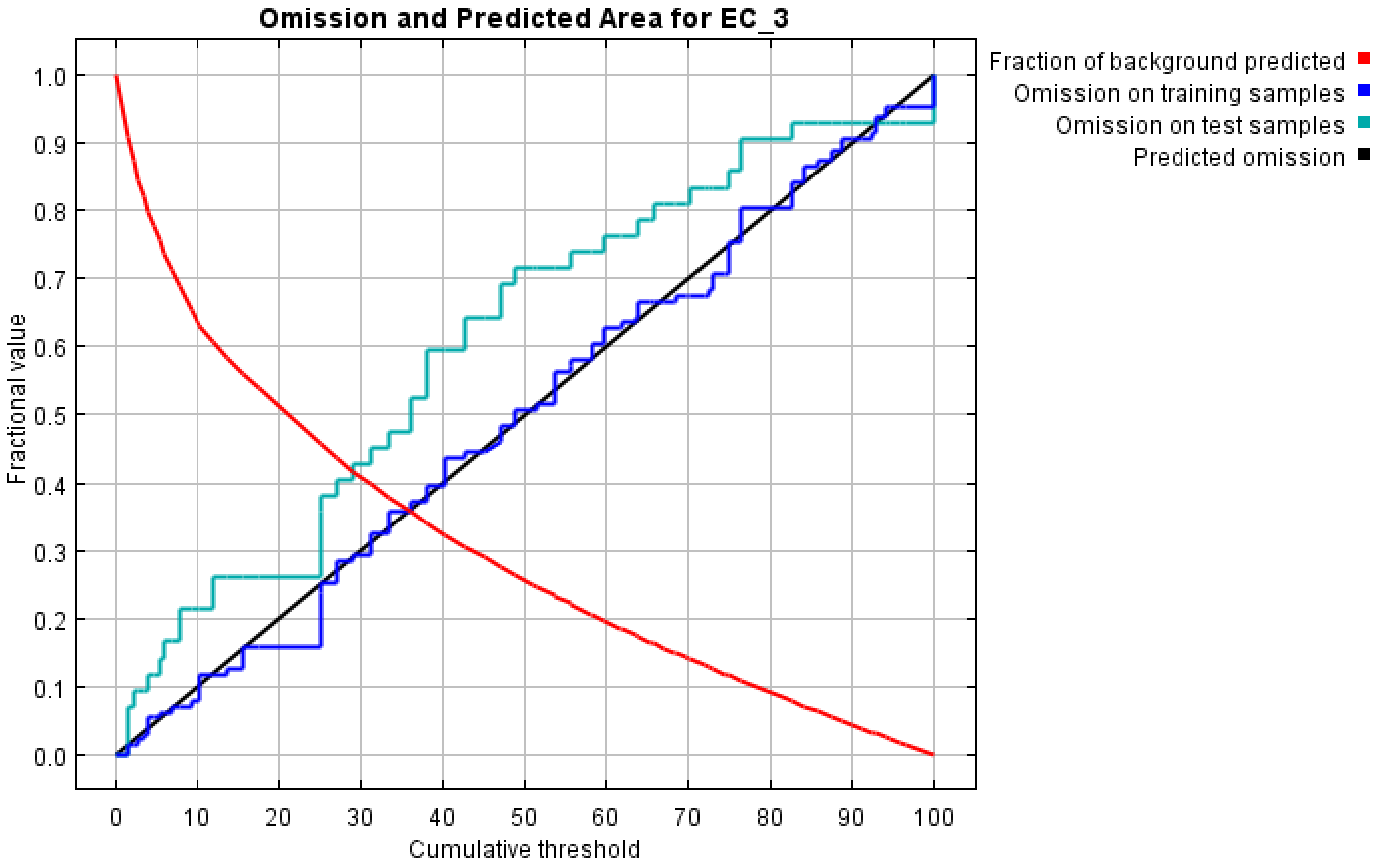

The next picture is the receiver operating characteristic (ROC) curve for the same data. Note that the specificity is defined using predicted area, rather than true commission (see the paper by Phillips, Anderson and Schapire cited on the help page for discussion of what this means). This implies that the maximum achievable AUC is less than 1. If test data is drawn from the Maxent distribution itself, then the maximum possible test AUC would be 0.693 rather than 1; in practice the test AUC may exceed this bound.

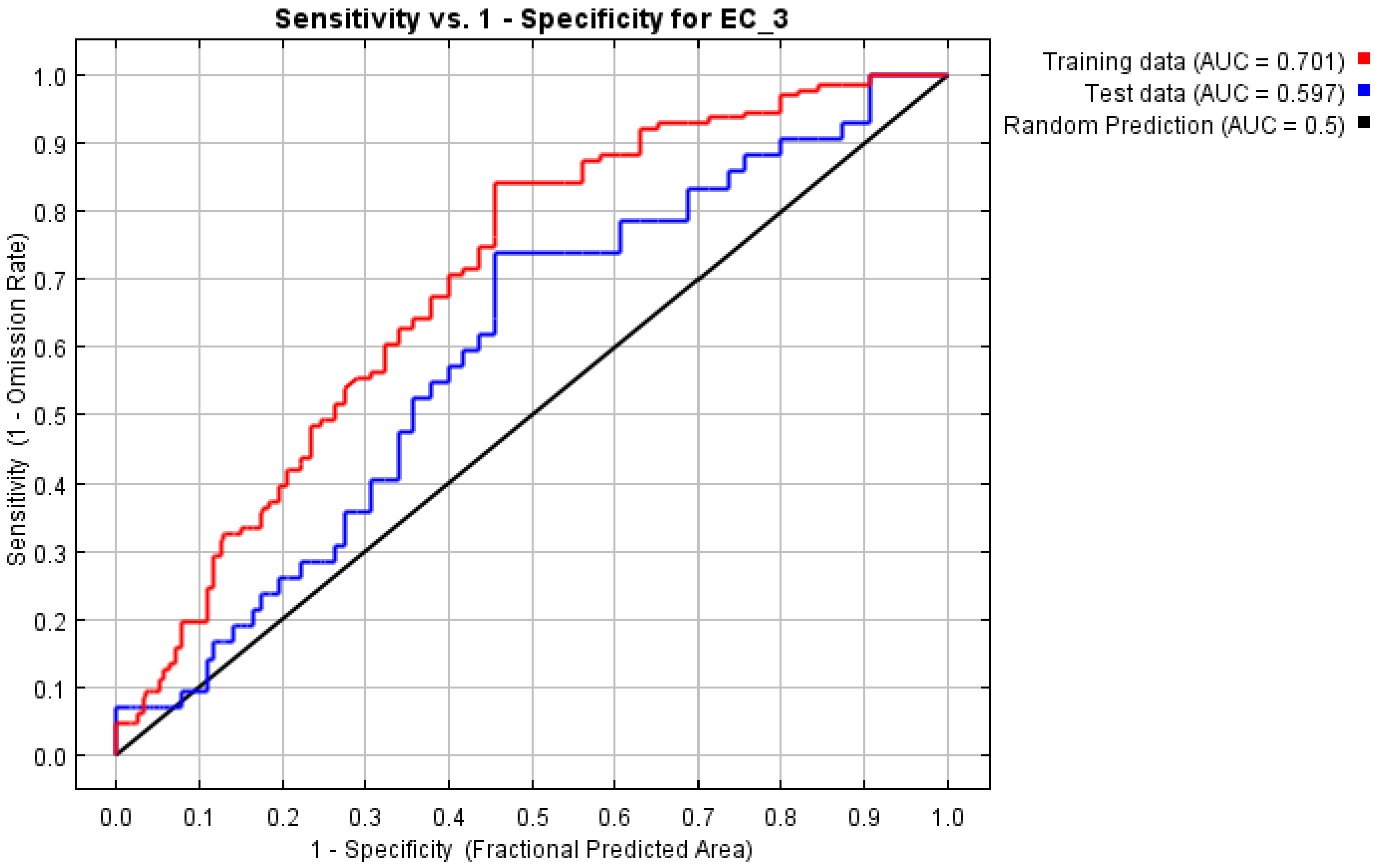

Some common thresholds and corresponding omission rates are as follows. If test data are available, binomial probabilities are calculated exactly if the number of test samples is at most 25, otherwise using a normal approximation to the binomial. These are 1-sided p-values for the null hypothesis that test points are predicted no better than by a random prediction with the same fractional predicted area. The "Balance" threshold minimizes 6 * training omission rate + .04 * cumulative threshold + 1.6 * fractional predicted area.

Cumulative threshold	Logistic threshold	Description	Fractional predicted area	Training omission rate	Test omission rate	P-value
1.000	0.115	Fixed cumulative value 1	0.908	0.000	0.000	1.95E-2
5.000	0.214	Fixed cumulative value 5	0.756	0.056	0.119	2.977E-2
10.000	0.360	Fixed cumulative value 10	0.630	0.079	0.214	1.825E-2
1.520	0.115	Minimum training presence	0.908	0.000	0.000	1.95E-2
10.361	0.360	10 percentile training presence	0.630	0.079	0.214	1.825E-2
36.132	0.496	Equal training sensitivity and specificity	0.357	0.357	0.476	1.193E-2
25.209	0.419	Maximum training sensitivity plus specificity	0.457	0.159	0.262	1.256E-4
		Equal test sensitivity and				

29.075	0.460	specificity	0.418	0.294	0.429	2.19E-2
25.209	0.419	Maximum test sensitivity plus specificity	0.457	0.159	0.262	1.256E-4
1.520	0.115	Balance training omission, predicted area and threshold value	0.908	0.000	0.000	1.95E-2
4.584	0.202	Equate entropy of thresholded and original distributions	0.777	0.056	0.119	5.223E-2

Click here to interactively explore this prediction using the Explain tool. If clicking from your browser does not succeed in starting the tool, try running the script in C:\Users\Bill\Desktop\Maxent Outputs EC 7-8-15\EC_3_explain.bat directly. This tool requires the environmental grids to be small enough that they all fit in memory.

Response curves

These curves show how each environmental variable affects the Maxent prediction. The curves show how the logistic prediction changes as each environmental variable is varied, keeping all other environmental variables at their average sample value. Click on a response curve to see a larger version. Note that the curves can be hard to interpret if you have strongly correlated variables, as the model may depend on the correlations in ways that are not evident in the curves. In other words, the curves show the marginal effect of changing exactly one variable, whereas the model may take advantage of sets of variables changing together.

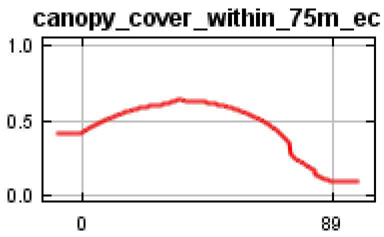

In contrast to the above marginal response curves, each of the following curves represents a different model, namely, a Maxent model created using only the corresponding variable. These plots reflect the dependence of predicted suitability both on the selected variable and on dependencies induced by correlations between the selected variable and other variables. They may be easier to interpret if there are strong correlations between variables.

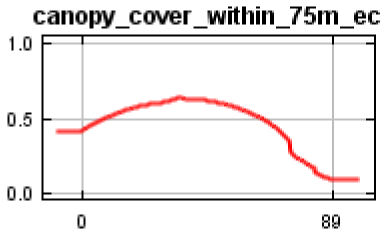

Analysis of variable contributions

The following table gives estimates of relative contributions of the environmental variables to the Maxent model. To determine the first estimate, in each iteration of the training algorithm, the increase in regularized gain is added to the contribution of the corresponding variable, or subtracted from it if the change to the absolute value of lambda is negative. For the second estimate, for each environmental variable in turn, the values of that variable on training presence and background data are randomly permuted. The model is reevaluated on the permuted data, and the resulting drop in training AUC is shown in the table, normalized to percentages. As with the variable jackknife, variable contributions should be interpreted with caution when the predictor variables are correlated.

Variable	Percent contribution	Permutation importance
canopy_cover_within_75m_ec	100	100

Raw data outputs and control parameters

The data used in the above analysis is contained in the next links. Please see the Help button for more information on these.
The model applied to the training environmental layers
The coefficients of the model
The omission and predicted area for varying cumulative and raw thresholds
The prediction strength at the training and (optionally) test presence sites
Results for all species modeled in the same Maxent run, with summary statistics and (optionally) jackknife results

Regularized training gain is 0.246, training AUC is 0.701, unregularized training gain is 0.273.
Unregularized test gain is -0.022.
Test AUC is 0.597, standard deviation is 0.041 (calculated as in DeLong, DeLong & Clarke-Pearson 1988, equation 2).
Algorithm converged after 440 iterations (1 seconds).

The follow settings were used during the run:
126 presence records used for training, 42 for testing.
10000 points used to determine the Maxent distribution (background points and presence points).
Environmental layers used (all continuous): canopy_cover_within_75m_ec
Regularization values: linear/quadratic/product: 0.050, categorical: 0.250, threshold: 1.000, hinge: 0.500
Feature types used: hinge linear threshold quadratic
responsecurves: true
pictures: false
outputdirectory: C:\Users\Bill\Desktop\Maxent Outputs EC 7-8-15
samplesfile: C:\Users\Bill\Desktop\Maxent_samples\Compare_NEC_EC_150m_311_WGS_84_m.csv
environmentallayers: C:\Users\Bill\Desktop\Maxent Layers\maxent.cache
randomseed: true
askoverwrite: false
randomtestpoints: 25
replicates: 10
replicatetype: subsample
Command line used:

Maxent model for EC_4

This page contains some analysis of the Maxent model for EC_4, created Wed Jul 08 11:11:26 EDT 2015 using Maxent version 3.3.3k. If you would like to do further analyses, the raw data used here is linked to at the end of this page.

Analysis of omission/commission

The following picture shows the omission rate and predicted area as a function of the cumulative threshold. The omission rate is is calculated both on the training presence records, and (if test data are used) on the test records. The omission rate should be close to the predicted omission, because of the definition of the cumulative threshold.

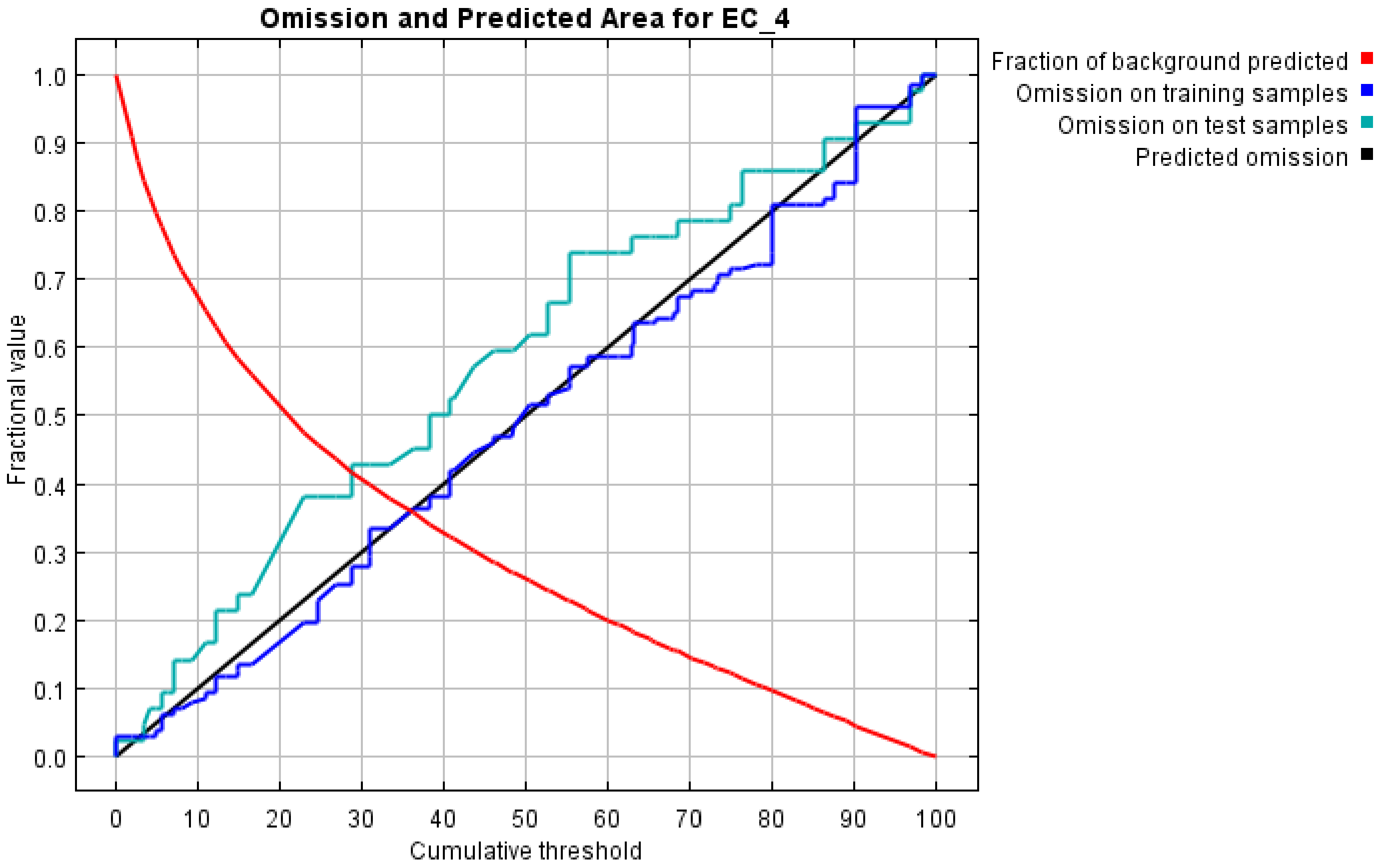

The next picture is the receiver operating characteristic (ROC) curve for the same data. Note that the specificity is defined using predicted area, rather than true commission (see the paper by Phillips, Anderson and Schapire cited on the help page for discussion of what this means). This implies that the maximum achievable AUC is less than 1. If test data is drawn from the Maxent distribution itself, then the maximum possible test AUC would be 0.686 rather than 1; in practice the test AUC may exceed this bound.

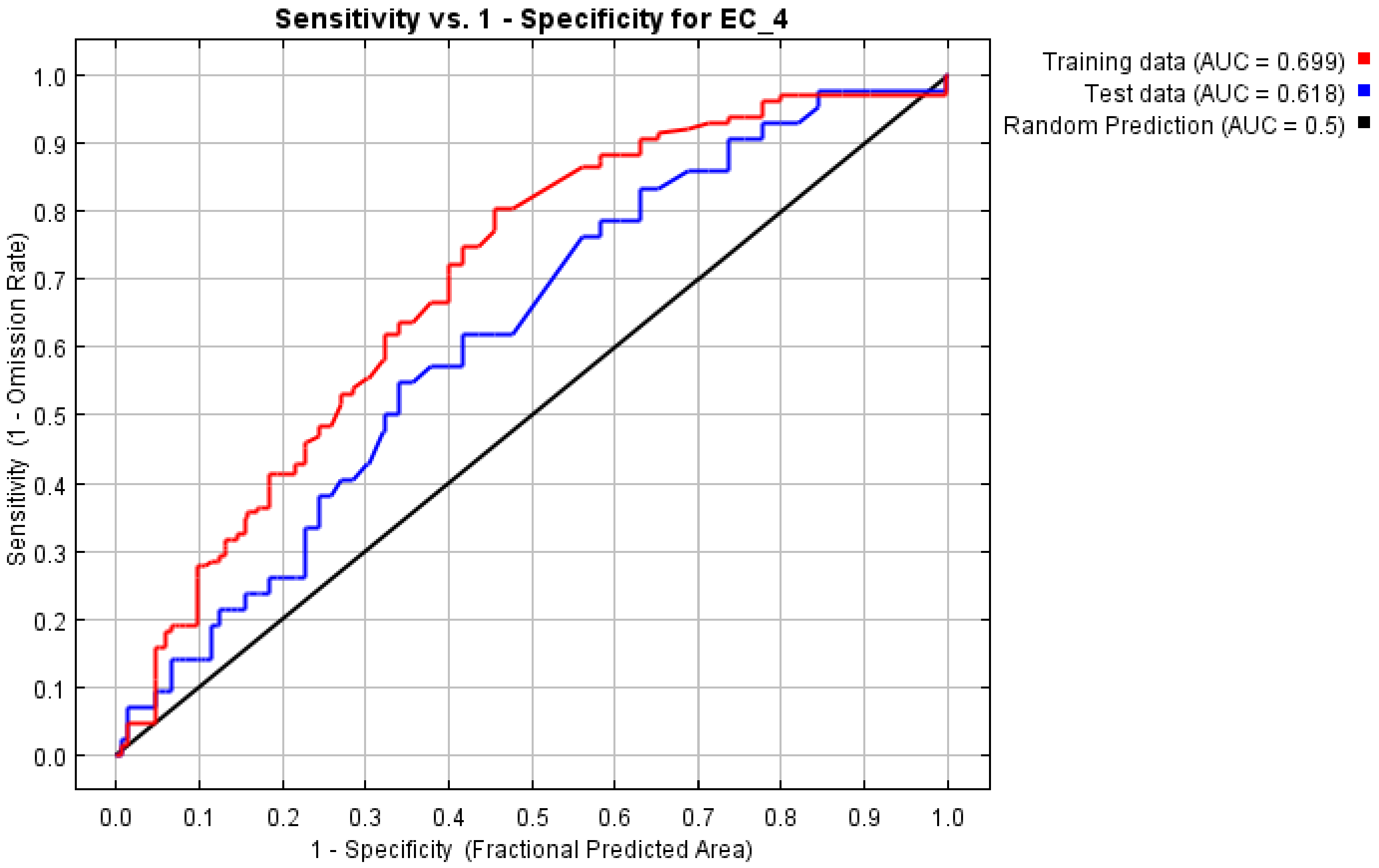

Some common thresholds and corresponding omission rates are as follows. If test data are available, binomial probabilities are calculated exactly if the number of test samples is at most 25, otherwise using a normal approximation to the binomial. These are 1-sided p-values for the null hypothesis that test points are predicted no better than by a random prediction with the same fractional predicted area. The "Balance" threshold minimizes 6 * training omission rate + .04 * cumulative threshold + 1.6 * fractional predicted area.

Cumulative threshold	Logistic threshold	Description	Fractional predicted area	Training omission rate	Test omission rate	P-value
1.000	0.156	Fixed cumulative value 1	0.908	0.032	0.024	6.295E-2
5.000	0.209	Fixed cumulative value 5	0.777	0.040	0.071	9.026E-3
10.000	0.301	Fixed cumulative value 10	0.654	0.087	0.167	7.207E-3
0.047	0.145	Minimum training presence	0.998	0.000	0.000	3.753E-1
12.177	0.319	10 percentile training presence	0.630	0.095	0.167	3.161E-3
36.302	0.514	Equal training sensitivity and specificity	0.357	0.365	0.452	4.92E-3
24.728	0.430	Maximum training sensitivity plus	0.457	0.198	0.381	1.733E-

		specificity				2
28.809	0.481	Equal test sensitivity and specificity	0.418	0.278	0.429	2.19E-2
38.353	0.514	Maximum test sensitivity plus specificity	0.341	0.365	0.452	2.389E-3
0.047	0.145	Balance training omission, predicted area and threshold value	0.998	0.000	0.000	3.753E-1
5.533	0.209	Equate entropy of thresholded and original distributions	0.777	0.040	0.071	9.026E-3

Click here to interactively explore this prediction using the Explain tool. If clicking from your browser does not succeed in starting the tool, try running the script in C:\Users\Bill\Desktop\Maxent Outputs EC 7-8-15\EC_4_explain.bat directly. This tool requires the environmental grids to be small enough that they all fit in memory.

Response curves

These curves show how each environmental variable affects the Maxent prediction. The curves show how the logistic prediction changes as each environmental variable is varied, keeping all other environmental variables at their average sample value. Click on a response curve to see a larger version. Note that the curves can be hard to interpret if you have strongly correlated variables, as the model may depend on the correlations in ways that are not evident in the curves. In other words, the curves show the marginal effect of changing exactly one variable, whereas the model may take advantage of sets of variables changing together.

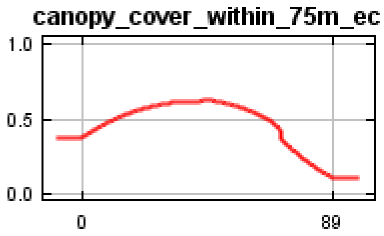

In contrast to the above marginal response curves, each of the following curves represents a different model, namely, a Maxent model created using only the corresponding variable. These plots reflect the dependence of predicted suitability both on the selected variable and on dependencies induced by correlations between the selected variable and other variables. They may be easier to interpret if there are strong correlations between variables.

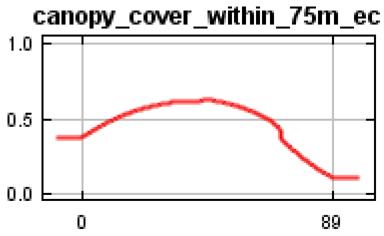

Analysis of variable contributions

The following table gives estimates of relative contributions of the environmental variables to the Maxent model. To determine the first estimate, in each iteration of the training algorithm, the increase in regularized gain is added to the contribution of the corresponding variable, or subtracted from it if the change to the absolute value of lambda is negative. For the second estimate, for each environmental variable in turn, the values of that variable on training presence and background data are randomly permuted. The model is reevaluated on the permuted data, and the resulting drop in training AUC is shown in the table, normalized to percentages. As with the variable jackknife, variable contributions should be interpreted with caution when the predictor variables are correlated.

Variable	Percent contribution	Permutation importance
canopy_cover_within_75m_ec	100	100

Raw data outputs and control parameters

The data used in the above analysis is contained in the next links. Please see the Help button for more information on these.
The model applied to the training environmental layers
The coefficients of the model
The omission and predicted area for varying cumulative and raw thresholds
The prediction strength at the training and (optionally) test presence sites
Results for all species modeled in the same Maxent run, with summary statistics and (optionally) jackknife results

Regularized training gain is 0.226, training AUC is 0.699, unregularized training gain is 0.253.
Unregularized test gain is 0.046.
Test AUC is 0.618, standard deviation is 0.039 (calculated as in DeLong, DeLong & Clarke-Pearson 1988, equation 2).
Algorithm converged after 480 iterations (1 seconds).

The follow settings were used during the run:
126 presence records used for training, 42 for testing.
10000 points used to determine the Maxent distribution (background points and presence points).
Environmental layers used (all continuous): canopy_cover_within_75m_ec
Regularization values: linear/quadratic/product: 0.050, categorical: 0.250, threshold: 1.000, hinge: 0.500
Feature types used: hinge linear threshold quadratic
responsecurves: true
pictures: false
outputdirectory: C:\Users\Bill\Desktop\Maxent Outputs EC 7-8-15
samplesfile: C:\Users\Bill\Desktop\Maxent_samples\Compare_NEC_EC_150m_311_WGS_84_m.csv
environmentallayers: C:\Users\Bill\Desktop\Maxent Layers\maxent.cache
randomseed: true
askoverwrite: false
randomtestpoints: 25
replicates: 10
replicatetype: subsample

Maxent model for EC_5

This page contains some analysis of the Maxent model for EC_5, created Wed Jul 08 11:17:44 EDT 2015 using Maxent version 3.3.3k. If you would like to do further analyses, the raw data used here is linked to at the end of this page.

Analysis of omission/commission

The following picture shows the omission rate and predicted area as a function of the cumulative threshold. The omission rate is is calculated both on the training presence records, and (if test data are used) on the test records. The omission rate should be close to the predicted omission, because of the definition of the cumulative threshold.

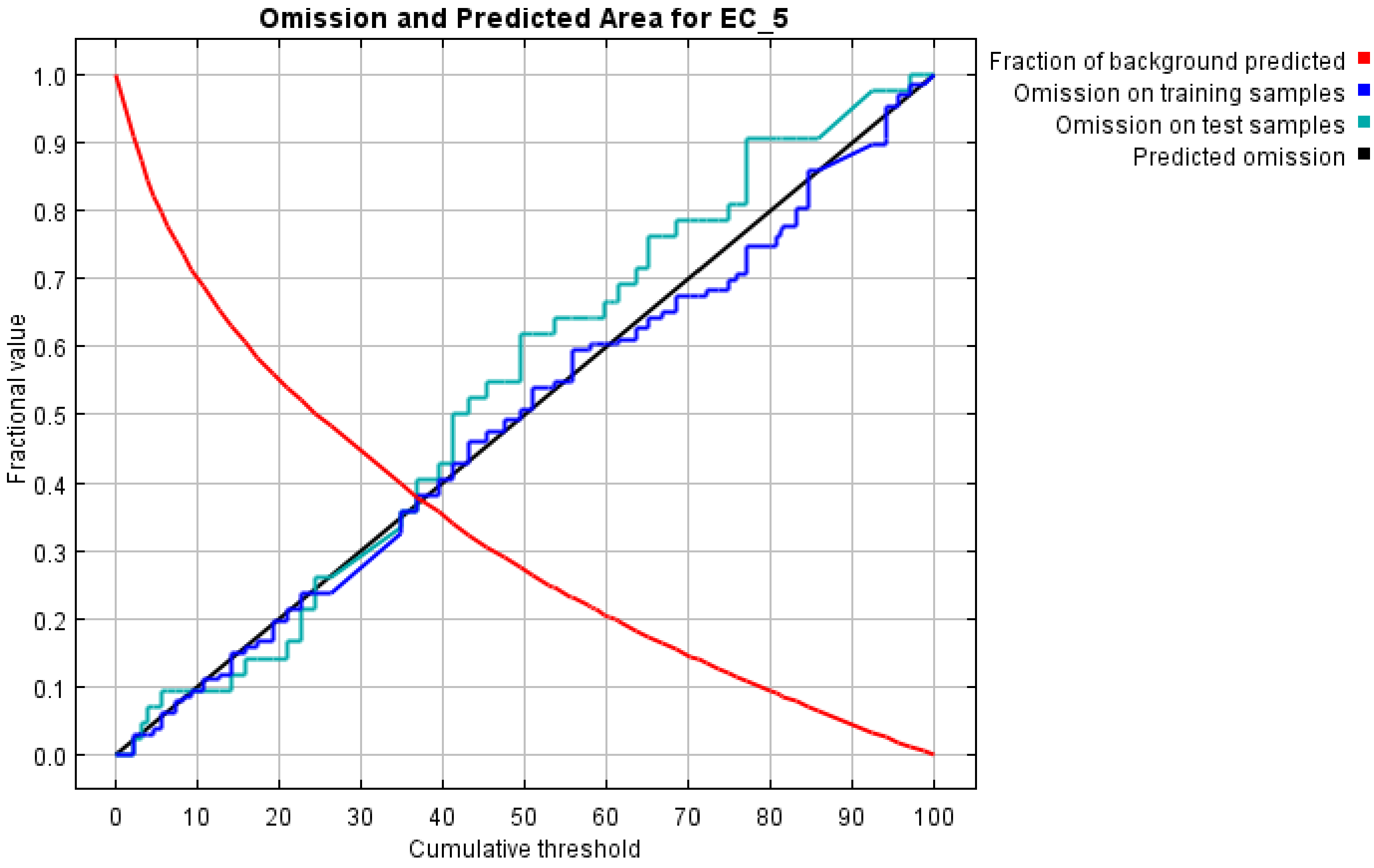

The next picture is the receiver operating characteristic (ROC) curve for the same data. Note that the specificity is defined using predicted area, rather than true commission (see the paper by Phillips, Anderson and Schapire cited on the help page for discussion of what this means). This implies that the maximum achievable AUC is less than 1. If test data is drawn from the Maxent distribution itself, then the maximum possible test AUC would be 0.672 rather than 1; in practice the test AUC may exceed this bound.

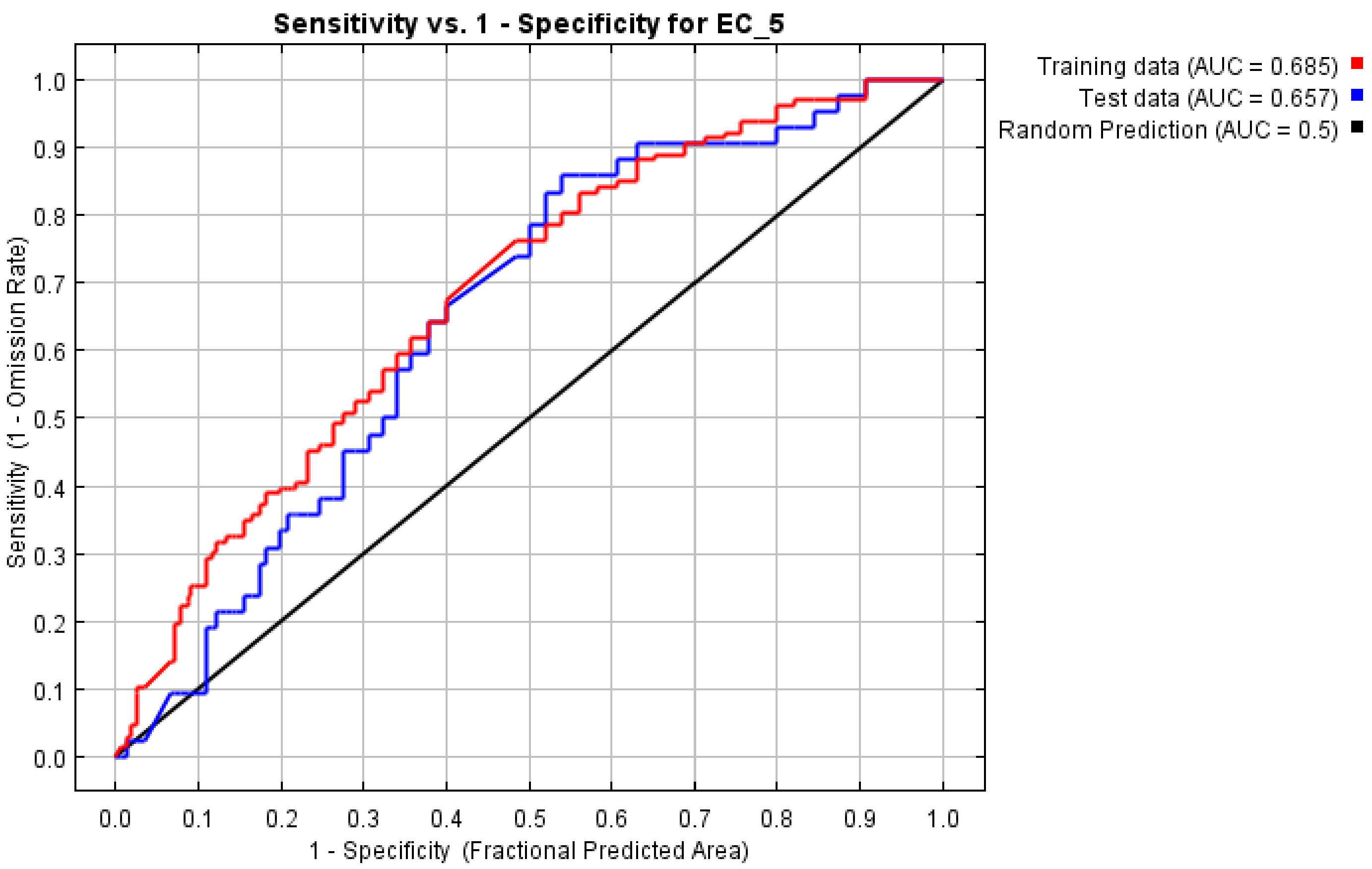

Some common thresholds and corresponding omission rates are as follows. If test data are available, binomial probabilities are calculated exactly if the number of test samples is at most 25, otherwise using a normal approximation to the binomial. These are 1-sided p-values for the null hypothesis that test points are predicted no better than by a random prediction with the same fractional predicted area. The "Balance" threshold minimizes 6 * training omission rate + .04 * cumulative threshold + 1.6 * fractional predicted area.

Cumulative threshold	Logistic threshold	Description	Fractional predicted area	Training omission rate	Test omission rate	P-value
1.000	0.167	Fixed cumulative value 1	0.908	0.000	0.000	1.95E-2
5.000	0.247	Fixed cumulative value 5	0.798	0.040	0.071	1.774E-2
10.000	0.326	Fixed cumulative value 10	0.688	0.095	0.095	1.201E-3
2.222	0.167	Minimum training presence	0.908	0.000	0.000	1.95E-2
10.713	0.326	10 percentile training presence	0.688	0.095	0.095	1.201E-3
36.936	0.475	Equal training sensitivity and specificity	0.379	0.381	0.405	1.971E-3
26.321	0.457	Maximum training sensitivity plus specificity	0.483	0.238	0.262	4.76E-4
		Equal test sensitivity and				2.168E-

36.936	0.475	specificity	0.379	0.357	0.357	4
20.930	0.425	Maximum test sensitivity plus specificity	0.541	0.198	0.143	1.934E-5
2.222	0.167	Balance training omission, predicted area and threshold value	0.908	0.000	0.000	1.95E-2
4.723	0.232	Equate entropy of thresholded and original distributions	0.820	0.032	0.071	3.371E-2

Click here to interactively explore this prediction using the Explain tool. If clicking from your browser does not succeed in starting the tool, try running the script in C:\Users\Bill\Desktop\Maxent Outputs EC 7-8-15\EC_5_explain.bat directly. This tool requires the environmental grids to be small enough that they all fit in memory.

Response curves

These curves show how each environmental variable affects the Maxent prediction. The curves show how the logistic prediction changes as each environmental variable is varied, keeping all other environmental variables at their average sample value. Click on a response curve to see a larger version. Note that the curves can be hard to interpret if you have strongly correlated variables, as the model may depend on the correlations in ways that are not evident in the curves. In other words, the curves show the marginal effect of changing exactly one variable, whereas the model may take advantage of sets of variables changing together.

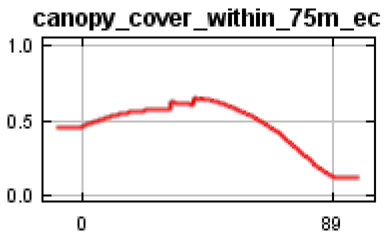

-

In contrast to the above marginal response curves, each of the following curves represents a different model, namely, a Maxent model created using only the corresponding variable. These plots reflect the dependence of predicted suitability both on the selected variable and on dependencies induced by correlations between the selected variable and other variables. They may be easier to interpret if there are strong correlations between variables.

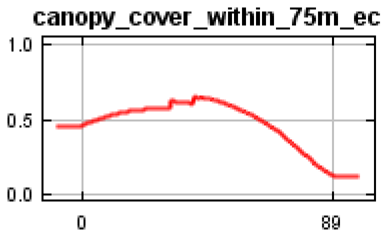

-

Analysis of variable contributions

The following table gives estimates of relative contributions of the environmental variables to the Maxent model. To determine the first estimate, in each iteration of the training algorithm, the increase in regularized gain is added to the contribution of the corresponding variable, or subtracted from it if the change to the absolute value of lambda is negative. For the second estimate, for each environmental variable in turn, the values of that variable on training presence and background data are randomly permuted. The model is reevaluated on the permuted data, and the resulting drop in training AUC is shown in the table, normalized to percentages. As with the variable jackknife, variable contributions should be interpreted with caution when the predictor variables are correlated.

Variable	Percent contribution	Permutation importance
canopy_cover_within_75m_ec	100	100

Raw data outputs and control parameters

The data used in the above analysis is contained in the next links. Please see the Help button for more information on these.
The model applied to the training environmental layers
The coefficients of the model
The omission and predicted area for varying cumulative and raw thresholds
The prediction strength at the training and (optionally) test presence sites
Results for all species modeled in the same Maxent run, with summary statistics and (optionally) jackknife results

Regularized training gain is 0.188, training AUC is 0.685, unregularized training gain is 0.214.
Unregularized test gain is 0.158.
Test AUC is 0.657, standard deviation is 0.036 (calculated as in DeLong, DeLong & Clarke-Pearson 1988, equation 2).
Algorithm converged after 420 iterations (1 seconds).

The follow settings were used during the run:
126 presence records used for training, 42 for testing.
10000 points used to determine the Maxent distribution (background points and presence points).
Environmental layers used (all continuous): canopy_cover_within_75m_ec
Regularization values: linear/quadratic/product: 0.050, categorical: 0.250, threshold: 1.000, hinge: 0.500
Feature types used: hinge linear threshold quadratic
responsecurves: true
pictures: false
outputdirectory: C:\Users\Bill\Desktop\Maxent Outputs EC 7-8-15
samplesfile: C:\Users\Bill\Desktop\Maxent_samples\Compare_NEC_EC_150m_311_WGS_84_m.csv
environmentallayers: C:\Users\Bill\Desktop\Maxent Layers\maxent.cache
randomseed: true
askoverwrite: false
randomtestpoints: 25
replicates: 10
replicatetype: subsample
Command line used:

Maxent model for EC_6

This page contains some analysis of the Maxent model for EC_6, created Wed Jul 08 11:24:06 EDT 2015 using Maxent version 3.3.3k. If you would like to do further analyses, the raw data used here is linked to at the end of this page.

Analysis of omission/commission

The following picture shows the omission rate and predicted area as a function of the cumulative threshold. The omission rate is is calculated both on the training presence records, and (if test data are used) on the test records. The omission rate should be close to the predicted omission, because of the definition of the cumulative threshold.

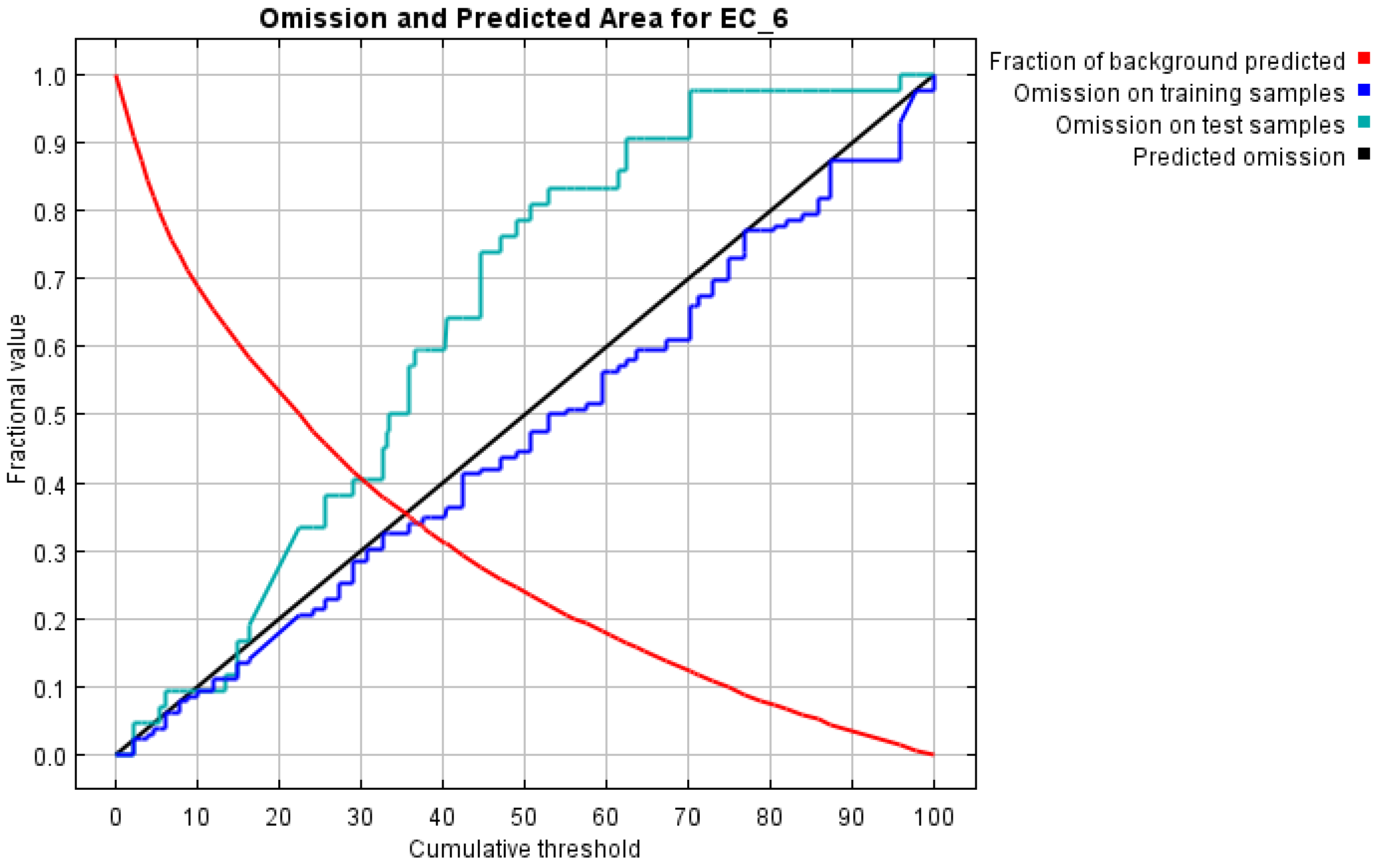

The next picture is the receiver operating characteristic (ROC) curve for the same data. Note that the specificity is defined using predicted area, rather than true commission (see the paper by Phillips, Anderson and Schapire cited on the help page for discussion of what this means). This implies that the maximum achievable AUC is less than 1. If test data is drawn from the Maxent distribution itself, then the maximum possible test AUC would be 0.694 rather than 1; in practice the test AUC may exceed this bound.

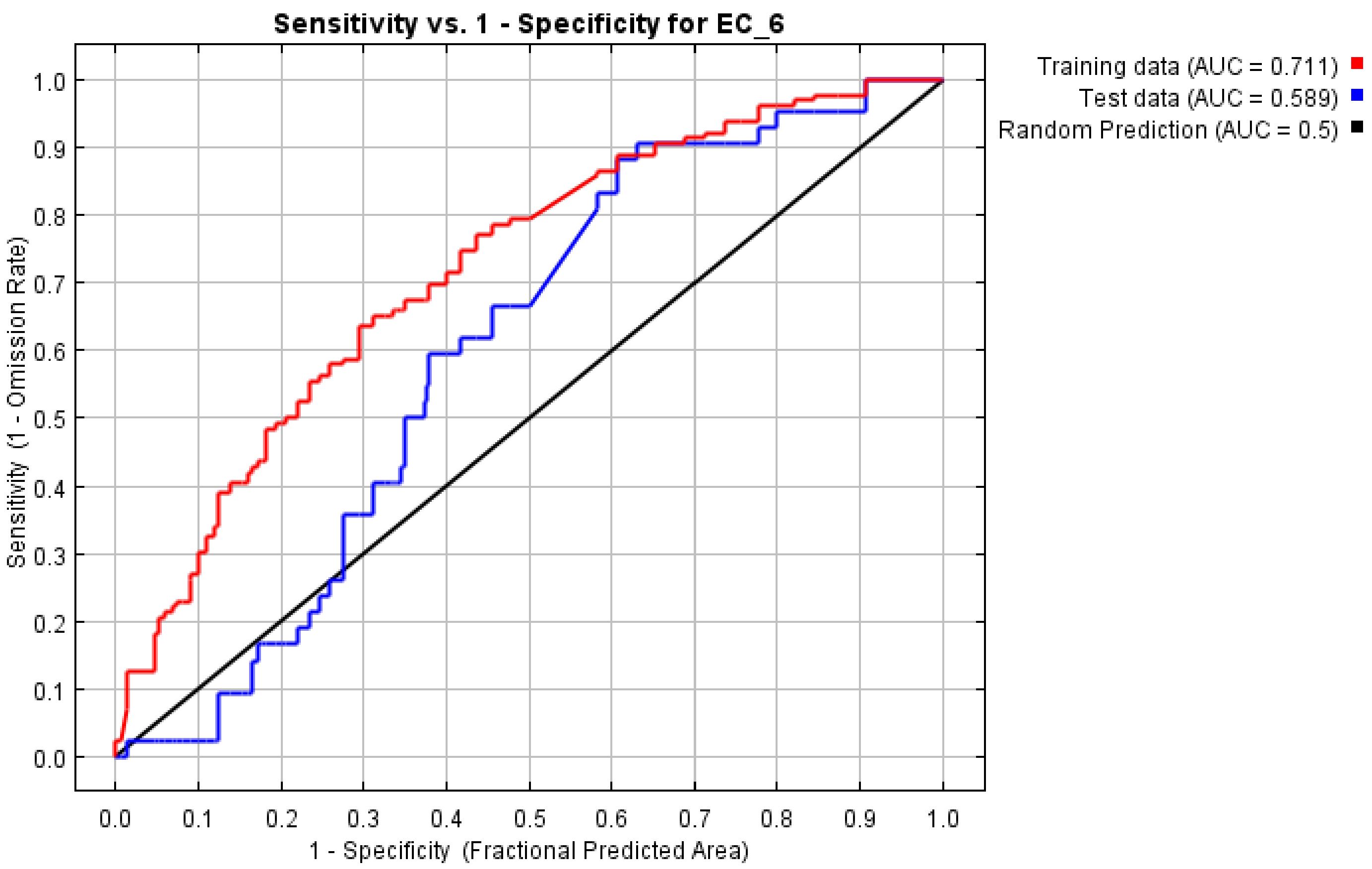

Some common thresholds and corresponding omission rates are as follows. If test data are available, binomial probabilities are calculated exactly if the number of test samples is at most 25, otherwise using a normal approximation to the binomial. These are 1-sided p-values for the null hypothesis that test points are predicted no better than by a random prediction with the same fractional predicted area. The "Balance" threshold minimizes 6 * training omission rate + .04 * cumulative threshold + 1.6 * fractional predicted area.

Cumulative threshold	Logistic threshold	Description	Fractional predicted area	Training omission rate	Test omission rate	P-value
1.000	0.155	Fixed cumulative value 1	0.908	0.000	0.000	1.95E-2
5.000	0.208	Fixed cumulative value 5	0.798	0.040	0.048	6.435E-3
10.000	0.283	Fixed cumulative value 10	0.688	0.087	0.095	1.201E-3
2.142	0.155	Minimum training presence	0.908	0.000	0.000	1.95E-2
11.930	0.299	10 percentile training presence	0.654	0.095	0.095	3.134E-4
36.943	0.468	Equal training sensitivity and specificity	0.341	0.341	0.595	1.925E-1
42.447	0.490	Maximum training sensitivity plus specificity	0.294	0.365	0.643	1.841E-1
		Equal test sensitivity and				

30.764	0.430	specificity	0.399	0.286	0.405	4.76E-3
13.323	0.316	Maximum test sensitivity plus specificity	0.630	0.111	0.095	1.124E-4
2.142	0.155	Balance training omission, predicted area and threshold value	0.908	0.000	0.000	1.95E-2
6.085	0.222	Equate entropy of thresholded and original distributions	0.777	0.040	0.071	9.026E-3

Click here to interactively explore this prediction using the Explain tool. If clicking from your browser does not succeed in starting the tool, try running the script in C:\Users\Bill\Desktop\Maxent Outputs EC 7-8-15\EC_6_explain.bat directly. This tool requires the environmental grids to be small enough that they all fit in memory.

Response curves

These curves show how each environmental variable affects the Maxent prediction. The curves show how the logistic prediction changes as each environmental variable is varied, keeping all other environmental variables at their average sample value. Click on a response curve to see a larger version. Note that the curves can be hard to interpret if you have strongly correlated variables, as the model may depend on the correlations in ways that are not evident in the curves. In other words, the curves show the marginal effect of changing exactly one variable, whereas the model may take advantage of sets of variables changing together.

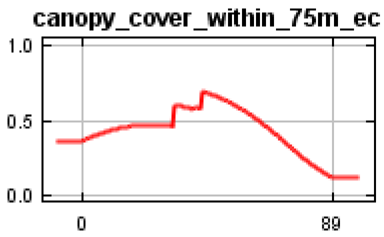

-

In contrast to the above marginal response curves, each of the following curves represents a different model, namely, a Maxent model created using only the corresponding variable. These plots reflect the dependence of predicted suitability both on the selected variable and on dependencies induced by correlations between the selected variable and other variables. They may be easier to interpret if there are strong correlations between variables.

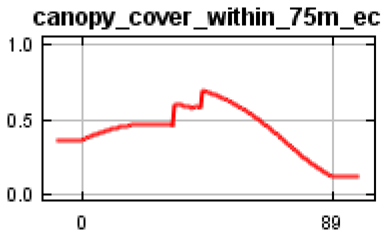

-

Analysis of variable contributions

The following table gives estimates of relative contributions of the environmental variables to the Maxent model. To determine the first estimate, in each iteration of the training algorithm, the increase in regularized gain is added to the contribution of the corresponding variable, or subtracted from it if the change to the absolute value of lambda is negative. For the second estimate, for each environmental variable in turn, the values of that variable on training presence and background data are randomly permuted. The model is reevaluated on the permuted data, and the resulting drop in training AUC is shown in the table, normalized to percentages. As with the variable jackknife, variable contributions should be interpreted with caution when the predictor variables are correlated.

Variable	Percent contribution	Permutation importance
canopy_cover_within_75m_ec	100	100

Raw data outputs and control parameters

The data used in the above analysis is contained in the next links. Please see the Help button for more information on these.
The model applied to the training environmental layers
The coefficients of the model
The omission and predicted area for varying cumulative and raw thresholds
The prediction strength at the training and (optionally) test presence sites
Results for all species modeled in the same Maxent run, with summary statistics and (optionally) jackknife results

Regularized training gain is 0.239, training AUC is 0.711, unregularized training gain is 0.282.
Unregularized test gain is -0.035.
Test AUC is 0.589, standard deviation is 0.033 (calculated as in DeLong, DeLong & Clarke-Pearson 1988, equation 2).
Algorithm converged after 480 iterations (1 seconds).

The follow settings were used during the run:
126 presence records used for training, 42 for testing.
10000 points used to determine the Maxent distribution (background points and presence points).
Environmental layers used (all continuous): canopy_cover_within_75m_ec
Regularization values: linear/quadratic/product: 0.050, categorical: 0.250, threshold: 1.000, hinge: 0.500
Feature types used: hinge linear threshold quadratic
responsecurves: true
pictures: false
outputdirectory: C:\Users\Bill\Desktop\Maxent Outputs EC 7-8-15
samplesfile: C:\Users\Bill\Desktop\Maxent_samples\Compare_NEC_EC_150m_311_WGS_84_m.csv
environmentallayers: C:\Users\Bill\Desktop\Maxent Layers\maxent.cache
randomseed: true
askoverwrite: false
randomtestpoints: 25
replicates: 10
replicatetype: subsample
Command line used:

Maxent model for EC_7

This page contains some analysis of the Maxent model for EC_7, created Wed Jul 08 11:30:21 EDT 2015 using Maxent version 3.3.3k. If you would like to do further analyses, the raw data used here is linked to at the end of this page.

Analysis of omission/commission

The following picture shows the omission rate and predicted area as a function of the cumulative threshold. The omission rate is is calculated both on the training presence records, and (if test data are used) on the test records. The omission rate should be close to the predicted omission, because of the definition of the cumulative threshold.

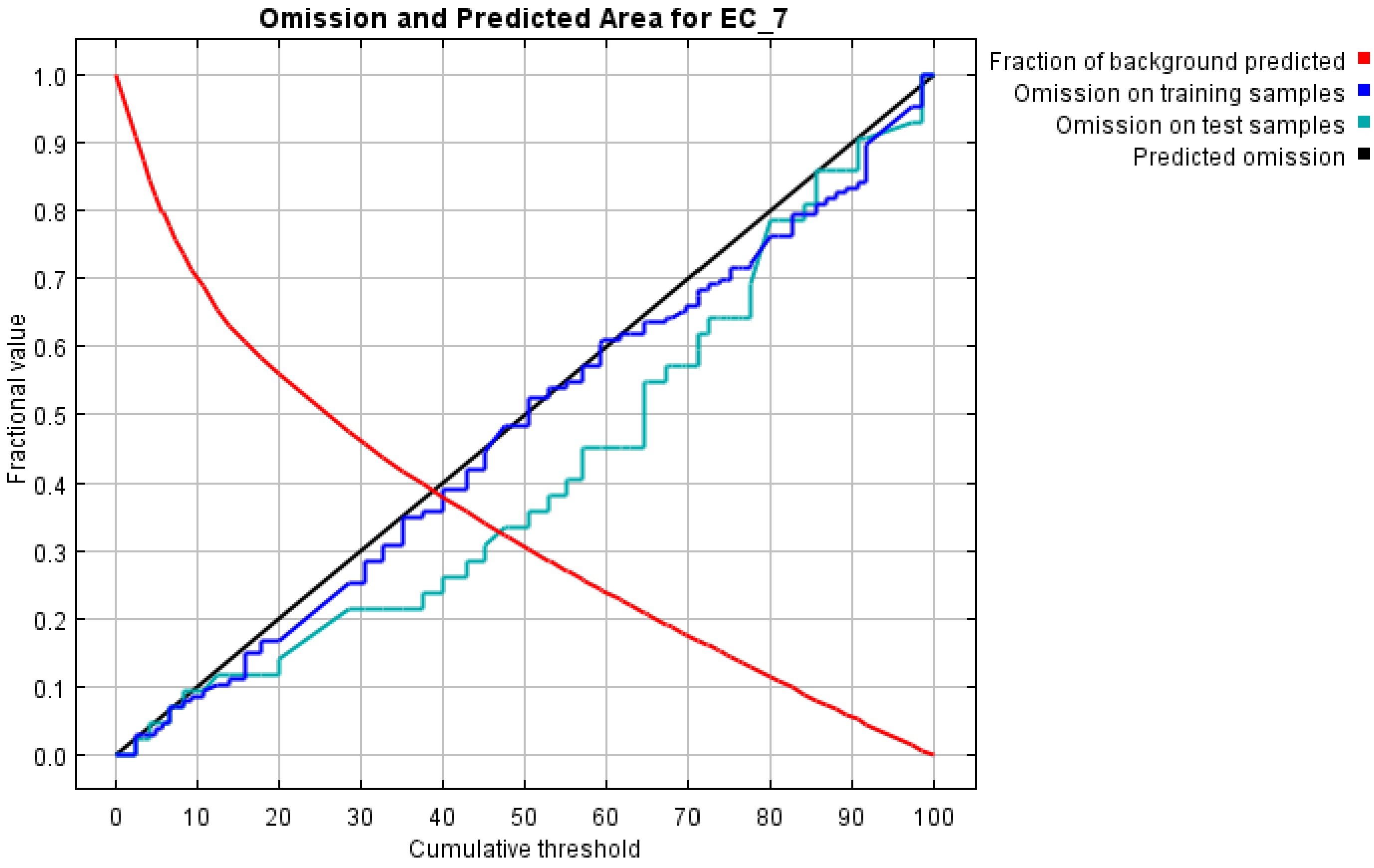

The next picture is the receiver operating characteristic (ROC) curve for the same data. Note that the specificity is defined using predicted area, rather than true commission (see the paper by Phillips, Anderson and Schapire cited on the help page for discussion of what this means). This implies that the maximum achievable AUC is less than 1. If test data is drawn from the Maxent distribution itself, then the maximum possible test AUC would be 0.654 rather than 1; in practice the test AUC may exceed this bound.

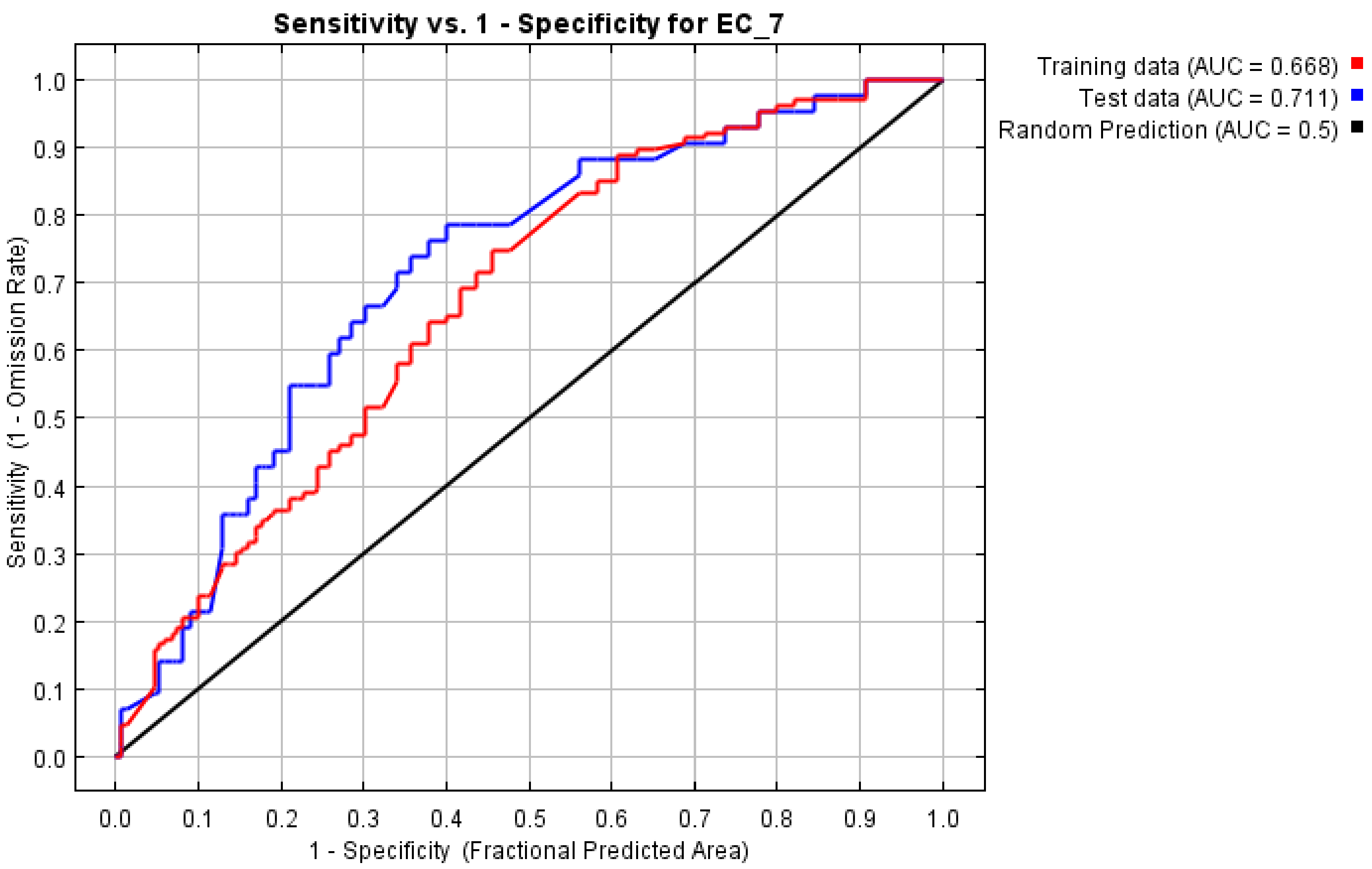

Some common thresholds and corresponding omission rates are as follows. If test data are available, binomial probabilities are calculated exactly if the number of test samples is at most 25, otherwise using a normal approximation to the binomial. These are 1-sided p-values for the null hypothesis that test points are predicted no better than by a random prediction with the same fractional predicted area. The "Balance" threshold minimizes 6 * training omission rate + .04 * cumulative threshold + 1.6 * fractional predicted area.

Cumulative threshold	Logistic threshold	Description	Fractional predicted area	Training omission rate	Test omission rate	P-value
1.000	0.179	Fixed cumulative value 1	0.908	0.000	0.000	1.95E-2
5.000	0.230	Fixed cumulative value 5	0.798	0.040	0.048	6.435E-3
10.000	0.303	Fixed cumulative value 10	0.688	0.087	0.095	1.201E-3
2.355	0.179	Minimum training presence	0.908	0.000	0.000	1.95E-2
10.665	0.319	10 percentile training presence	0.688	0.095	0.095	1.201E-3
40.010	0.530	Equal training sensitivity and specificity	0.379	0.389	0.262	8.312E-7
30.565	0.477	Maximum training sensitivity plus specificity	0.457	0.254	0.214	9.32E-6
		Equal test sensitivity and				1.051E-

47.508	0.542	specificity	0.324	0.484	0.333	6
37.439	0.515	Maximum test sensitivity plus specificity	0.399	0.349	0.214	1.583E-7
2.355	0.179	Balance training omission, predicted area and threshold value	0.908	0.000	0.000	1.95E-2
4.191	0.204	Equate entropy of thresholded and original distributions	0.844	0.032	0.024	9.142E-3

Click here to interactively explore this prediction using the Explain tool. If clicking from your browser does not succeed in starting the tool, try running the script in C:\Users\Bill\Desktop\Maxent Outputs EC 7-8-15\EC_7_explain.bat directly. This tool requires the environmental grids to be small enough that they all fit in memory.

Response curves

These curves show how each environmental variable affects the Maxent prediction. The curves show how the logistic prediction changes as each environmental variable is varied, keeping all other environmental variables at their average sample value. Click on a response curve to see a larger version. Note that the curves can be hard to interpret if you have strongly correlated variables, as the model may depend on the correlations in ways that are not evident in the curves. In other words, the curves show the marginal effect of changing exactly one variable, whereas the model may take advantage of sets of variables changing together.

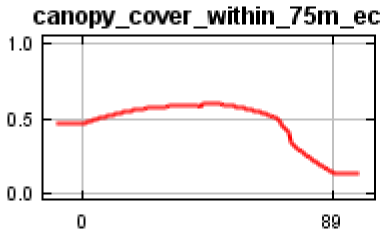

In contrast to the above marginal response curves, each of the following curves represents a different model, namely, a Maxent model created using only the corresponding variable. These plots reflect the dependence of predicted suitability both on the selected variable and on dependencies induced by correlations between the selected variable and other variables. They may be easier to interpret if there are strong correlations between variables.

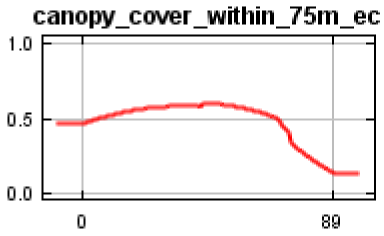

Analysis of variable contributions

The following table gives estimates of relative contributions of the environmental variables to the Maxent model. To determine the first estimate, in each iteration of the training algorithm, the increase in regularized gain is added to the contribution of the corresponding variable, or subtracted from it if the change to the absolute value of lambda is negative. For the second estimate, for each environmental variable in turn, the values of that variable on training presence and background data are randomly permuted. The model is reevaluated on the permuted data, and the resulting drop in training AUC is shown in the table, normalized to percentages. As with the variable jackknife, variable contributions should be interpreted with caution when the predictor variables are correlated.

Variable	Percent contribution	Permutation importance
canopy_cover_within_75m_ec	100	100

Raw data outputs and control parameters

The data used in the above analysis is contained in the next links. Please see the Help button for more information on these.
The model applied to the training environmental layers
The coefficients of the model
The omission and predicted area for varying cumulative and raw thresholds
The prediction strength at the training and (optionally) test presence sites
Results for all species modeled in the same Maxent run, with summary statistics and (optionally) jackknife results

Regularized training gain is 0.161, training AUC is 0.668, unregularized training gain is 0.188.
Unregularized test gain is 0.240.
Test AUC is 0.711, standard deviation is 0.037 (calculated as in DeLong, DeLong & Clarke-Pearson 1988, equation 2).
Algorithm converged after 320 iterations (1 seconds).

The follow settings were used during the run:
126 presence records used for training, 42 for testing.
10000 points used to determine the Maxent distribution (background points and presence points).
Environmental layers used (all continuous): canopy_cover_within_75m_ec
Regularization values: linear/quadratic/product: 0.050, categorical: 0.250, threshold: 1.000, hinge: 0.500
Feature types used: hinge linear threshold quadratic
responsecurves: true
pictures: false
outputdirectory: C:\Users\Bill\Desktop\Maxent Outputs EC 7-8-15
samplesfile: C:\Users\Bill\Desktop\Maxent_samples\Compare_NEC_EC_150m_311_WGS_84_m.csv
environmentallayers: C:\Users\Bill\Desktop\Maxent Layers\maxent.cache
randomseed: true
askoverwrite: false
randomtestpoints: 25
replicates: 10
replicatetype: subsample
Command line used:

Maxent model for EC_8

This page contains some analysis of the Maxent model for EC_8, created Wed Jul 08 11:36:40 EDT 2015 using Maxent version 3.3.3k. If you would like to do further analyses, the raw data used here is linked to at the end of this page.

Analysis of omission/commission

The following picture shows the omission rate and predicted area as a function of the cumulative threshold. The omission rate is is calculated both on the training presence records, and (if test data are used) on the test records. The omission rate should be close to the predicted omission, because of the definition of the cumulative threshold.

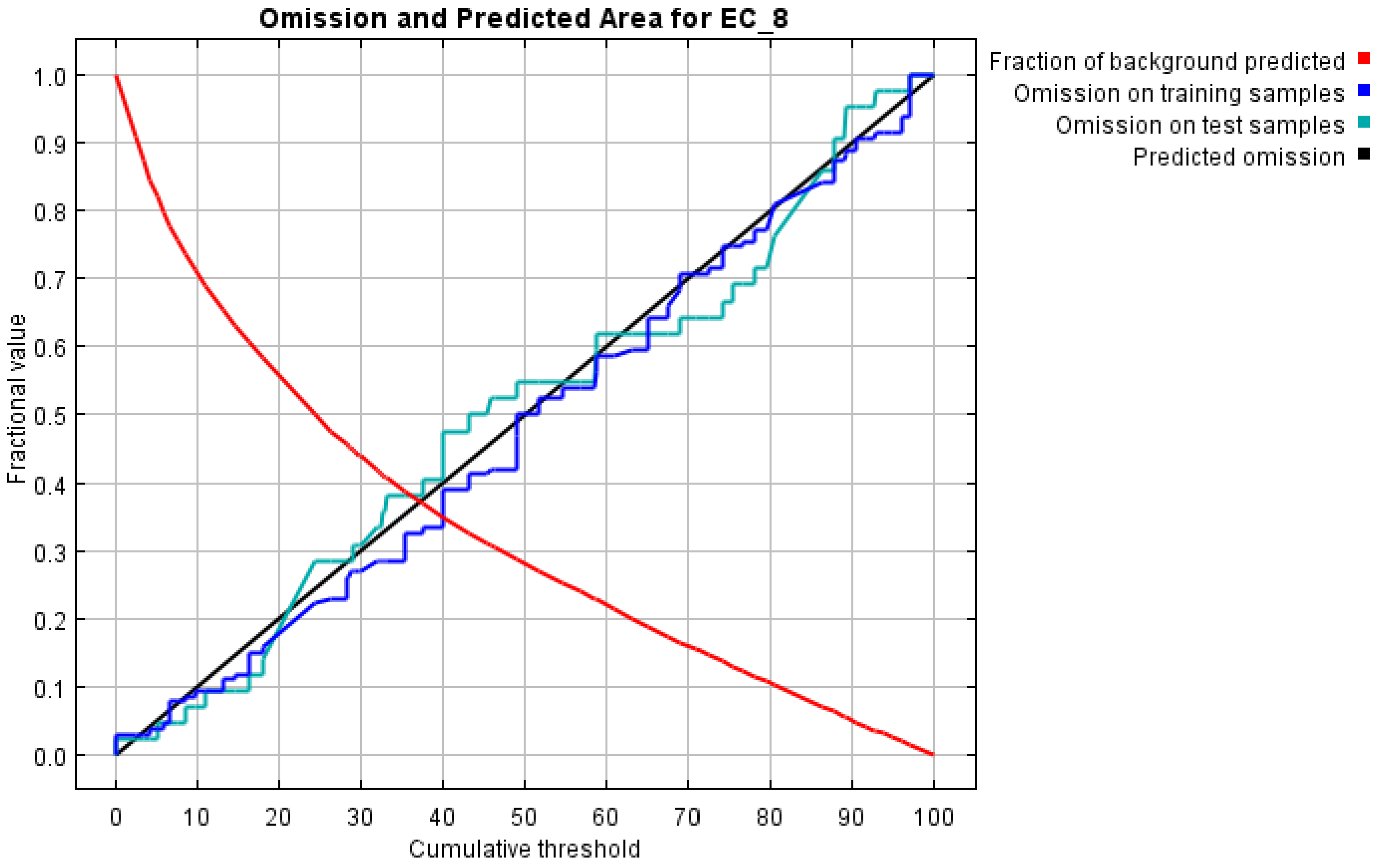

The next picture is the receiver operating characteristic (ROC) curve for the same data. Note that the specificity is defined using predicted area, rather than true commission (see the paper by Phillips, Anderson and Schapire cited on the help page for discussion of what this means). This implies that the maximum achievable AUC is less than 1. If test data is drawn from the Maxent distribution itself, then the maximum possible test AUC would be 0.666 rather than 1; in practice the test AUC may exceed this bound.

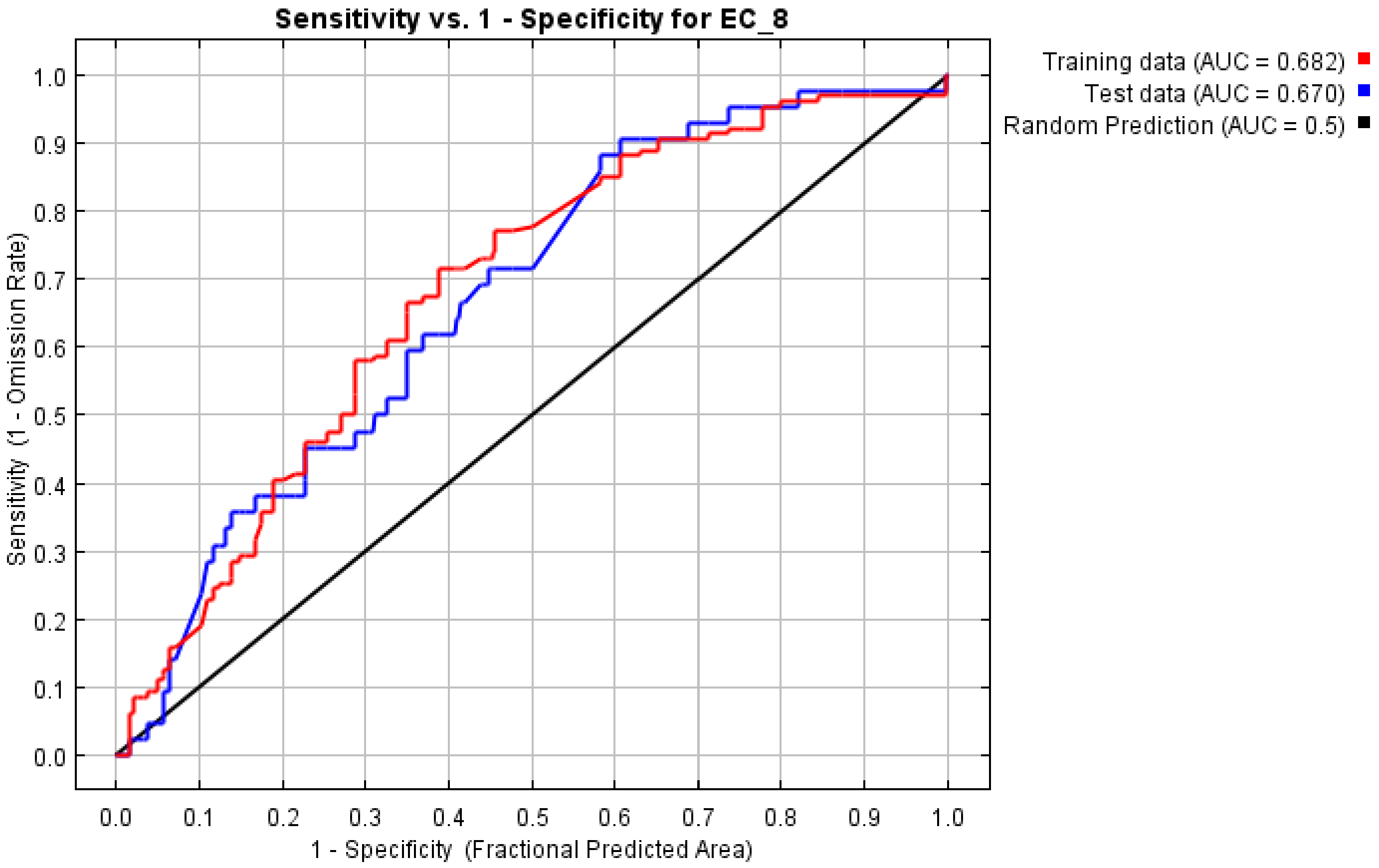

Some common thresholds and corresponding omission rates are as follows. If test data are available, binomial probabilities are calculated exactly if the number of test samples is at most 25, otherwise using a normal approximation to the binomial. These are 1-sided p-values for the null hypothesis that test points are predicted no better than by a random prediction with the same fractional predicted area. The "Balance" threshold minimizes 6 * training omission rate + .04 * cumulative threshold + 1.6 * fractional predicted area.

Cumulative threshold	Logistic threshold	Description	Fractional predicted area	Training omission rate	Test omission rate	P-value
1.000	0.190	Fixed cumulative value 1	0.908	0.032	0.024	6.295E-2
5.000	0.218	Fixed cumulative value 5	0.820	0.040	0.024	4.238E-3
10.000	0.316	Fixed cumulative value 10	0.688	0.095	0.071	3.78E-4
0.057	0.176	Minimum training presence	0.998	0.000	0.000	3.753E-1
13.076	0.334	10 percentile training presence	0.654	0.095	0.095	3.134E-4
40.048	0.535	Equal training sensitivity and specificity	0.349	0.349	0.476	8.805E-3
35.303	0.476	Maximum training sensitivity plus specificity	0.388	0.286	0.381	1.05E-3

35.303	0.476	Equal test sensitivity and specificity	0.388	0.286	0.381	1.05E-3
16.262	0.372	Maximum test sensitivity plus specificity	0.607	0.119	0.095	3.855E-5
0.057	0.176	Balance training omission, predicted area and threshold value	0.998	0.000	0.000	3.753E-1
5.001	0.218	Equate entropy of thresholded and original distributions	0.820	0.040	0.024	4.238E-3

Click here to interactively explore this prediction using the Explain tool. If clicking from your browser does not succeed in starting the tool, try running the script in C:\Users\Bill\Desktop\Maxent Outputs EC 7-8-15\EC_8_explain.bat directly. This tool requires the environmental grids to be small enough that they all fit in memory.

Response curves

These curves show how each environmental variable affects the Maxent prediction. The curves show how the logistic prediction changes as each environmental variable is varied, keeping all other environmental variables at their average sample value. Click on a response curve to see a larger version. Note that the curves can be hard to interpret if you have strongly correlated variables, as the model may depend on the correlations in ways that are not evident in the curves. In other words, the curves show the marginal effect of changing exactly one variable, whereas the model may take advantage of sets of variables changing together.

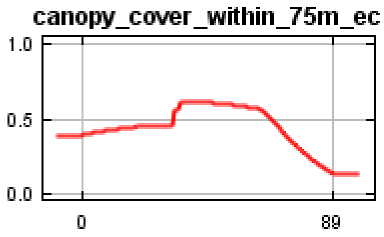

In contrast to the above marginal response curves, each of the following curves represents a different model, namely, a Maxent model created using only the corresponding variable. These plots reflect the dependence of predicted suitability both on the selected variable and on dependencies induced by correlations between the selected variable and other variables. They may be easier to interpret if there are strong correlations between variables.

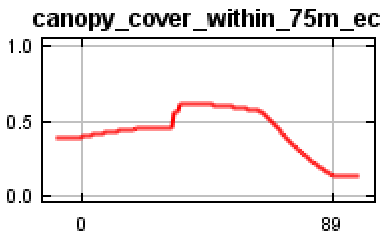

Analysis of variable contributions

The following table gives estimates of relative contributions of the environmental variables to the Maxent model. To determine the first estimate, in each iteration of the training algorithm, the increase in regularized gain is added to the contribution of the corresponding variable, or subtracted from it if the change to the absolute value of lambda is negative. For the second estimate, for each environmental variable in turn, the values of that variable on training presence and background data are randomly permuted. The model is reevaluated on the permuted data, and the resulting drop in training AUC is shown in the table, normalized to percentages. As with the variable jackknife, variable contributions should be interpreted with caution when the predictor variables are correlated.

Variable	Percent contribution	Permutation importance
canopy_cover_within_75m_ec	100	100

Raw data outputs and control parameters

The data used in the above analysis is contained in the next links. Please see the Help button for more information on these.
The model applied to the training environmental layers
The coefficients of the model
The omission and predicted area for varying cumulative and raw thresholds
The prediction strength at the training and (optionally) test presence sites
Results for all species modeled in the same Maxent run, with summary statistics and (optionally) jackknife results

Regularized training gain is 0.178, training AUC is 0.682, unregularized training gain is 0.213.
Unregularized test gain is 0.180.
Test AUC is 0.670, standard deviation is 0.037 (calculated as in DeLong, DeLong & Clarke-Pearson 1988, equation 2).
Algorithm converged after 300 iterations (1 seconds).

The follow settings were used during the run:
126 presence records used for training, 42 for testing.
10000 points used to determine the Maxent distribution (background points and presence points).
Environmental layers used (all continuous): canopy_cover_within_75m_ec
Regularization values: linear/quadratic/product: 0.050, categorical: 0.250, threshold: 1.000, hinge: 0.500
Feature types used: hinge linear threshold quadratic
responsecurves: true
pictures: false
outputdirectory: C:\Users\Bill\Desktop\Maxent Outputs EC 7-8-15
samplesfile: C:\Users\Bill\Desktop\Maxent_samples\Compare_NEC_EC_150m_311_WGS_84_m.csv
environmentallayers: C:\Users\Bill\Desktop\Maxent Layers\maxent.cache
randomseed: true
askoverwrite: false
randomtestpoints: 25
replicates: 10
replicatetype: subsample
Command line used:

Maxent model for EC_9

This page contains some analysis of the Maxent model for EC_9, created Wed Jul 08 11:42:52 EDT 2015 using Maxent version 3.3.3k. If you would like to do further analyses, the raw data used here is linked to at the end of this page.

Analysis of omission/commission

The following picture shows the omission rate and predicted area as a function of the cumulative threshold. The omission rate is is calculated both on the training presence records, and (if test data are used) on the test records. The omission rate should be close to the predicted omission, because of the definition of the cumulative threshold.

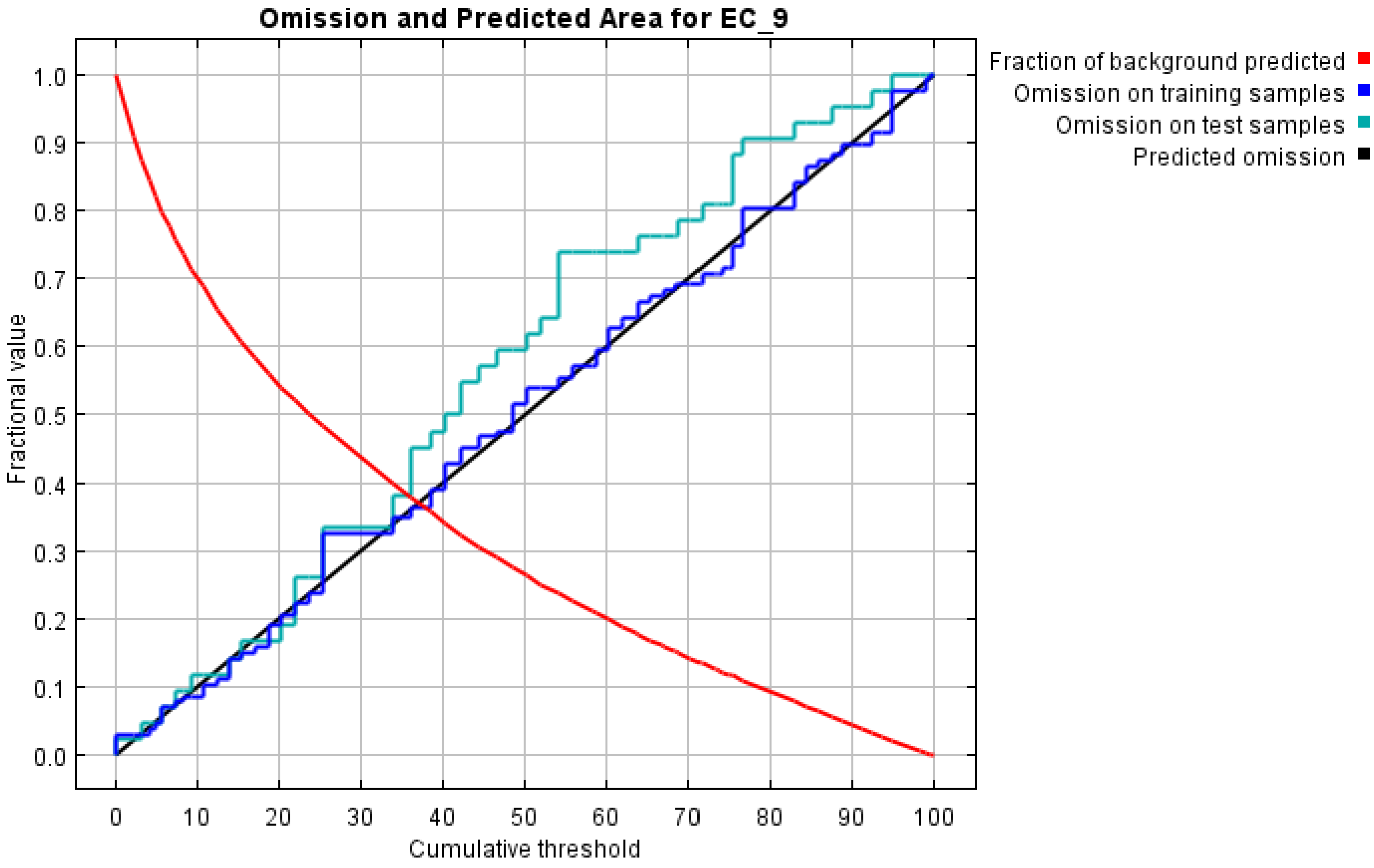

The next picture is the receiver operating characteristic (ROC) curve for the same data. Note that the specificity is defined using predicted area, rather than true commission (see the paper by Phillips, Anderson and Schapire cited on the help page for discussion of what this means). This implies that the maximum achievable AUC is less than 1. If test data is drawn from the Maxent distribution itself, then the maximum possible test AUC would be 0.676 rather than 1; in practice the test AUC may exceed this bound.

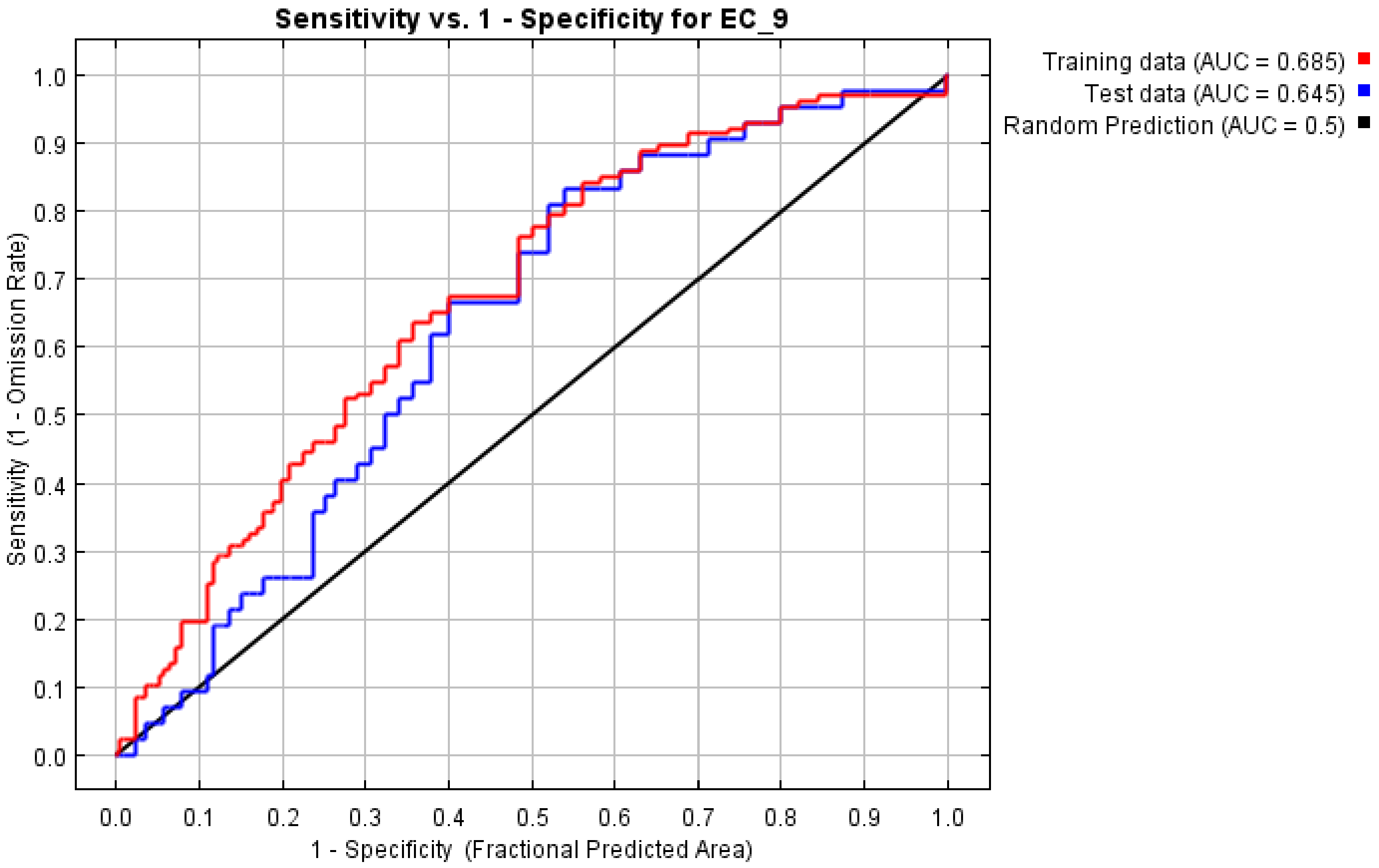

Some common thresholds and corresponding omission rates are as follows. If test data are available, binomial probabilities are calculated exactly if the number of test samples is at most 25, otherwise using a normal approximation to the binomial. These are 1-sided p-values for the null hypothesis that test points are predicted no better than by a random prediction with the same fractional predicted area. The "Balance" threshold minimizes 6 * training omission rate + .04 * cumulative threshold + 1.6 * fractional predicted area.

Cumulative threshold	Logistic threshold	Description	Fractional predicted area	Training omission rate	Test omission rate	P-value
1.000	0.184	Fixed cumulative value 1	0.908	0.032	0.024	6.295E-2
5.000	0.238	Fixed cumulative value 5	0.798	0.048	0.048	6.435E-3
10.000	0.313	Fixed cumulative value 10	0.688	0.087	0.119	3.441E-3
0.056	0.171	Minimum training presence	0.998	0.000	0.000	3.753E-1
10.642	0.313	10 percentile training presence	0.688	0.087	0.119	3.441E-3
38.417	0.485	Equal training sensitivity and specificity	0.357	0.365	0.452	4.92E-3
18.738	0.393	Maximum training sensitivity plus	0.560	0.159	0.167	1.837E-

		specificity				4
35.979	0.470	Equal test sensitivity and specificity	0.379	0.349	0.381	6.855E-4
20.299	0.409	Maximum test sensitivity plus specificity	0.541	0.190	0.167	7.073E-5
0.056	0.171	Balance training omission, predicted area and threshold value	0.998	0.000	0.000	3.753E-1
4.906	0.224	Equate entropy of thresholded and original distributions	0.820	0.040	0.048	1.285E-2

Click here to interactively explore this prediction using the Explain tool. If clicking from your browser does not succeed in starting the tool, try running the script in C:\Users\Bill\Desktop\Maxent Outputs EC 7-8-15\EC_9_explain.bat directly. This tool requires the environmental grids to be small enough that they all fit in memory.

Response curves

These curves show how each environmental variable affects the Maxent prediction. The curves show how the logistic prediction changes as each environmental variable is varied, keeping all other environmental variables at their average sample value. Click on a response curve to see a larger version. Note that the curves can be hard to interpret if you have strongly correlated variables, as the model may depend on the correlations in ways that are not evident in the curves. In other words, the curves show the marginal effect of changing exactly one variable, whereas the model may take advantage of sets of variables changing together.

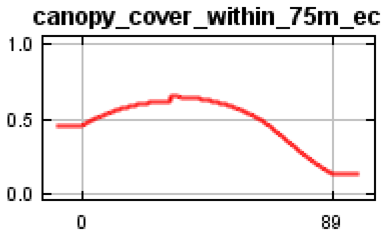

In contrast to the above marginal response curves, each of the following curves represents a different model, namely, a Maxent model created using only the corresponding variable. These plots reflect the dependence of predicted suitability both on the selected variable and on dependencies induced by correlations between the selected variable and other variables. They may be easier to interpret if there are strong correlations between variables.

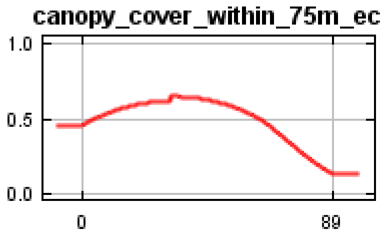

Analysis of variable contributions

The following table gives estimates of relative contributions of the environmental variables to the Maxent model. To determine the first estimate, in each iteration of the training algorithm, the increase in regularized gain is added to the contribution of the corresponding variable, or subtracted from it if the change to the absolute value of lambda is negative. For the second estimate, for each environmental variable in turn, the values of that variable on training presence and background data are randomly permuted. The model is reevaluated on the permuted data, and the resulting drop in training AUC is shown in the table, normalized to percentages. As with the variable jackknife, variable contributions should be interpreted with caution when the predictor variables are correlated.

Variable	Percent contribution	Permutation importance
canopy_cover_within_75m_ec	100	100

Raw data outputs and control parameters

The data used in the above analysis is contained in the next links. Please see the Help button for more information on these.
The model applied to the training environmental layers
The coefficients of the model
The omission and predicted area for varying cumulative and raw thresholds
The prediction strength at the training and (optionally) test presence sites
Results for all species modeled in the same Maxent run, with summary statistics and (optionally) jackknife results

Regularized training gain is 0.196, training AUC is 0.685, unregularized training gain is 0.214.
Unregularized test gain is 0.130.
Test AUC is 0.645, standard deviation is 0.035 (calculated as in DeLong, DeLong & Clarke-Pearson 1988, equation 2).
Algorithm converged after 360 iterations (1 seconds).

The follow settings were used during the run:
126 presence records used for training, 42 for testing.
10000 points used to determine the Maxent distribution (background points and presence points).
Environmental layers used (all continuous): canopy_cover_within_75m_ec
Regularization values: linear/quadratic/product: 0.050, categorical: 0.250, threshold: 1.000, hinge: 0.500
Feature types used: hinge linear threshold quadratic
responsecurves: true
pictures: false
outputdirectory: C:\Users\Bill\Desktop\Maxent Outputs EC 7-8-15
samplesfile: C:\Users\Bill\Desktop\Maxent_samples\Compare_NEC_EC_150m_311_WGS_84_m.csv
environmentallayers: C:\Users\Bill\Desktop\Maxent Layers\maxent.cache
randomseed: true
askoverwrite: false
randomtestpoints: 25
replicates: 10
replicatetype: subsample
