# Supplementary material for: An Analysis of Overstory Tree Canopy Cover in Sites Occupied by Native and Introduced Cottontails in the Northeastern United States with Recommendations for Habitat Management for New England Cottontail
Source: PLoS One. 2015 Aug 12;10(8):e0135067. doi: 10.1371/journal.pone.0135067 (PMC4534376; doi:10.1371/journal.pone.0135067)
Supplement: S3 Model Outputs — (PDF) [file pone.0135067.s004.pdf]

Replicated maxent model for NEC

This page summarizes the results of 10 split-sample models for NEC, created Sun Jul 12 11:09:20 EDT 2015 using Maxent version 3.3.3k. The individual models are here: [0] [1] [2] [3] [4] [5] [6] [7] [8] [9]

Analysis of omission/commission

The following picture shows the test omission rate and predicted area as a function of the cumulative threshold, averaged over the replicate runs. The omission rate should be close to the predicted omission, because of the definition of the cumulative threshold.

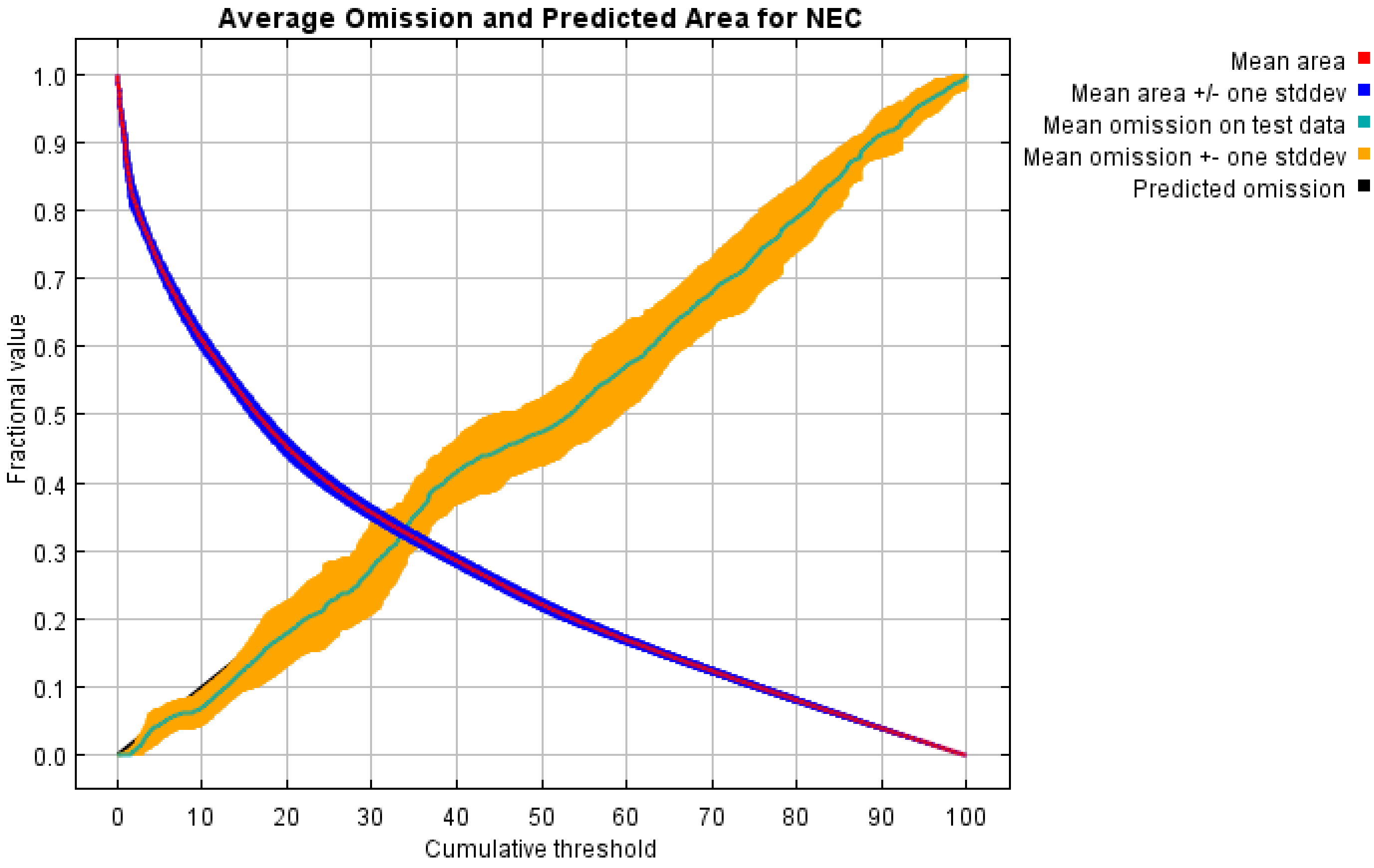

The next picture is the receiver operating characteristic (ROC) curve for the same data, again averaged over the replicate runs. Note that the specificity is defined using predicted area, rather than true commission (see the paper by Phillips, Anderson and Schapire cited on the help page for discussion of what this means). The average test AUC for the replicate runs is 0.733, and the standard deviation is 0.019.

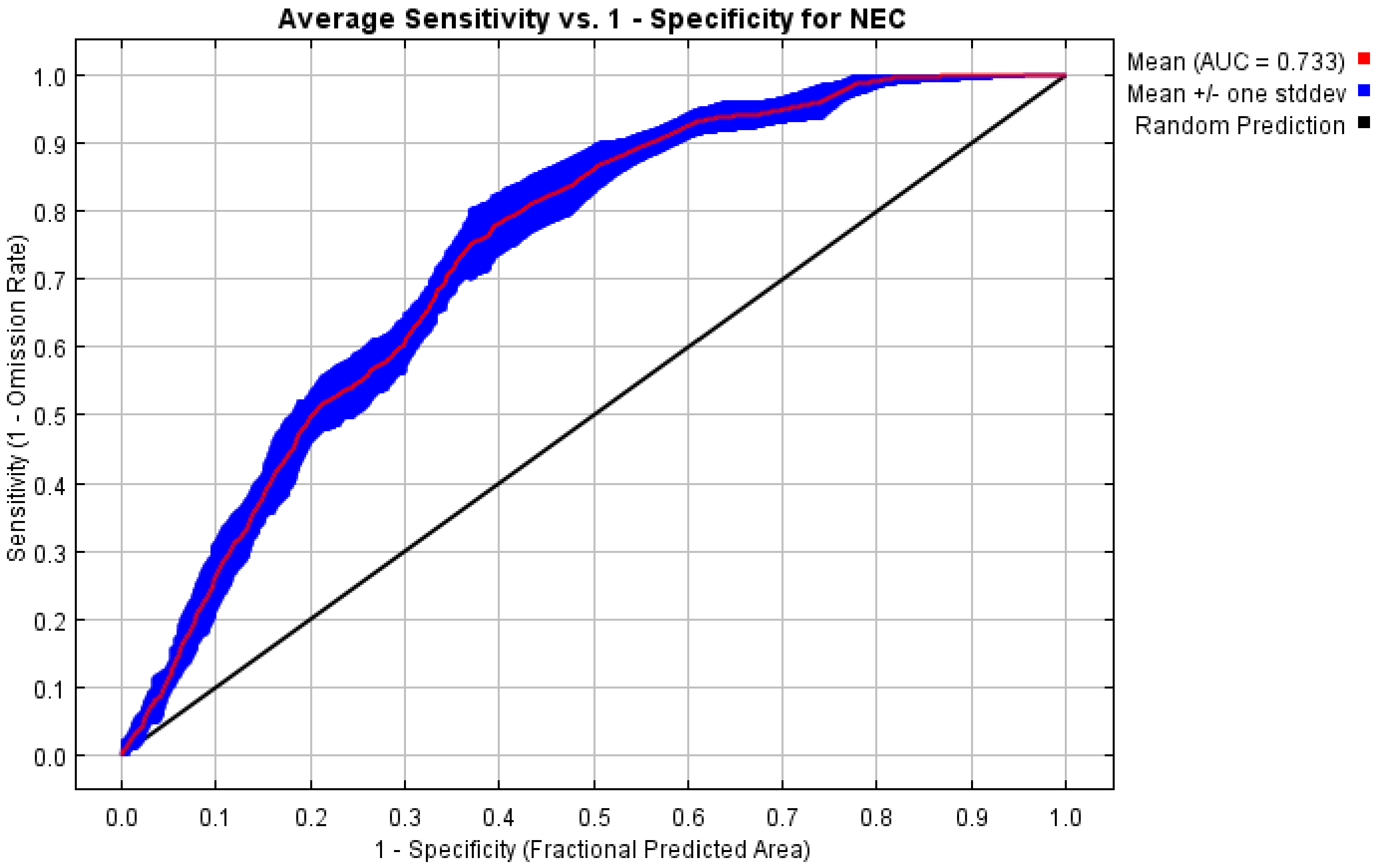

Response curves

These curves show how each environmental variable affects the Maxent prediction. The curves show how the logistic prediction changes as each environmental variable is varied, keeping all other environmental variables at their average sample value. Click on a response curve to see a larger version. Note that the curves can be hard to interpret if you have strongly correlated variables, as the model may depend on the correlations in ways that are not evident in the curves. In other words, the curves show the marginal effect of changing exactly one variable, whereas the model may take advantage of sets of variables changing together. The curves show the mean response of the 10 replicate Maxent runs (red) and and the mean +/- one standard deviation (blue, two shades for categorical variables).

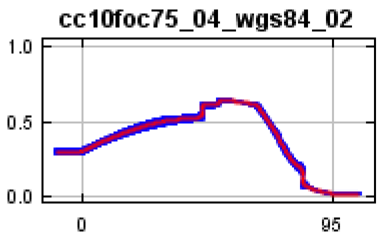

In contrast to the above marginal response curves, each of the following curves represents a different model, namely, a Maxent model created using only the corresponding variable. These plots reflect the dependence of predicted suitability both on the selected variable and on dependencies induced by correlations between the selected variable and other variables. They may be easier to interpret if there are strong correlations between variables.

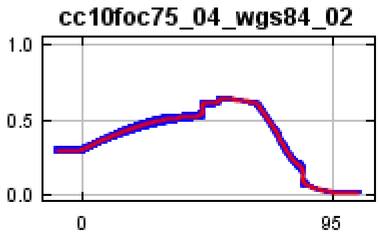

Analysis of variable contributions

The following table gives estimates of relative contributions of the environmental variables to the Maxent model. To determine the first estimate, in each iteration of the training algorithm, the increase in regularized gain is added to the contribution of the corresponding variable, or subtracted from it if the change to the absolute value of lambda is negative. For the second estimate, for each environmental variable in turn, the values of that variable on training presence and background data are randomly permuted. The model is reevaluated on the permuted data, and the resulting drop in training AUC is shown in the table, normalized to percentages. As with the variable jackknife, variable contributions should be interpreted with caution when the predictor variables are correlated. Values shown are averages over replicate runs.

Variable	Percent contribution	Permutation importance
cc10foc75_04_wgs84_02	100	100

Command line to repeat this species model: java density.MaxEnt nowarnings noprefixes -E "" -E NEC responsecurves nopictures "outputdirectory=C:\Users\Bill\Desktop\Maxent Results NEC samples entire area" samplesfile=C:\Users\Bill\Desktop\Maxent_samples\Compare_NEC_EC_150m_311_WGS_84_m.csv "environmentallayers=C:\Users\Bill\Desktop\Maxent Layers\maxent.cache" randomseed randomtestpoints=25 replicates=10 replicatetype=subsample -N canopy_cover_within_75m_ec -N canopy_cover_within_75m_nec -N cc10foc75_02_buff3km_nec_01_wgs84_01 -N cc10foc75_04_buff3km_nec_20_wgs84_20 -N cc10mfoc75_04_buff3km_ec_20_wgs84_20_01 -N cc_10foc75_d_buff3km_nec_01_wgs84_10 -N cc_10mfoc75_d_buff3km_ec_01_wgs84_10 -N vpd_b_wgs_buff10_ec_01 -N vpd_b_wgs_buff50_ec_01 -N vpd_b_wgs_ec_01 -N vpd_b_wgs_nec_01

Maxent model for NEC_1

This page contains some analysis of the Maxent model for NEC_1, created Sat Jul 11 22:20:22 EDT 2015 using Maxent version 3.3.3k. If you would like to do further analyses, the raw data used here is linked to at the end of this page.

Analysis of omission/commission

The following picture shows the omission rate and predicted area as a function of the cumulative threshold. The omission rate is is calculated both on the training presence records, and (if test data are used) on the test records. The omission rate should be close to the predicted omission, because of the definition of the cumulative threshold.

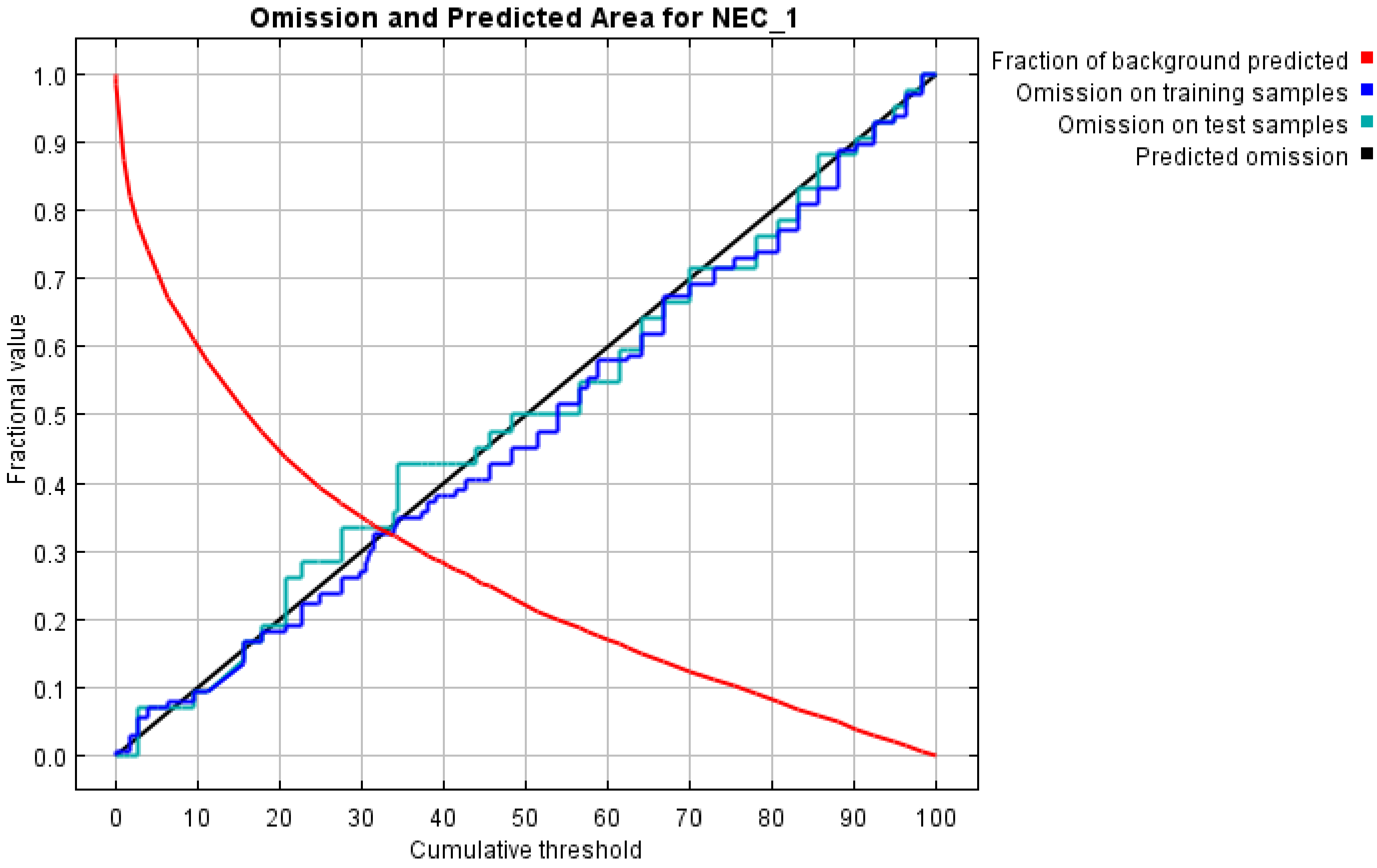

The next picture is the receiver operating characteristic (ROC) curve for the same data. Note that the specificity is defined using predicted area, rather than true commission (see the paper by Phillips, Anderson and Schapire cited on the help page for discussion of what this means). This implies that the maximum achievable AUC is less than 1. If test data is drawn from the Maxent distribution itself, then the maximum possible test AUC would be 0.726 rather than 1; in practice the test AUC may exceed this bound.

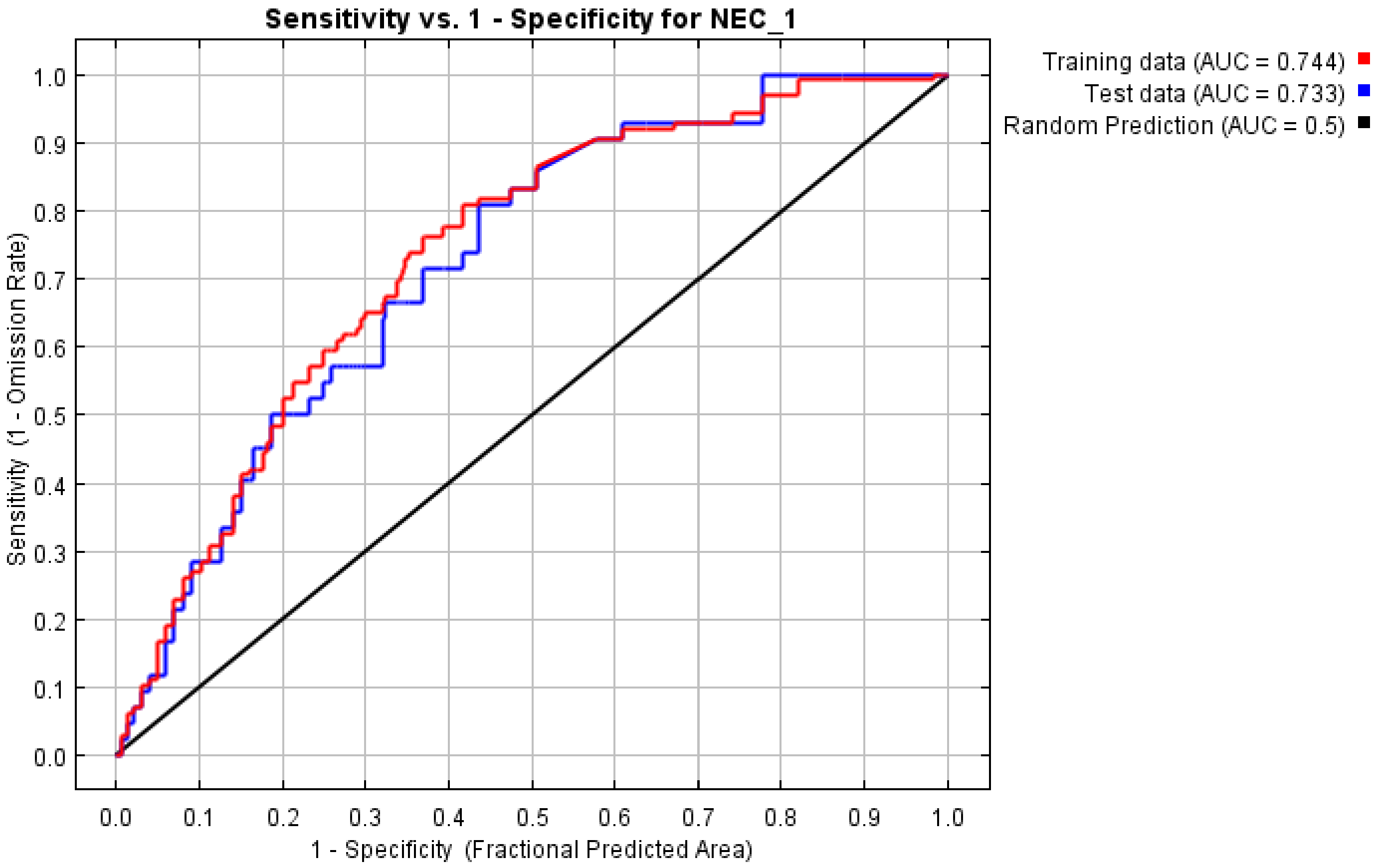

Some common thresholds and corresponding omission rates are as follows. If test data are available, binomial probabilities are calculated exactly if the number of test samples is at most 25, otherwise using a normal approximation to the binomial. These are 1-sided p-values for the null hypothesis that test points are predicted no better than by a random prediction with the same fractional predicted area. The "Balance" threshold minimizes 6 * training omission rate + .04 * cumulative threshold + 1.6 * fractional predicted area.

**Cumulative threshold**	**Logistic threshold**	**Description**	**Fractional predicted area**	**Training omission rate**	**Test omission rate**	**P-value**
1.000	0.065	Fixed cumulative value 1	0.874	0.008	0.000	6.999E-3
5.000	0.221	Fixed cumulative value 5	0.706	0.071	0.071	7.609E-4
10.000	0.302	Fixed cumulative value 10	0.578	0.095	0.095	9.118E-6
0.101	0.057	Minimum training presence	0.985	0.000	0.000	2.119E-1
11.309	0.302	10 percentile training presence	0.578	0.095	0.095	9.118E-6
33.859	0.511	Equal training sensitivity and specificity	0.323	0.325	0.333	9.628E-7
22.575	0.414	Maximum training sensitivity plus	0.416	0.190	0.262	1.15E-5

		specificity				
31.546	0.508	Equal test sensitivity and specificity	0.339	0.302	0.333	3.629E-6
20.779	0.383	Maximum test sensitivity plus specificity	0.437	0.183	0.190	5.496E-7
1.590	0.159	Balance training omission, predicted area and threshold value	0.820	0.008	0.000	1.189E-3
5.075	0.221	Equate entropy of thresholded and original distributions	0.706	0.071	0.071	7.609E-4

Click here to interactively explore this prediction using the Explain tool. If clicking from your browser does not succeed in starting the tool, try running the script in C:\Users\Bill\Desktop\Maxent Results NEC samples entire area\NEC_1_explain.bat directly. This tool requires the environmental grids to be small enough that they all fit in memory.

Response curves

These curves show how each environmental variable affects the Maxent prediction. The curves show how the logistic prediction changes as each environmental variable is varied, keeping all other environmental variables at their average sample value. Click on a response curve to see a larger version. Note that the curves can be hard to interpret if you have strongly correlated variables, as the model may depend on the correlations in ways that are not evident in the curves. In other words, the curves show the marginal effect of changing exactly one variable, whereas the model may take advantage of sets of variables changing together.

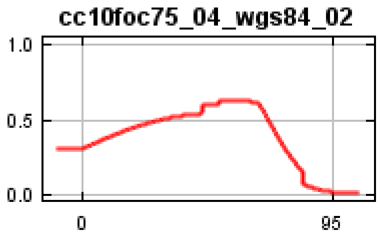

In contrast to the above marginal response curves, each of the following curves represents a different model, namely, a Maxent model created using only the corresponding variable. These plots reflect the dependence of predicted suitability both on the selected variable and on dependencies induced by correlations between the selected variable and other variables. They may be easier to interpret if there are strong correlations between variables.

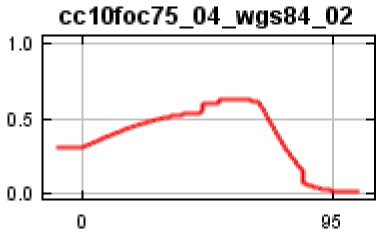

Analysis of variable contributions

The following table gives estimates of relative contributions of the environmental variables to the Maxent model. To determine the first estimate, in each iteration of the training algorithm, the increase in regularized gain is added to the contribution of the corresponding variable, or subtracted from it if the change to the absolute value of lambda is negative. For the second estimate, for each environmental variable in turn, the values of that variable on training presence and background data are randomly permuted. The model is reevaluated on the permuted data, and the resulting drop in training AUC is shown in the table, normalized to percentages. As with the variable jackknife, variable contributions should be interpreted with caution when the predictor variables are correlated.

Variable	Percent contribution	Permutation importance
cc10foc75_04_wgs84_02	100	100

Raw data outputs and control parameters

The data used in the above analysis is contained in the next links. Please see the Help button for more information on these.
The model applied to the training environmental layers
The coefficients of the model
The omission and predicted area for varying cumulative and raw thresholds
The prediction strength at the training and (optionally) test presence sites
Results for all species modeled in the same Maxent run, with summary statistics and (optionally) jackknife results

Regularized training gain is 0.343, training AUC is 0.744, unregularized training gain is 0.394.
Unregularized test gain is 0.361.
Test AUC is 0.733, standard deviation is 0.033 (calculated as in DeLong, DeLong & Clarke-Pearson 1988, equation 2).
Algorithm terminated after 500 iterations (1 seconds).

The follow settings were used during the run:
126 presence records used for training, 42 for testing.
10000 points used to determine the Maxent distribution (background points and presence points).
Environmental layers used (all continuous): cc10foc75_04_wgs84_02
Regularization values: linear/quadratic/product: 0.050, categorical: 0.250, threshold: 1.000, hinge: 0.500
Feature types used: hinge linear threshold quadratic
responsecurves: true
pictures: false
outputdirectory: C:\Users\Bill\Desktop\Maxent Results NEC samples entire area
samplesfile: C:\Users\Bill\Desktop\Maxent_samples\Compare_NEC_EC_150m_311_WGS_84_m.csv
environmentallayers: C:\Users\Bill\Desktop\Maxent Layers\maxent.cache
randomseed: true
randomtestpoints: 25
replicates: 10
replicatetype: subsample

Maxent model for NEC_2

This page contains some analysis of the Maxent model for NEC_2, created Sat Jul 11 23:51:45 EDT 2015 using Maxent version 3.3.3k. If you would like to do further analyses, the raw data used here is linked to at the end of this page.

Analysis of omission/commission

The following picture shows the omission rate and predicted area as a function of the cumulative threshold. The omission rate is is calculated both on the training presence records, and (if test data are used) on the test records. The omission rate should be close to the predicted omission, because of the definition of the cumulative threshold.

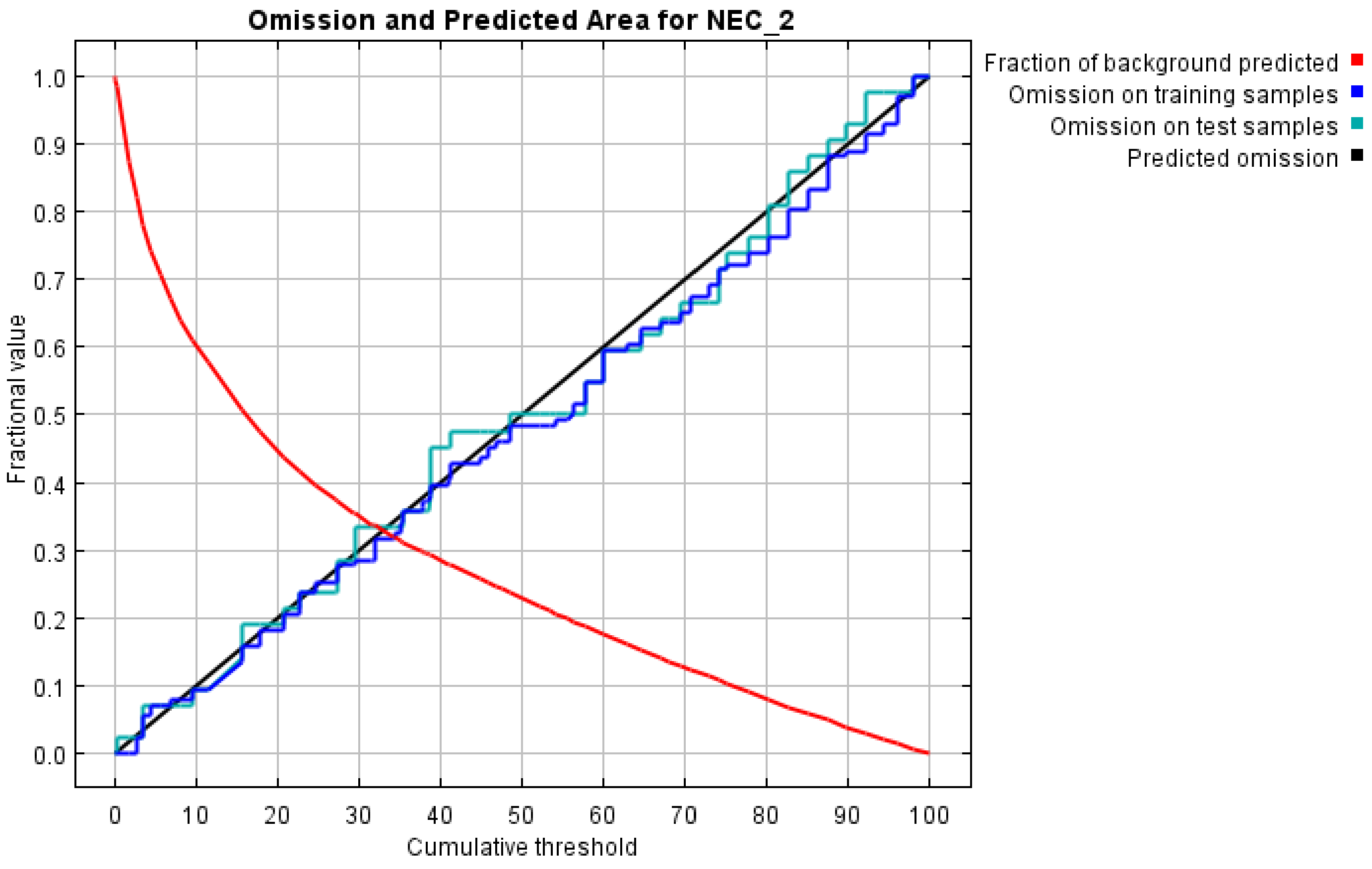

The next picture is the receiver operating characteristic (ROC) curve for the same data. Note that the specificity is defined using predicted area, rather than true commission (see the paper by Phillips, Anderson and Schapire cited on the help page for discussion of what this means). This implies that the maximum achievable AUC is less than 1. If test data is drawn from the Maxent distribution itself, then the maximum possible test AUC would be 0.723 rather than 1; in practice the test AUC may exceed this bound.

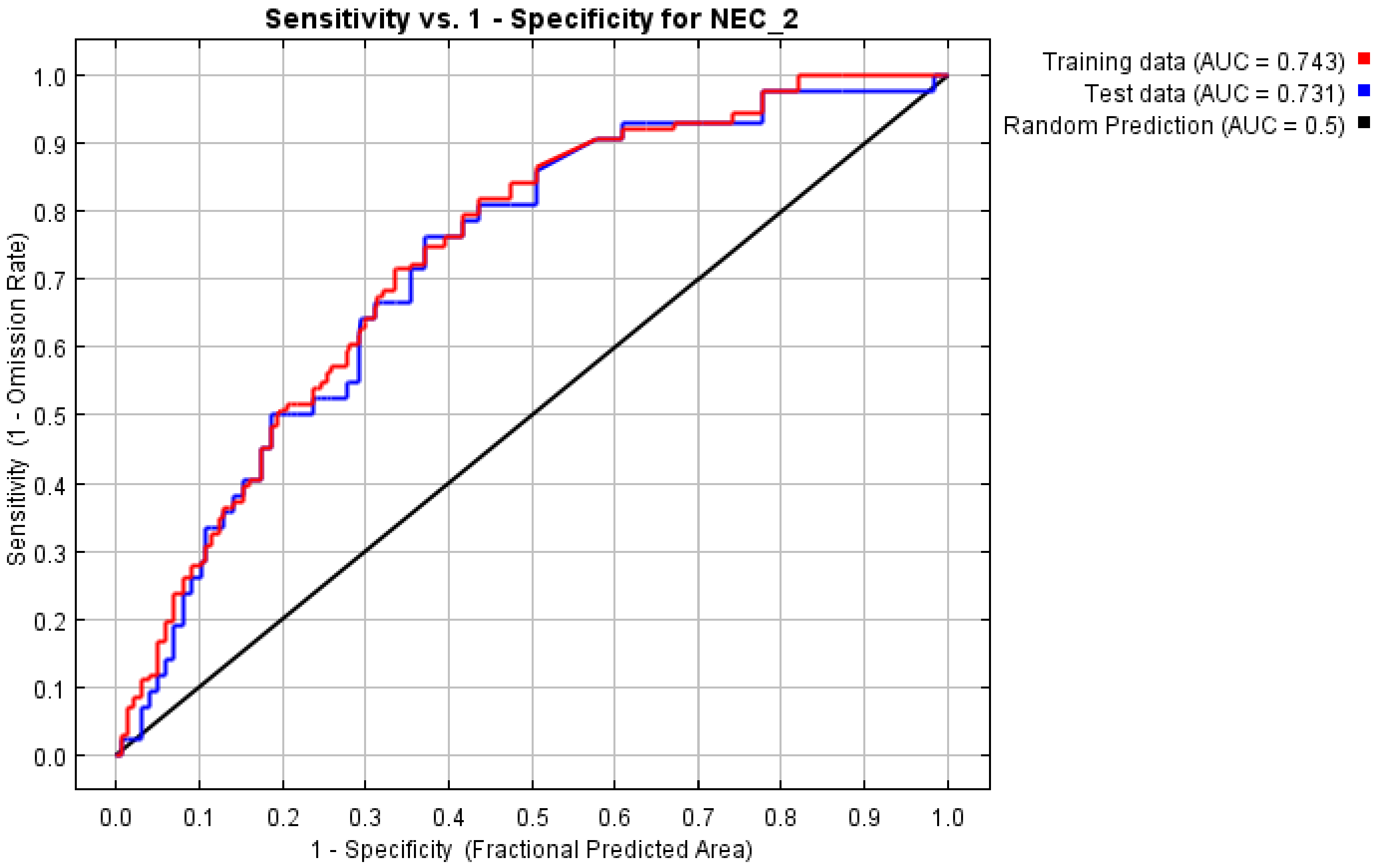

Some common thresholds and corresponding omission rates are as follows. If test data are available, binomial probabilities are calculated exactly if the number of test samples is at most 25, otherwise using a normal approximation to the binomial. These are 1-sided p-values for the null hypothesis that test points are predicted no better than by a random prediction with the same fractional predicted area. The "Balance" threshold minimizes 6 * training omission rate + .04 * cumulative threshold + 1.6 * fractional predicted area.

Cumulative threshold	Logistic threshold	Description	Fractional predicted area	Training omission rate	Test omission rate	P-value
1.000	0.110	Fixed cumulative value 1	0.874	0.000	0.024	2.319E-2
5.000	0.205	Fixed cumulative value 5	0.706	0.071	0.071	7.609E-4
10.000	0.302	Fixed cumulative value 10	0.578	0.095	0.095	9.118E-6
2.568	0.134	Minimum training presence	0.820	0.000	0.024	4.183E-3
11.343	0.302	10 percentile training presence	0.578	0.095	0.095	9.118E-6
34.322	0.523	Equal training sensitivity and specificity	0.320	0.317	0.333	7.25E-7
20.730	0.392	Maximum training sensitivity plus	0.437	0.183	0.190	5.496E-

		specificity				7
31.869	0.514	Equal test sensitivity and specificity	0.337	0.286	0.333	2.996E-6
27.291	0.468	Maximum test sensitivity plus specificity	0.372	0.254	0.238	8.645E-8
2.568	0.134	Balance training omission, predicted area and threshold value	0.820	0.000	0.024	4.183E-3
5.525	0.205	Equate entropy of thresholded and original distributions	0.706	0.071	0.071	7.609E-4

Click here to interactively explore this prediction using the Explain tool. If clicking from your browser does not succeed in starting the tool, try running the script in C:\Users\Bill\Desktop\Maxent Results NEC samples entire area\NEC_2_explain.bat directly. This tool requires the environmental grids to be small enough that they all fit in memory.

Response curves

These curves show how each environmental variable affects the Maxent prediction. The curves show how the logistic prediction changes as each environmental variable is varied, keeping all other environmental variables at their average sample value. Click on a response curve to see a larger version. Note that the curves can be hard to interpret if you have strongly correlated variables, as the model may depend on the correlations in ways that are not evident in the curves. In other words, the curves show the marginal effect of changing exactly one variable, whereas the model may take advantage of sets of variables changing together.

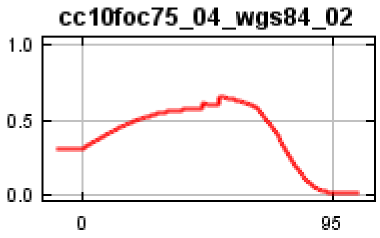

In contrast to the above marginal response curves, each of the following curves represents a different model, namely, a Maxent model created using only the corresponding variable. These plots reflect the dependence of predicted suitability both on the selected variable and on dependencies induced by correlations between the selected variable and other variables. They may be easier to interpret if there are strong correlations between variables.

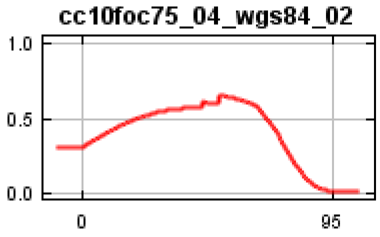

Analysis of variable contributions

The following table gives estimates of relative contributions of the environmental variables to the Maxent model. To determine the first estimate, in each iteration of the training algorithm, the increase in regularized gain is added to the contribution of the corresponding variable, or subtracted from it if the change to the absolute value of lambda is negative. For the second estimate, for each environmental variable in turn, the values of that variable on training presence and background data are randomly permuted. The model is reevaluated on the permuted data, and the resulting drop in training AUC is shown in the table, normalized to percentages. As with the variable jackknife, variable contributions should be interpreted with caution when the predictor variables are correlated.

Variable	Percent contribution	Permutation importance
cc10foc75_04_wgs84_02	100	100

Raw data outputs and control parameters

The data used in the above analysis is contained in the next links. Please see the Help button for more information on these.
The model applied to the training environmental layers
The coefficients of the model
The omission and predicted area for varying cumulative and raw thresholds
The prediction strength at the training and (optionally) test presence sites
Results for all species modeled in the same Maxent run, with summary statistics and (optionally) jackknife results

Regularized training gain is 0.331, training AUC is 0.743, unregularized training gain is 0.378.
Unregularized test gain is 0.349.
Test AUC is 0.731, standard deviation is 0.034 (calculated as in DeLong, DeLong & Clarke-Pearson 1988, equation 2).
Algorithm converged after 460 iterations (1 seconds).

The follow settings were used during the run:
126 presence records used for training, 42 for testing.
10000 points used to determine the Maxent distribution (background points and presence points).
Environmental layers used (all continuous): cc10foc75_04_wgs84_02
Regularization values: linear/quadratic/product: 0.050, categorical: 0.250, threshold: 1.000, hinge: 0.500
Feature types used: hinge linear threshold quadratic
responsecurves: true
pictures: false
outputdirectory: C:\Users\Bill\Desktop\Maxent Results NEC samples entire area
samplesfile: C:\Users\Bill\Desktop\Maxent_samples\Compare_NEC_EC_150m_311_WGS_84_m.csv
environmentallayers: C:\Users\Bill\Desktop\Maxent Layers\maxent.cache
randomseed: true
randomtestpoints: 25
replicates: 10
replicatetype: subsample

Maxent model for NEC_3

This page contains some analysis of the Maxent model for NEC_3, created Sun Jul 12 01:48:49 EDT 2015 using Maxent version 3.3.3k. If you would like to do further analyses, the raw data used here is linked to at the end of this page.

Analysis of omission/commission

The following picture shows the omission rate and predicted area as a function of the cumulative threshold. The omission rate is is calculated both on the training presence records, and (if test data are used) on the test records. The omission rate should be close to the predicted omission, because of the definition of the cumulative threshold.

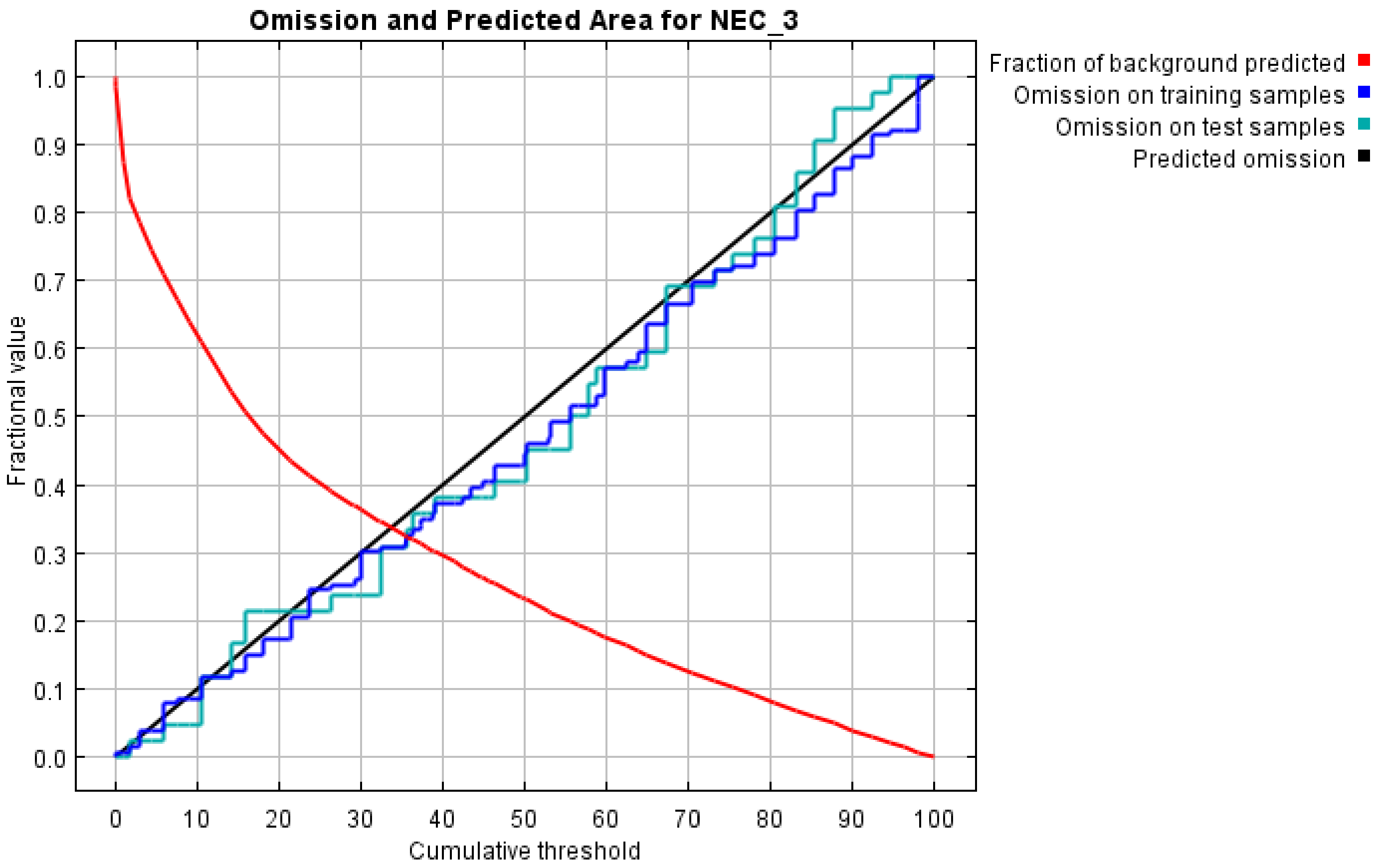

The next picture is the receiver operating characteristic (ROC) curve for the same data. Note that the specificity is defined using predicted area, rather than true commission (see the paper by Phillips, Anderson and Schapire cited on the help page for discussion of what this means). This implies that the maximum achievable AUC is less than 1. If test data is drawn from the Maxent distribution itself, then the maximum possible test AUC would be 0.719 rather than 1; in practice the test AUC may exceed this bound.

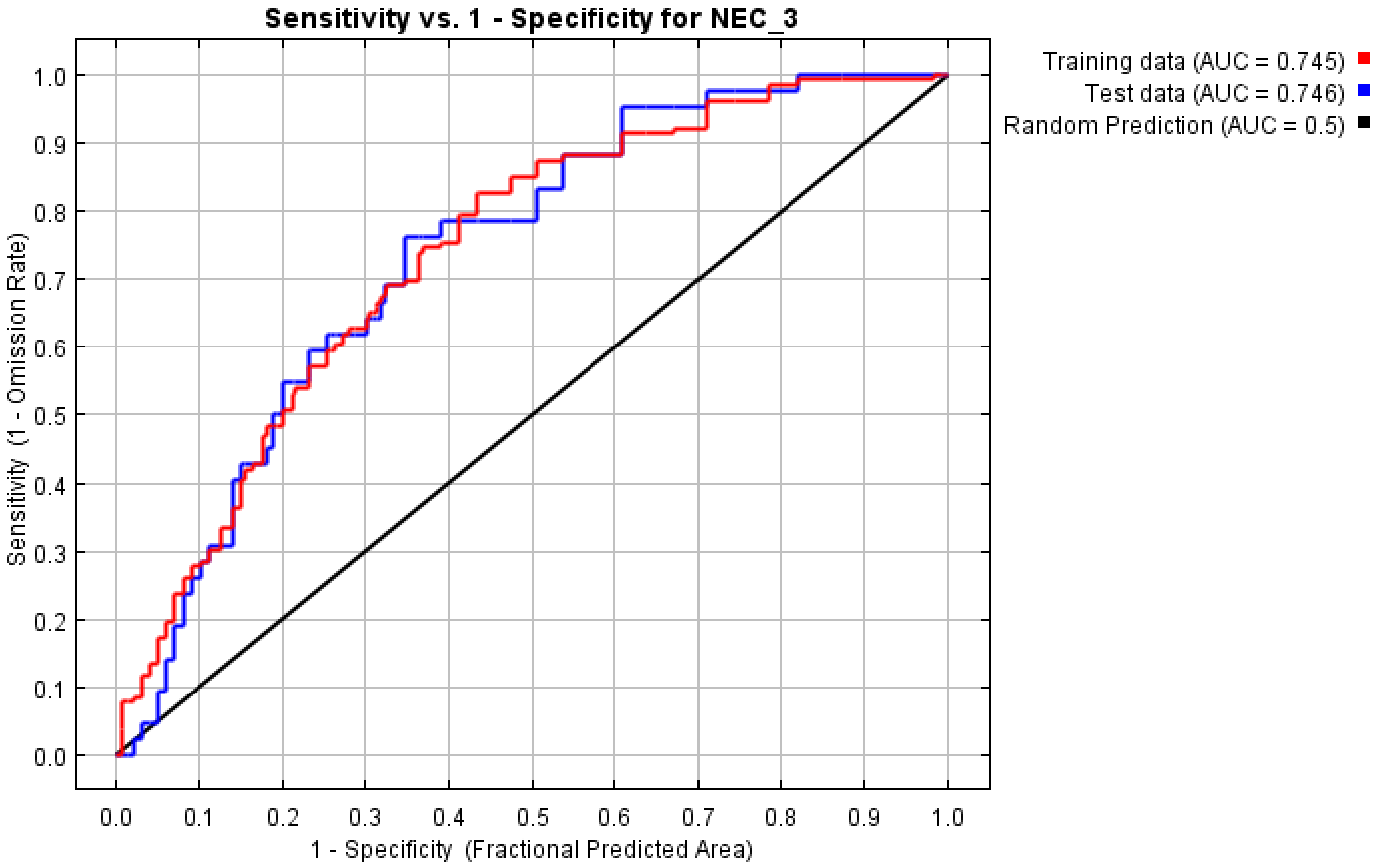

Some common thresholds and corresponding omission rates are as follows. If test data are available, binomial probabilities are calculated exactly if the number of test samples is at most 25, otherwise using a normal approximation to the binomial. These are 1-sided p-values for the null hypothesis that test points are predicted no better than by a random prediction with the same fractional predicted area. The "Balance" threshold minimizes 6 * training omission rate + .04 * cumulative threshold + 1.6 * fractional predicted area.

Cumulative threshold	Logistic threshold	Description	Fractional predicted area	Training omission rate	Test omission rate	P-value
1.000	0.065	Fixed cumulative value 1	0.874	0.008	0.000	6.999E-3
5.000	0.237	Fixed cumulative value 5	0.709	0.040	0.024	6.916E-5
10.000	0.271	Fixed cumulative value 10	0.609	0.087	0.048	2.608E-6
0.107	0.059	Minimum training presence	0.985	0.000	0.000	2.119E-1
10.467	0.271	10 percentile training presence	0.609	0.087	0.048	2.608E-6
35.631	0.517	Equal training sensitivity and specificity	0.324	0.325	0.310	2.013E-7
21.398	0.441	Maximum training sensitivity plus	0.434	0.175	0.214	2.152E-

		specificity				6
35.631	0.517	Equal test sensitivity and specificity	0.324	0.325	0.333	1.079E-6
32.415	0.513	Maximum test sensitivity plus specificity	0.346	0.302	0.238	7.604E-9
1.590	0.212	Balance training omission, predicted area and threshold value	0.820	0.008	0.000	1.189E-3
5.915	0.237	Equate entropy of thresholded and original distributions	0.709	0.040	0.024	6.916E-5

Click here to interactively explore this prediction using the Explain tool. If clicking from your browser does not succeed in starting the tool, try running the script in C:\Users\Bill\Desktop\Maxent Results NEC samples entire area\NEC_3_explain.bat directly. This tool requires the environmental grids to be small enough that they all fit in memory.

Response curves

These curves show how each environmental variable affects the Maxent prediction. The curves show how the logistic prediction changes as each environmental variable is varied, keeping all other environmental variables at their average sample value. Click on a response curve to see a larger version. Note that the curves can be hard to interpret if you have strongly correlated variables, as the model may depend on the correlations in ways that are not evident in the curves. In other words, the curves show the marginal effect of changing exactly one variable, whereas the model may take advantage of sets of variables changing together.

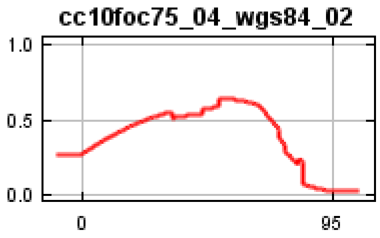

In contrast to the above marginal response curves, each of the following curves represents a different model, namely, a Maxent model created using only the corresponding variable. These plots reflect the dependence of predicted suitability both on the selected variable and on dependencies induced by correlations between the selected variable and other variables. They may be easier to interpret if there are strong correlations between variables.

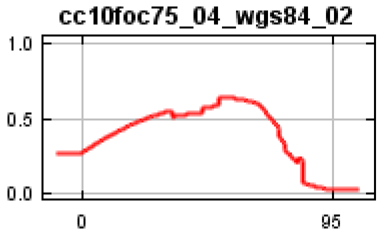

Analysis of variable contributions

The following table gives estimates of relative contributions of the environmental variables to the Maxent model. To determine the first estimate, in each iteration of the training algorithm, the increase in regularized gain is added to the contribution of the corresponding variable, or subtracted from it if the change to the absolute value of lambda is negative. For the second estimate, for each environmental variable in turn, the values of that variable on training presence and background data are randomly permuted. The model is reevaluated on the permuted data, and the resulting drop in training AUC is shown in the table, normalized to percentages. As with the variable jackknife, variable contributions should be interpreted with caution when the predictor variables are correlated.

Variable	Percent contribution	Permutation importance
cc10foc75_04_wgs84_02	100	100

Raw data outputs and control parameters

The data used in the above analysis is contained in the next links. Please see the Help button for more information on these.
The model applied to the training environmental layers
The coefficients of the model
The omission and predicted area for varying cumulative and raw thresholds
The prediction strength at the training and (optionally) test presence sites
Results for all species modeled in the same Maxent run, with summary statistics and (optionally) jackknife results

Regularized training gain is 0.328, training AUC is 0.745, unregularized training gain is 0.401.
Unregularized test gain is 0.391.
Test AUC is 0.746, standard deviation is 0.031 (calculated as in DeLong, DeLong & Clarke-Pearson 1988, equation 2).
Algorithm terminated after 500 iterations (1 seconds).

The follow settings were used during the run:
126 presence records used for training, 42 for testing.
10000 points used to determine the Maxent distribution (background points and presence points).
Environmental layers used (all continuous): cc10foc75_04_wgs84_02
Regularization values: linear/quadratic/product: 0.050, categorical: 0.250, threshold: 1.000, hinge: 0.500
Feature types used: hinge linear threshold quadratic
responsecurves: true
pictures: false
outputdirectory: C:\Users\Bill\Desktop\Maxent Results NEC samples entire area
samplesfile: C:\Users\Bill\Desktop\Maxent_samples\Compare_NEC_EC_150m_311_WGS_84_m.csv
environmentallayers: C:\Users\Bill\Desktop\Maxent Layers\maxent.cache
randomseed: true
randomtestpoints: 25
replicates: 10
replicatetype: subsample

Maxent model for NEC_4

This page contains some analysis of the Maxent model for NEC_4, created Sun Jul 12 03:10:24 EDT 2015 using Maxent version 3.3.3k. If you would like to do further analyses, the raw data used here is linked to at the end of this page.

Analysis of omission/commission

The following picture shows the omission rate and predicted area as a function of the cumulative threshold. The omission rate is is calculated both on the training presence records, and (if test data are used) on the test records. The omission rate should be close to the predicted omission, because of the definition of the cumulative threshold.

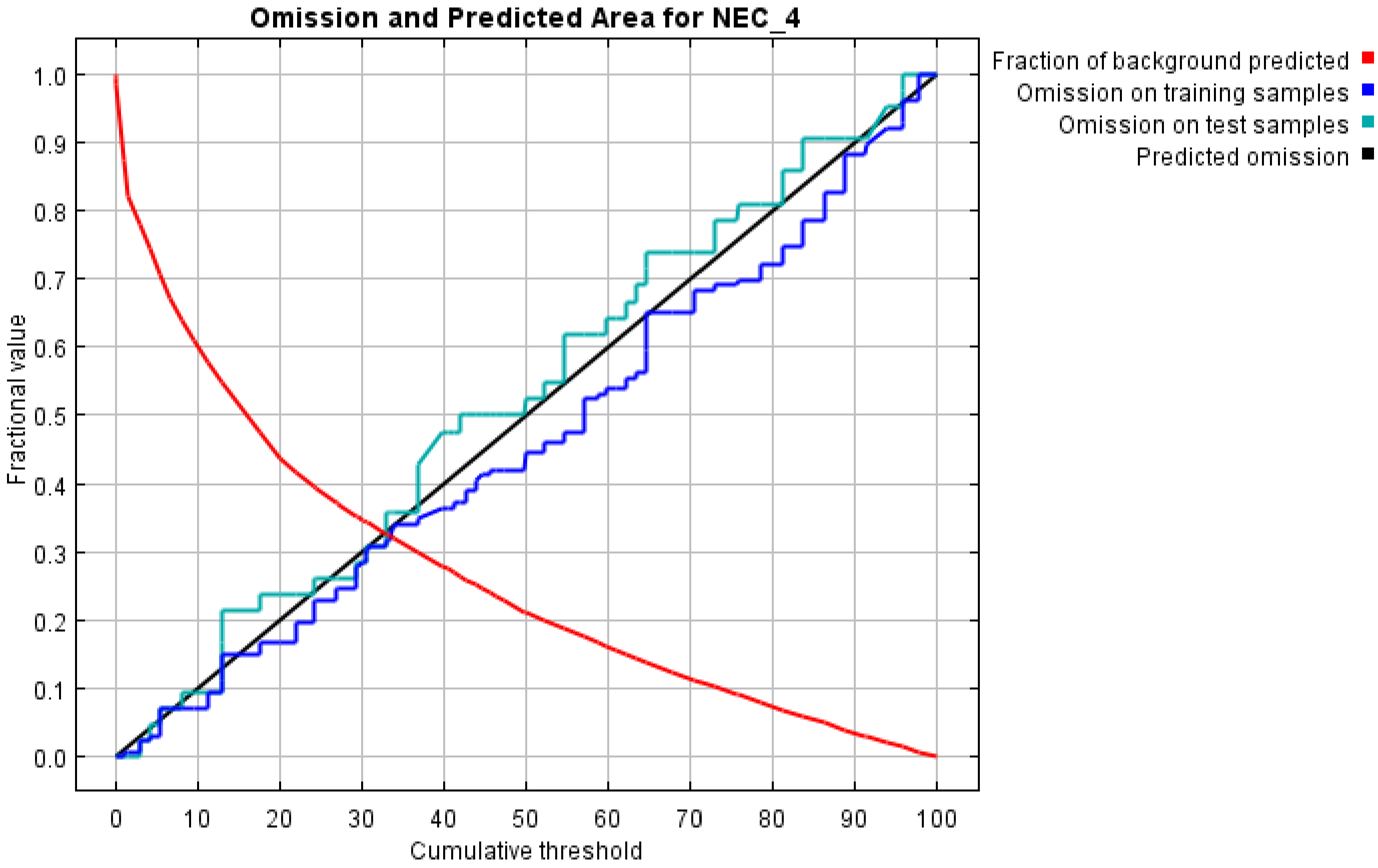

The next picture is the receiver operating characteristic (ROC) curve for the same data. Note that the specificity is defined using predicted area, rather than true commission (see the paper by Phillips, Anderson and Schapire cited on the help page for discussion of what this means). This implies that the maximum achievable AUC is less than 1. If test data is drawn from the Maxent distribution itself, then the maximum possible test AUC would be 0.731 rather than 1; in practice the test AUC may exceed this bound.

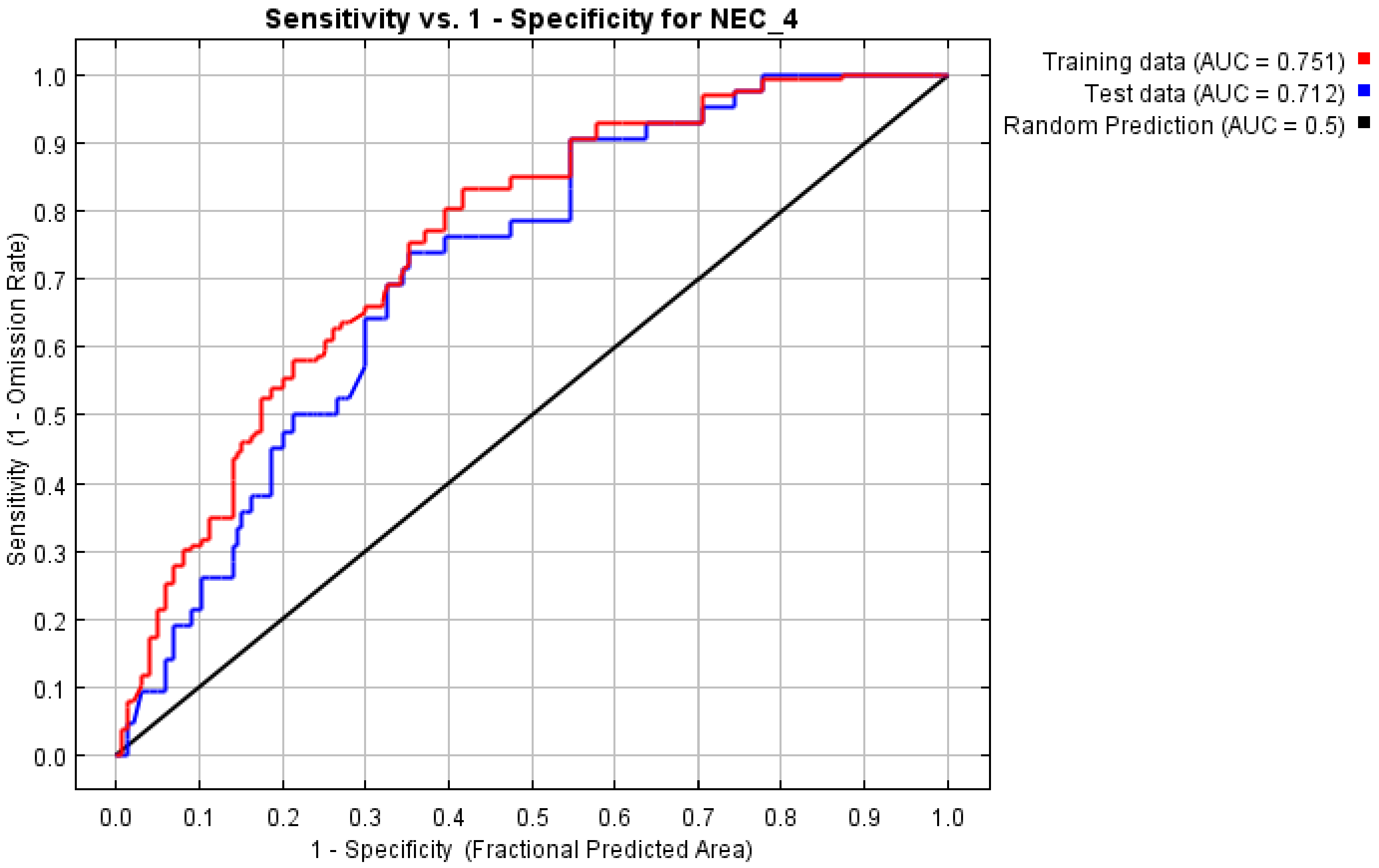

Some common thresholds and corresponding omission rates are as follows. If test data are available, binomial probabilities are calculated exactly if the number of test samples is at most 25, otherwise using a normal approximation to the binomial. These are 1-sided p-values for the null hypothesis that test points are predicted no better than by a random prediction with the same fractional predicted area. The "Balance" threshold minimizes 6 * training omission rate + .04 * cumulative threshold + 1.6 * fractional predicted area.

Cumulative threshold	Logistic threshold	Description	Fractional predicted area	Training omission rate	Test omission rate	P-value
1.000	0.057	Fixed cumulative value 1	0.874	0.000	0.000	6.999E-3
5.000	0.193	Fixed cumulative value 5	0.706	0.032	0.048	2.249E-4
10.000	0.277	Fixed cumulative value 10	0.578	0.071	0.095	9.118E-6
1.052	0.057	Minimum training presence	0.874	0.000	0.000	6.999E-3
12.962	0.299	10 percentile training presence	0.548	0.095	0.095	1.666E-6
33.436	0.505	Equal training sensitivity and specificity	0.323	0.317	0.357	4.687E-6
22.028	0.396	Maximum training sensitivity plus	0.416	0.167	0.238	2.726E-

		specificity				6
32.862	0.497	Equal test sensitivity and specificity	0.327	0.310	0.310	2.566E-7
29.254	0.472	Maximum test sensitivity plus specificity	0.353	0.246	0.262	8.922E-8
2.851	0.176	Balance training omission, predicted area and threshold value	0.778	0.008	0.000	2.682E-4
6.630	0.229	Equate entropy of thresholded and original distributions	0.671	0.071	0.071	1.93E-4

Click here to interactively explore this prediction using the Explain tool. If clicking from your browser does not succeed in starting the tool, try running the script in C:\Users\Bill\Desktop\Maxent Results NEC samples entire area\NEC_4_explain.bat directly. This tool requires the environmental grids to be small enough that they all fit in memory.

Response curves

These curves show how each environmental variable affects the Maxent prediction. The curves show how the logistic prediction changes as each environmental variable is varied, keeping all other environmental variables at their average sample value. Click on a response curve to see a larger version. Note that the curves can be hard to interpret if you have strongly correlated variables, as the model may depend on the correlations in ways that are not evident in the curves. In other words, the curves show the marginal effect of changing exactly one variable, whereas the model may take advantage of sets of variables changing together.

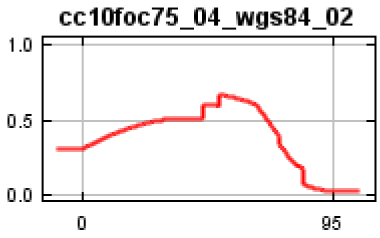

In contrast to the above marginal response curves, each of the following curves represents a different model, namely, a Maxent model created using only the corresponding variable. These plots reflect the dependence of predicted suitability both on the selected variable and on dependencies induced by correlations between the selected variable and other variables. They may be easier to interpret if there are strong correlations between variables.

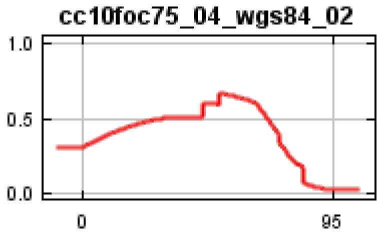

Analysis of variable contributions

The following table gives estimates of relative contributions of the environmental variables to the Maxent model. To determine the first estimate, in each iteration of the training algorithm, the increase in regularized gain is added to the contribution of the corresponding variable, or subtracted from it if the change to the absolute value of lambda is negative. For the second estimate, for each environmental variable in turn, the values of that variable on training presence and background data are randomly permuted. The model is reevaluated on the permuted data, and the resulting drop in training AUC is shown in the table, normalized to percentages. As with the variable jackknife, variable contributions should be interpreted with caution when the predictor variables are correlated.

Variable	Percent contribution	Permutation importance
cc10foc75_04_wgs84_02	100	100

Raw data outputs and control parameters

The data used in the above analysis is contained in the next links. Please see the Help button for more information on these.
The model applied to the training environmental layers
The coefficients of the model
The omission and predicted area for varying cumulative and raw thresholds
The prediction strength at the training and (optionally) test presence sites
Results for all species modeled in the same Maxent run, with summary statistics and (optionally) jackknife results

Regularized training gain is 0.356, training AUC is 0.751, unregularized training gain is 0.425.
Unregularized test gain is 0.328.
Test AUC is 0.712, standard deviation is 0.033 (calculated as in DeLong, DeLong & Clarke-Pearson 1988, equation 2).
Algorithm converged after 480 iterations (1 seconds).

The follow settings were used during the run:
126 presence records used for training, 42 for testing.
10000 points used to determine the Maxent distribution (background points and presence points).
Environmental layers used (all continuous): cc10foc75_04_wgs84_02
Regularization values: linear/quadratic/product: 0.050, categorical: 0.250, threshold: 1.000, hinge: 0.500
Feature types used: hinge linear threshold quadratic
responsecurves: true
pictures: false
outputdirectory: C:\Users\Bill\Desktop\Maxent Results NEC samples entire area
samplesfile: C:\Users\Bill\Desktop\Maxent_samples\Compare_NEC_EC_150m_311_WGS_84_m.csv
environmentallayers: C:\Users\Bill\Desktop\Maxent Layers\maxent.cache
randomseed: true
randomtestpoints: 25
replicates: 10
replicatetype: subsample

Maxent model for NEC_5

This page contains some analysis of the Maxent model for NEC_5, created Sun Jul 12 04:30:59 EDT 2015 using Maxent version 3.3.3k. If you would like to do further analyses, the raw data used here is linked to at the end of this page.

Analysis of omission/commission

The following picture shows the omission rate and predicted area as a function of the cumulative threshold. The omission rate is is calculated both on the training presence records, and (if test data are used) on the test records. The omission rate should be close to the predicted omission, because of the definition of the cumulative threshold.

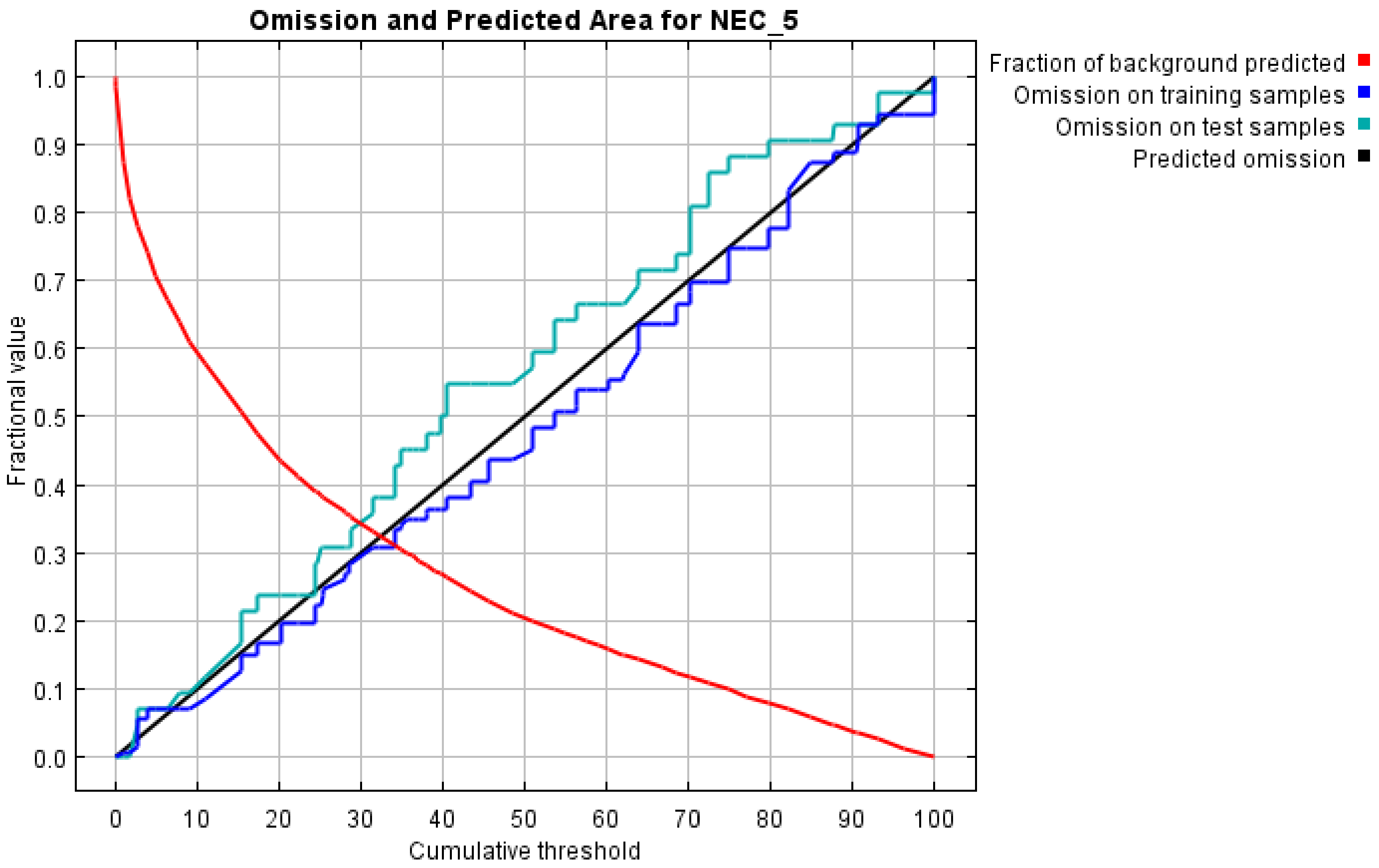

The next picture is the receiver operating characteristic (ROC) curve for the same data. Note that the specificity is defined using predicted area, rather than true commission (see the paper by Phillips, Anderson and Schapire cited on the help page for discussion of what this means). This implies that the maximum achievable AUC is less than 1. If test data is drawn from the Maxent distribution itself, then the maximum possible test AUC would be 0.734 rather than 1; in practice the test AUC may exceed this bound.

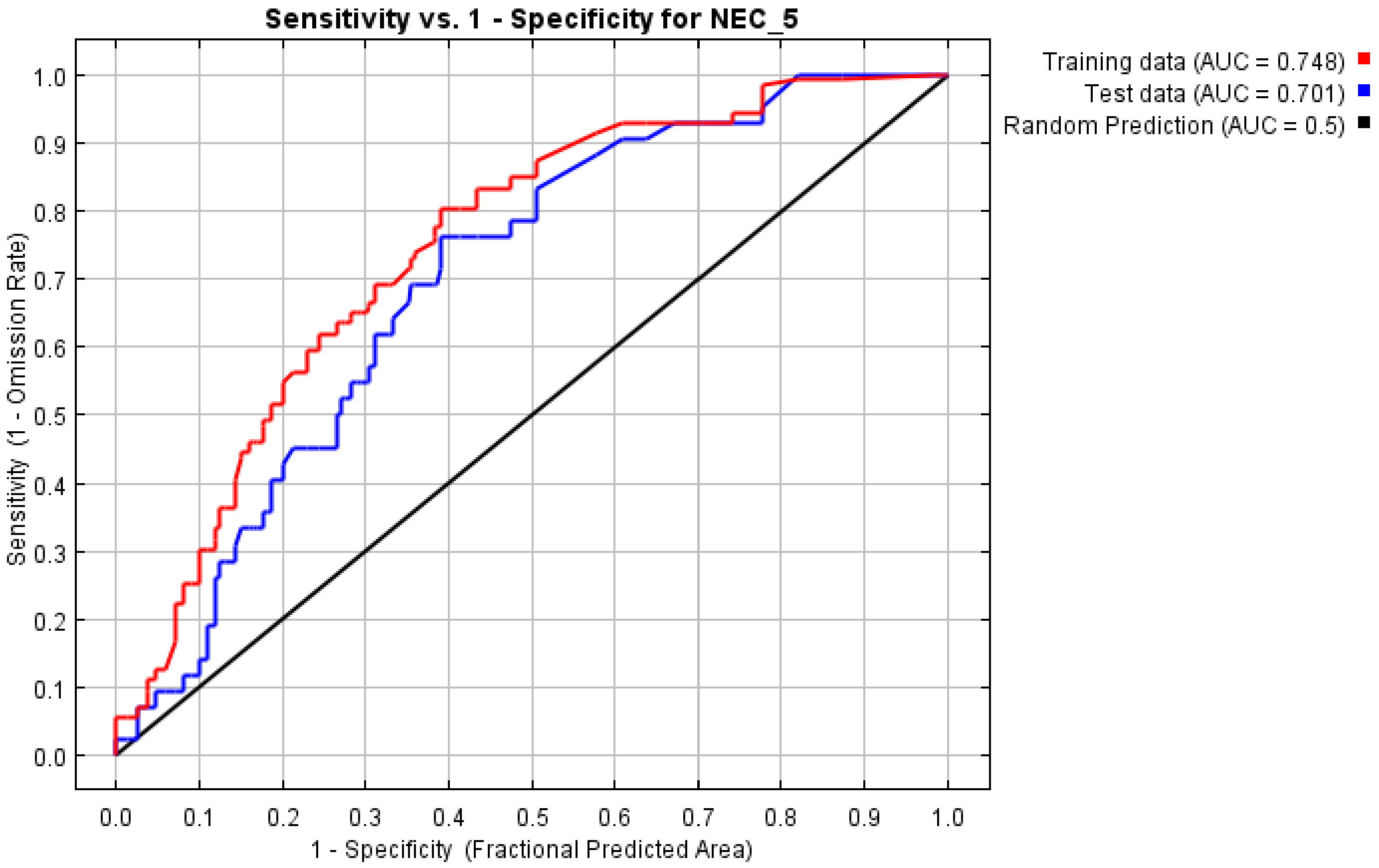

Some common thresholds and corresponding omission rates are as follows. If test data are available, binomial probabilities are calculated exactly if the number of test samples is at most 25, otherwise using a normal approximation to the binomial. These are 1-sided p-values for the null hypothesis that test points are predicted no better than by a random prediction with the same fractional predicted area. The "Balance" threshold minimizes 6 * training omission rate + .04 * cumulative threshold + 1.6 * fractional predicted area.

Cumulative threshold	Logistic threshold	Description	Fractional predicted area	Training omission rate	Test omission rate	P-value
1.000	0.064	Fixed cumulative value 1	0.874	0.008	0.000	6.999E-3
5.000	0.232	Fixed cumulative value 5	0.671	0.071	0.071	1.93E-4
10.000	0.298	Fixed cumulative value 10	0.578	0.087	0.119	3.548E-5
0.102	0.056	Minimum training presence	0.985	0.000	0.000	2.119E-1
10.884	0.298	10 percentile training presence	0.578	0.087	0.119	3.548E-5
34.235	0.476	Equal training sensitivity and specificity	0.311	0.310	0.381	7.933E-6
24.382	0.413	Maximum training sensitivity plus specificity	0.391	0.198	0.238	4.333E-7

28.883	0.469	Equal test sensitivity and specificity	0.352	0.286	0.333	1.007E-5
24.382	0.413	Maximum test sensitivity plus specificity	0.391	0.198	0.238	4.333E-7
1.593	0.152	Balance training omission, predicted area and threshold value	0.820	0.008	0.000	1.189E-3
6.253	0.232	Equate entropy of thresholded and original distributions	0.671	0.071	0.071	1.93E-4

Click here to interactively explore this prediction using the Explain tool. If clicking from your browser does not succeed in starting the tool, try running the script in C:\Users\Bill\Desktop\Maxent Results NEC samples entire area\NEC_5_explain.bat directly. This tool requires the environmental grids to be small enough that they all fit in memory.

Response curves

These curves show how each environmental variable affects the Maxent prediction. The curves show how the logistic prediction changes as each environmental variable is varied, keeping all other environmental variables at their average sample value. Click on a response curve to see a larger version. Note that the curves can be hard to interpret if you have strongly correlated variables, as the model may depend on the correlations in ways that are not evident in the curves. In other words, the curves show the marginal effect of changing exactly one variable, whereas the model may take advantage of sets of variables changing together.

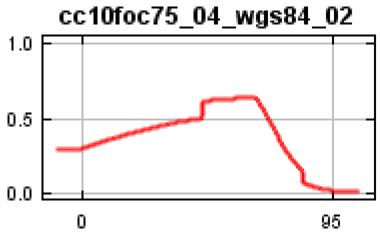

In contrast to the above marginal response curves, each of the following curves represents a different model, namely, a Maxent model created using only the corresponding variable. These plots reflect the dependence of predicted suitability both on the selected variable and on dependencies induced by correlations between the selected variable and other variables. They may be easier to interpret if there are strong correlations between variables.

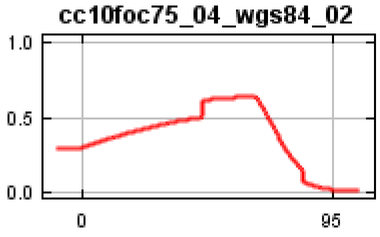

Analysis of variable contributions

The following table gives estimates of relative contributions of the environmental variables to the Maxent model. To determine the first estimate, in each iteration of the training algorithm, the increase in regularized gain is added to the contribution of the corresponding variable, or subtracted from it if the change to the absolute value of lambda is negative. For the second estimate, for each environmental variable in turn, the values of that variable on training presence and background data are randomly permuted. The model is reevaluated on the permuted data, and the resulting drop in training AUC is shown in the table, normalized to percentages. As with the variable jackknife, variable contributions should be interpreted with caution when the predictor variables are correlated.

Variable	Percent contribution	Permutation importance
cc10foc75_04_wgs84_02	100	100

Raw data outputs and control parameters

The data used in the above analysis is contained in the next links. Please see the Help button for more information on these.
The model applied to the training environmental layers
The coefficients of the model
The omission and predicted area for varying cumulative and raw thresholds
The prediction strength at the training and (optionally) test presence sites
Results for all species modeled in the same Maxent run, with summary statistics and (optionally) jackknife results

Regularized training gain is 0.365, training AUC is 0.748, unregularized training gain is 0.421.
Unregularized test gain is 0.290.
Test AUC is 0.701, standard deviation is 0.033 (calculated as in DeLong, DeLong & Clarke-Pearson 1988, equation 2).
Algorithm converged after 480 iterations (1 seconds).

The follow settings were used during the run:
126 presence records used for training, 42 for testing.
10000 points used to determine the Maxent distribution (background points and presence points).
Environmental layers used (all continuous): cc10foc75_04_wgs84_02
Regularization values: linear/quadratic/product: 0.050, categorical: 0.250, threshold: 1.000, hinge: 0.500
Feature types used: hinge linear threshold quadratic
responsecurves: true
pictures: false
outputdirectory: C:\Users\Bill\Desktop\Maxent Results NEC samples entire area
samplesfile: C:\Users\Bill\Desktop\Maxent_samples\Compare_NEC_EC_150m_311_WGS_84_m.csv
environmentallayers: C:\Users\Bill\Desktop\Maxent Layers\maxent.cache
randomseed: true
randomtestpoints: 25
replicates: 10
replicatetype: subsample
Command line used:

Maxent model for NEC_6

This page contains some analysis of the Maxent model for NEC_6, created Sun Jul 12 05:49:55 EDT 2015 using Maxent version 3.3.3k. If you would like to do further analyses, the raw data used here is linked to at the end of this page.

Analysis of omission/commission

The following picture shows the omission rate and predicted area as a function of the cumulative threshold. The omission rate is is calculated both on the training presence records, and (if test data are used) on the test records. The omission rate should be close to the predicted omission, because of the definition of the cumulative threshold.

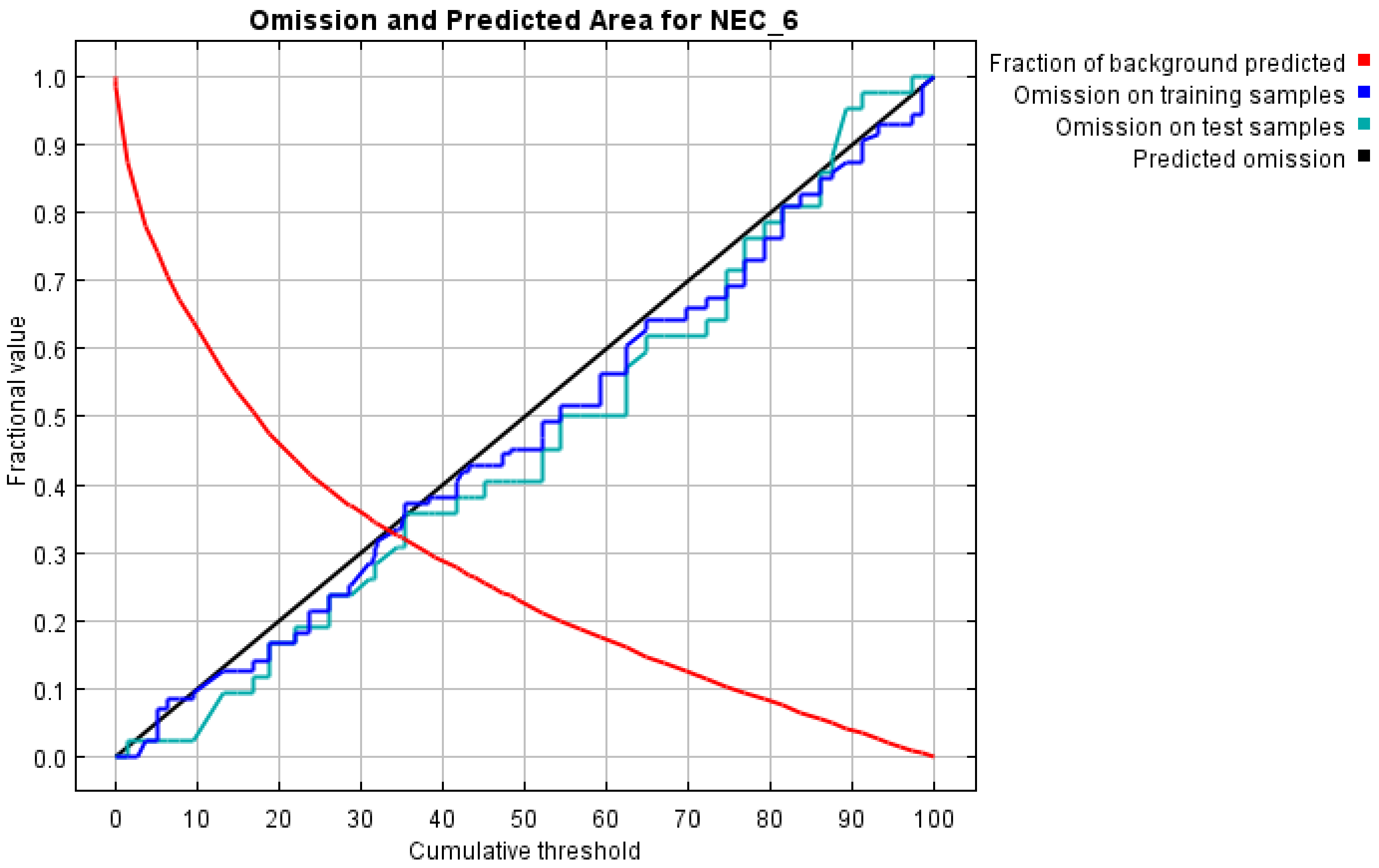

The next picture is the receiver operating characteristic (ROC) curve for the same data. Note that the specificity is defined using predicted area, rather than true commission (see the paper by Phillips, Anderson and Schapire cited on the help page for discussion of what this means). This implies that the maximum achievable AUC is less than 1. If test data is drawn from the Maxent distribution itself, then the maximum possible test AUC would be 0.718 rather than 1; in practice the test AUC may exceed this bound.

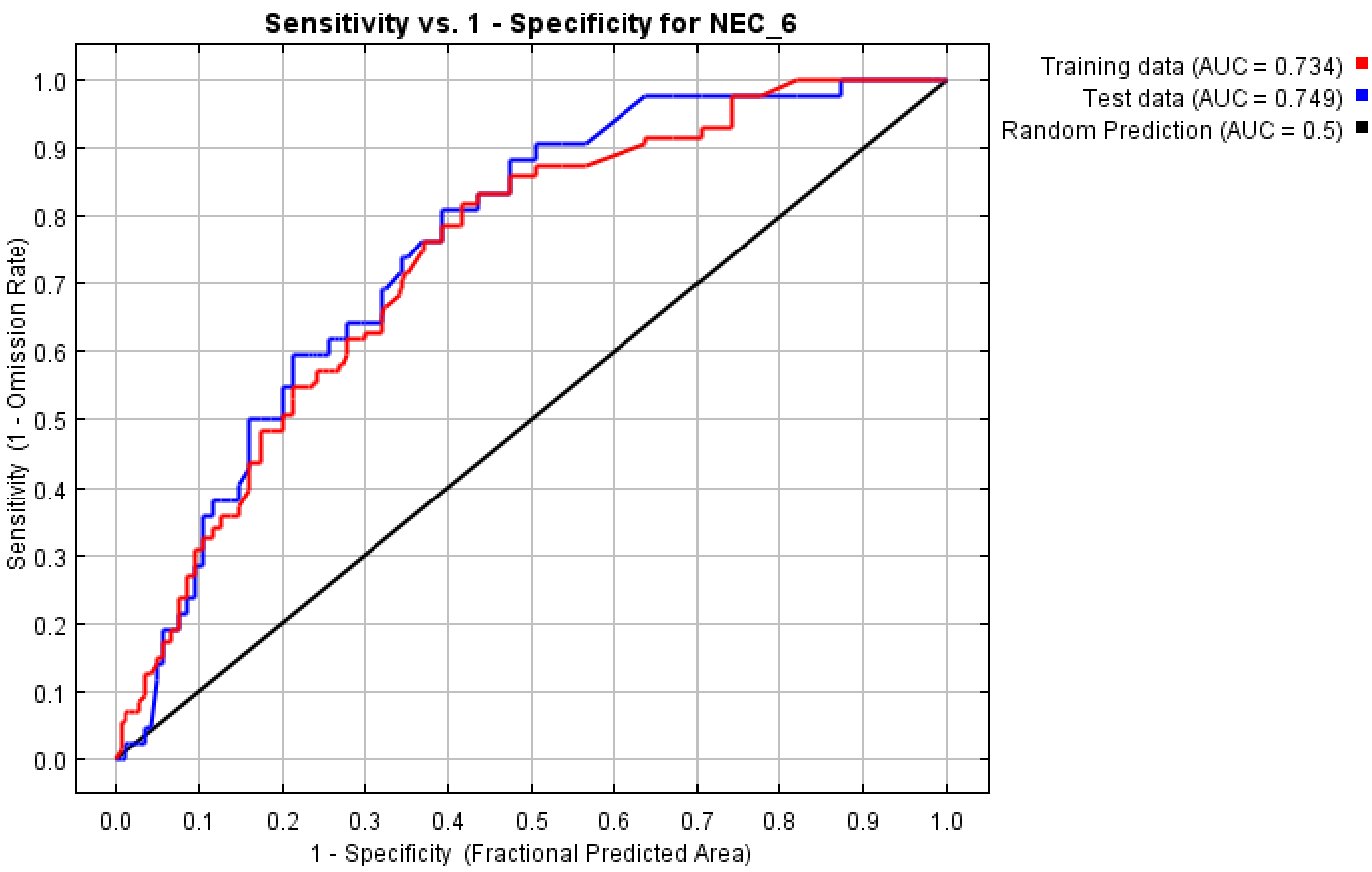

Some common thresholds and corresponding omission rates are as follows. If test data are available, binomial probabilities are calculated exactly if the number of test samples is at most 25, otherwise using a normal approximation to the binomial. These are 1-sided p-values for the null hypothesis that test points are predicted no better than by a random prediction with the same fractional predicted area. The "Balance" threshold minimizes 6 * training omission rate + .04 * cumulative threshold + 1.6 * fractional predicted area.

Cumulative threshold	Logistic threshold	Description	Fractional predicted area	Training omission rate	Test omission rate	P-value
1.000	0.088	Fixed cumulative value 1	0.874	0.000	0.000	6.999E-3
5.000	0.201	Fixed cumulative value 5	0.740	0.024	0.024	2.437E-4
10.000	0.289	Fixed cumulative value 10	0.566	0.127	0.095	4.72E-6
2.588	0.165	Minimum training presence	0.820	0.000	0.024	4.183E-3
9.513	0.270	10 percentile training presence	0.638	0.095	0.024	2.564E-6
34.271	0.507	Equal training sensitivity and specificity	0.327	0.333	0.310	2.566E-7
23.767	0.394	Maximum training sensitivity plus specificity	0.416	0.183	0.190	1.154E-7

35.278	0.526	Equal test sensitivity and specificity	0.320	0.357	0.310	1.336E-7
25.988	0.421	Maximum test sensitivity plus specificity	0.394	0.214	0.190	1.736E-8
2.588	0.165	Balance training omission, predicted area and threshold value	0.820	0.000	0.024	4.183E-3
6.361	0.221	Equate entropy of thresholded and original distributions	0.706	0.071	0.024	5.964E-5

Click here to interactively explore this prediction using the Explain tool. If clicking from your browser does not succeed in starting the tool, try running the script in C:\Users\Bill\Desktop\Maxent Results NEC samples entire area\NEC_6_explain.bat directly. This tool requires the environmental grids to be small enough that they all fit in memory.

Response curves

These curves show how each environmental variable affects the Maxent prediction. The curves show how the logistic prediction changes as each environmental variable is varied, keeping all other environmental variables at their average sample value. Click on a response curve to see a larger version. Note that the curves can be hard to interpret if you have strongly correlated variables, as the model may depend on the correlations in ways that are not evident in the curves. In other words, the curves show the marginal effect of changing exactly one variable, whereas the model may take advantage of sets of variables changing together.

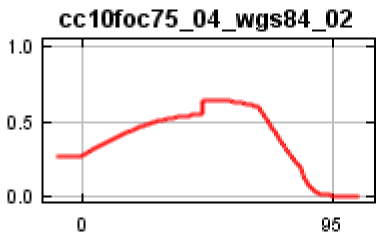

In contrast to the above marginal response curves, each of the following curves represents a different model, namely, a Maxent model created using only the corresponding variable. These plots reflect the dependence of predicted suitability both on the selected variable and on dependencies induced by correlations between the selected variable and other variables. They may be easier to interpret if there are strong correlations between variables.

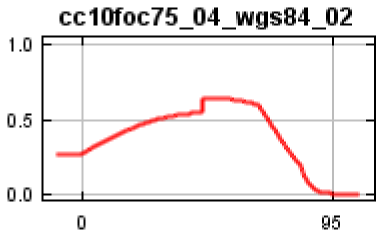

Analysis of variable contributions

The following table gives estimates of relative contributions of the environmental variables to the Maxent model. To determine the first estimate, in each iteration of the training algorithm, the increase in regularized gain is added to the contribution of the corresponding variable, or subtracted from it if the change to the absolute value of lambda is negative. For the second estimate, for each environmental variable in turn, the values of that variable on training presence and background data are randomly permuted. The model is reevaluated on the permuted data, and the resulting drop in training AUC is shown in the table, normalized to percentages. As with the variable jackknife, variable contributions should be interpreted with caution when the predictor variables are correlated.

Variable	Percent contribution	Permutation importance
cc10foc75_04_wgs84_02	100	100

Raw data outputs and control parameters

The data used in the above analysis is contained in the next links. Please see the Help button for more information on these.
The model applied to the training environmental layers
The coefficients of the model
The omission and predicted area for varying cumulative and raw thresholds
The prediction strength at the training and (optionally) test presence sites
Results for all species modeled in the same Maxent run, with summary statistics and (optionally) jackknife results

Regularized training gain is 0.313, training AUC is 0.734, unregularized training gain is 0.363.
Unregularized test gain is 0.403.
Test AUC is 0.749, standard deviation is 0.031 (calculated as in DeLong, DeLong & Clarke-Pearson 1988, equation 2).
Algorithm converged after 440 iterations (1 seconds).

The follow settings were used during the run:
126 presence records used for training, 42 for testing.
10000 points used to determine the Maxent distribution (background points and presence points).
Environmental layers used (all continuous): cc10foc75_04_wgs84_02
Regularization values: linear/quadratic/product: 0.050, categorical: 0.250, threshold: 1.000, hinge: 0.500
Feature types used: hinge linear threshold quadratic
responsecurves: true
pictures: false
outputdirectory: C:\Users\Bill\Desktop\Maxent Results NEC samples entire area
samplesfile: C:\Users\Bill\Desktop\Maxent_samples\Compare_NEC_EC_150m_311_WGS_84_m.csv
environmentallayers: C:\Users\Bill\Desktop\Maxent Layers\maxent.cache
randomseed: true
randomtestpoints: 25
replicates: 10
replicatetype: subsample
Command line used:

Maxent model for NEC_7

This page contains some analysis of the Maxent model for NEC_7, created Sun Jul 12 07:07:31 EDT 2015 using Maxent version 3.3.3k. If you would like to do further analyses, the raw data used here is linked to at the end of this page.

Analysis of omission/commission

The following picture shows the omission rate and predicted area as a function of the cumulative threshold. The omission rate is is calculated both on the training presence records, and (if test data are used) on the test records. The omission rate should be close to the predicted omission, because of the definition of the cumulative threshold.

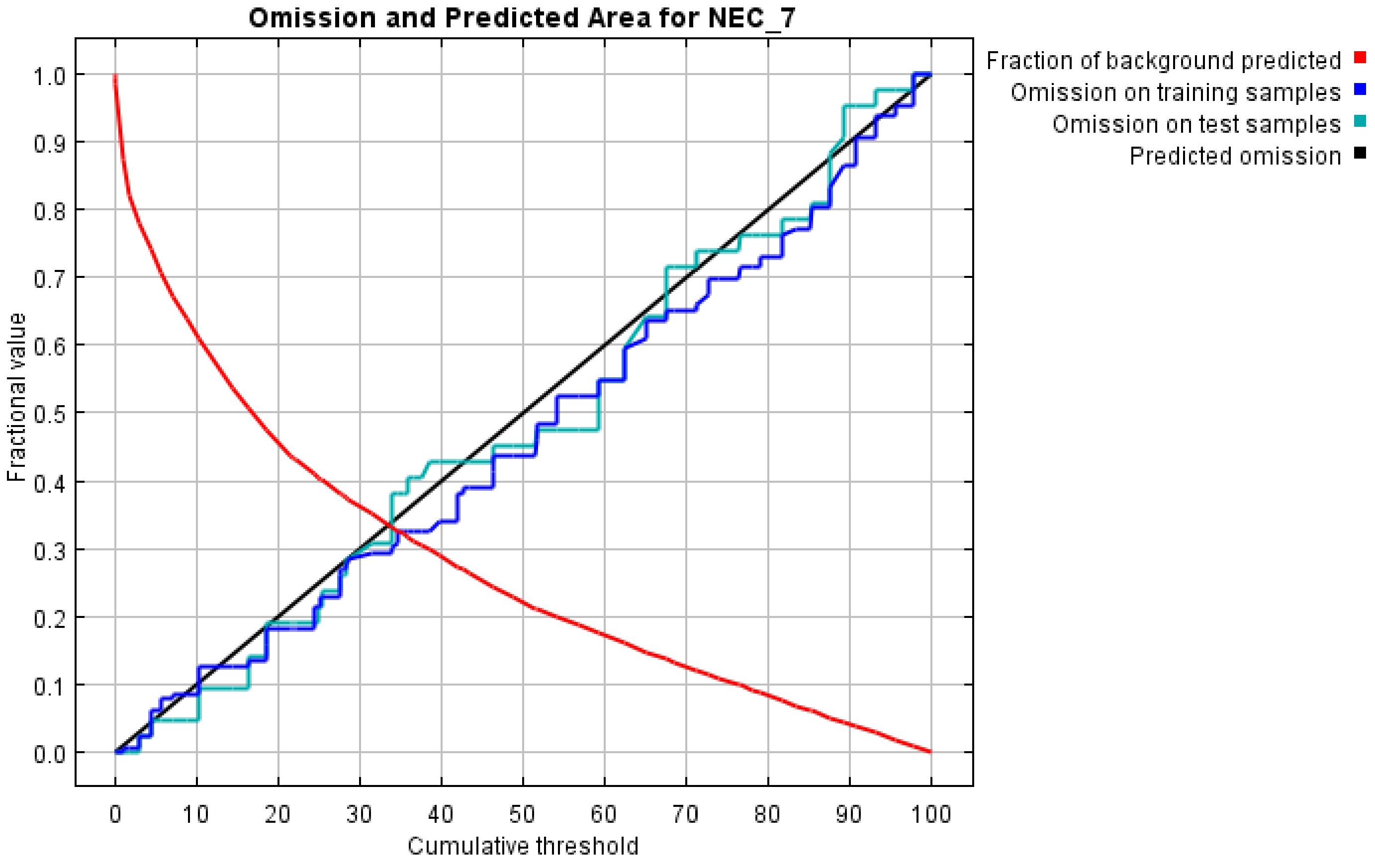

The next picture is the receiver operating characteristic (ROC) curve for the same data. Note that the specificity is defined using predicted area, rather than true commission (see the paper by Phillips, Anderson and Schapire cited on the help page for discussion of what this means). This implies that the maximum achievable AUC is less than 1. If test data is drawn from the Maxent distribution itself, then the maximum possible test AUC would be 0.721 rather than 1; in practice the test AUC may exceed this bound.

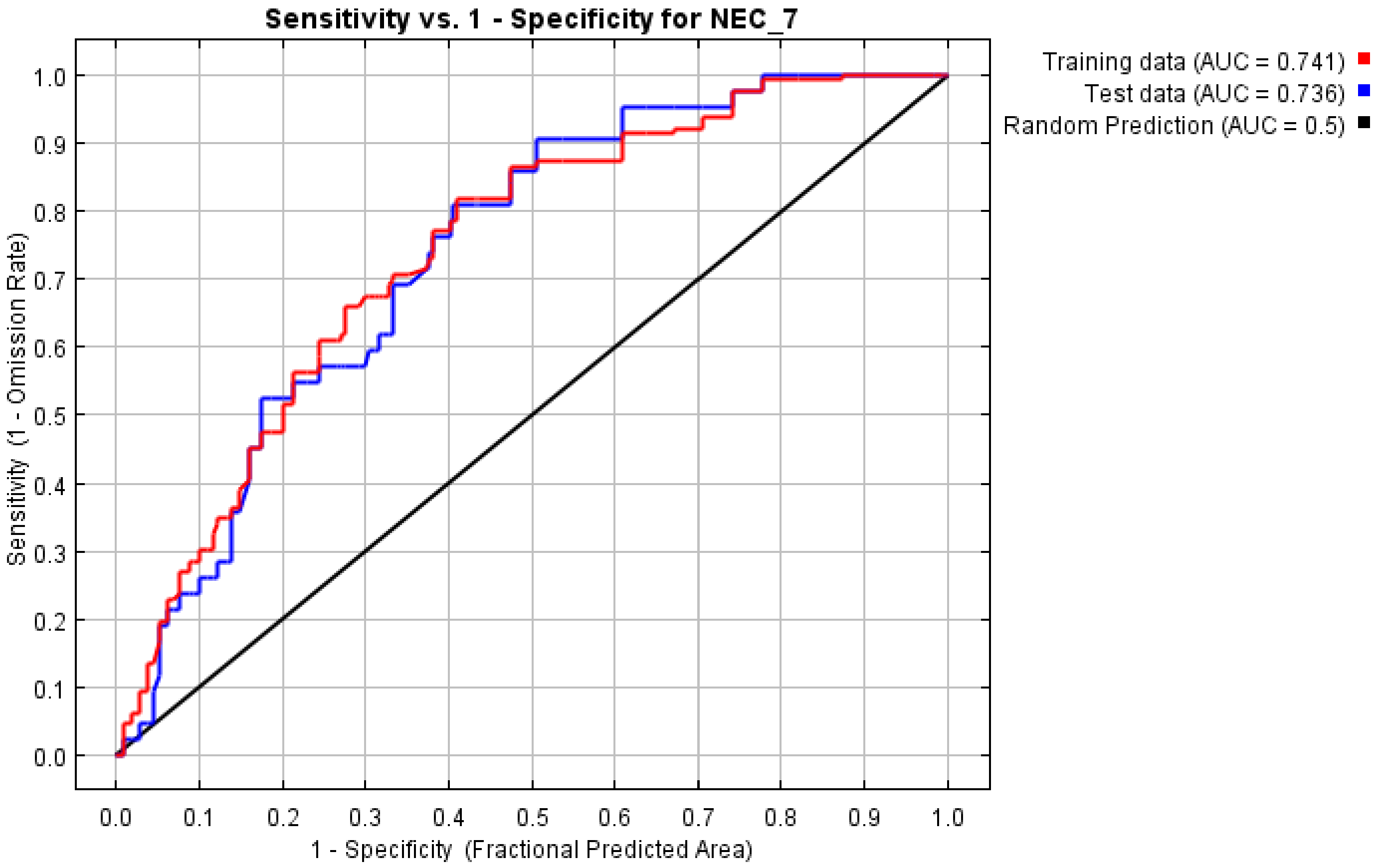

Some common thresholds and corresponding omission rates are as follows. If test data are available, binomial probabilities are calculated exactly if the number of test samples is at most 25, otherwise using a normal approximation to the binomial. These are 1-sided p-values for the null hypothesis that test points are predicted no better than by a random prediction with the same fractional predicted area. The "Balance" threshold minimizes 6 * training omission rate + .04 * cumulative threshold + 1.6 * fractional predicted area.

Cumulative threshold	Logistic threshold	Description	Fractional predicted area	Training omission rate	Test omission rate	P-value
1.000	0.059	Fixed cumulative value 1	0.874	0.000	0.000	6.999E-3
5.000	0.222	Fixed cumulative value 5	0.706	0.063	0.048	2.249E-4
10.000	0.292	Fixed cumulative value 10	0.609	0.087	0.048	2.608E-6
1.062	0.059	Minimum training presence	0.874	0.000	0.000	6.999E-3
10.308	0.292	10 percentile training presence	0.609	0.087	0.048	2.608E-6
34.654	0.490	Equal training sensitivity and specificity	0.327	0.325	0.381	2.775E-5
24.272	0.433	Maximum training sensitivity plus	0.410	0.183	0.190	7.327E-

		specificity				8
33.994	0.484	Equal test sensitivity and specificity	0.332	0.294	0.310	4.081E-7
24.844	0.442	Maximum test sensitivity plus specificity	0.405	0.214	0.190	4.753E-8
2.927	0.188	Balance training omission, predicted area and threshold value	0.778	0.008	0.000	2.682E-4
5.641	0.222	Equate entropy of thresholded and original distributions	0.706	0.063	0.048	2.249E-4

Click here to interactively explore this prediction using the Explain tool. If clicking from your browser does not succeed in starting the tool, try running the script in C:\Users\Bill\Desktop\Maxent Results NEC samples entire area\NEC_7_explain.bat directly. This tool requires the environmental grids to be small enough that they all fit in memory.

Response curves

These curves show how each environmental variable affects the Maxent prediction. The curves show how the logistic prediction changes as each environmental variable is varied, keeping all other environmental variables at their average sample value. Click on a response curve to see a larger version. Note that the curves can be hard to interpret if you have strongly correlated variables, as the model may depend on the correlations in ways that are not evident in the curves. In other words, the curves show the marginal effect of changing exactly one variable, whereas the model may take advantage of sets of variables changing together.

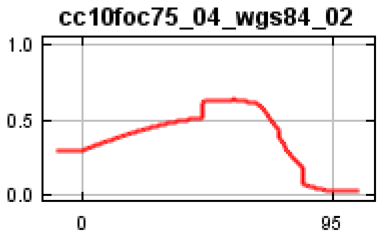

In contrast to the above marginal response curves, each of the following curves represents a different model, namely, a Maxent model created using only the corresponding variable. These plots reflect the dependence of predicted suitability both on the selected variable and on dependencies induced by correlations between the selected variable and other variables. They may be easier to interpret if there are strong correlations between variables.

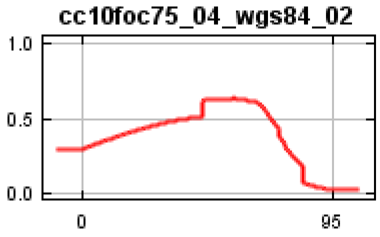

Analysis of variable contributions

The following table gives estimates of relative contributions of the environmental variables to the Maxent model. To determine the first estimate, in each iteration of the training algorithm, the increase in regularized gain is added to the contribution of the corresponding variable, or subtracted from it if the change to the absolute value of lambda is negative. For the second estimate, for each environmental variable in turn, the values of that variable on training presence and background data are randomly permuted. The model is reevaluated on the permuted data, and the resulting drop in training AUC is shown in the table, normalized to percentages. As with the variable jackknife, variable contributions should be interpreted with caution when the predictor variables are correlated.

Variable	Percent contribution	Permutation importance
cc10foc75_04_wgs84_02	100	100

Raw data outputs and control parameters

The data used in the above analysis is contained in the next links. Please see the Help button for more information on these.
The model applied to the training environmental layers
The coefficients of the model
The omission and predicted area for varying cumulative and raw thresholds
The prediction strength at the training and (optionally) test presence sites
Results for all species modeled in the same Maxent run, with summary statistics and (optionally) jackknife results

Regularized training gain is 0.329, training AUC is 0.741, unregularized training gain is 0.387.
Unregularized test gain is 0.393.
Test AUC is 0.736, standard deviation is 0.031 (calculated as in DeLong, DeLong & Clarke-Pearson 1988, equation 2).
Algorithm terminated after 500 iterations (1 seconds).

The follow settings were used during the run:
126 presence records used for training, 42 for testing.
10000 points used to determine the Maxent distribution (background points and presence points).
Environmental layers used (all continuous): cc10foc75_04_wgs84_02
Regularization values: linear/quadratic/product: 0.050, categorical: 0.250, threshold: 1.000, hinge: 0.500
Feature types used: hinge linear threshold quadratic
responsecurves: true
pictures: false
outputdirectory: C:\Users\Bill\Desktop\Maxent Results NEC samples entire area
samplesfile: C:\Users\Bill\Desktop\Maxent_samples\Compare_NEC_EC_150m_311_WGS_84_m.csv
environmentallayers: C:\Users\Bill\Desktop\Maxent Layers\maxent.cache
randomseed: true
randomtestpoints: 25
replicates: 10
replicatetype: subsample

Maxent model for NEC_8

This page contains some analysis of the Maxent model for NEC_8, created Sun Jul 12 08:28:16 EDT 2015 using Maxent version 3.3.3k. If you would like to do further analyses, the raw data used here is linked to at the end of this page.

Analysis of omission/commission

The following picture shows the omission rate and predicted area as a function of the cumulative threshold. The omission rate is is calculated both on the training presence records, and (if test data are used) on the test records. The omission rate should be close to the predicted omission, because of the definition of the cumulative threshold.

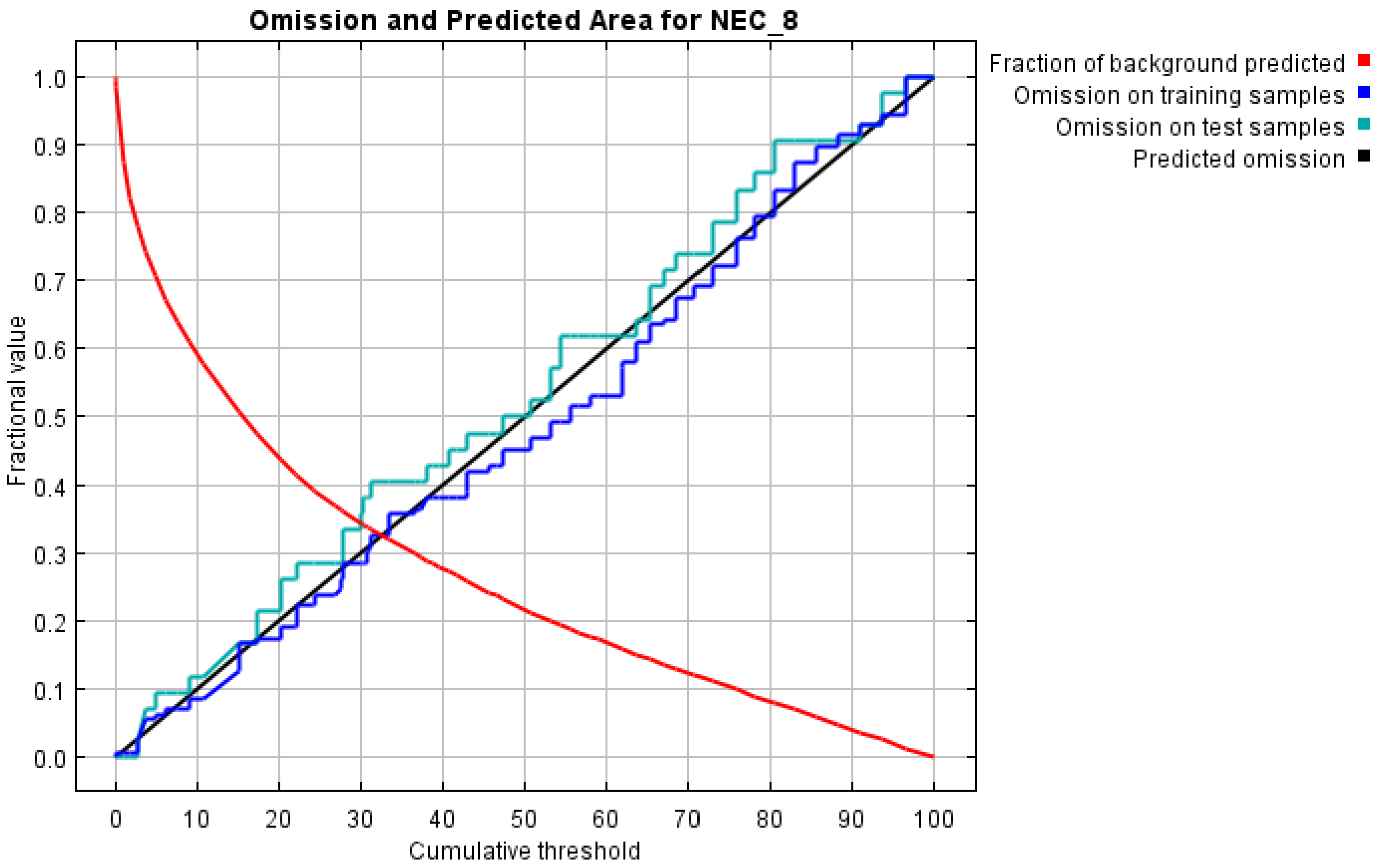

The next picture is the receiver operating characteristic (ROC) curve for the same data. Note that the specificity is defined using predicted area, rather than true commission (see the paper by Phillips, Anderson and Schapire cited on the help page for discussion of what this means). This implies that the maximum achievable AUC is less than 1. If test data is drawn from the Maxent distribution itself, then the maximum possible test AUC would be 0.730 rather than 1; in practice the test AUC may exceed this bound.

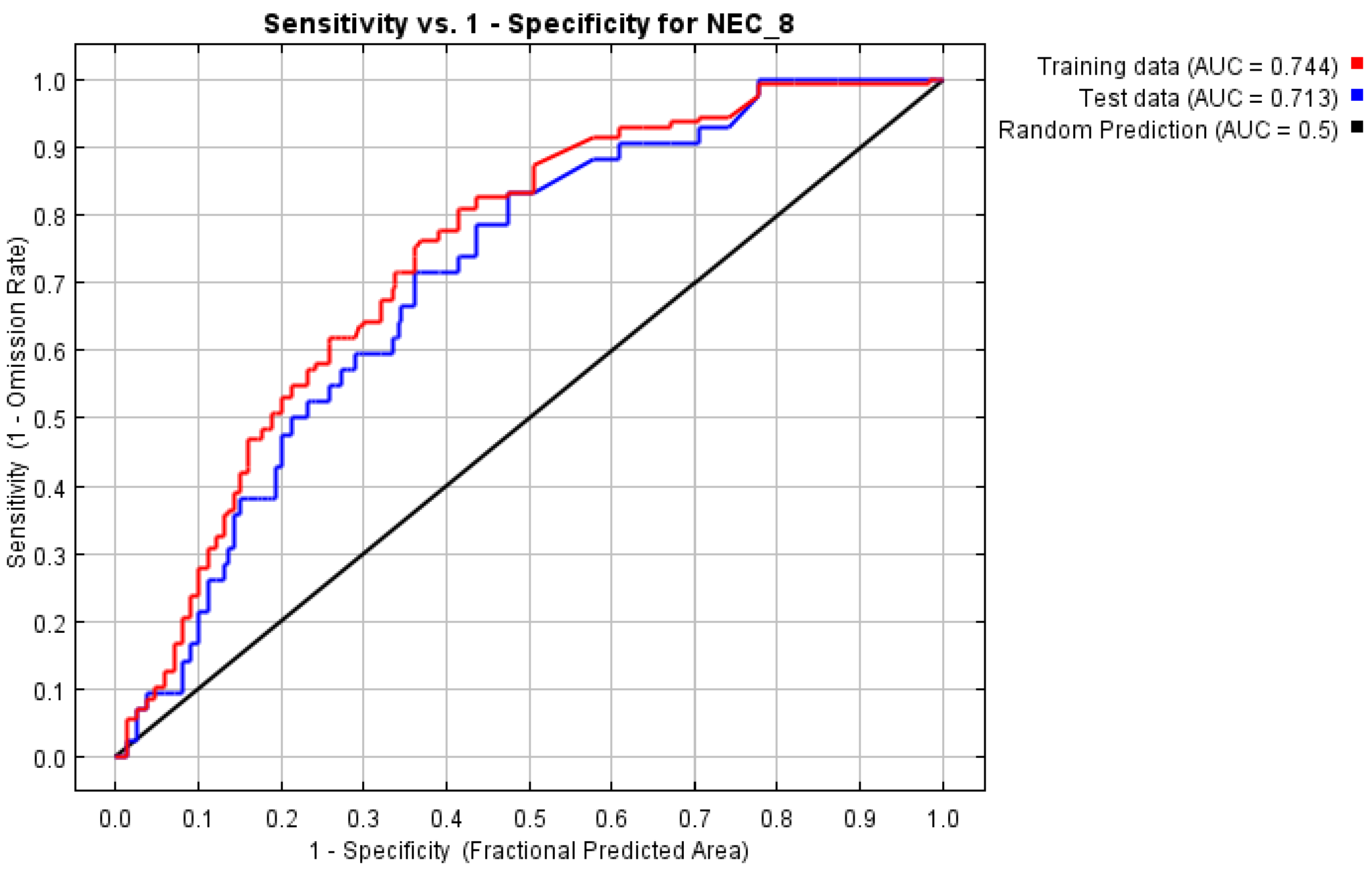

Some common thresholds and corresponding omission rates are as follows. If test data are available, binomial probabilities are calculated exactly if the number of test samples is at most 25, otherwise using a normal approximation to the binomial. These are 1-sided p-values for the null hypothesis that test points are predicted no better than by a random prediction with the same fractional predicted area. The "Balance" threshold minimizes 6 * training omission rate + .04 * cumulative threshold + 1.6 * fractional predicted area.

Cumulative threshold	Logistic threshold	Description	Fractional predicted area	Training omission rate	Test omission rate	P-value
1.000	0.065	Fixed cumulative value 1	0.874	0.008	0.000	6.999E-3
5.000	0.234	Fixed cumulative value 5	0.671	0.063	0.095	6.389E-4
10.000	0.299	Fixed cumulative value 10	0.578	0.087	0.119	3.548E-5
0.100	0.057	Minimum training presence	0.985	0.000	0.000	2.119E-1
10.831	0.299	10 percentile training presence	0.578	0.087	0.119	3.548E-5
33.457	0.510	Equal training sensitivity and specificity	0.320	0.325	0.405	6.614E-5
22.103	0.407	Maximum training sensitivity plus	0.414	0.190	0.262	9.961E-

		specificity				6
29.920	0.477	Equal test sensitivity and specificity	0.345	0.286	0.333	5.66E-6
17.212	0.344	Maximum test sensitivity plus specificity	0.476	0.167	0.167	1.723E-6
2.636	0.149	Balance training omission, predicted area and threshold value	0.778	0.008	0.000	2.682E-4
6.172	0.234	Equate entropy of thresholded and original distributions	0.671	0.063	0.095	6.389E-4

Click here to interactively explore this prediction using the Explain tool. If clicking from your browser does not succeed in starting the tool, try running the script in C:\Users\Bill\Desktop\Maxent Results NEC samples entire area\NEC_8_explain.bat directly. This tool requires the environmental grids to be small enough that they all fit in memory.

Response curves

These curves show how each environmental variable affects the Maxent prediction. The curves show how the logistic prediction changes as each environmental variable is varied, keeping all other environmental variables at their average sample value. Click on a response curve to see a larger version. Note that the curves can be hard to interpret if you have strongly correlated variables, as the model may depend on the correlations in ways that are not evident in the curves. In other words, the curves show the marginal effect of changing exactly one variable, whereas the model may take advantage of sets of variables changing together.

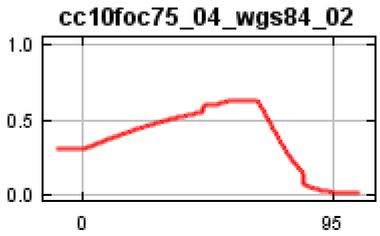

In contrast to the above marginal response curves, each of the following curves represents a different model, namely, a Maxent model created using only the corresponding variable. These plots reflect the dependence of predicted suitability both on the selected variable and on dependencies induced by correlations between the selected variable and other variables. They may be easier to interpret if there are strong correlations between variables.

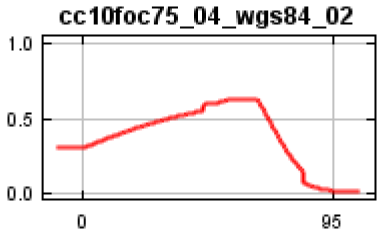

Analysis of variable contributions

The following table gives estimates of relative contributions of the environmental variables to the Maxent model. To determine the first estimate, in each iteration of the training algorithm, the increase in regularized gain is added to the contribution of the corresponding variable, or subtracted from it if the change to the absolute value of lambda is negative. For the second estimate, for each environmental variable in turn, the values of that variable on training presence and background data are randomly permuted. The model is reevaluated on the permuted data, and the resulting drop in training AUC is shown in the table, normalized to percentages. As with the variable jackknife, variable contributions should be interpreted with caution when the predictor variables are correlated.

Variable	Percent contribution	Permutation importance
cc10foc75_04_wgs84_02	100	100

Raw data outputs and control parameters

The data used in the above analysis is contained in the next links. Please see the Help button for more information on these.
The model applied to the training environmental layers
The coefficients of the model
The omission and predicted area for varying cumulative and raw thresholds
The prediction strength at the training and (optionally) test presence sites
Results for all species modeled in the same Maxent run, with summary statistics and (optionally) jackknife results

Regularized training gain is 0.355, training AUC is 0.744, unregularized training gain is 0.403.
Unregularized test gain is 0.320.
Test AUC is 0.713, standard deviation is 0.033 (calculated as in DeLong, DeLong & Clarke-Pearson 1988, equation 2).
Algorithm terminated after 500 iterations (1 seconds).

The follow settings were used during the run:
126 presence records used for training, 42 for testing.
10000 points used to determine the Maxent distribution (background points and presence points).
Environmental layers used (all continuous): cc10foc75_04_wgs84_02
Regularization values: linear/quadratic/product: 0.050, categorical: 0.250, threshold: 1.000, hinge: 0.500
Feature types used: hinge linear threshold quadratic
responsecurves: true
pictures: false
outputdirectory: C:\Users\Bill\Desktop\Maxent Results NEC samples entire area
samplesfile: C:\Users\Bill\Desktop\Maxent_samples\Compare_NEC_EC_150m_311_WGS_84_m.csv
environmentallayers: C:\Users\Bill\Desktop\Maxent Layers\maxent.cache
randomseed: true
randomtestpoints: 25
replicates: 10
replicatetype: subsample

Maxent model for NEC_9

This page contains some analysis of the Maxent model for NEC_9, created Sun Jul 12 09:47:58 EDT 2015 using Maxent version 3.3.3k. If you would like to do further analyses, the raw data used here is linked to at the end of this page.

Analysis of omission/commission

The following picture shows the omission rate and predicted area as a function of the cumulative threshold. The omission rate is is calculated both on the training presence records, and (if test data are used) on the test records. The omission rate should be close to the predicted omission, because of the definition of the cumulative threshold.

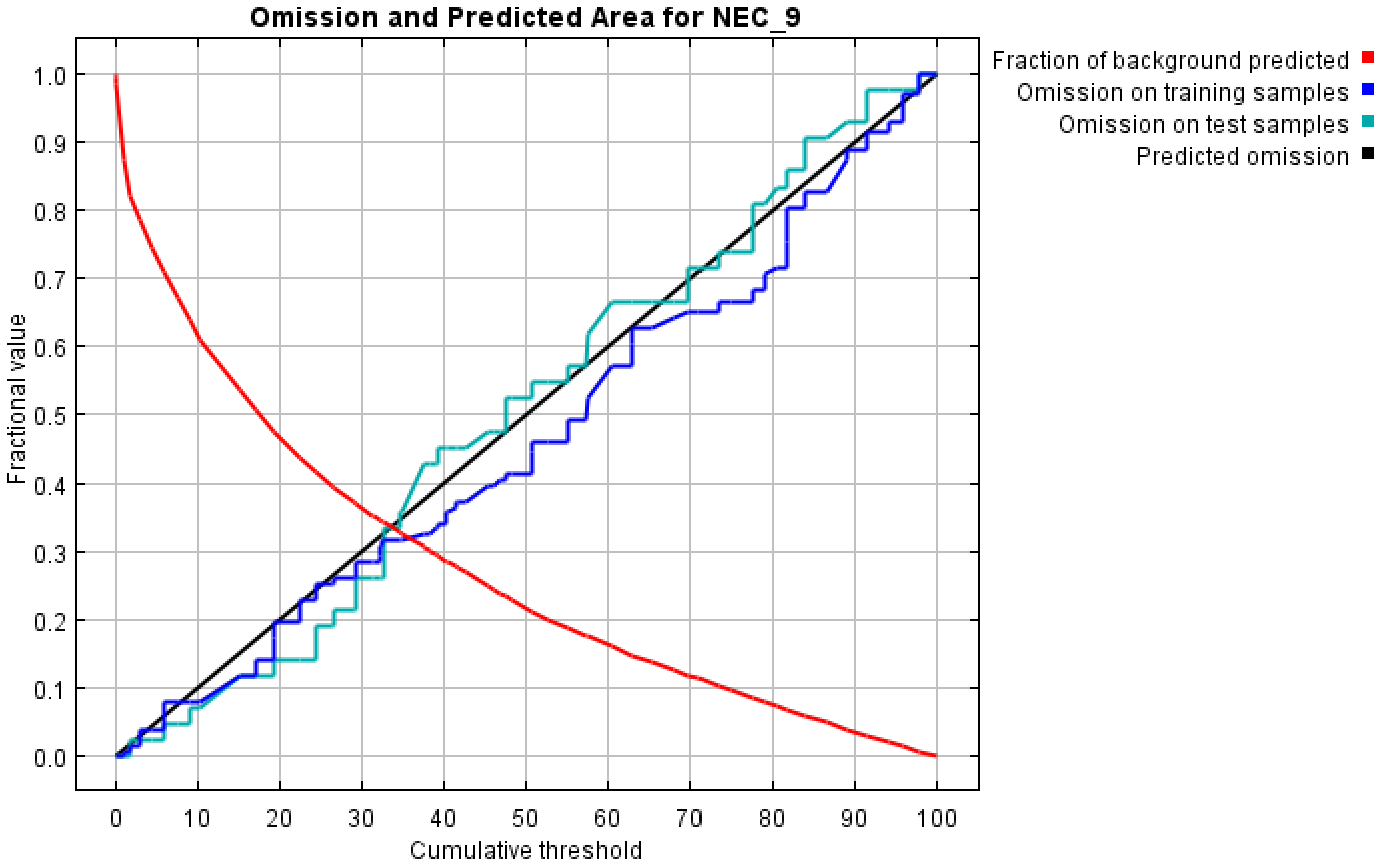

The next picture is the receiver operating characteristic (ROC) curve for the same data. Note that the specificity is defined using predicted area, rather than true commission (see the paper by Phillips, Anderson and Schapire cited on the help page for discussion of what this means). This implies that the maximum achievable AUC is less than 1. If test data is drawn from the Maxent distribution itself, then the maximum possible test AUC would be 0.723 rather than 1; in practice the test AUC may exceed this bound.

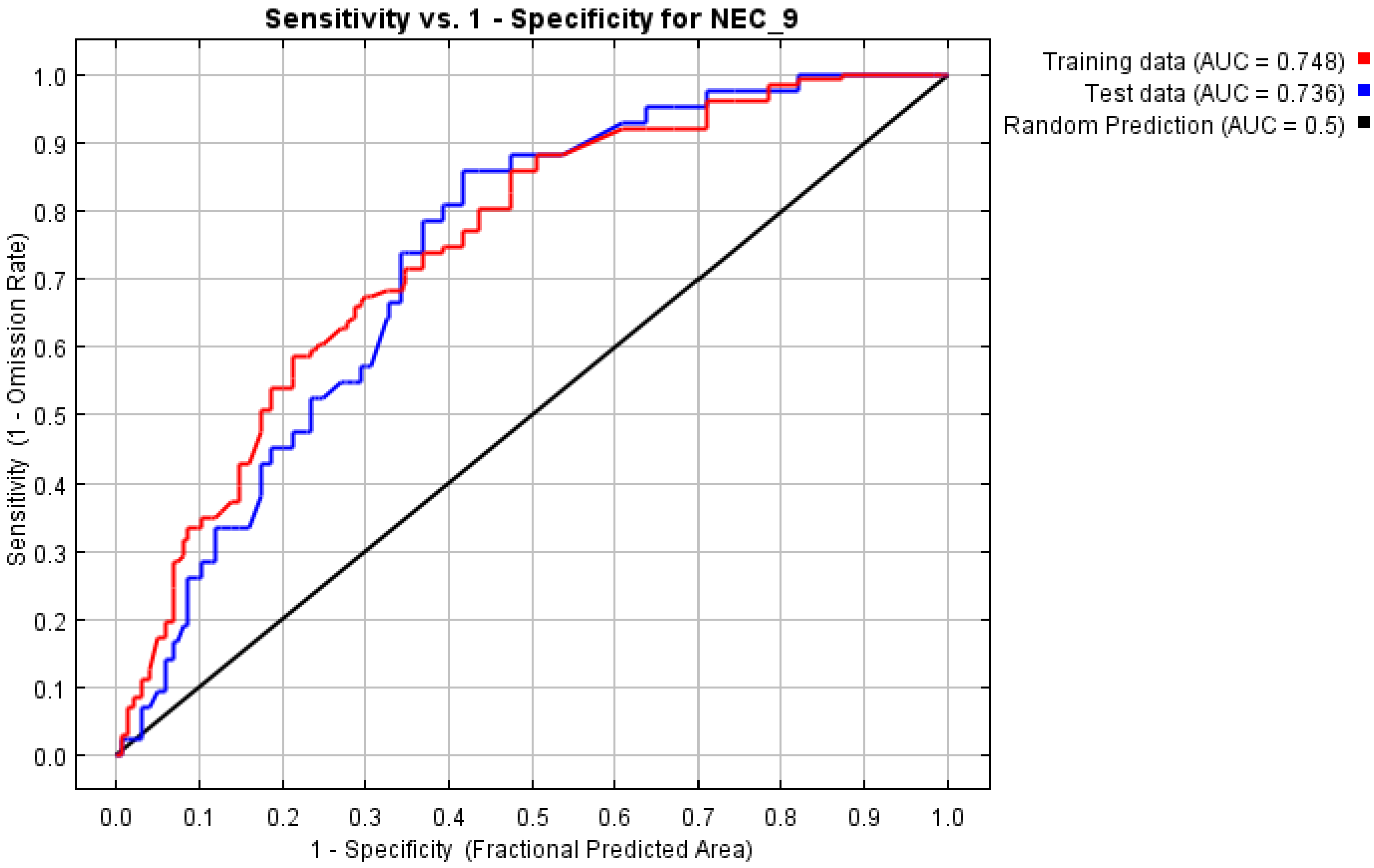

Some common thresholds and corresponding omission rates are as follows. If test data are available, binomial probabilities are calculated exactly if the number of test samples is at most 25, otherwise using a normal approximation to the binomial. These are 1-sided p-values for the null hypothesis that test points are predicted no better than by a random prediction with the same fractional predicted area. The "Balance" threshold minimizes 6 * training omission rate + .04 * cumulative threshold + 1.6 * fractional predicted area.

Cumulative threshold	Logistic threshold	Description	Fractional predicted area	Training omission rate	Test omission rate	P-value
1.000	0.058	Fixed cumulative value 1	0.874	0.000	0.000	6.999E-3
5.000	0.237	Fixed cumulative value 5	0.709	0.040	0.024	6.916E-5
10.000	0.317	Fixed cumulative value 10	0.609	0.079	0.071	1.119E-5
1.059	0.058	Minimum training presence	0.874	0.000	0.000	6.999E-3
10.352	0.317	10 percentile training presence	0.609	0.079	0.071	1.119E-5
34.812	0.492	Equal training sensitivity and specificity	0.327	0.317	0.357	6.4E-6
19.328	0.347	Maximum training sensitivity plus	0.476	0.143	0.119	7.194E-

		specificity				8
34.704	0.492	Equal test sensitivity and specificity	0.328	0.317	0.333	1.445E-6
24.388	0.419	Maximum test sensitivity plus specificity	0.416	0.230	0.143	3.34E-9
1.589	0.205	Balance training omission, predicted area and threshold value	0.820	0.008	0.000	1.189E-3
5.820	0.237	Equate entropy of thresholded and original distributions	0.709	0.040	0.024	6.916E-5

Click here to interactively explore this prediction using the Explain tool. If clicking from your browser does not succeed in starting the tool, try running the script in C:\Users\Bill\Desktop\Maxent Results NEC samples entire area\NEC_9_explain.bat directly. This tool requires the environmental grids to be small enough that they all fit in memory.

Response curves

These curves show how each environmental variable affects the Maxent prediction. The curves show how the logistic prediction changes as each environmental variable is varied, keeping all other environmental variables at their average sample value. Click on a response curve to see a larger version. Note that the curves can be hard to interpret if you have strongly correlated variables, as the model may depend on the correlations in ways that are not evident in the curves. In other words, the curves show the marginal effect of changing exactly one variable, whereas the model may take advantage of sets of variables changing together.

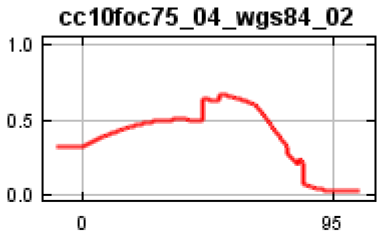

In contrast to the above marginal response curves, each of the following curves represents a different model, namely, a Maxent model created using only the corresponding variable. These plots reflect the dependence of predicted suitability both on the selected variable and on dependencies induced by correlations between the selected variable and other variables. They may be easier to interpret if there are strong correlations between variables.

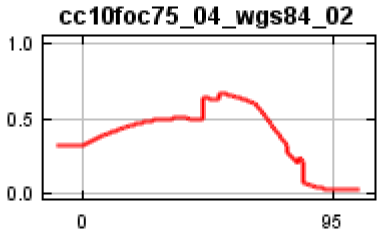

Analysis of variable contributions

The following table gives estimates of relative contributions of the environmental variables to the Maxent model. To determine the first estimate, in each iteration of the training algorithm, the increase in regularized gain is added to the contribution of the corresponding variable, or subtracted from it if the change to the absolute value of lambda is negative. For the second estimate, for each environmental variable in turn, the values of that variable on training presence and background data are randomly permuted. The model is reevaluated on the permuted data, and the resulting drop in training AUC is shown in the table, normalized to percentages. As with the variable jackknife, variable contributions should be interpreted with caution when the predictor variables are correlated.

Variable	Percent contribution	Permutation importance
cc10foc75_04_wgs84_02	100	100

Raw data outputs and control parameters

The data used in the above analysis is contained in the next links. Please see the Help button for more information on these.
The model applied to the training environmental layers
The coefficients of the model
The omission and predicted area for varying cumulative and raw thresholds
The prediction strength at the training and (optionally) test presence sites
Results for all species modeled in the same Maxent run, with summary statistics and (optionally) jackknife results

Regularized training gain is 0.332, training AUC is 0.748, unregularized training gain is 0.405.
Unregularized test gain is 0.399.
Test AUC is 0.736, standard deviation is 0.030 (calculated as in DeLong, DeLong & Clarke-Pearson 1988, equation 2).
Algorithm terminated after 500 iterations (1 seconds).

The follow settings were used during the run:
126 presence records used for training, 42 for testing.
10000 points used to determine the Maxent distribution (background points and presence points).
Environmental layers used (all continuous): cc10foc75_04_wgs84_02
Regularization values: linear/quadratic/product: 0.050, categorical: 0.250, threshold: 1.000, hinge: 0.500
Feature types used: hinge linear threshold quadratic
responsecurves: true
pictures: false
outputdirectory: C:\Users\Bill\Desktop\Maxent Results NEC samples entire area
samplesfile: C:\Users\Bill\Desktop\Maxent_samples\Compare_NEC_EC_150m_311_WGS_84_m.csv
environmentallayers: C:\Users\Bill\Desktop\Maxent Layers\maxent.cache
randomseed: true
randomtestpoints: 25
replicates: 10
replicatetype: subsample

Maxent model for NEC_0

This page contains some analysis of the Maxent model for NEC_0, created Sat Jul 11 19:51:49 EDT 2015 using Maxent version 3.3.3k. If you would like to do further analyses, the raw data used here is linked to at the end of this page.

Analysis of omission/commission

The following picture shows the omission rate and predicted area as a function of the cumulative threshold. The omission rate is is calculated both on the training presence records, and (if test data are used) on the test records. The omission rate should be close to the predicted omission, because of the definition of the cumulative threshold.

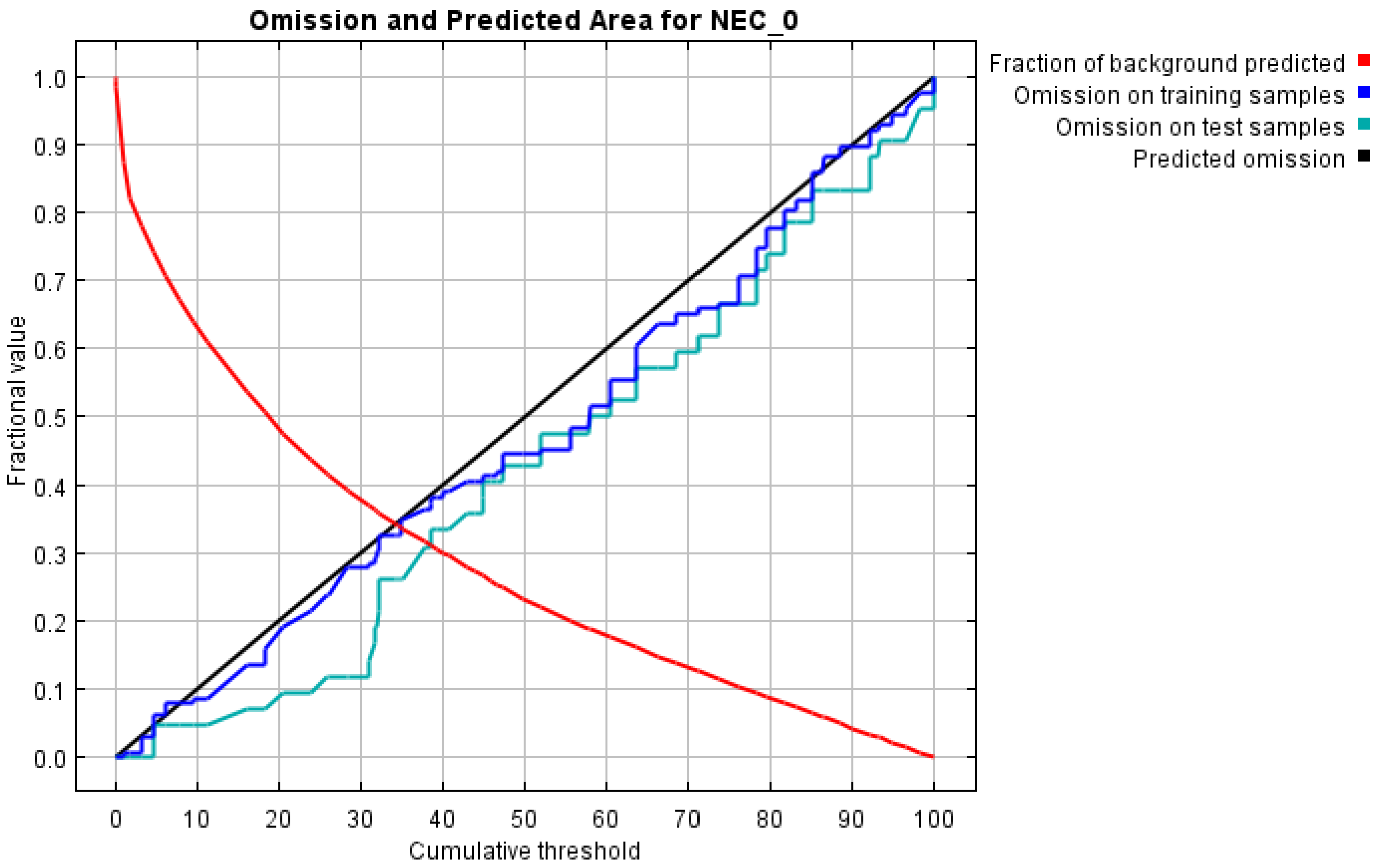

The next picture is the receiver operating characteristic (ROC) curve for the same data. Note that the specificity is defined using predicted area, rather than true commission (see the paper by Phillips, Anderson and Schapire cited on the help page for discussion of what this means). This implies that the maximum achievable AUC is less than 1. If test data is drawn from the Maxent distribution itself, then the maximum possible test AUC would be 0.711 rather than 1; in practice the test AUC may exceed this bound.

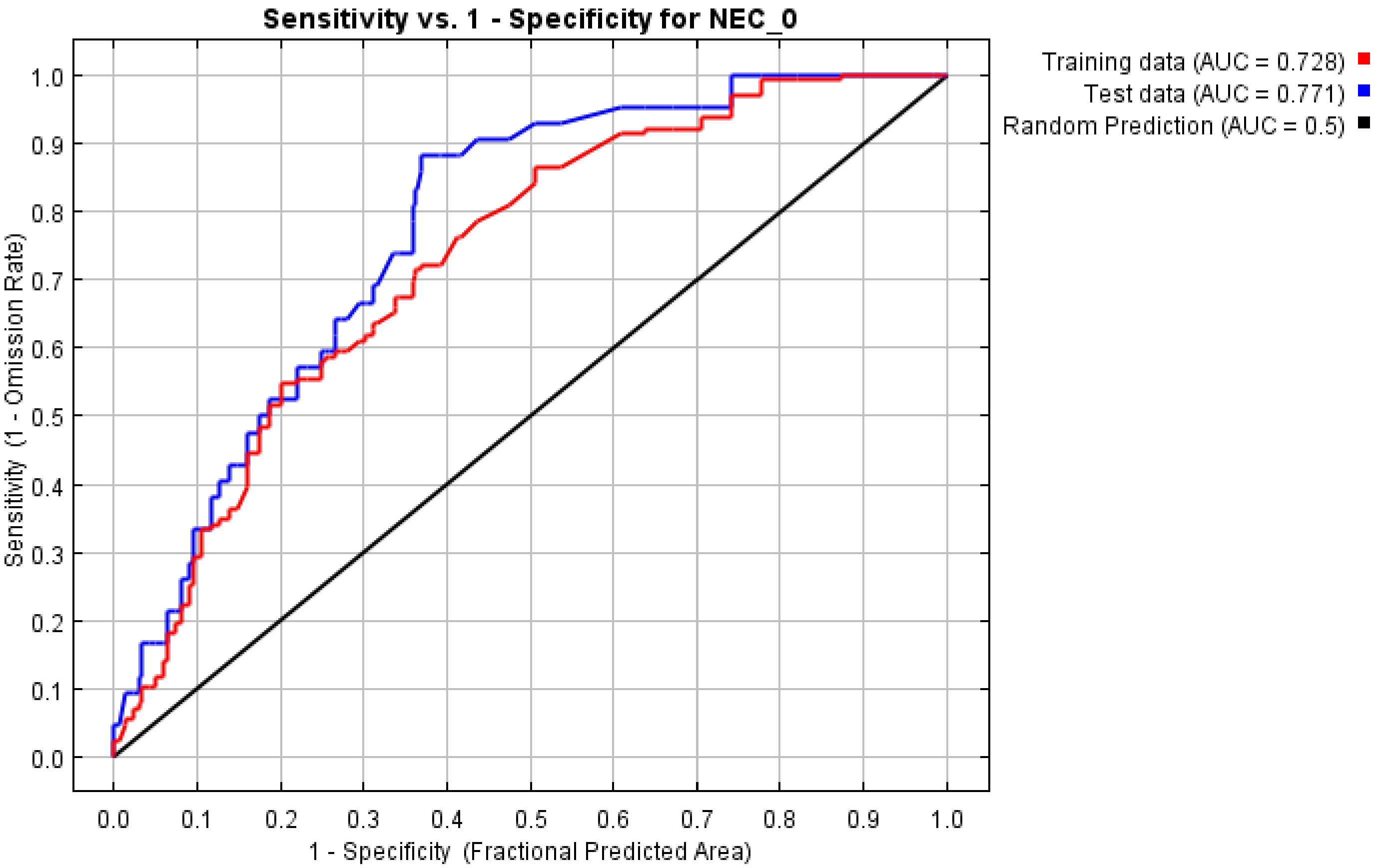

Some common thresholds and corresponding omission rates are as follows. If test data are available, binomial probabilities are calculated exactly if the number of test samples is at most 25, otherwise using a normal approximation to the binomial. These are 1-sided p-values for the null hypothesis that test points are predicted no better than by a random prediction with the same fractional predicted area. The "Balance" threshold minimizes 6 * training omission rate + .04 * cumulative threshold + 1.6 * fractional predicted area.

Cumulative threshold	Logistic threshold	Description	Fractional predicted area	Training omission rate	Test omission rate	P-value
1.000	0.060	Fixed cumulative value 1	0.874	0.000	0.000	6.999E-3
5.000	0.245	Fixed cumulative value 5	0.706	0.063	0.048	2.249E-4
10.000	0.332	Fixed cumulative value 10	0.609	0.087	0.048	2.608E-6
1.063	0.060	Minimum training presence	0.874	0.000	0.000	6.999E-3
11.258	0.332	10 percentile training presence	0.609	0.087	0.048	2.608E-6
34.930	0.497	Equal training sensitivity and specificity	0.338	0.341	0.262	2.046E-8
18.258	0.338	Maximum training sensitivity plus	0.506	0.135	0.071	2.172E-

		specificity				8
38.554	0.502	Equal test sensitivity and specificity	0.311	0.365	0.310	5.289E-8
30.995	0.460	Maximum test sensitivity plus specificity	0.369	0.278	0.119	3.044E-12
3.068	0.208	Balance training omission, predicted area and threshold value	0.778	0.008	0.000	2.682E-4
6.071	0.245	Equate entropy of thresholded and original distributions	0.706	0.063	0.048	2.249E-4

Click here to interactively explore this prediction using the Explain tool. If clicking from your browser does not succeed in starting the tool, try running the script in C:\Users\Bill\Desktop\Maxent Results NEC samples entire area\NEC_0_explain.bat directly. This tool requires the environmental grids to be small enough that they all fit in memory.

Response curves

These curves show how each environmental variable affects the Maxent prediction. The curves show how the logistic prediction changes as each environmental variable is varied, keeping all other environmental variables at their average sample value. Click on a response curve to see a larger version. Note that the curves can be hard to interpret if you have strongly correlated variables, as the model may depend on the correlations in ways that are not evident in the curves. In other words, the curves show the marginal effect of changing exactly one variable, whereas the model may take advantage of sets of variables changing together.

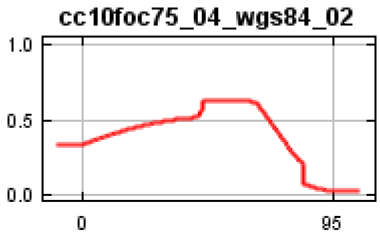

In contrast to the above marginal response curves, each of the following curves represents a different model, namely, a Maxent model created using only the corresponding variable. These plots reflect the dependence of predicted suitability both on the selected variable and on dependencies induced by correlations between the selected variable and other variables. They may be easier to interpret if there are strong correlations between variables.

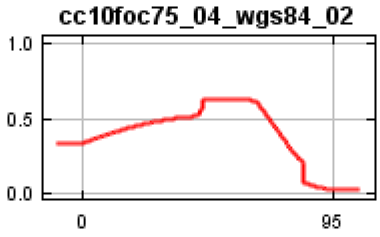

Analysis of variable contributions

The following table gives estimates of relative contributions of the environmental variables to the Maxent model. To determine the first estimate, in each iteration of the training algorithm, the increase in regularized gain is added to the contribution of the corresponding variable, or subtracted from it if the change to the absolute value of lambda is negative. For the second estimate, for each environmental variable in turn, the values of that variable on training presence and background data are randomly permuted. The model is reevaluated on the permuted data, and the resulting drop in training AUC is shown in the table, normalized to percentages. As with the variable jackknife, variable contributions should be interpreted with caution when the predictor variables are correlated.

Variable	Percent contribution	Permutation importance
cc10foc75_04_wgs84_02	100	100

Raw data outputs and control parameters

The data used in the above analysis is contained in the next links. Please see the Help button for more information on these.
The model applied to the training environmental layers
The coefficients of the model
The omission and predicted area for varying cumulative and raw thresholds
The prediction strength at the training and (optionally) test presence sites
Results for all species modeled in the same Maxent run, with summary statistics and (optionally) jackknife results

Regularized training gain is 0.301, training AUC is 0.728, unregularized training gain is 0.353.
Unregularized test gain is 0.472.
Test AUC is 0.771, standard deviation is 0.029 (calculated as in DeLong, DeLong & Clarke-Pearson 1988, equation 2).
Algorithm converged after 380 iterations (1 seconds).

The follow settings were used during the run:
126 presence records used for training, 42 for testing.
10000 points used to determine the Maxent distribution (background points and presence points).
Environmental layers used (all continuous): cc10foc75_04_wgs84_02
Regularization values: linear/quadratic/product: 0.050, categorical: 0.250, threshold: 1.000, hinge: 0.500
Feature types used: hinge linear threshold quadratic
responsecurves: true
pictures: false
outputdirectory: C:\Users\Bill\Desktop\Maxent Results NEC samples entire area
samplesfile: C:\Users\Bill\Desktop\Maxent_samples\Compare_NEC_EC_150m_311_WGS_84_m.csv
environmentallayers: C:\Users\Bill\Desktop\Maxent Layers\maxent.cache
randomseed: true
randomtestpoints: 25
replicates: 10
replicatetype: subsample
