# Supplementary material for: An Analysis of Overstory Tree Canopy Cover in Sites Occupied by Native and Introduced Cottontails in the Northeastern United States with Recommendations for Habitat Management for New England Cottontail
Source: PLoS One. 2015 Aug 12;10(8):e0135067. doi: 10.1371/journal.pone.0135067 (PMC4534376; doi:10.1371/journal.pone.0135067)
Supplement: S4 Model Outputs — (PDF) [file pone.0135067.s005.pdf]

Replicated maxent model for EC

This page summarizes the results of 10 split-sample models for EC, created Fri Jul 10 06:08:28 EDT 2015 using Maxent version 3.3.3k. The individual models are here: [0] [1] [2] [3] [4] [5] [6] [7] [8] [9]

Analysis of omission/commission

The following picture shows the test omission rate and predicted area as a function of the cumulative threshold, averaged over the replicate runs. The omission rate should be close to the predicted omission, because of the definition of the cumulative threshold.

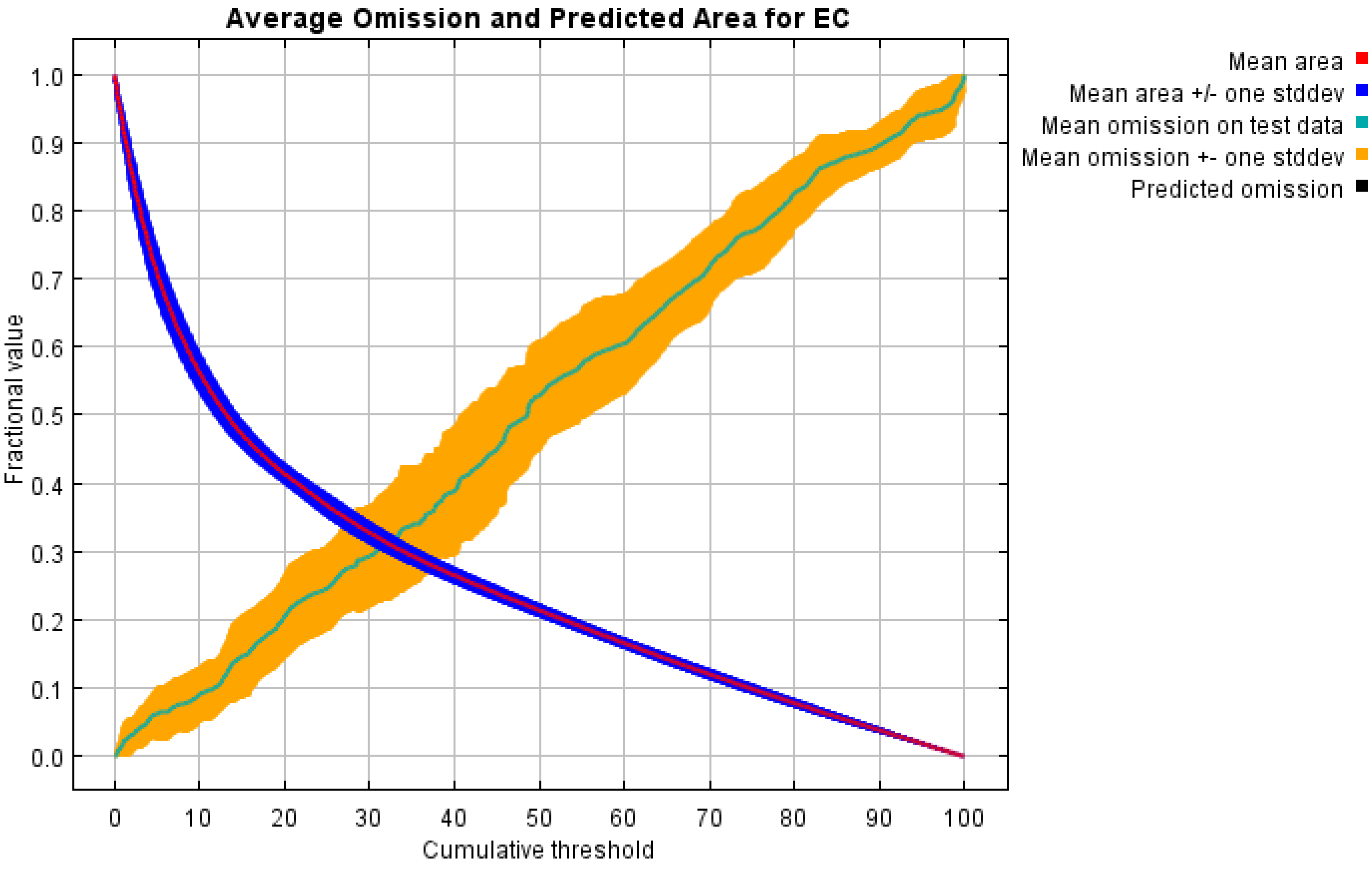

The next picture is the receiver operating characteristic (ROC) curve for the same data, again averaged over the replicate runs. Note that the specificity is defined using predicted area, rather than true commission (see the paper by Phillips, Anderson and Schapire cited on the help page for discussion of what this means). The average test AUC for the replicate runs is 0.727, and the standard deviation is 0.027.

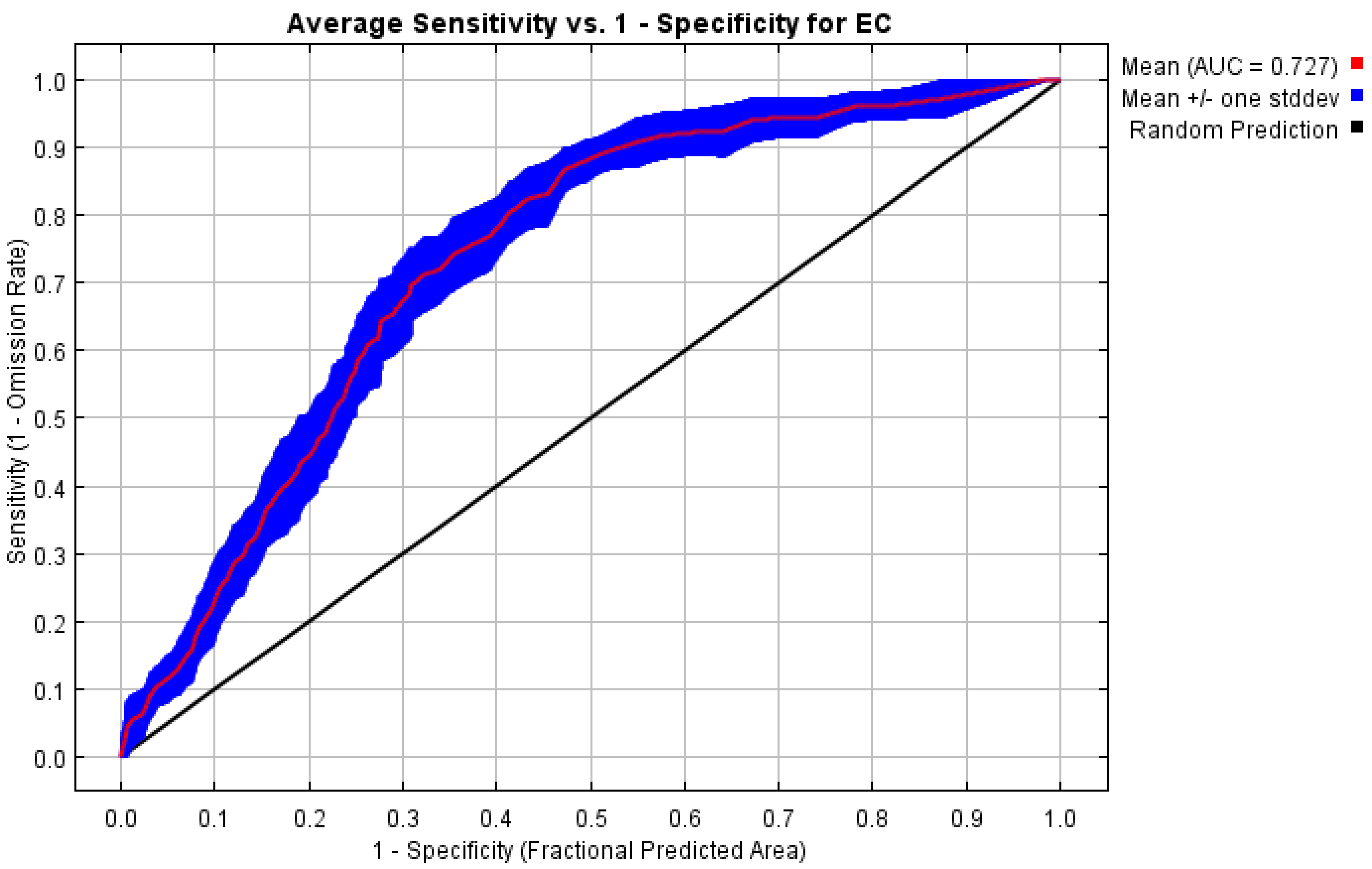

Response curves

These curves show how each environmental variable affects the Maxent prediction. The curves show how the logistic prediction changes as each environmental variable is varied, keeping all other environmental variables at their average sample value. Click on a response curve to see a larger version. Note that the curves can be hard to interpret if you have strongly correlated variables, as the model may depend on the correlations in ways that are not evident in the curves. In other words, the curves show the marginal effect of changing exactly one variable, whereas the model may take advantage of sets of variables changing together. The curves show the mean response of the 10 replicate Maxent runs (red) and and the mean +/- one standard deviation (blue, two shades for categorical variables).

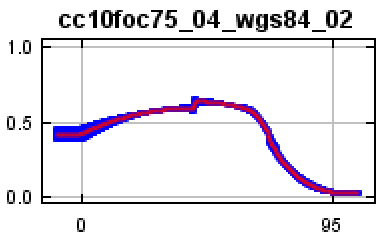

In contrast to the above marginal response curves, each of the following curves represents a different model, namely, a Maxent model created using only the corresponding variable. These plots reflect the dependence of predicted suitability both on the selected variable and on dependencies induced by correlations between the selected variable and other variables. They may be easier to interpret if there are strong correlations between variables.

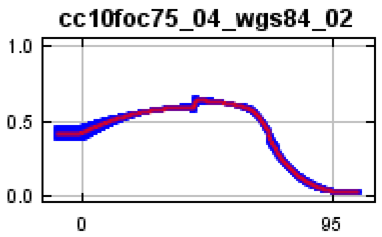

Analysis of variable contributions

The following table gives estimates of relative contributions of the environmental variables to the Maxent model. To determine the first estimate, in each iteration of the training algorithm, the increase in regularized gain is added to the contribution of the corresponding variable, or subtracted from it if the change to the absolute value of lambda is negative. For the second estimate, for each environmental variable in turn, the values of that variable on training presence and background data are randomly permuted. The model is reevaluated on the permuted data, and the resulting drop in training AUC is shown in the table, normalized to percentages. As with the variable jackknife, variable contributions should be interpreted with caution when the predictor variables are correlated. Values shown are averages over replicate runs.

Variable	Percent contribution	Permutation importance
cc10foc75_04_wgs84_02	100	100

Command line to repeat this species model: java density.MaxEnt nowarnings noprefixes -E "" -E EC responsecurves nopictures "outputdirectory=G:\Users\BB\Desktop\Maxent Results EC samples entire area" samplesfile=C:\Users\Bill\Desktop\Maxent_samples\Compare_NEC_EC_150m_311_WGS_84_m.csv "environmentallayers=C:\Users\Bill\Desktop\Maxent Layers\maxent.cache" randomseed randomtestpoints=25 replicates=10 replicatetype=subsample -N canopy_cover_within_75m_ec -N canopy_cover_within_75m_nec -N cc10foc75_02_buff3km_nec_01_wgs84_01 -N cc10foc75_04_buff3km_nec_20_wgs84_20 -N cc10mfoc75_04_buff3km_ec_20_wgs84_20_01 -N cc_10foc75_d_buff3km_nec_01_wgs84_10 -N cc_10mfoc75_d_buff3km_ec_01_wgs84_10 -N vpd_b_wgs_buff10_ec_01 -N vpd_b_wgs_buff50_ec_01 -N vpd_b_wgs_ec_01 -N vpd_b_wgs_nec_01

Maxent model for EC_0

This page contains some analysis of the Maxent model for EC_0, created Thu Jul 09 13:42:59 EDT 2015 using Maxent version 3.3.3k. If you would like to do further analyses, the raw data used here is linked to at the end of this page.

Analysis of omission/commission

The following picture shows the omission rate and predicted area as a function of the cumulative threshold. The omission rate is is calculated both on the training presence records, and (if test data are used) on the test records. The omission rate should be close to the predicted omission, because of the definition of the cumulative threshold.

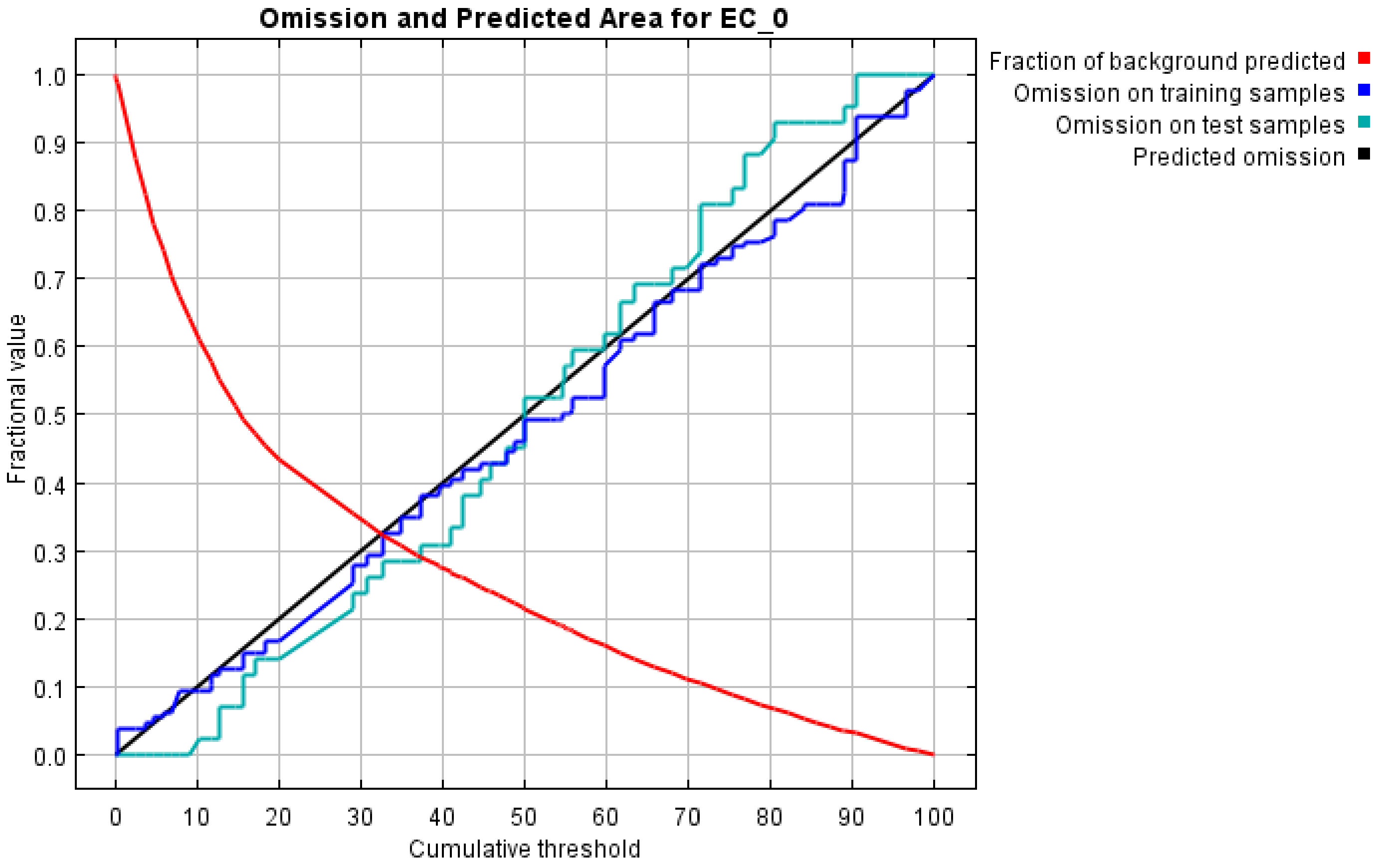

The next picture is the receiver operating characteristic (ROC) curve for the same data. Note that the specificity is defined using predicted area, rather than true commission (see the paper by Phillips, Anderson and Schapire cited on the help page for discussion of what this means). This implies that the maximum achievable AUC is less than 1. If test data is drawn from the Maxent distribution itself, then the maximum possible test AUC would be 0.728 rather than 1; in practice the test AUC may exceed this bound.

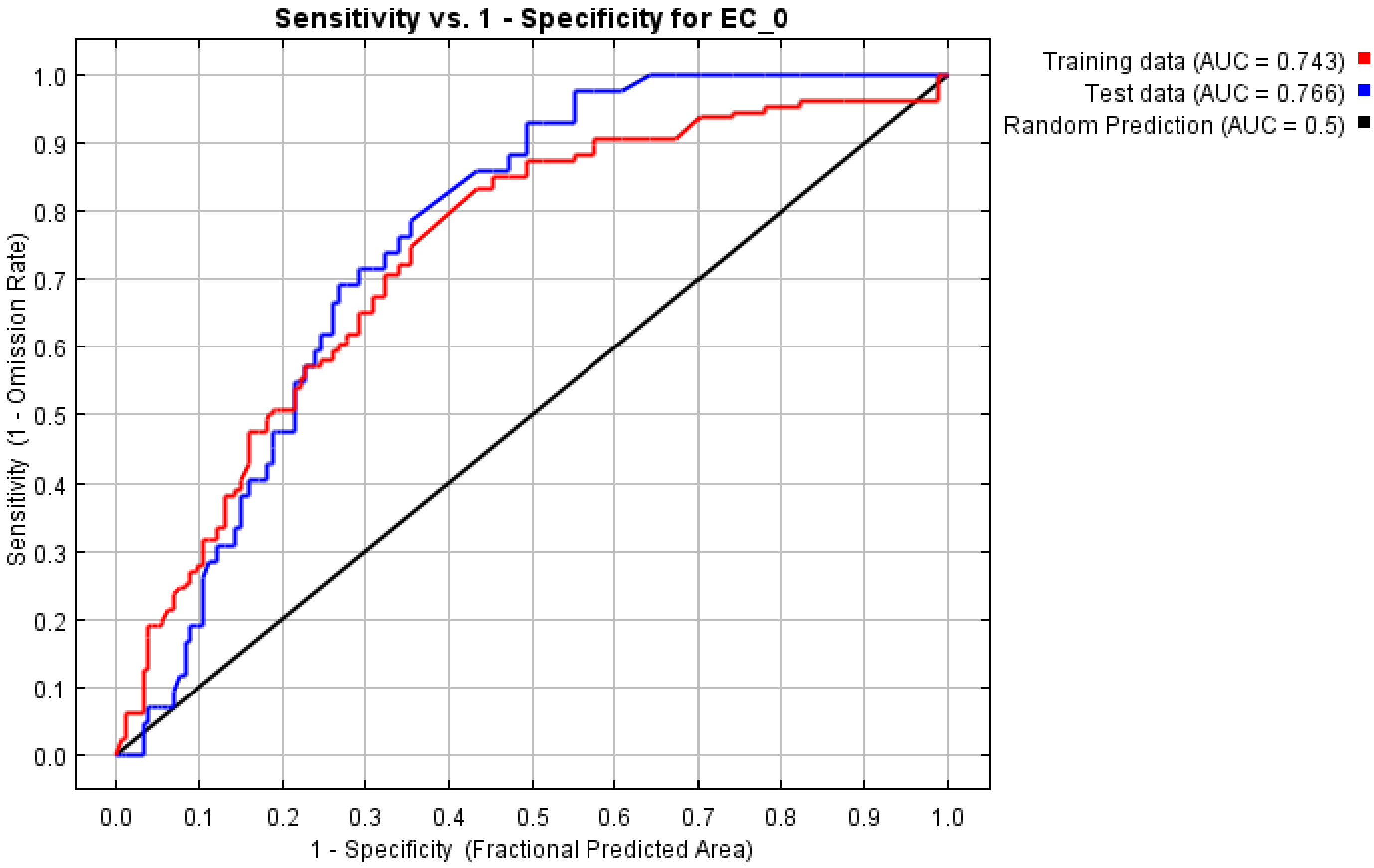

Some common thresholds and corresponding omission rates are as follows. If test data are available, binomial probabilities are calculated exactly if the number of test samples is at most 25, otherwise using a normal approximation to the binomial. These are 1-sided p-values for the null hypothesis that test points are predicted no better than by a random prediction with the same fractional predicted area. The "Balance" threshold minimizes 6 * training omission rate + .04 * cumulative threshold + 1.6 * fractional predicted area.

Cumulative threshold	Logistic threshold	Description	Fractional predicted area	Training omission rate	Test omission rate	P-value
1.000	0.138	Fixed cumulative value 1	0.877	0.040	0.000	7.517E-3
5.000	0.174	Fixed cumulative value 5	0.741	0.056	0.000	6.266E-5
10.000	0.229	Fixed cumulative value 10	0.610	0.095	0.024	5.674E-7
0.201	0.127	Minimum training presence	0.988	0.000	0.000	2.385E-1
11.608	0.229	10 percentile training presence	0.577	0.095	0.024	8.028E-8
32.740	0.496	Equal training sensitivity and specificity	0.323	0.325	0.286	2.934E-8
20.003	0.443	Maximum training sensitivity plus	0.434	0.167	0.143	1.523E-

		specificity				8
37.261	0.531	Equal test sensitivity and specificity	0.292	0.349	0.286	8.558E-10
15.675	0.306	Maximum test sensitivity plus specificity	0.494	0.127	0.071	8.783E-9
0.201	0.127	Balance training omission, predicted area and threshold value	0.988	0.000	0.000	2.385E-1
6.904	0.187	Equate entropy of thresholded and original distributions	0.704	0.063	0.000	1.321E-5

Click here to interactively explore this prediction using the Explain tool. If clicking from your browser does not succeed in starting the tool, try running the script in G:\Users\BB\Desktop\Maxent Results EC samples entire area\EC_0_explain.bat directly. This tool requires the environmental grids to be small enough that they all fit in memory.

Response curves

These curves show how each environmental variable affects the Maxent prediction. The curves show how the logistic prediction changes as each environmental variable is varied, keeping all other environmental variables at their average sample value. Click on a response curve to see a larger version. Note that the curves can be hard to interpret if you have strongly correlated variables, as the model may depend on the correlations in ways that are not evident in the curves. In other words, the curves show the marginal effect of changing exactly one variable, whereas the model may take advantage of sets of variables changing together.

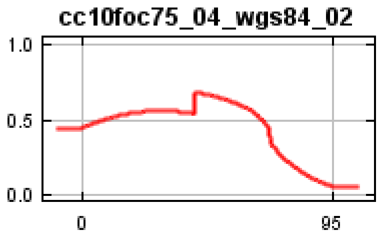

In contrast to the above marginal response curves, each of the following curves represents a different model, namely, a Maxent model created using only the corresponding variable. These plots reflect the dependence of predicted suitability both on the selected variable and on dependencies induced by correlations between the selected variable and other variables. They may be easier to interpret if there are strong correlations between variables.

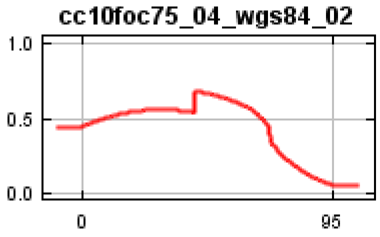

Analysis of variable contributions

The following table gives estimates of relative contributions of the environmental variables to the Maxent model. To determine the first estimate, in each iteration of the training algorithm, the increase in regularized gain is added to the contribution of the corresponding variable, or subtracted from it if the change to the absolute value of lambda is negative. For the second estimate, for each environmental variable in turn, the values of that variable on training presence and background data are randomly permuted. The model is reevaluated on the permuted data, and the resulting drop in training AUC is shown in the table, normalized to percentages. As with the variable jackknife, variable contributions should be interpreted with caution when the predictor variables are correlated.

Variable	Percent contribution	Permutation importance
cc10foc75_04_wgs84_02	100	100

Raw data outputs and control parameters

The data used in the above analysis is contained in the next links. Please see the Help button for more information on these.
The model applied to the training environmental layers
The coefficients of the model
The omission and predicted area for varying cumulative and raw thresholds
The prediction strength at the training and (optionally) test presence sites
Results for all species modeled in the same Maxent run, with summary statistics and (optionally) jackknife results

Regularized training gain is 0.340, training AUC is 0.743, unregularized training gain is 0.394.
Unregularized test gain is 0.456.
Test AUC is 0.766, standard deviation is 0.024 (calculated as in DeLong, DeLong & Clarke-Pearson 1988, equation 2).
Algorithm converged after 300 iterations (1 seconds).

The follow settings were used during the run:
126 presence records used for training, 42 for testing.
10000 points used to determine the Maxent distribution (background points and presence points).
Environmental layers used (all continuous): cc10foc75_04_wgs84_02
Regularization values: linear/quadratic/product: 0.050, categorical: 0.250, threshold: 1.000, hinge: 0.500
Feature types used: hinge linear threshold quadratic
responsecurves: true
pictures: false
outputdirectory: G:\Users\BB\Desktop\Maxent Results EC samples entire area
samplesfile: C:\Users\Bill\Desktop\Maxent_samples\Compare_NEC_EC_150m_311_WGS_84_m.csv
environmentallayers: C:\Users\Bill\Desktop\Maxent Layers\maxent.cache
randomseed: true
randomtestpoints: 25
replicates: 10
replicatetype: subsample

Maxent model for EC_1

This page contains some analysis of the Maxent model for EC_1, created Thu Jul 09 15:10:54 EDT 2015 using Maxent version 3.3.3k. If you would like to do further analyses, the raw data used here is linked to at the end of this page.

Analysis of omission/commission

The following picture shows the omission rate and predicted area as a function of the cumulative threshold. The omission rate is is calculated both on the training presence records, and (if test data are used) on the test records. The omission rate should be close to the predicted omission, because of the definition of the cumulative threshold.

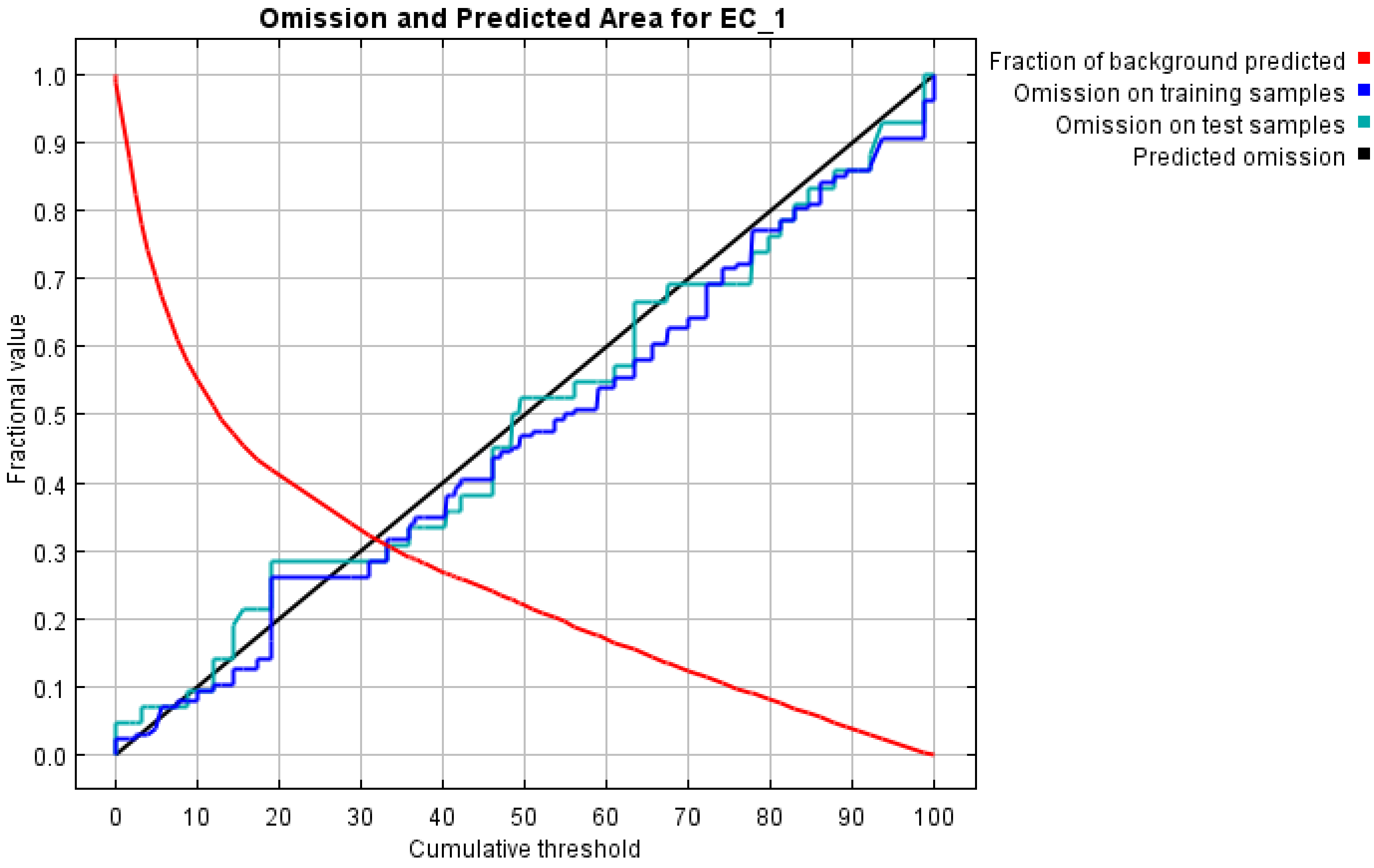

The next picture is the receiver operating characteristic (ROC) curve for the same data. Note that the specificity is defined using predicted area, rather than true commission (see the paper by Phillips, Anderson and Schapire cited on the help page for discussion of what this means). This implies that the maximum achievable AUC is less than 1. If test data is drawn from the Maxent distribution itself, then the maximum possible test AUC would be 0.737 rather than 1; in practice the test AUC may exceed this bound.

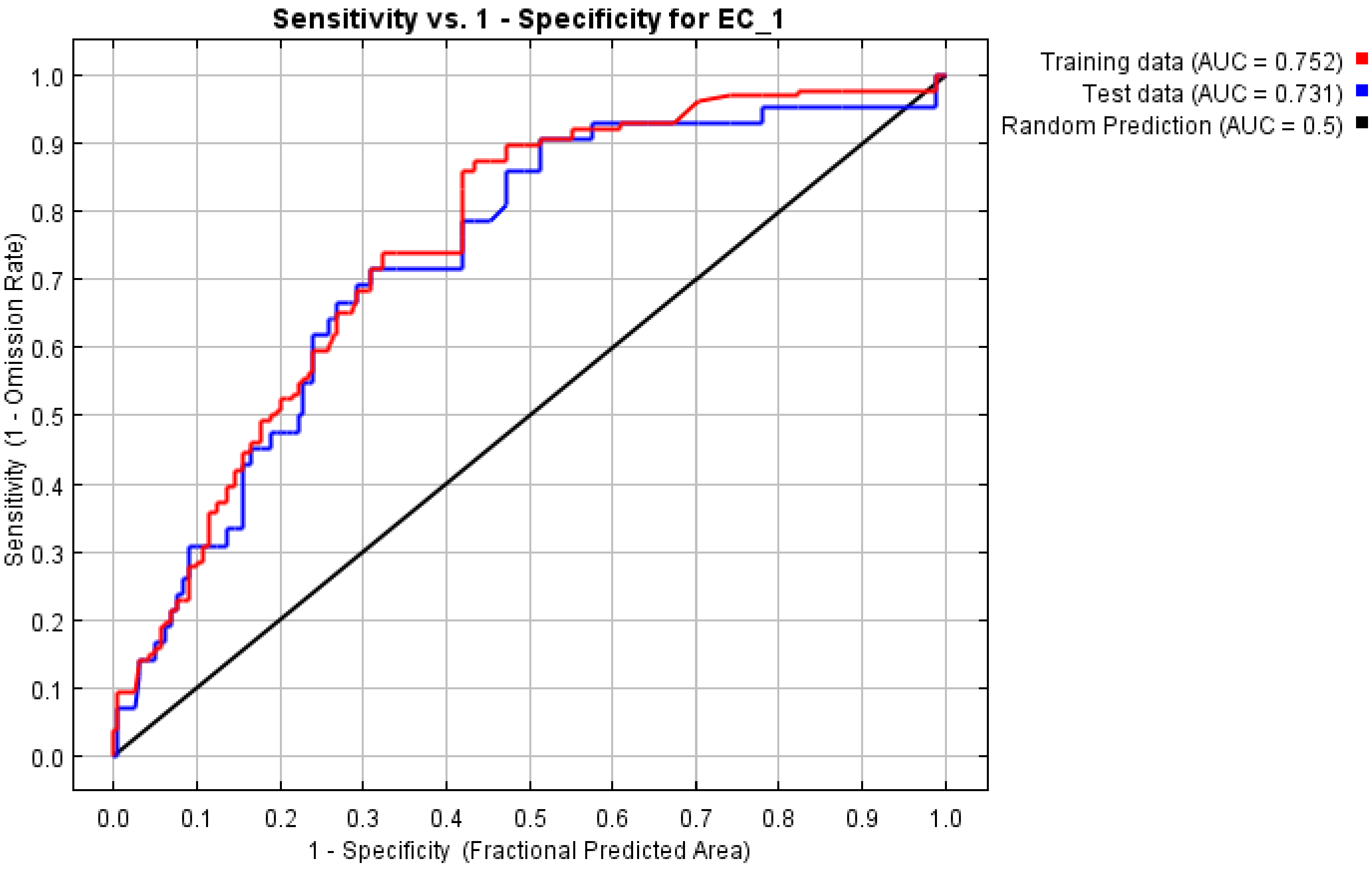

Some common thresholds and corresponding omission rates are as follows. If test data are available, binomial probabilities are calculated exactly if the number of test samples is at most 25, otherwise using a normal approximation to the binomial. These are 1-sided p-values for the null hypothesis that test points are predicted no better than by a random prediction with the same fractional predicted area. The "Balance" threshold minimizes 6 * training omission rate + .04 * cumulative threshold + 1.6 * fractional predicted area.

Cumulative threshold	Logistic threshold	Description	Fractional predicted area	Training omission rate	Test omission rate	P-value
1.000	0.095	Fixed cumulative value 1	0.877	0.024	0.048	6.769E-2
5.000	0.167	Fixed cumulative value 5	0.673	0.071	0.071	2.108E-4
10.000	0.251	Fixed cumulative value 10	0.513	0.095	0.095	1.892E-7
0.122	0.084	Minimum training presence	0.988	0.000	0.000	2.385E-1
11.850	0.251	10 percentile training presence	0.513	0.095	0.095	1.892E-7
33.195	0.531	Equal training sensitivity and specificity	0.308	0.317	0.310	3.998E-8
17.369	0.373	Maximum training sensitivity plus	0.434	0.127	0.214	2.065E-

		specificity				6
33.195	0.531	Equal test sensitivity and specificity	0.308	0.317	0.310	3.998E-8
33.178	0.504	Maximum test sensitivity plus specificity	0.308	0.286	0.286	5.941E-9
3.978	0.134	Balance training omission, predicted area and threshold value	0.741	0.032	0.071	2.724E-3
5.603	0.167	Equate entropy of thresholded and original distributions	0.673	0.071	0.071	2.108E-4

Click here to interactively explore this prediction using the Explain tool. If clicking from your browser does not succeed in starting the tool, try running the script in G:\Users\BB\Desktop\Maxent Results EC samples entire area\EC_1_explain.bat directly. This tool requires the environmental grids to be small enough that they all fit in memory.

Response curves

These curves show how each environmental variable affects the Maxent prediction. The curves show how the logistic prediction changes as each environmental variable is varied, keeping all other environmental variables at their average sample value. Click on a response curve to see a larger version. Note that the curves can be hard to interpret if you have strongly correlated variables, as the model may depend on the correlations in ways that are not evident in the curves. In other words, the curves show the marginal effect of changing exactly one variable, whereas the model may take advantage of sets of variables changing together.

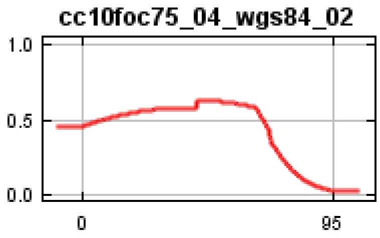

In contrast to the above marginal response curves, each of the following curves represents a different model, namely, a Maxent model created using only the corresponding variable. These plots reflect the dependence of predicted suitability both on the selected variable and on dependencies induced by correlations between the selected variable and other variables. They may be easier to interpret if there are strong correlations between variables.

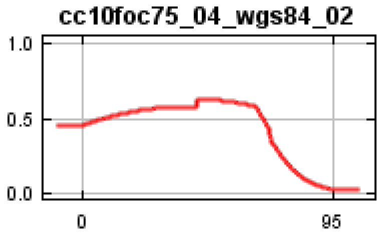

Analysis of variable contributions

The following table gives estimates of relative contributions of the environmental variables to the Maxent model. To determine the first estimate, in each iteration of the training algorithm, the increase in regularized gain is added to the contribution of the corresponding variable, or subtracted from it if the change to the absolute value of lambda is negative. For the second estimate, for each environmental variable in turn, the values of that variable on training presence and background data are randomly permuted. The model is reevaluated on the permuted data, and the resulting drop in training AUC is shown in the table, normalized to percentages. As with the variable jackknife, variable contributions should be interpreted with caution when the predictor variables are correlated.

Variable	Percent contribution	Permutation importance
cc10foc75_04_wgs84_02	100	100

Raw data outputs and control parameters

The data used in the above analysis is contained in the next links. Please see the Help button for more information on these.
The model applied to the training environmental layers
The coefficients of the model
The omission and predicted area for varying cumulative and raw thresholds
The prediction strength at the training and (optionally) test presence sites
Results for all species modeled in the same Maxent run, with summary statistics and (optionally) jackknife results

Regularized training gain is 0.383, training AUC is 0.752, unregularized training gain is 0.427.
Unregularized test gain is 0.346.
Test AUC is 0.731, standard deviation is 0.036 (calculated as in DeLong, DeLong & Clarke-Pearson 1988, equation 2).
Algorithm converged after 360 iterations (1 seconds).

The follow settings were used during the run:
126 presence records used for training, 42 for testing.
10000 points used to determine the Maxent distribution (background points and presence points).
Environmental layers used (all continuous): cc10foc75_04_wgs84_02
Regularization values: linear/quadratic/product: 0.050, categorical: 0.250, threshold: 1.000, hinge: 0.500
Feature types used: hinge linear threshold quadratic
responsecurves: true
pictures: false
outputdirectory: G:\Users\BB\Desktop\Maxent Results EC samples entire area
samplesfile: C:\Users\Bill\Desktop\Maxent_samples\Compare_NEC_EC_150m_311_WGS_84_m.csv
environmentallayers: C:\Users\Bill\Desktop\Maxent Layers\maxent.cache
randomseed: true
randomtestpoints: 25
replicates: 10
replicatetype: subsample

Maxent model for EC_2

This page contains some analysis of the Maxent model for EC_2, created Thu Jul 09 16:35:49 EDT 2015 using Maxent version 3.3.3k. If you would like to do further analyses, the raw data used here is linked to at the end of this page.

Analysis of omission/commission

The following picture shows the omission rate and predicted area as a function of the cumulative threshold. The omission rate is is calculated both on the training presence records, and (if test data are used) on the test records. The omission rate should be close to the predicted omission, because of the definition of the cumulative threshold.

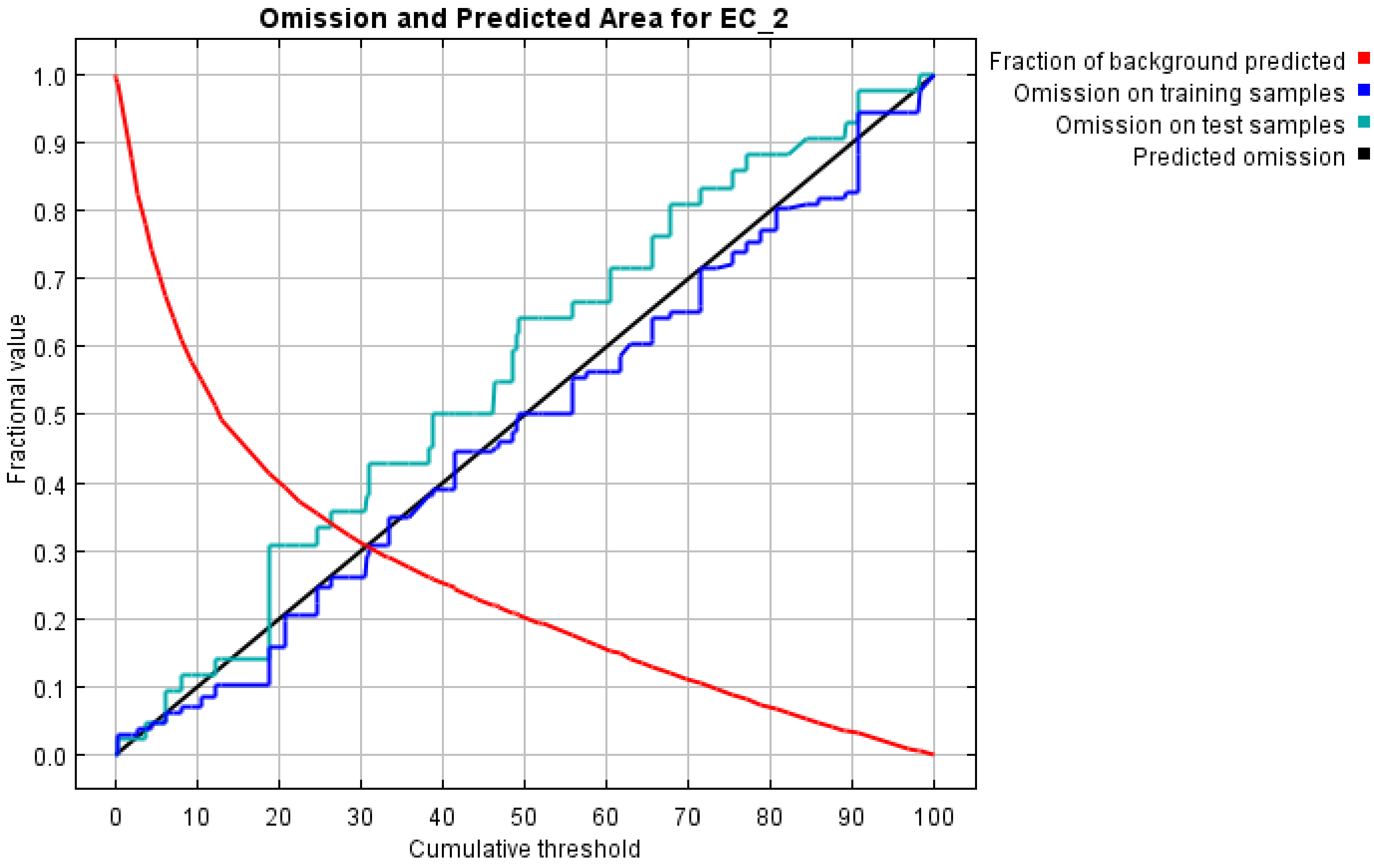

The next picture is the receiver operating characteristic (ROC) curve for the same data. Note that the specificity is defined using predicted area, rather than true commission (see the paper by Phillips, Anderson and Schapire cited on the help page for discussion of what this means). This implies that the maximum achievable AUC is less than 1. If test data is drawn from the Maxent distribution itself, then the maximum possible test AUC would be 0.746 rather than 1; in practice the test AUC may exceed this bound.

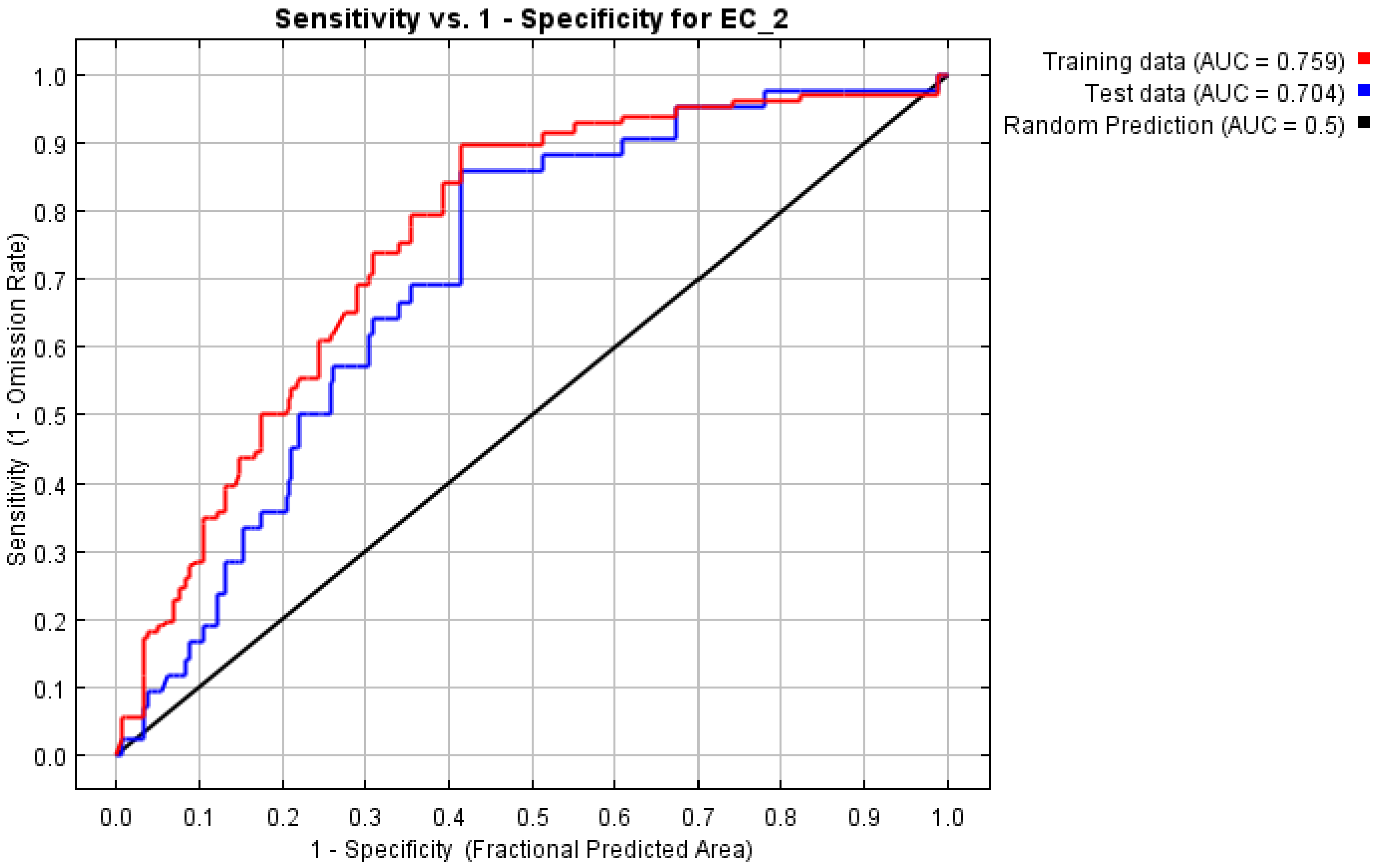

Some common thresholds and corresponding omission rates are as follows. If test data are available, binomial probabilities are calculated exactly if the number of test samples is at most 25, otherwise using a normal approximation to the binomial. These are 1-sided p-values for the null hypothesis that test points are predicted no better than by a random prediction with the same fractional predicted area. The "Balance" threshold minimizes 6 * training omission rate + .04 * cumulative threshold + 1.6 * fractional predicted area.

Cumulative threshold	Logistic threshold	Description	Fractional predicted area	Training omission rate	Test omission rate	P-value
1.000	0.103	Fixed cumulative value 1	0.877	0.032	0.024	2.486E-2
5.000	0.151	Fixed cumulative value 5	0.704	0.048	0.048	2.108E-4
10.000	0.213	Fixed cumulative value 10	0.551	0.071	0.119	8.481E-6
0.143	0.093	Minimum training presence	0.988	0.000	0.000	2.385E-1
12.082	0.231	10 percentile training presence	0.513	0.087	0.119	9.174E-7
31.082	0.509	Equal training sensitivity and specificity	0.305	0.302	0.381	4.881E-6
18.771	0.325	Maximum training sensitivity plus	0.414	0.103	0.143	2.811E-

		specificity				9
26.406	0.464	Equal test sensitivity and specificity	0.339	0.246	0.333	3.687E-6
18.771	0.325	Maximum test sensitivity plus specificity	0.414	0.103	0.143	2.811E-9
0.143	0.093	Balance training omission, predicted area and threshold value	0.988	0.000	0.000	2.385E-1
7.049	0.181	Equate entropy of thresholded and original distributions	0.642	0.063	0.095	1.944E-4

Click here to interactively explore this prediction using the Explain tool. If clicking from your browser does not succeed in starting the tool, try running the script in G:\Users\BB\Desktop\Maxent Results EC samples entire area\EC_2_explain.bat directly. This tool requires the environmental grids to be small enough that they all fit in memory.

Response curves

These curves show how each environmental variable affects the Maxent prediction. The curves show how the logistic prediction changes as each environmental variable is varied, keeping all other environmental variables at their average sample value. Click on a response curve to see a larger version. Note that the curves can be hard to interpret if you have strongly correlated variables, as the model may depend on the correlations in ways that are not evident in the curves. In other words, the curves show the marginal effect of changing exactly one variable, whereas the model may take advantage of sets of variables changing together.

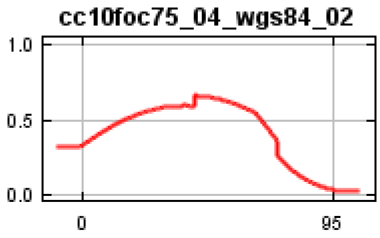

In contrast to the above marginal response curves, each of the following curves represents a different model, namely, a Maxent model created using only the corresponding variable. These plots reflect the dependence of predicted suitability both on the selected variable and on dependencies induced by correlations between the selected variable and other variables. They may be easier to interpret if there are strong correlations between variables.

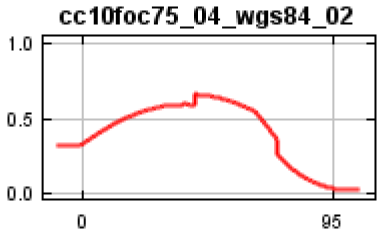

Analysis of variable contributions

The following table gives estimates of relative contributions of the environmental variables to the Maxent model. To determine the first estimate, in each iteration of the training algorithm, the increase in regularized gain is added to the contribution of the corresponding variable, or subtracted from it if the change to the absolute value of lambda is negative. For the second estimate, for each environmental variable in turn, the values of that variable on training presence and background data are randomly permuted. The model is reevaluated on the permuted data, and the resulting drop in training AUC is shown in the table, normalized to percentages. As with the variable jackknife, variable contributions should be interpreted with caution when the predictor variables are correlated.

Variable	Percent contribution	Permutation importance
cc10foc75_04_wgs84_02	100	100

Raw data outputs and control parameters

The data used in the above analysis is contained in the next links. Please see the Help button for more information on these.
The model applied to the training environmental layers
The coefficients of the model
The omission and predicted area for varying cumulative and raw thresholds
The prediction strength at the training and (optionally) test presence sites
Results for all species modeled in the same Maxent run, with summary statistics and (optionally) jackknife results

Regularized training gain is 0.405, training AUC is 0.759, unregularized training gain is 0.451.
Unregularized test gain is 0.267.
Test AUC is 0.704, standard deviation is 0.033 (calculated as in DeLong, DeLong & Clarke-Pearson 1988, equation 2).
Algorithm terminated after 500 iterations (1 seconds).

The follow settings were used during the run:
126 presence records used for training, 42 for testing.
10000 points used to determine the Maxent distribution (background points and presence points).
Environmental layers used (all continuous): cc10foc75_04_wgs84_02
Regularization values: linear/quadratic/product: 0.050, categorical: 0.250, threshold: 1.000, hinge: 0.500
Feature types used: hinge linear threshold quadratic
responsecurves: true
pictures: false
outputdirectory: G:\Users\BB\Desktop\Maxent Results EC samples entire area
samplesfile: C:\Users\Bill\Desktop\Maxent_samples\Compare_NEC_EC_150m_311_WGS_84_m.csv
environmentallayers: C:\Users\Bill\Desktop\Maxent Layers\maxent.cache
randomseed: true
randomtestpoints: 25
replicates: 10
replicatetype: subsample

Maxent model for EC_3

This page contains some analysis of the Maxent model for EC_3, created Thu Jul 09 18:01:18 EDT 2015 using Maxent version 3.3.3k. If you would like to do further analyses, the raw data used here is linked to at the end of this page.

Analysis of omission/commission

The following picture shows the omission rate and predicted area as a function of the cumulative threshold. The omission rate is is calculated both on the training presence records, and (if test data are used) on the test records. The omission rate should be close to the predicted omission, because of the definition of the cumulative threshold.

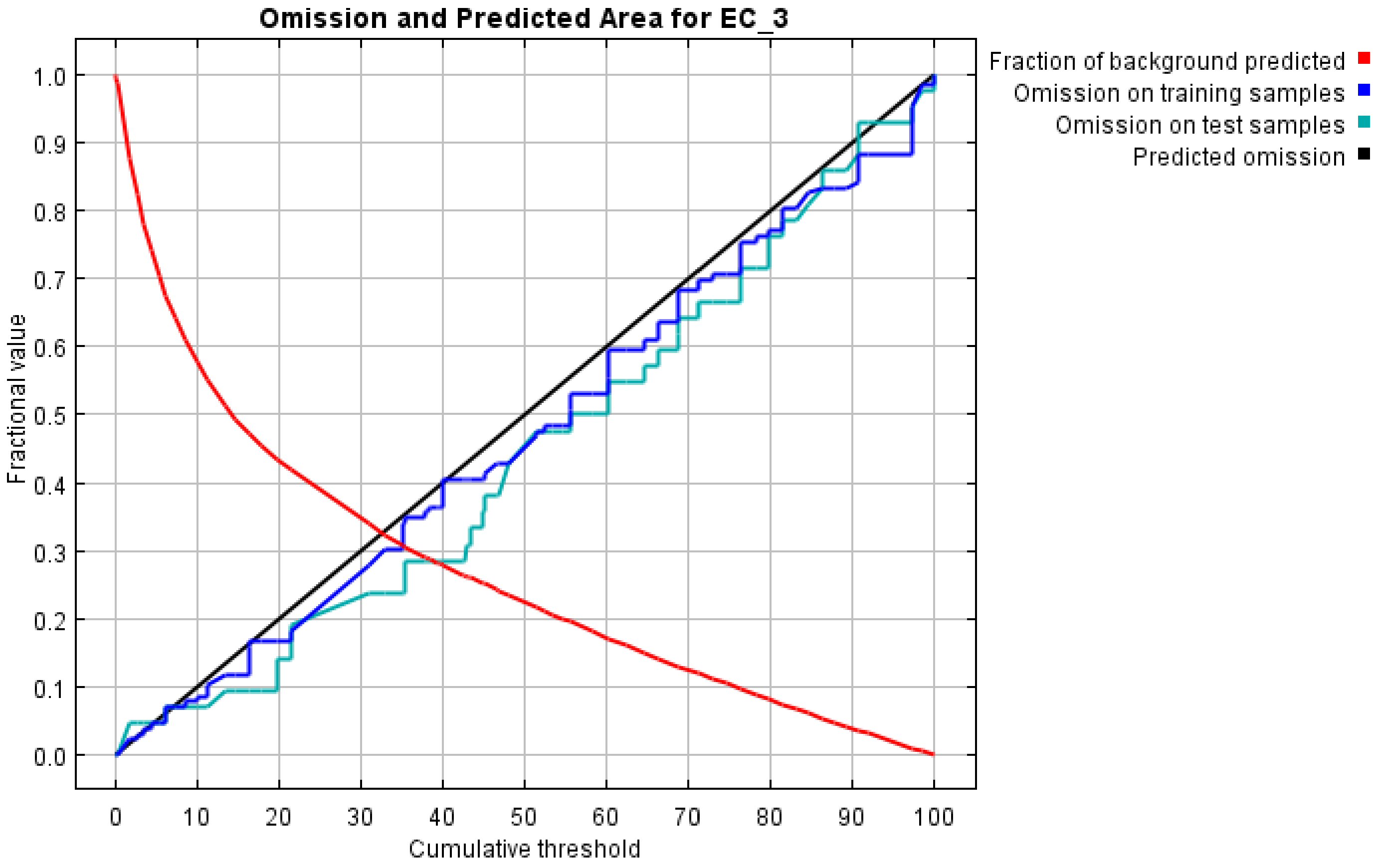

The next picture is the receiver operating characteristic (ROC) curve for the same data. Note that the specificity is defined using predicted area, rather than true commission (see the paper by Phillips, Anderson and Schapire cited on the help page for discussion of what this means). This implies that the maximum achievable AUC is less than 1. If test data is drawn from the Maxent distribution itself, then the maximum possible test AUC would be 0.728 rather than 1; in practice the test AUC may exceed this bound.

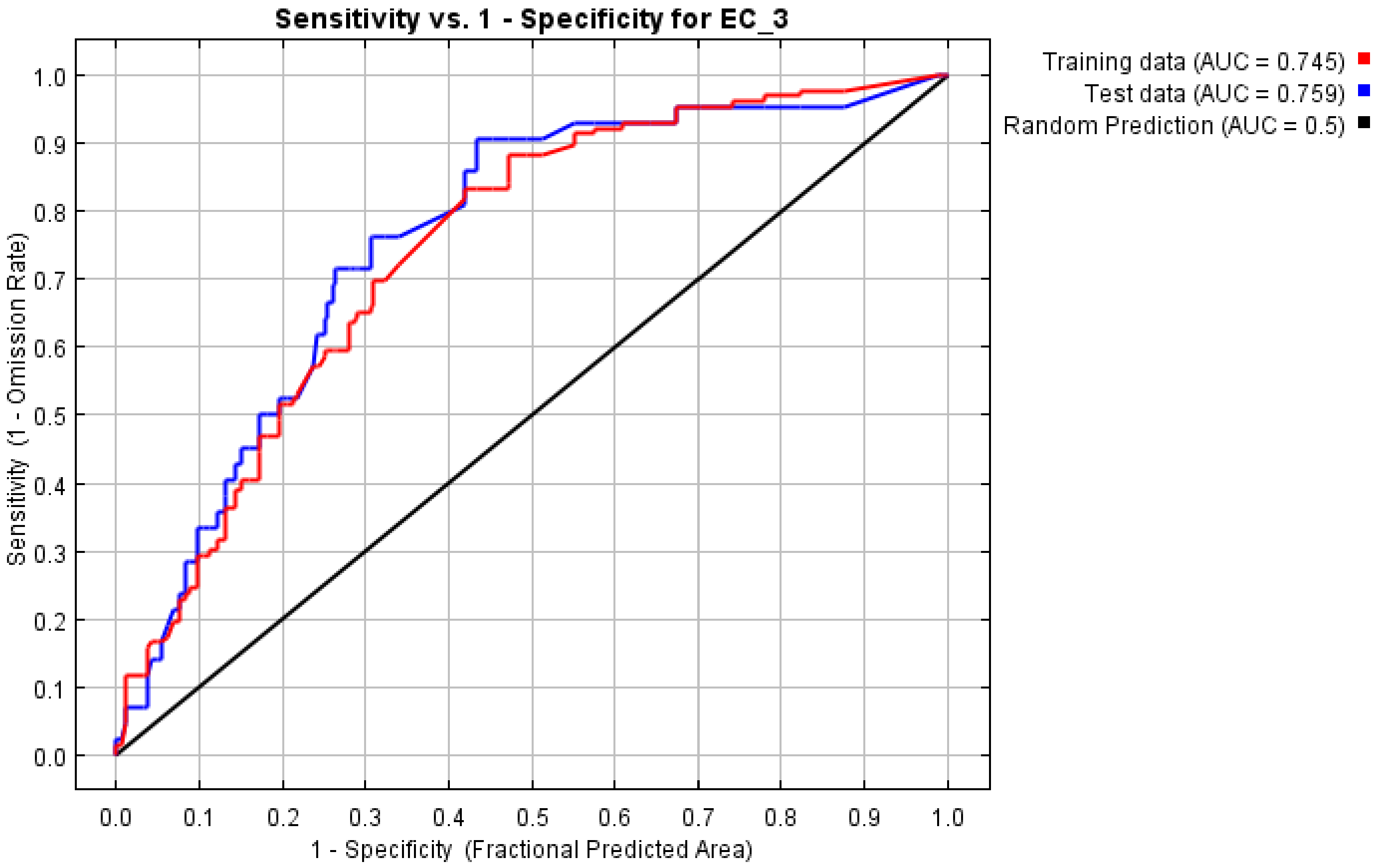

Some common thresholds and corresponding omission rates are as follows. If test data are available, binomial probabilities are calculated exactly if the number of test samples is at most 25, otherwise using a normal approximation to the binomial. These are 1-sided p-values for the null hypothesis that test points are predicted no better than by a random prediction with the same fractional predicted area. The "Balance" threshold minimizes 6 * training omission rate + .04 * cumulative threshold + 1.6 * fractional predicted area.

Cumulative threshold	Logistic threshold	Description	Fractional predicted area	Training omission rate	Test omission rate	P-value
1.000	0.106	Fixed cumulative value 1	0.877	0.024	0.048	6.769E-2
5.000	0.171	Fixed cumulative value 5	0.704	0.048	0.048	2.108E-4
10.000	0.261	Fixed cumulative value 10	0.551	0.087	0.071	4.284E-7
0.132	0.094	Minimum training presence	0.988	0.000	0.000	2.385E-1
11.242	0.261	10 percentile training presence	0.551	0.087	0.071	4.284E-7
35.108	0.501	Equal training sensitivity and specificity	0.308	0.302	0.238	9.463E-11
21.401	0.437	Maximum training sensitivity plus	0.419	0.167	0.143	4.294E-

		specificity				9
38.568	0.558	Equal test sensitivity and specificity	0.287	0.365	0.286	4.696E-10
19.782	0.406	Maximum test sensitivity plus specificity	0.434	0.167	0.095	3.602E-10
2.667	0.120	Balance training omission, predicted area and threshold value	0.823	0.024	0.048	1.419E-2
5.318	0.171	Equate entropy of thresholded and original distributions	0.704	0.048	0.048	2.108E-4

Click here to interactively explore this prediction using the Explain tool. If clicking from your browser does not succeed in starting the tool, try running the script in G:\Users\BB\Desktop\Maxent Results EC samples entire area\EC_3_explain.bat directly. This tool requires the environmental grids to be small enough that they all fit in memory.

Response curves

These curves show how each environmental variable affects the Maxent prediction. The curves show how the logistic prediction changes as each environmental variable is varied, keeping all other environmental variables at their average sample value. Click on a response curve to see a larger version. Note that the curves can be hard to interpret if you have strongly correlated variables, as the model may depend on the correlations in ways that are not evident in the curves. In other words, the curves show the marginal effect of changing exactly one variable, whereas the model may take advantage of sets of variables changing together.

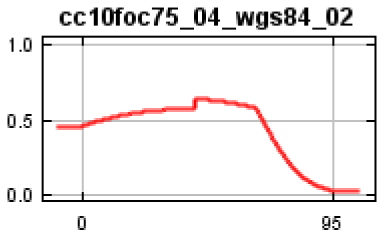

In contrast to the above marginal response curves, each of the following curves represents a different model, namely, a Maxent model created using only the corresponding variable. These plots reflect the dependence of predicted suitability both on the selected variable and on dependencies induced by correlations between the selected variable and other variables. They may be easier to interpret if there are strong correlations between variables.

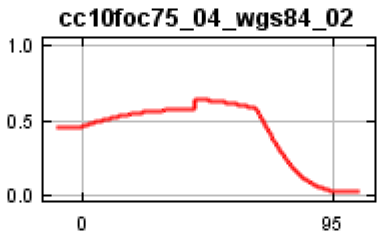

Analysis of variable contributions

The following table gives estimates of relative contributions of the environmental variables to the Maxent model. To determine the first estimate, in each iteration of the training algorithm, the increase in regularized gain is added to the contribution of the corresponding variable, or subtracted from it if the change to the absolute value of lambda is negative. For the second estimate, for each environmental variable in turn, the values of that variable on training presence and background data are randomly permuted. The model is reevaluated on the permuted data, and the resulting drop in training AUC is shown in the table, normalized to percentages. As with the variable jackknife, variable contributions should be interpreted with caution when the predictor variables are correlated.

Variable	Percent contribution	Permutation importance
cc10foc75_04_wgs84_02	100	100

Raw data outputs and control parameters

The data used in the above analysis is contained in the next links. Please see the Help button for more information on these.
[The model applied to the training environmental layers]
[The coefficients of the model]
[The omission and predicted area for varying cumulative and raw thresholds]
[The prediction strength at the training and (optionally) test presence sites]
[Results for all species modeled in the same Maxent run, with summary statistics and (optionally) jackknife results]

Regularized training gain is 0.348, training AUC is 0.745, unregularized training gain is 0.388.
Unregularized test gain is 0.454.
Test AUC is 0.759, standard deviation is 0.034 (calculated as in DeLong, DeLong & Clarke-Pearson 1988, equation 2).
Algorithm converged after 320 iterations (1 seconds).

The follow settings were used during the run:
126 presence records used for training, 42 for testing.
10000 points used to determine the Maxent distribution (background points and presence points).
Environmental layers used (all continuous): cc10foc75_04_wgs84_02
Regularization values: linear/quadratic/product: 0.050, categorical: 0.250, threshold: 1.000, hinge: 0.500
Feature types used: hinge linear threshold quadratic
responsecurves: true
pictures: false
outputdirectory: G:\Users\BB\Desktop\Maxent Results EC samples entire area
samplesfile: C:\Users\Bill\Desktop\Maxent_samples\Compare_NEC_EC_150m_311_WGS_84_m.csv
environmentallayers: C:\Users\Bill\Desktop\Maxent Layers\maxent.cache
randomseed: true
randomtestpoints: 25
replicates: 10
replicatetype: subsample

Maxent model for EC_4

This page contains some analysis of the Maxent model for EC_4, created Thu Jul 09 19:38:00 EDT 2015 using Maxent version 3.3.3k. If you would like to do further analyses, the raw data used here is linked to at the end of this page.

Analysis of omission/commission

The following picture shows the omission rate and predicted area as a function of the cumulative threshold. The omission rate is is calculated both on the training presence records, and (if test data are used) on the test records. The omission rate should be close to the predicted omission, because of the definition of the cumulative threshold.

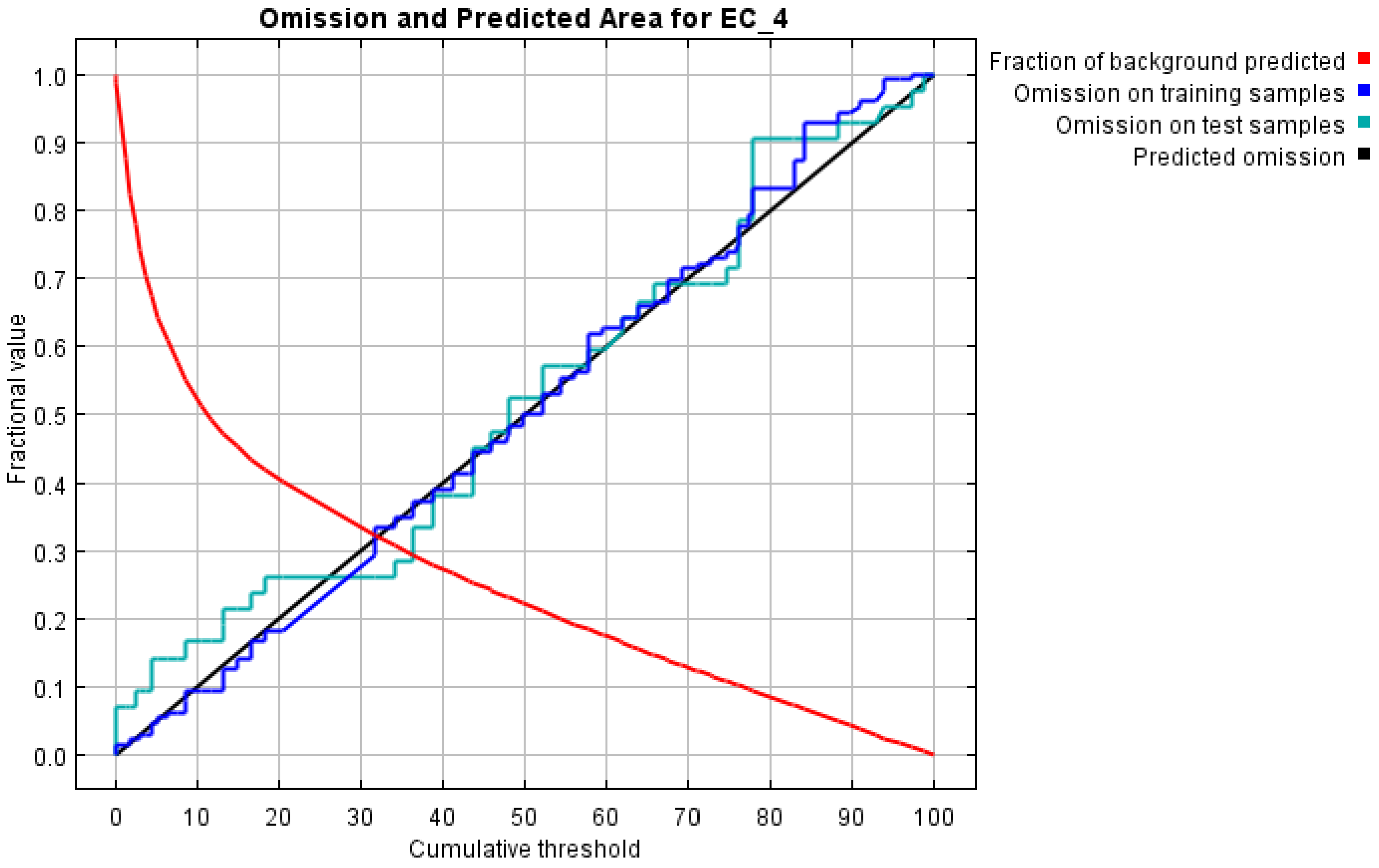

The next picture is the receiver operating characteristic (ROC) curve for the same data. Note that the specificity is defined using predicted area, rather than true commission (see the paper by Phillips, Anderson and Schapire cited on the help page for discussion of what this means). This implies that the maximum achievable AUC is less than 1. If test data is drawn from the Maxent distribution itself, then the maximum possible test AUC would be 0.740 rather than 1; in practice the test AUC may exceed this bound.

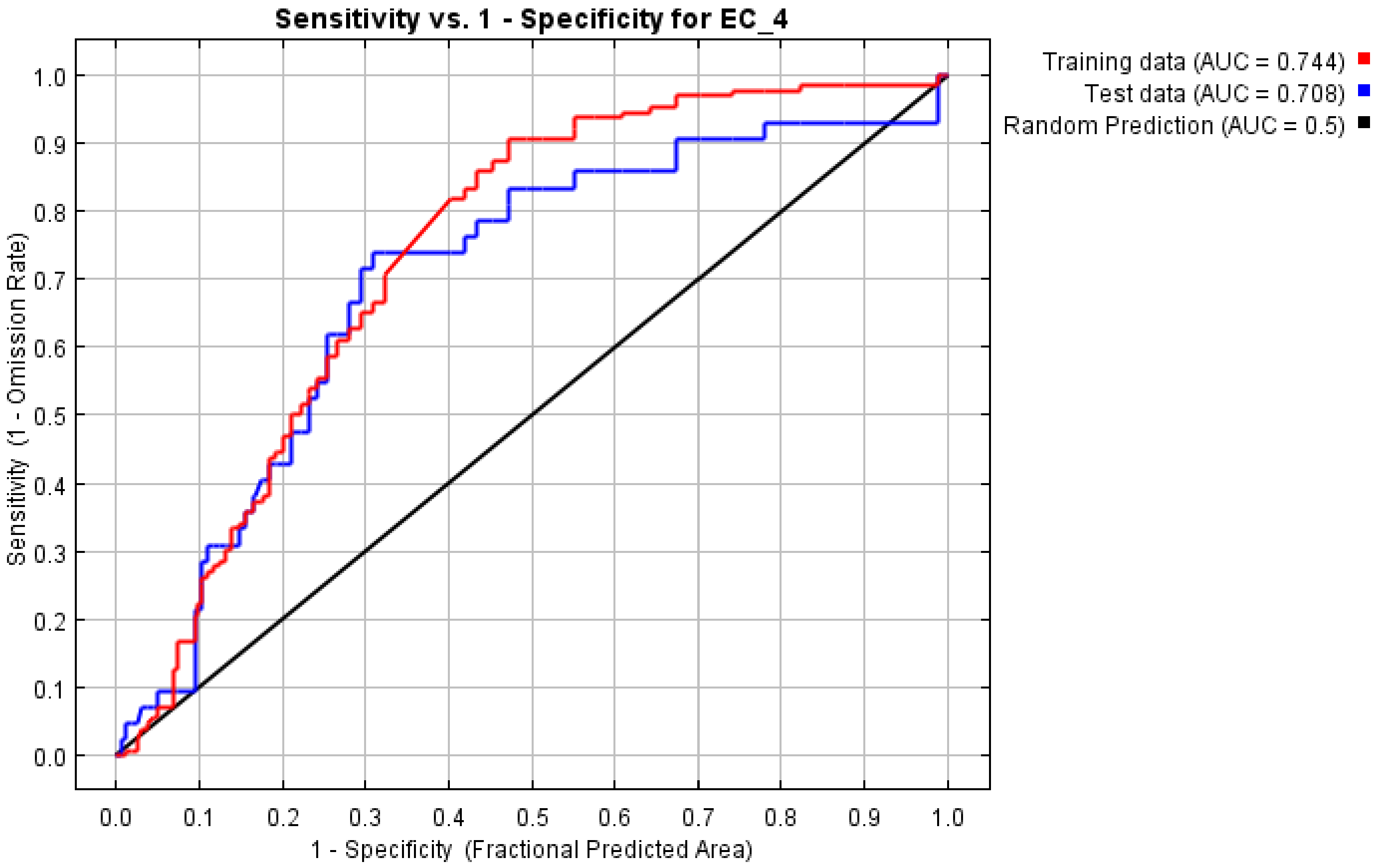

Some common thresholds and corresponding omission rates are as follows. If test data are available, binomial probabilities are calculated exactly if the number of test samples is at most 25, otherwise using a normal approximation to the binomial. These are 1-sided p-values for the null hypothesis that test points are predicted no better than by a random prediction with the same fractional predicted area. The "Balance" threshold minimizes 6 * training omission rate + .04 * cumulative threshold + 1.6 * fractional predicted area.

Cumulative threshold	Logistic threshold	Description	Fractional predicted area	Training omission rate	Test omission rate	P-value
1.000	0.072	Fixed cumulative value 1	0.877	0.016	0.071	1.529E-1
5.000	0.173	Fixed cumulative value 5	0.642	0.048	0.143	1.844E-3
10.000	0.286	Fixed cumulative value 10	0.513	0.095	0.167	1.638E-5
0.083	0.061	Minimum training presence	0.988	0.000	0.000	2.385E-1
13.186	0.320	10 percentile training presence	0.472	0.095	0.167	1.397E-6
31.829	0.503	Equal training sensitivity and specificity	0.323	0.333	0.262	4.39E-9
13.186	0.320	Maximum training sensitivity plus	0.472	0.095	0.167	1.397E-

		specificity				6
36.398	0.535	Equal test sensitivity and specificity	0.294	0.349	0.286	1.126E-9
34.089	0.522	Maximum test sensitivity plus specificity	0.308	0.333	0.262	7.918E-10
4.412	0.131	Balance training omission, predicted area and threshold value	0.673	0.032	0.095	6.938E-4
5.238	0.173	Equate entropy of thresholded and original distributions	0.642	0.048	0.143	1.844E-3

Click here to interactively explore this prediction using the Explain tool. If clicking from your browser does not succeed in starting the tool, try running the script in G:\Users\BB\Desktop\Maxent Results EC samples entire area\EC_4_explain.bat directly. This tool requires the environmental grids to be small enough that they all fit in memory.

Response curves

These curves show how each environmental variable affects the Maxent prediction. The curves show how the logistic prediction changes as each environmental variable is varied, keeping all other environmental variables at their average sample value. Click on a response curve to see a larger version. Note that the curves can be hard to interpret if you have strongly correlated variables, as the model may depend on the correlations in ways that are not evident in the curves. In other words, the curves show the marginal effect of changing exactly one variable, whereas the model may take advantage of sets of variables changing together.

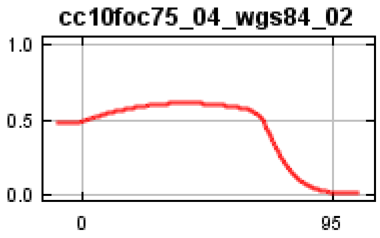

In contrast to the above marginal response curves, each of the following curves represents a different model, namely, a Maxent model created using only the corresponding variable. These plots reflect the dependence of predicted suitability both on the selected variable and on dependencies induced by correlations between the selected variable and other variables. They may be easier to interpret if there are strong correlations between variables.

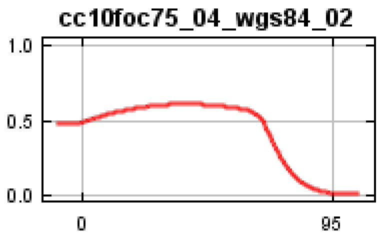

Analysis of variable contributions

The following table gives estimates of relative contributions of the environmental variables to the Maxent model. To determine the first estimate, in each iteration of the training algorithm, the increase in regularized gain is added to the contribution of the corresponding variable, or subtracted from it if the change to the absolute value of lambda is negative. For the second estimate, for each environmental variable in turn, the values of that variable on training presence and background data are randomly permuted. The model is reevaluated on the permuted data, and the resulting drop in training AUC is shown in the table, normalized to percentages. As with the variable jackknife, variable contributions should be interpreted with caution when the predictor variables are correlated.

Variable	Percent contribution	Permutation importance
cc10foc75_04_wgs84_02	100	100

Raw data outputs and control parameters

The data used in the above analysis is contained in the next links. Please see the Help button for more information on these.
The model applied to the training environmental layers
The coefficients of the model
The omission and predicted area for varying cumulative and raw thresholds
The prediction strength at the training and (optionally) test presence sites
Results for all species modeled in the same Maxent run, with summary statistics and (optionally) jackknife results

Regularized training gain is 0.404, training AUC is 0.744, unregularized training gain is 0.433.
Unregularized test gain is 0.254.
Test AUC is 0.708, standard deviation is 0.039 (calculated as in DeLong, DeLong & Clarke-Pearson 1988, equation 2).
Algorithm converged after 240 iterations (0 seconds).

The follow settings were used during the run:
126 presence records used for training, 42 for testing.
10000 points used to determine the Maxent distribution (background points and presence points).
Environmental layers used (all continuous): cc10foc75_04_wgs84_02
Regularization values: linear/quadratic/product: 0.050, categorical: 0.250, threshold: 1.000, hinge: 0.500
Feature types used: hinge linear threshold quadratic
responsecurves: true
pictures: false
outputdirectory: G:\Users\BB\Desktop\Maxent Results EC samples entire area
samplesfile: C:\Users\Bill\Desktop\Maxent_samples\Compare_NEC_EC_150m_311_WGS_84_m.csv
environmentallayers: C:\Users\Bill\Desktop\Maxent Layers\maxent.cache
randomseed: true
randomtestpoints: 25
replicates: 10
replicatetype: subsample

Maxent model for EC_5

This page contains some analysis of the Maxent model for EC_5, created Thu Jul 09 21:28:18 EDT 2015 using Maxent version 3.3.3k. If you would like to do further analyses, the raw data used here is linked to at the end of this page.

Analysis of omission/commission

The following picture shows the omission rate and predicted area as a function of the cumulative threshold. The omission rate is is calculated both on the training presence records, and (if test data are used) on the test records. The omission rate should be close to the predicted omission, because of the definition of the cumulative threshold.

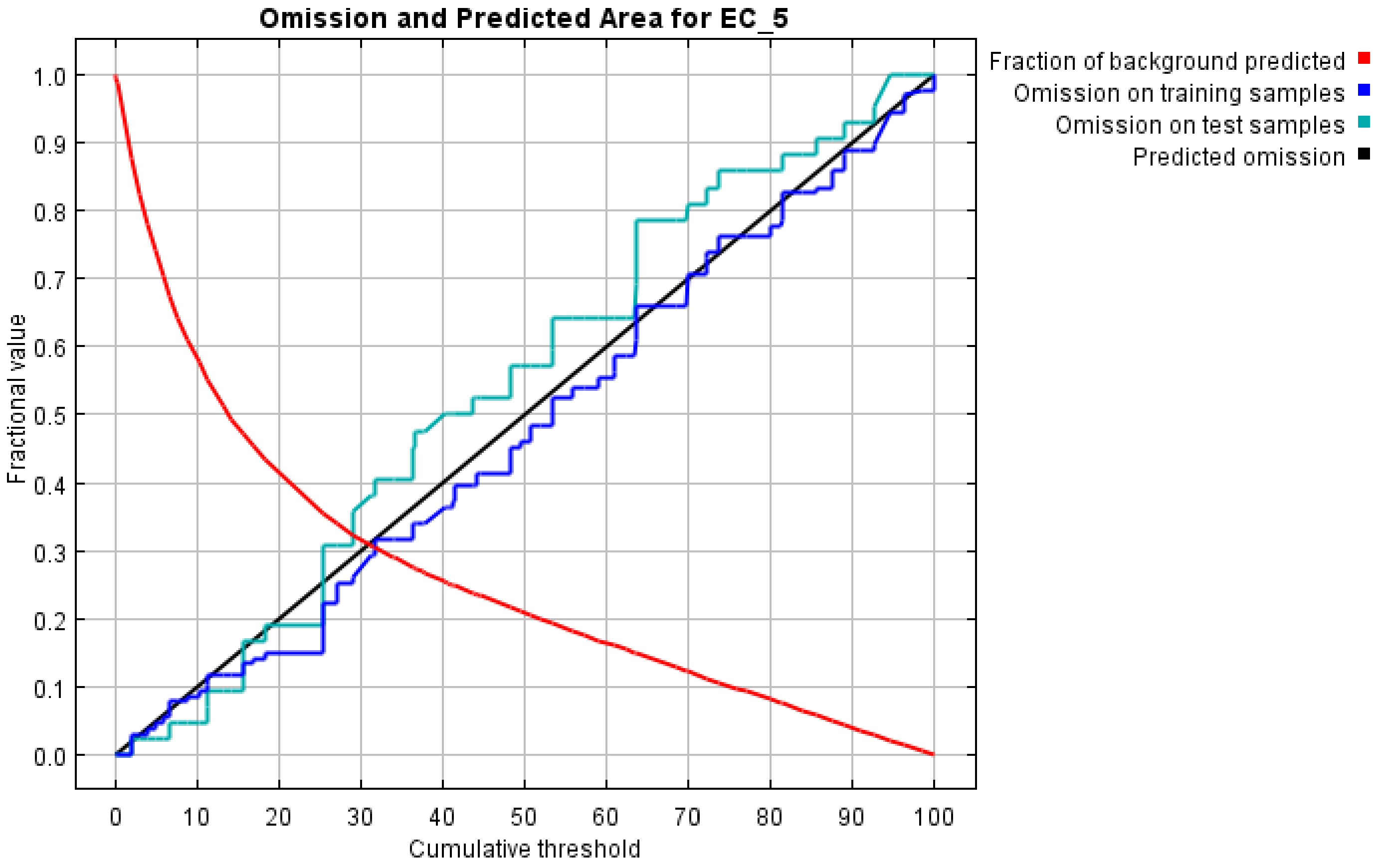

The next picture is the receiver operating characteristic (ROC) curve for the same data. Note that the specificity is defined using predicted area, rather than true commission (see the paper by Phillips, Anderson and Schapire cited on the help page for discussion of what this means). This implies that the maximum achievable AUC is less than 1. If test data is drawn from the Maxent distribution itself, then the maximum possible test AUC would be 0.737 rather than 1; in practice the test AUC may exceed this bound.

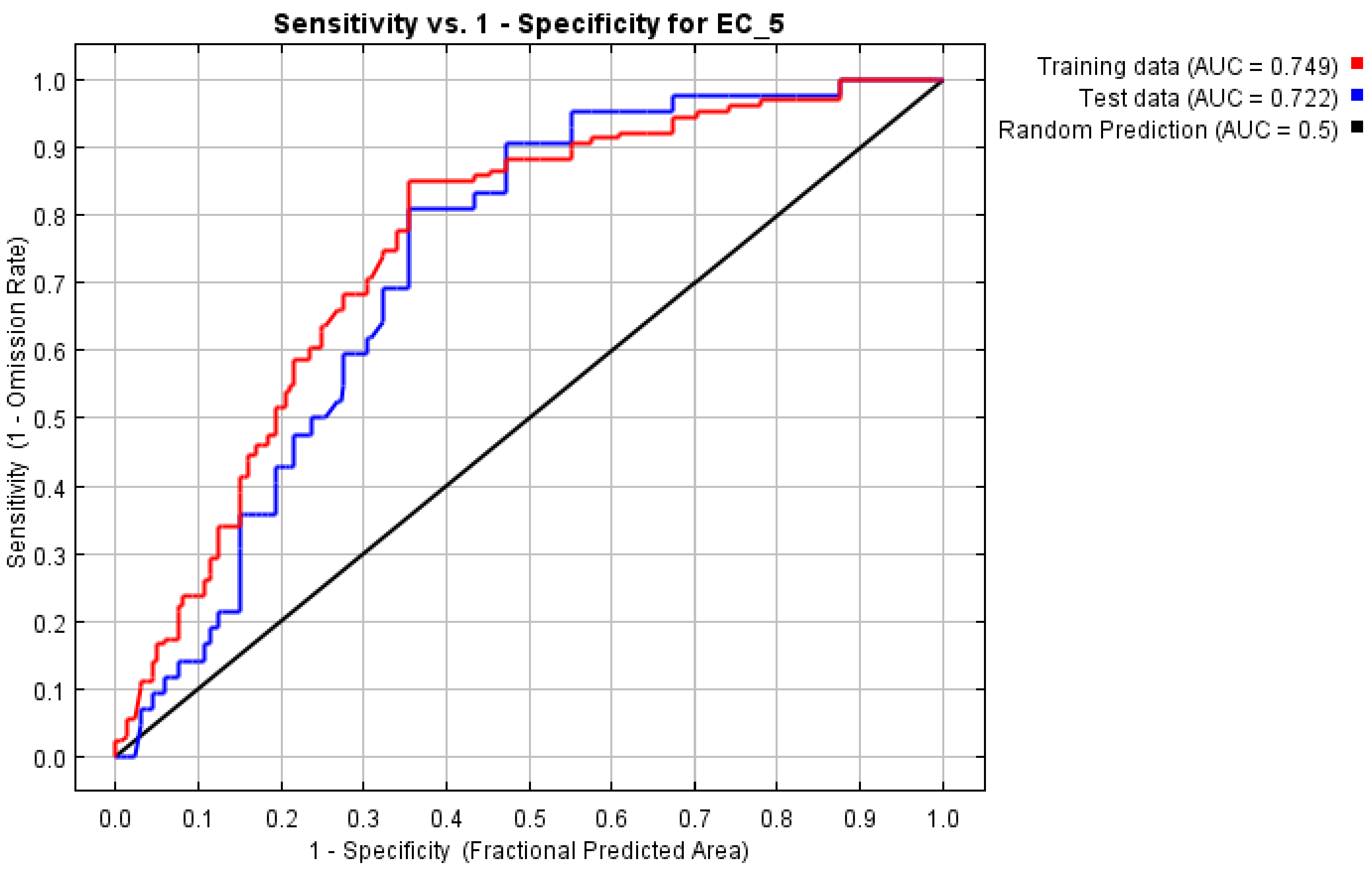

Some common thresholds and corresponding omission rates are as follows. If test data are available, binomial probabilities are calculated exactly if the number of test samples is at most 25, otherwise using a normal approximation to the binomial. These are 1-sided p-values for the null hypothesis that test points are predicted no better than by a random prediction with the same fractional predicted area. The "Balance" threshold minimizes 6 * training omission rate + .04 * cumulative threshold + 1.6 * fractional predicted area.

Cumulative threshold	Logistic threshold	Description	Fractional predicted area	Training omission rate	Test omission rate	P-value
1.000	0.102	Fixed cumulative value 1	0.877	0.000	0.000	7.517E-3
5.000	0.150	Fixed cumulative value 5	0.704	0.048	0.024	5.572E-5
10.000	0.215	Fixed cumulative value 10	0.577	0.087	0.048	4.16E-7
2.005	0.102	Minimum training presence	0.877	0.000	0.000	7.517E-3
11.278	0.233	10 percentile training presence	0.551	0.095	0.048	8.377E-8
31.663	0.513	Equal training sensitivity and specificity	0.305	0.302	0.381	4.881E-6
25.395	0.379	Maximum training sensitivity plus specificity	0.354	0.151	0.190	3.307E-10

29.105	0.463	Equal test sensitivity and specificity	0.323	0.254	0.310	1.764E-7
25.395	0.379	Maximum test sensitivity plus specificity	0.354	0.151	0.190	3.307E-10
2.005	0.102	Balance training omission, predicted area and threshold value	0.877	0.000	0.000	7.517E-3
6.638	0.165	Equate entropy of thresholded and original distributions	0.673	0.056	0.024	1.43E-5

Click here to interactively explore this prediction using the Explain tool. If clicking from your browser does not succeed in starting the tool, try running the script in G:\Users\BB\Desktop\Maxent Results EC samples entire area\EC_5_explain.bat directly. This tool requires the environmental grids to be small enough that they all fit in memory.

Response curves

These curves show how each environmental variable affects the Maxent prediction. The curves show how the logistic prediction changes as each environmental variable is varied, keeping all other environmental variables at their average sample value. Click on a response curve to see a larger version. Note that the curves can be hard to interpret if you have strongly correlated variables, as the model may depend on the correlations in ways that are not evident in the curves. In other words, the curves show the marginal effect of changing exactly one variable, whereas the model may take advantage of sets of variables changing together.

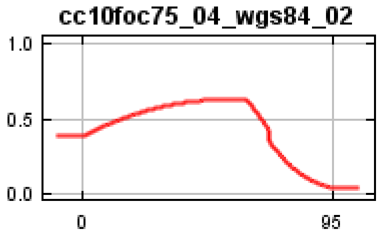

In contrast to the above marginal response curves, each of the following curves represents a different model, namely, a Maxent model created using only the corresponding variable. These plots reflect the dependence of predicted suitability both on the selected variable and on dependencies induced by correlations between the selected variable and other variables. They may be easier to interpret if there are strong correlations between variables.

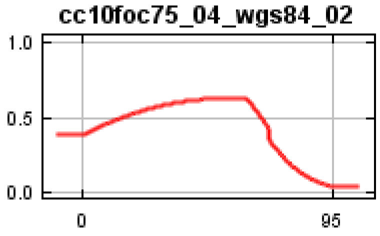

Analysis of variable contributions

The following table gives estimates of relative contributions of the environmental variables to the Maxent model. To determine the first estimate, in each iteration of the training algorithm, the increase in regularized gain is added to the contribution of the corresponding variable, or subtracted from it if the change to the absolute value of lambda is negative. For the second estimate, for each environmental variable in turn, the values of that variable on training presence and background data are randomly permuted. The model is reevaluated on the permuted data, and the resulting drop in training AUC is shown in the table, normalized to percentages. As with the variable jackknife, variable contributions should be interpreted with caution when the predictor variables are correlated.

Variable	Percent contribution	Permutation importance
cc10foc75_04_wgs84_02	100	100

Raw data outputs and control parameters

The data used in the above analysis is contained in the next links. Please see the Help button for more information on these.
The model applied to the training environmental layers
The coefficients of the model
The omission and predicted area for varying cumulative and raw thresholds
The prediction strength at the training and (optionally) test presence sites
Results for all species modeled in the same Maxent run, with summary statistics and (optionally) jackknife results

Regularized training gain is 0.380, training AUC is 0.749, unregularized training gain is 0.421.
Unregularized test gain is 0.347.
Test AUC is 0.722, standard deviation is 0.030 (calculated as in DeLong, DeLong & Clarke-Pearson 1988, equation 2).
Algorithm converged after 360 iterations (1 seconds).

The follow settings were used during the run:
126 presence records used for training, 42 for testing.
10000 points used to determine the Maxent distribution (background points and presence points).
Environmental layers used (all continuous): cc10foc75_04_wgs84_02
Regularization values: linear/quadratic/product: 0.050, categorical: 0.250, threshold: 1.000, hinge: 0.500
Feature types used: hinge linear threshold quadratic
responsecurves: true
pictures: false
outputdirectory: G:\Users\BB\Desktop\Maxent Results EC samples entire area
samplesfile: C:\Users\Bill\Desktop\Maxent_samples\Compare_NEC_EC_150m_311_WGS_84_m.csv
environmentallayers: C:\Users\Bill\Desktop\Maxent Layers\maxent.cache
randomseed: true
randomtestpoints: 25
replicates: 10
replicatetype: subsample
Command line used:

Maxent model for EC_6

This page contains some analysis of the Maxent model for EC_6, created Thu Jul 09 23:28:34 EDT 2015 using Maxent version 3.3.3k. If you would like to do further analyses, the raw data used here is linked to at the end of this page.

Analysis of omission/commission

The following picture shows the omission rate and predicted area as a function of the cumulative threshold. The omission rate is is calculated both on the training presence records, and (if test data are used) on the test records. The omission rate should be close to the predicted omission, because of the definition of the cumulative threshold.

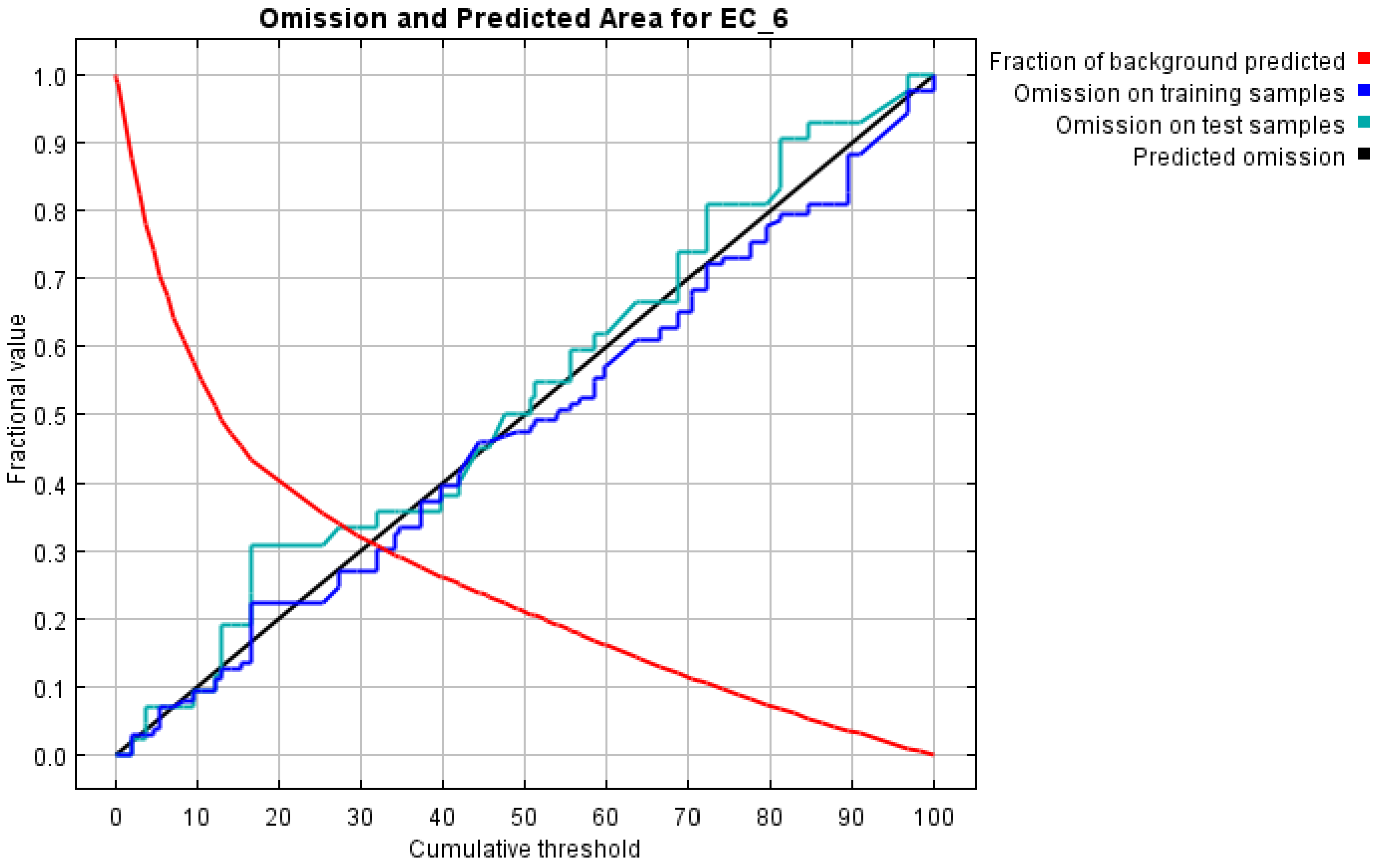

The next picture is the receiver operating characteristic (ROC) curve for the same data. Note that the specificity is defined using predicted area, rather than true commission (see the paper by Phillips, Anderson and Schapire cited on the help page for discussion of what this means). This implies that the maximum achievable AUC is less than 1. If test data is drawn from the Maxent distribution itself, then the maximum possible test AUC would be 0.742 rather than 1; in practice the test AUC may exceed this bound.

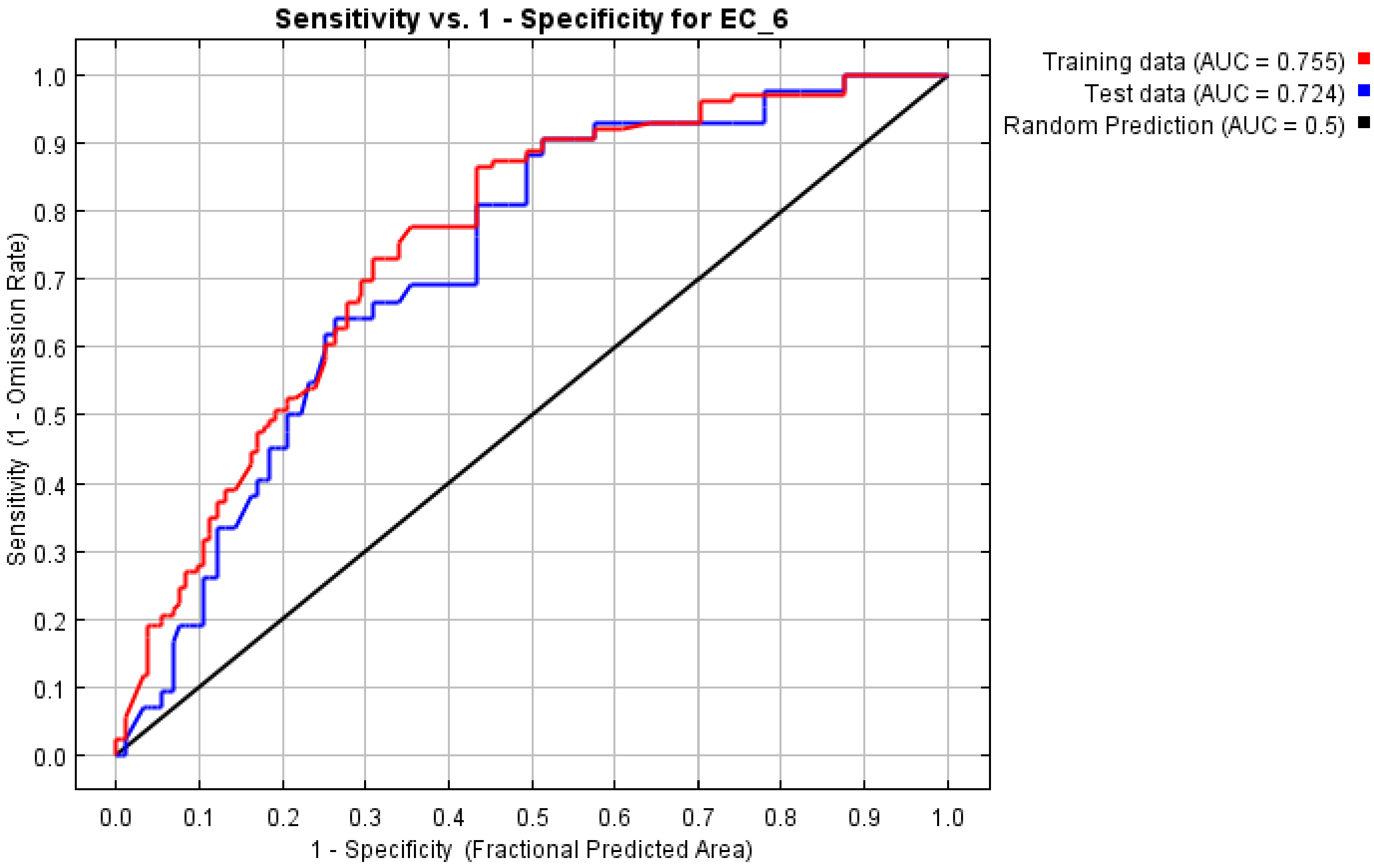

Some common thresholds and corresponding omission rates are as follows. If test data are available, binomial probabilities are calculated exactly if the number of test samples is at most 25, otherwise using a normal approximation to the binomial. These are 1-sided p-values for the null hypothesis that test points are predicted no better than by a random prediction with the same fractional predicted area. The "Balance" threshold minimizes 6 * training omission rate + .04 * cumulative threshold + 1.6 * fractional predicted area.

Cumulative threshold	Logistic threshold	Description	Fractional predicted area	Training omission rate	Test omission rate	P-value
1.000	0.096	Fixed cumulative value 1	0.877	0.000	0.000	7.517E-3
5.000	0.152	Fixed cumulative value 5	0.704	0.040	0.071	7.158E-4
10.000	0.228	Fixed cumulative value 10	0.551	0.095	0.095	1.997E-6
1.901	0.096	Minimum training presence	0.877	0.000	0.000	7.517E-3
12.088	0.228	10 percentile training presence	0.513	0.095	0.095	1.892E-7
31.872	0.530	Equal training sensitivity and specificity	0.308	0.302	0.357	1.308E-6
16.547	0.303	Maximum training sensitivity plus	0.434	0.135	0.190	4.415E-

		specificity				7
27.335	0.484	Equal test sensitivity and specificity	0.339	0.246	0.333	3.687E-6
12.088	0.228	Maximum test sensitivity plus specificity	0.513	0.095	0.095	1.892E-7
1.901	0.096	Balance training omission, predicted area and threshold value	0.877	0.000	0.000	7.517E-3
6.226	0.166	Equate entropy of thresholded and original distributions	0.673	0.071	0.071	2.108E-4

Click here to interactively explore this prediction using the Explain tool. If clicking from your browser does not succeed in starting the tool, try running the script in G:\Users\BB\Desktop\Maxent Results EC samples entire area\EC_6_explain.bat directly. This tool requires the environmental grids to be small enough that they all fit in memory.

Response curves

These curves show how each environmental variable affects the Maxent prediction. The curves show how the logistic prediction changes as each environmental variable is varied, keeping all other environmental variables at their average sample value. Click on a response curve to see a larger version. Note that the curves can be hard to interpret if you have strongly correlated variables, as the model may depend on the correlations in ways that are not evident in the curves. In other words, the curves show the marginal effect of changing exactly one variable, whereas the model may take advantage of sets of variables changing together.

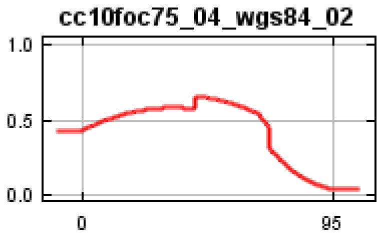

In contrast to the above marginal response curves, each of the following curves represents a different model, namely, a Maxent model created using only the corresponding variable. These plots reflect the dependence of predicted suitability both on the selected variable and on dependencies induced by correlations between the selected variable and other variables. They may be easier to interpret if there are strong correlations between variables.

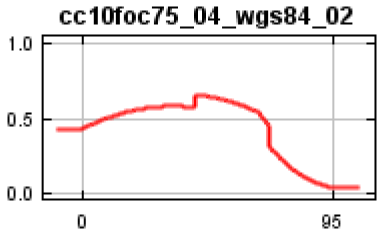

Analysis of variable contributions

The following table gives estimates of relative contributions of the environmental variables to the Maxent model. To determine the first estimate, in each iteration of the training algorithm, the increase in regularized gain is added to the contribution of the corresponding variable, or subtracted from it if the change to the absolute value of lambda is negative. For the second estimate, for each environmental variable in turn, the values of that variable on training presence and background data are randomly permuted. The model is reevaluated on the permuted data, and the resulting drop in training AUC is shown in the table, normalized to percentages. As with the variable jackknife, variable contributions should be interpreted with caution when the predictor variables are correlated.

Variable	Percent contribution	Permutation importance
cc10foc75_04_wgs84_02	100	100

Raw data outputs and control parameters

The data used in the above analysis is contained in the next links. Please see the Help button for more information on these.
The model applied to the training environmental layers
The coefficients of the model
The omission and predicted area for varying cumulative and raw thresholds
The prediction strength at the training and (optionally) test presence sites
Results for all species modeled in the same Maxent run, with summary statistics and (optionally) jackknife results

Regularized training gain is 0.394, training AUC is 0.755, unregularized training gain is 0.445.
Unregularized test gain is 0.312.
Test AUC is 0.724, standard deviation is 0.034 (calculated as in DeLong, DeLong & Clarke-Pearson 1988, equation 2).
Algorithm converged after 420 iterations (1 seconds).

The follow settings were used during the run:
126 presence records used for training, 42 for testing.
10000 points used to determine the Maxent distribution (background points and presence points).
Environmental layers used (all continuous): cc10foc75_04_wgs84_02
Regularization values: linear/quadratic/product: 0.050, categorical: 0.250, threshold: 1.000, hinge: 0.500
Feature types used: hinge linear threshold quadratic
responsecurves: true
pictures: false
outputdirectory: G:\Users\BB\Desktop\Maxent Results EC samples entire area
samplesfile: C:\Users\Bill\Desktop\Maxent_samples\Compare_NEC_EC_150m_311_WGS_84_m.csv
environmentallayers: C:\Users\Bill\Desktop\Maxent Layers\maxent.cache
randomseed: true
randomtestpoints: 25
replicates: 10
replicatetype: subsample

Maxent model for EC_7

This page contains some analysis of the Maxent model for EC_7, created Fri Jul 10 00:49:44 EDT 2015 using Maxent version 3.3.3k. If you would like to do further analyses, the raw data used here is linked to at the end of this page.

Analysis of omission/commission

The following picture shows the omission rate and predicted area as a function of the cumulative threshold. The omission rate is is calculated both on the training presence records, and (if test data are used) on the test records. The omission rate should be close to the predicted omission, because of the definition of the cumulative threshold.

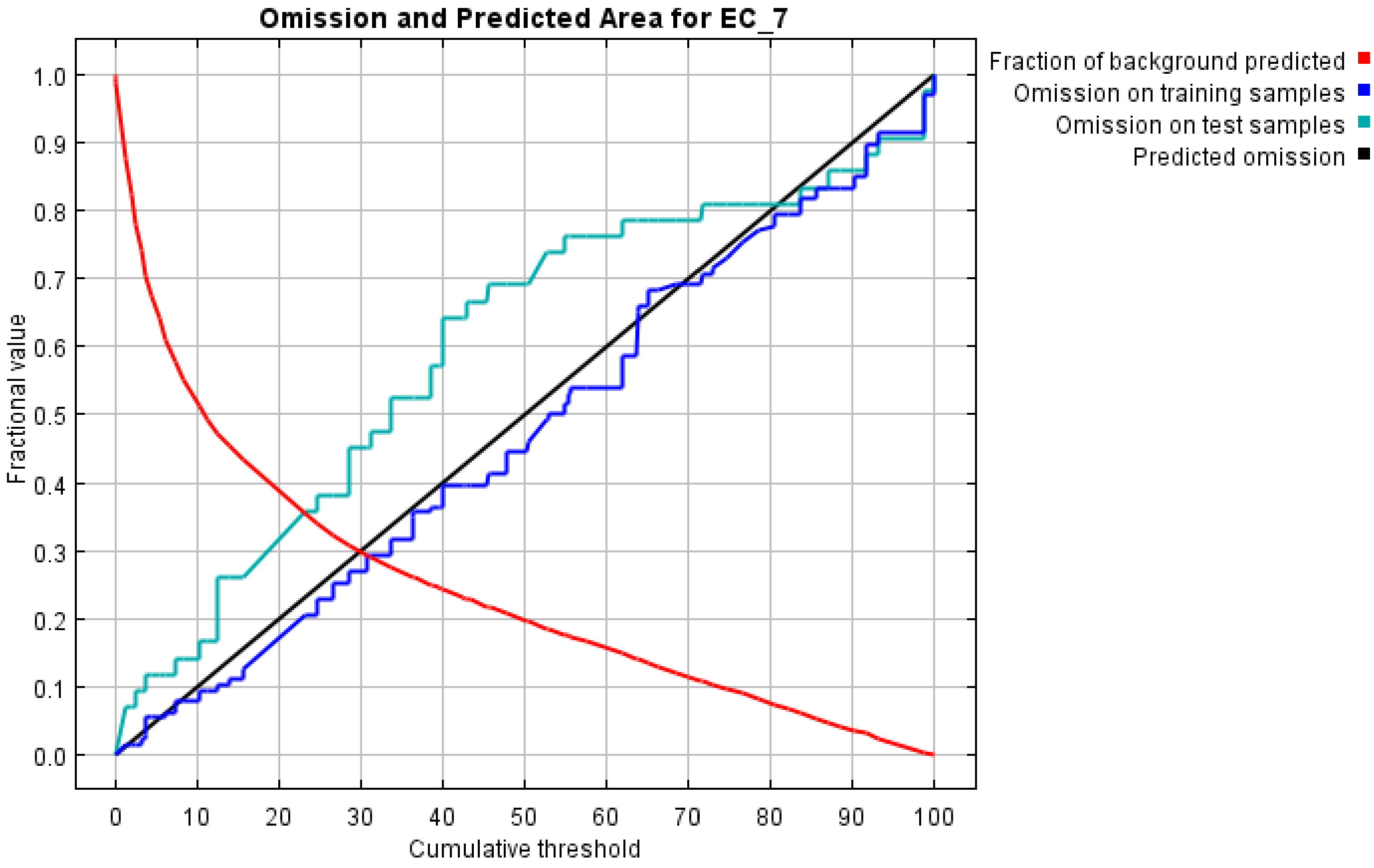

The next picture is the receiver operating characteristic (ROC) curve for the same data. Note that the specificity is defined using predicted area, rather than true commission (see the paper by Phillips, Anderson and Schapire cited on the help page for discussion of what this means). This implies that the maximum achievable AUC is less than 1. If test data is drawn from the Maxent distribution itself, then the maximum possible test AUC would be 0.755 rather than 1; in practice the test AUC may exceed this bound.

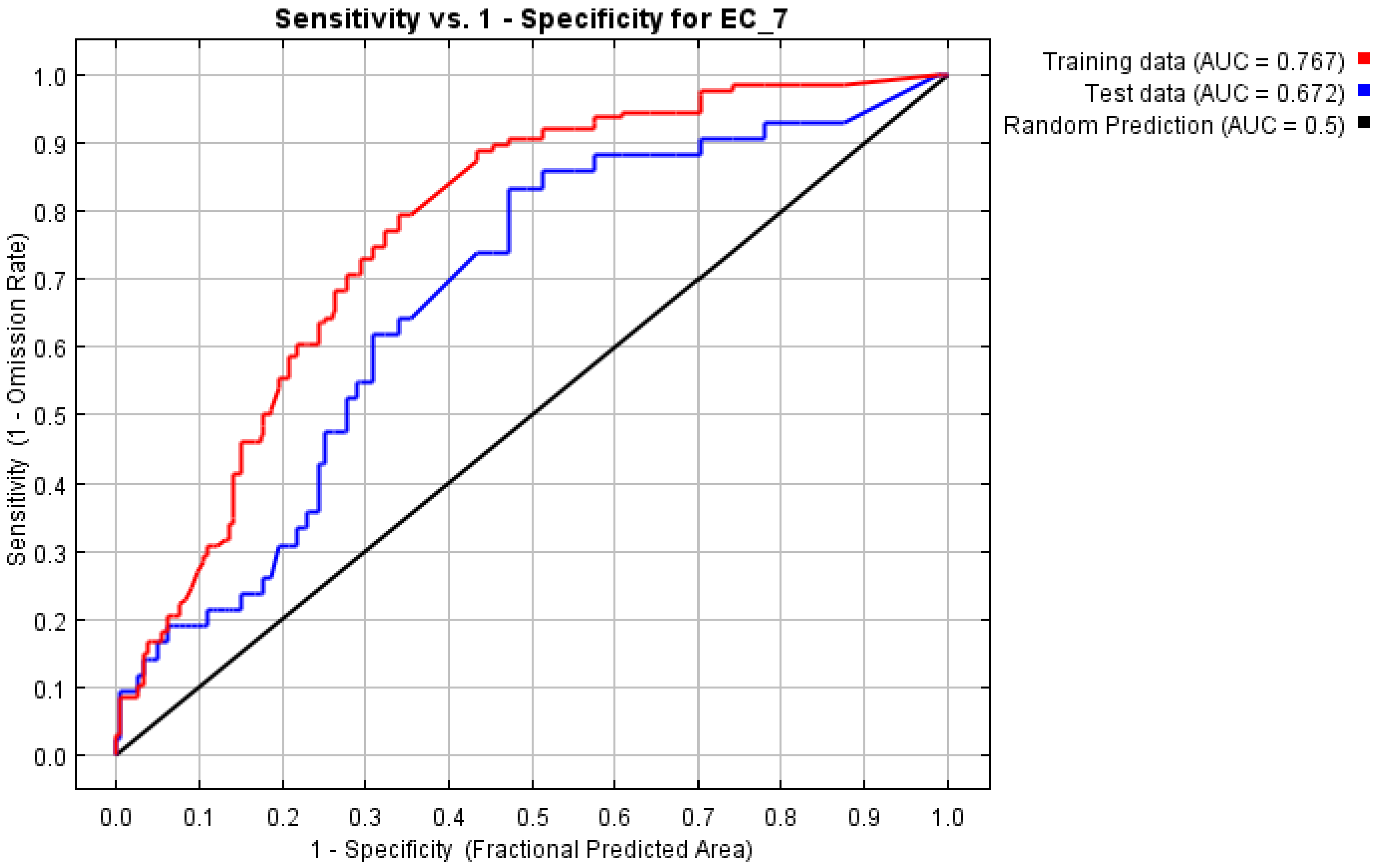

Some common thresholds and corresponding omission rates are as follows. If test data are available, binomial probabilities are calculated exactly if the number of test samples is at most 25, otherwise using a normal approximation to the binomial. These are 1-sided p-values for the null hypothesis that test points are predicted no better than by a random prediction with the same fractional predicted area. The "Balance" threshold minimizes 6 * training omission rate + .04 * cumulative threshold + 1.6 * fractional predicted area.

Cumulative threshold	Logistic threshold	Description	Fractional predicted area	Training omission rate	Test omission rate	P-value
1.000	0.070	Fixed cumulative value 1	0.877	0.016	0.071	1.529E-1
5.000	0.160	Fixed cumulative value 5	0.642	0.056	0.119	6.286E-4
10.000	0.229	Fixed cumulative value 10	0.513	0.079	0.143	4.058E-6
0.088	0.061	Minimum training presence	0.988	0.000	0.000	2.385E-1
12.547	0.285	10 percentile training presence	0.472	0.095	0.167	1.397E-6
30.636	0.508	Equal training sensitivity and specificity	0.294	0.294	0.452	1.545E-4
15.614	0.353	Maximum training sensitivity plus	0.434	0.111	0.262	3.417E-

		specificity				5
23.264	0.386	Equal test sensitivity and specificity	0.354	0.206	0.357	4.497E-5
12.547	0.285	Maximum test sensitivity plus specificity	0.472	0.095	0.167	1.397E-6
3.087	0.094	Balance training omission, predicted area and threshold value	0.741	0.016	0.095	7.606E-3
6.214	0.160	Equate entropy of thresholded and original distributions	0.610	0.056	0.119	1.583E-4

Click here to interactively explore this prediction using the Explain tool. If clicking from your browser does not succeed in starting the tool, try running the script in G:\Users\BB\Desktop\Maxent Results EC samples entire area\EC_7_explain.bat directly. This tool requires the environmental grids to be small enough that they all fit in memory.

Response curves

These curves show how each environmental variable affects the Maxent prediction. The curves show how the logistic prediction changes as each environmental variable is varied, keeping all other environmental variables at their average sample value. Click on a response curve to see a larger version. Note that the curves can be hard to interpret if you have strongly correlated variables, as the model may depend on the correlations in ways that are not evident in the curves. In other words, the curves show the marginal effect of changing exactly one variable, whereas the model may take advantage of sets of variables changing together.

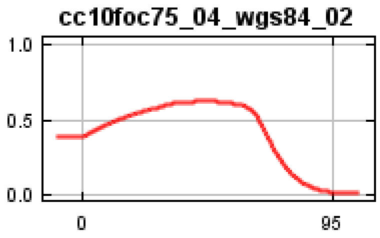

In contrast to the above marginal response curves, each of the following curves represents a different model, namely, a Maxent model created using only the corresponding variable. These plots reflect the dependence of predicted suitability both on the selected variable and on dependencies induced by correlations between the selected variable and other variables. They may be easier to interpret if there are strong correlations between variables.

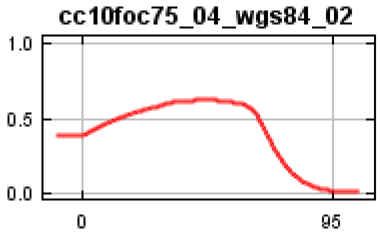

Analysis of variable contributions

The following table gives estimates of relative contributions of the environmental variables to the Maxent model. To determine the first estimate, in each iteration of the training algorithm, the increase in regularized gain is added to the contribution of the corresponding variable, or subtracted from it if the change to the absolute value of lambda is negative. For the second estimate, for each environmental variable in turn, the values of that variable on training presence and background data are randomly permuted. The model is reevaluated on the permuted data, and the resulting drop in training AUC is shown in the table, normalized to percentages. As with the variable jackknife, variable contributions should be interpreted with caution when the predictor variables are correlated.

Variable	Percent contribution	Permutation importance
cc10foc75_04_wgs84_02	100	100

Raw data outputs and control parameters

The data used in the above analysis is contained in the next links. Please see the Help button for more information on these.
The model applied to the training environmental layers
The coefficients of the model
The omission and predicted area for varying cumulative and raw thresholds
The prediction strength at the training and (optionally) test presence sites
Results for all species modeled in the same Maxent run, with summary statistics and (optionally) jackknife results

Regularized training gain is 0.449, training AUC is 0.767, unregularized training gain is 0.485.
Unregularized test gain is 0.134.
Test AUC is 0.672, standard deviation is 0.038 (calculated as in DeLong, DeLong & Clarke-Pearson 1988, equation 2).
Algorithm converged after 440 iterations (1 seconds).

The follow settings were used during the run:
126 presence records used for training, 42 for testing.
10000 points used to determine the Maxent distribution (background points and presence points).
Environmental layers used (all continuous): cc10foc75_04_wgs84_02
Regularization values: linear/quadratic/product: 0.050, categorical: 0.250, threshold: 1.000, hinge: 0.500
Feature types used: hinge linear threshold quadratic
responsecurves: true
pictures: false
outputdirectory: G:\Users\BB\Desktop\Maxent Results EC samples entire area
samplesfile: C:\Users\Bill\Desktop\Maxent_samples\Compare_NEC_EC_150m_311_WGS_84_m.csv
environmentallayers: C:\Users\Bill\Desktop\Maxent Layers\maxent.cache
randomseed: true
randomtestpoints: 25
replicates: 10
replicatetype: subsample

Maxent model for EC_8

This page contains some analysis of the Maxent model for EC_8, created Fri Jul 10 03:09:57 EDT 2015 using Maxent version 3.3.3k. If you would like to do further analyses, the raw data used here is linked to at the end of this page.

Analysis of omission/commission

The following picture shows the omission rate and predicted area as a function of the cumulative threshold. The omission rate is is calculated both on the training presence records, and (if test data are used) on the test records. The omission rate should be close to the predicted omission, because of the definition of the cumulative threshold.

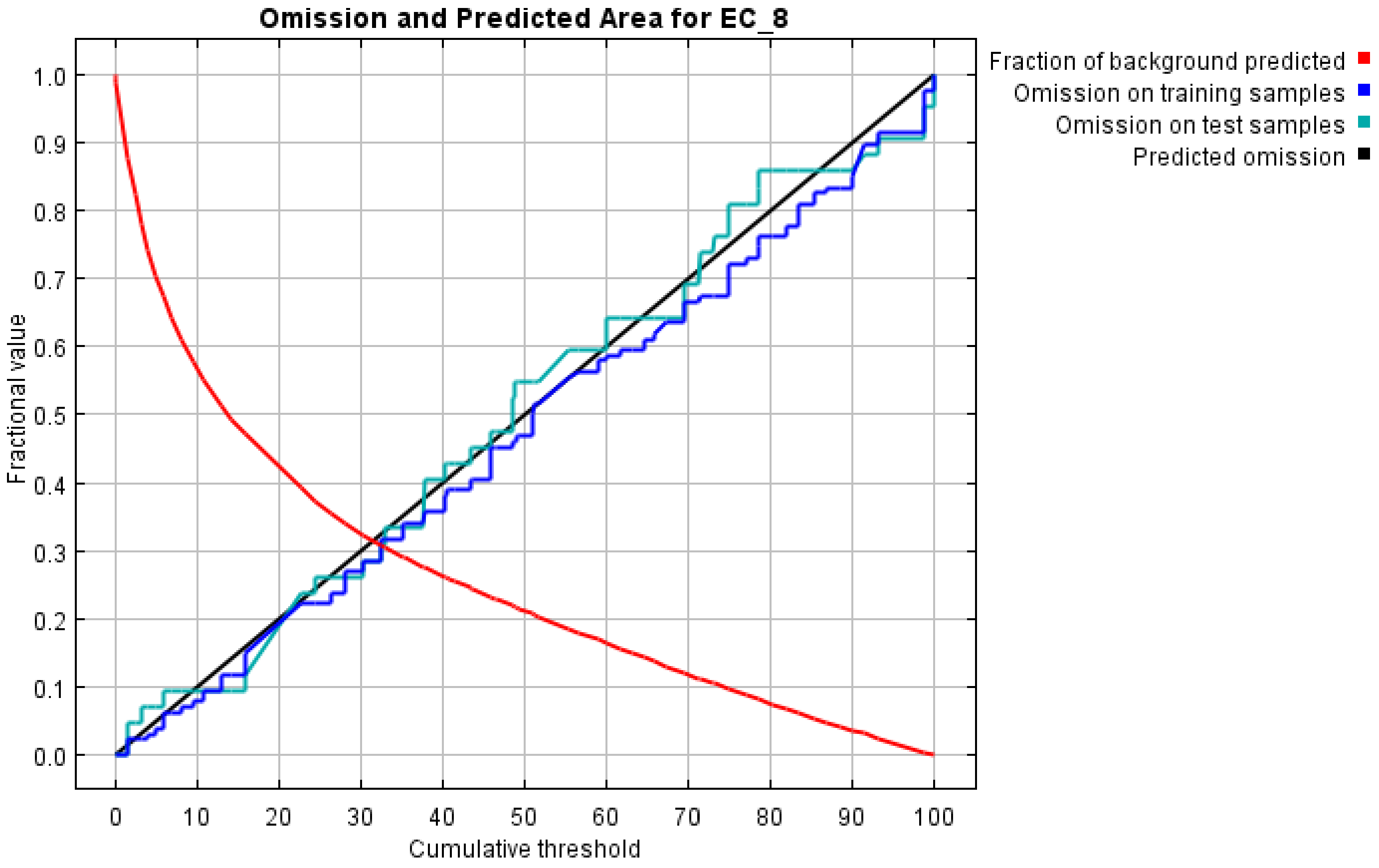

The next picture is the receiver operating characteristic (ROC) curve for the same data. Note that the specificity is defined using predicted area, rather than true commission (see the paper by Phillips, Anderson and Schapire cited on the help page for discussion of what this means). This implies that the maximum achievable AUC is less than 1. If test data is drawn from the Maxent distribution itself, then the maximum possible test AUC would be 0.738 rather than 1; in practice the test AUC may exceed this bound.

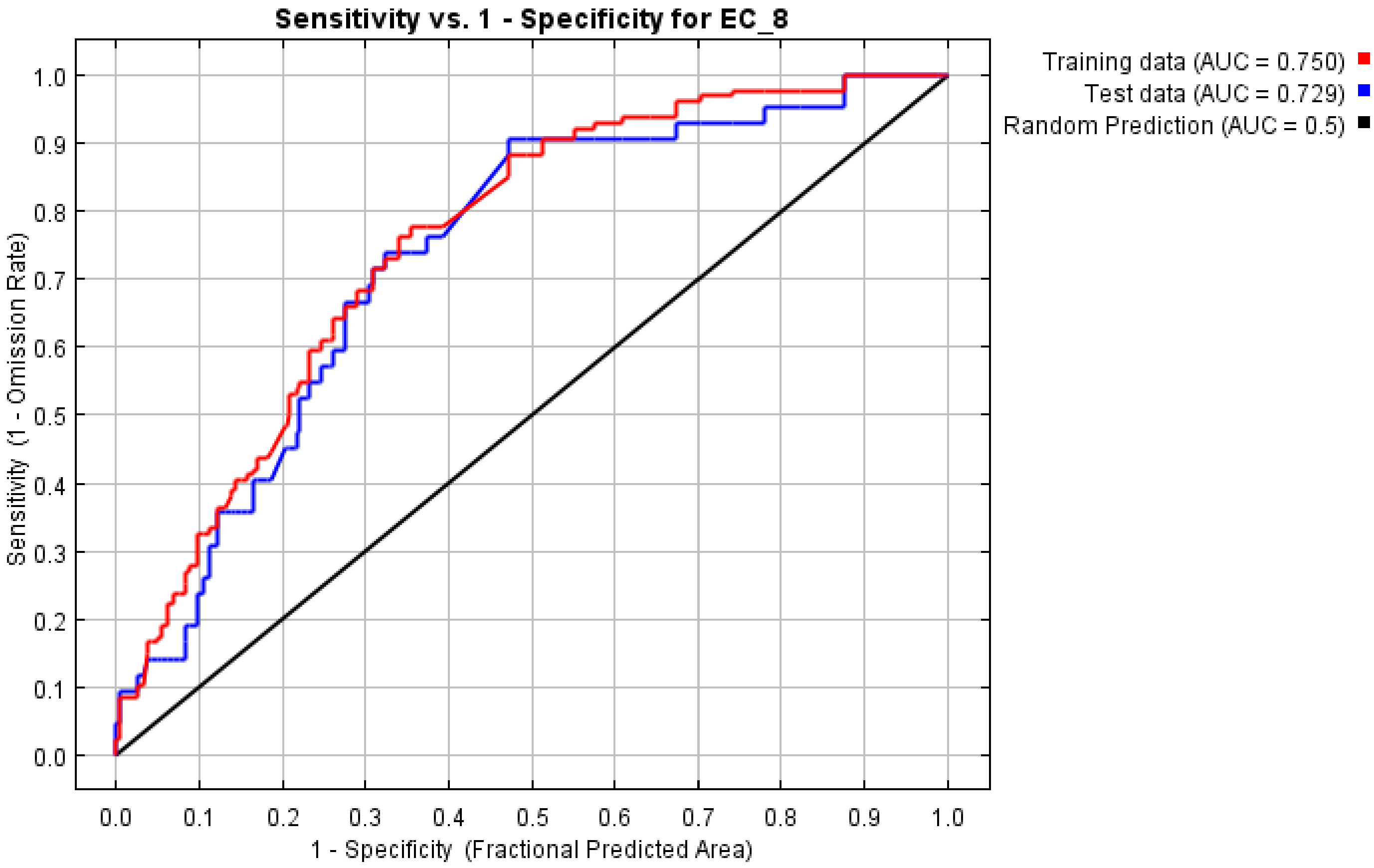

Some common thresholds and corresponding omission rates are as follows. If test data are available, binomial probabilities are calculated exactly if the number of test samples is at most 25, otherwise using a normal approximation to the binomial. These are 1-sided p-values for the null hypothesis that test points are predicted no better than by a random prediction with the same fractional predicted area. The "Balance" threshold minimizes 6 * training omission rate + .04 * cumulative threshold + 1.6 * fractional predicted area.

Cumulative threshold	Logistic threshold	Description	Fractional predicted area	Training omission rate	Test omission rate	P-value
1.000	0.083	Fixed cumulative value 1	0.877	0.000	0.000	7.517E-3
5.000	0.161	Fixed cumulative value 5	0.673	0.040	0.071	2.108E-4
10.000	0.256	Fixed cumulative value 10	0.551	0.079	0.095	1.997E-6
1.581	0.083	Minimum training presence	0.877	0.000	0.000	7.517E-3
12.911	0.284	10 percentile training presence	0.513	0.095	0.095	1.892E-7
32.480	0.507	Equal training sensitivity and specificity	0.308	0.317	0.310	3.998E-8
26.373	0.410	Maximum training sensitivity plus	0.354	0.222	0.262	9.597E-

		specificity				8
32.480	0.507	Equal test sensitivity and specificity	0.308	0.317	0.310	3.998E-8
15.846	0.345	Maximum test sensitivity plus specificity	0.472	0.119	0.095	9.965E-9
1.581	0.083	Balance training omission, predicted area and threshold value	0.877	0.000	0.000	7.517E-3
5.736	0.161	Equate entropy of thresholded and original distributions	0.673	0.040	0.071	2.108E-4

Click here to interactively explore this prediction using the Explain tool. If clicking from your browser does not succeed in starting the tool, try running the script in G:\Users\BB\Desktop\Maxent Results EC samples entire area\EC_8_explain.bat directly. This tool requires the environmental grids to be small enough that they all fit in memory.

Response curves

These curves show how each environmental variable affects the Maxent prediction. The curves show how the logistic prediction changes as each environmental variable is varied, keeping all other environmental variables at their average sample value. Click on a response curve to see a larger version. Note that the curves can be hard to interpret if you have strongly correlated variables, as the model may depend on the correlations in ways that are not evident in the curves. In other words, the curves show the marginal effect of changing exactly one variable, whereas the model may take advantage of sets of variables changing together.

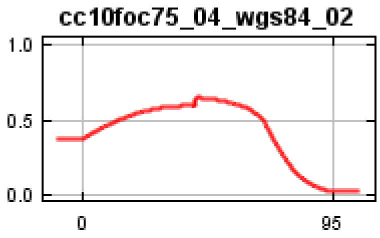

-

In contrast to the above marginal response curves, each of the following curves represents a different model, namely, a Maxent model created using only the corresponding variable. These plots reflect the dependence of predicted suitability both on the selected variable and on dependencies induced by correlations between the selected variable and other variables. They may be easier to interpret if there are strong correlations between variables.

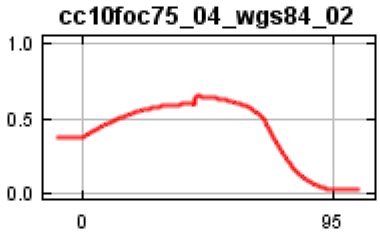

-

Analysis of variable contributions

The following table gives estimates of relative contributions of the environmental variables to the Maxent model. To determine the first estimate, in each iteration of the training algorithm, the increase in regularized gain is added to the contribution of the corresponding variable, or subtracted from it if the change to the absolute value of lambda is negative. For the second estimate, for each environmental variable in turn, the values of that variable on training presence and background data are randomly permuted. The model is reevaluated on the permuted data, and the resulting drop in training AUC is shown in the table, normalized to percentages. As with the variable jackknife, variable contributions should be interpreted with caution when the predictor variables are correlated.

Variable	Percent contribution	Permutation importance
cc10foc75_04_wgs84_02	100	100

Raw data outputs and control parameters

The data used in the above analysis is contained in the next links. Please see the Help button for more information on these.
The model applied to the training environmental layers
The coefficients of the model
The omission and predicted area for varying cumulative and raw thresholds
The prediction strength at the training and (optionally) test presence sites
Results for all species modeled in the same Maxent run, with summary statistics and (optionally) jackknife results

Regularized training gain is 0.379, training AUC is 0.750, unregularized training gain is 0.417.
Unregularized test gain is 0.356.
Test AUC is 0.729, standard deviation is 0.035 (calculated as in DeLong, DeLong & Clarke-Pearson 1988, equation 2).
Algorithm terminated after 500 iterations (1 seconds).

The follow settings were used during the run:
126 presence records used for training, 42 for testing.
10000 points used to determine the Maxent distribution (background points and presence points).
Environmental layers used (all continuous): cc10foc75_04_wgs84_02
Regularization values: linear/quadratic/product: 0.050, categorical: 0.250, threshold: 1.000, hinge: 0.500
Feature types used: hinge linear threshold quadratic
responsecurves: true
pictures: false
outputdirectory: G:\Users\BB\Desktop\Maxent Results EC samples entire area
samplesfile: C:\Users\Bill\Desktop\Maxent_samples\Compare_NEC_EC_150m_311_WGS_84_m.csv
environmentallayers: C:\Users\Bill\Desktop\Maxent Layers\maxent.cache
randomseed: true
randomtestpoints: 25
replicates: 10
replicatetype: subsample

Maxent model for EC_9

This page contains some analysis of the Maxent model for EC_9, created Fri Jul 10 04:47:34 EDT 2015 using Maxent version 3.3.3k. If you would like to do further analyses, the raw data used here is linked to at the end of this page.

Analysis of omission/commission

The following picture shows the omission rate and predicted area as a function of the cumulative threshold. The omission rate is is calculated both on the training presence records, and (if test data are used) on the test records. The omission rate should be close to the predicted omission, because of the definition of the cumulative threshold.

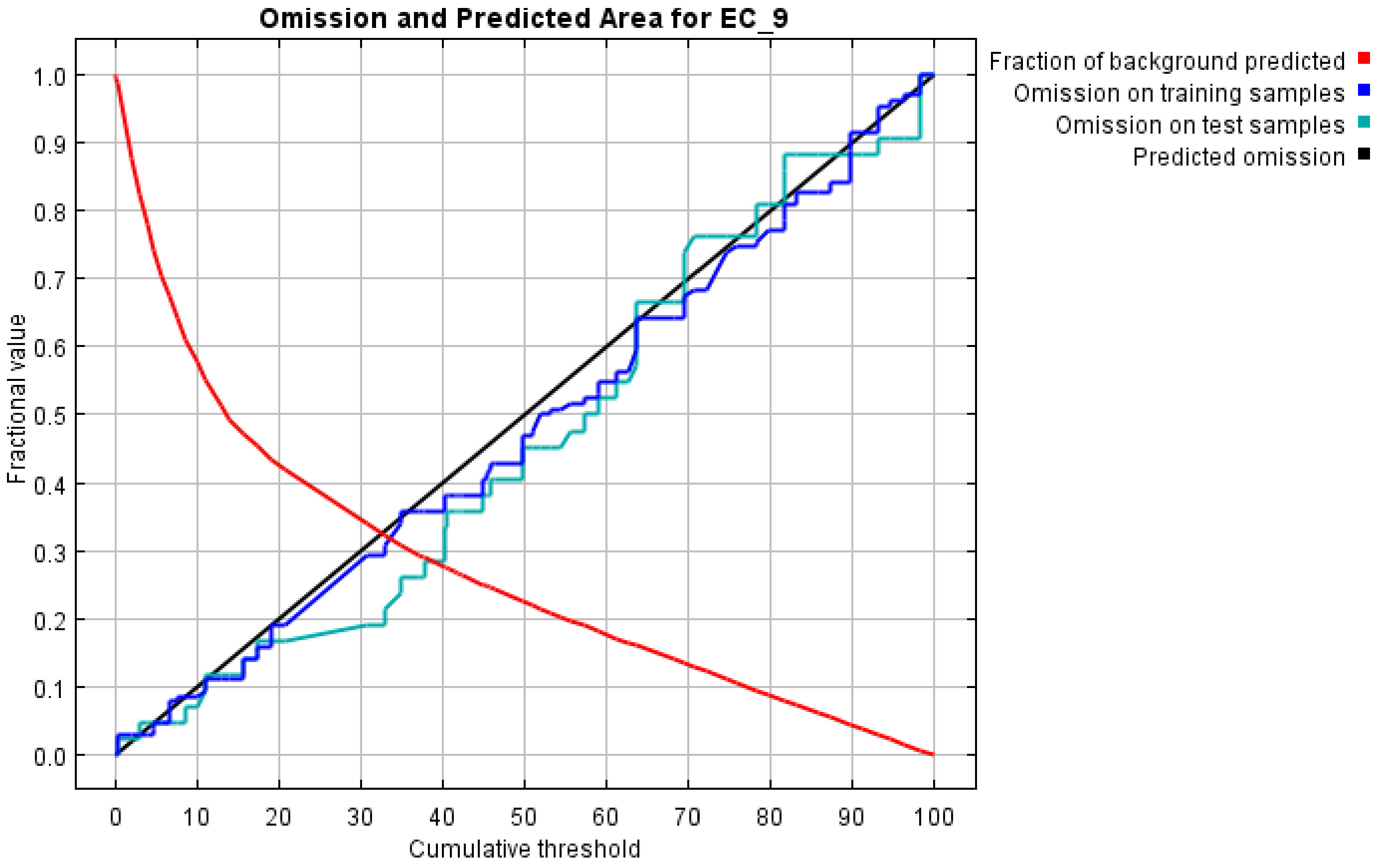

The next picture is the receiver operating characteristic (ROC) curve for the same data. Note that the specificity is defined using predicted area, rather than true commission (see the paper by Phillips, Anderson and Schapire cited on the help page for discussion of what this means). This implies that the maximum achievable AUC is less than 1. If test data is drawn from the Maxent distribution itself, then the maximum possible test AUC would be 0.726 rather than 1; in practice the test AUC may exceed this bound.

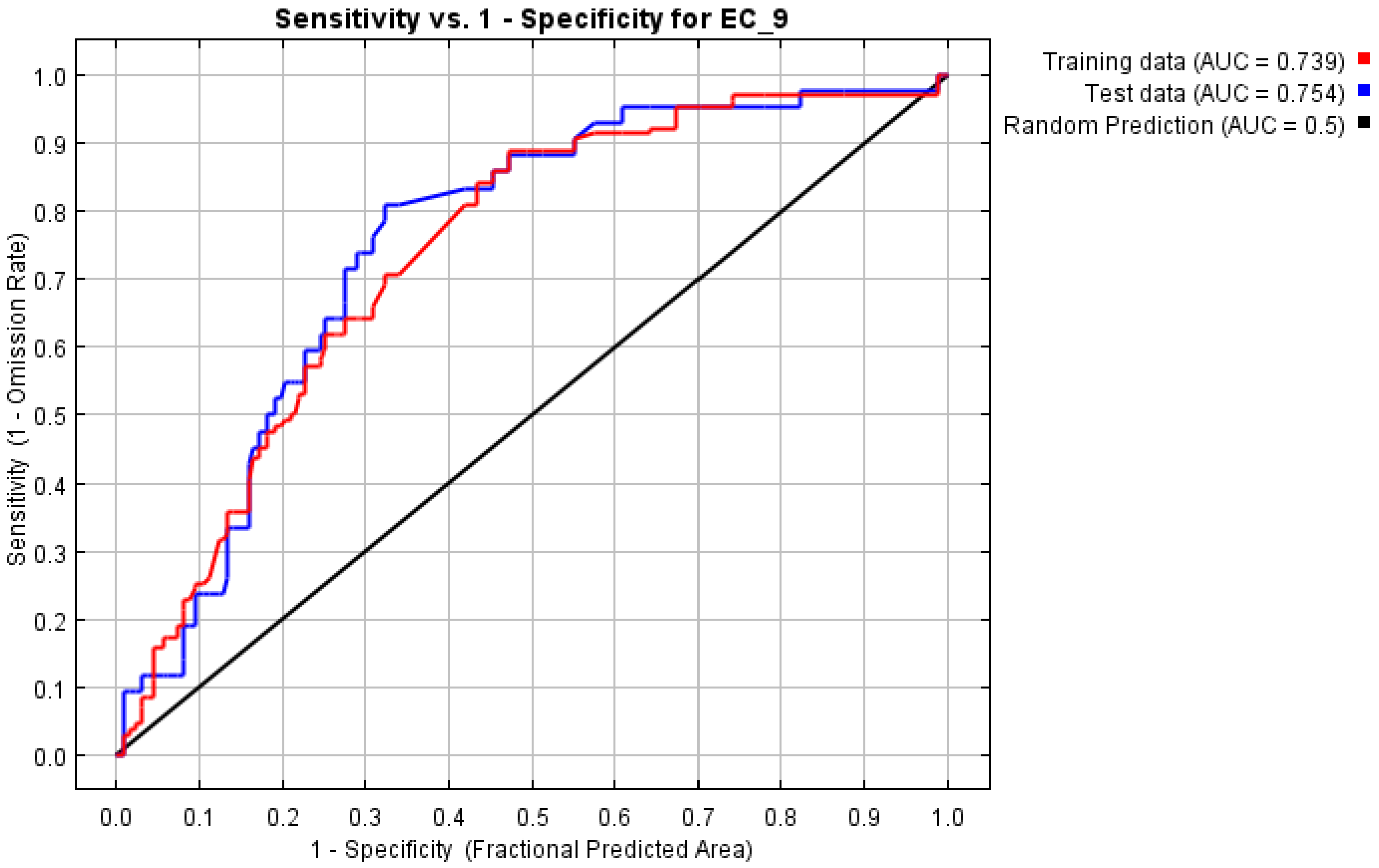

Some common thresholds and corresponding omission rates are as follows. If test data are available, binomial probabilities are calculated exactly if the number of test samples is at most 25, otherwise using a normal approximation to the binomial. These are 1-sided p-values for the null hypothesis that test points are predicted no better than by a random prediction with the same fractional predicted area. The "Balance" threshold minimizes 6 * training omission rate + .04 * cumulative threshold + 1.6 * fractional predicted area.

Cumulative threshold	Logistic threshold	Description	Fractional predicted area	Training omission rate	Test omission rate	P-value
1.000	0.114	Fixed cumulative value 1	0.877	0.032	0.024	2.486E-2
5.000	0.166	Fixed cumulative value 5	0.704	0.048	0.048	2.108E-4
10.000	0.254	Fixed cumulative value 10	0.551	0.095	0.095	1.997E-6
0.154	0.103	Minimum training presence	0.988	0.000	0.000	2.385E-1
11.044	0.254	10 percentile training presence	0.551	0.095	0.095	1.997E-6
32.873	0.498	Equal training sensitivity and specificity	0.323	0.310	0.214	7.148E-11
15.570	0.353	Maximum training sensitivity plus	0.472	0.111	0.119	5.679E-

		specificity				8
37.892	0.550	Equal test sensitivity and specificity	0.290	0.357	0.286	6.561E-10
32.859	0.472	Maximum test sensitivity plus specificity	0.323	0.294	0.190	7.774E-12
4.712	0.138	Balance training omission, predicted area and threshold value	0.741	0.032	0.048	8.7E-4
5.640	0.166	Equate entropy of thresholded and original distributions	0.704	0.048	0.048	2.108E-4

Click here to interactively explore this prediction using the Explain tool. If clicking from your browser does not succeed in starting the tool, try running the script in G:\Users\BB\Desktop\Maxent Results EC samples entire area\EC_9_explain.bat directly. This tool requires the environmental grids to be small enough that they all fit in memory.

Response curves

These curves show how each environmental variable affects the Maxent prediction. The curves show how the logistic prediction changes as each environmental variable is varied, keeping all other environmental variables at their average sample value. Click on a response curve to see a larger version. Note that the curves can be hard to interpret if you have strongly correlated variables, as the model may depend on the correlations in ways that are not evident in the curves. In other words, the curves show the marginal effect of changing exactly one variable, whereas the model may take advantage of sets of variables changing together.

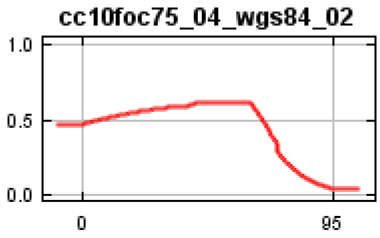

In contrast to the above marginal response curves, each of the following curves represents a different model, namely, a Maxent model created using only the corresponding variable. These plots reflect the dependence of predicted suitability both on the selected variable and on dependencies induced by correlations between the selected variable and other variables. They may be easier to interpret if there are strong correlations between variables.

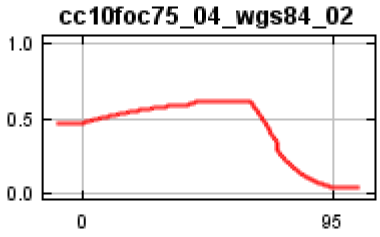

Analysis of variable contributions

The following table gives estimates of relative contributions of the environmental variables to the Maxent model. To determine the first estimate, in each iteration of the training algorithm, the increase in regularized gain is added to the contribution of the corresponding variable, or subtracted from it if the change to the absolute value of lambda is negative. For the second estimate, for each environmental variable in turn, the values of that variable on training presence and background data are randomly permuted. The model is reevaluated on the permuted data, and the resulting drop in training AUC is shown in the table, normalized to percentages. As with the variable jackknife, variable contributions should be interpreted with caution when the predictor variables are correlated.

Variable	Percent contribution	Permutation importance
cc10foc75_04_wgs84_02	100	100

Raw data outputs and control parameters

The data used in the above analysis is contained in the next links. Please see the Help button for more information on these.
The model applied to the training environmental layers
The coefficients of the model
The omission and predicted area for varying cumulative and raw thresholds
The prediction strength at the training and (optionally) test presence sites
Results for all species modeled in the same Maxent run, with summary statistics and (optionally) jackknife results

Regularized training gain is 0.351, training AUC is 0.739, unregularized training gain is 0.391.
Unregularized test gain is 0.438.
Test AUC is 0.754, standard deviation is 0.031 (calculated as in DeLong, DeLong & Clarke-Pearson 1988, equation 2).
Algorithm converged after 360 iterations (1 seconds).

The follow settings were used during the run:
126 presence records used for training, 42 for testing.
10000 points used to determine the Maxent distribution (background points and presence points).
Environmental layers used (all continuous): cc10foc75_04_wgs84_02
Regularization values: linear/quadratic/product: 0.050, categorical: 0.250, threshold: 1.000, hinge: 0.500
Feature types used: hinge linear threshold quadratic
responsecurves: true
pictures: false
outputdirectory: G:\Users\BB\Desktop\Maxent Results EC samples entire area
samplesfile: C:\Users\Bill\Desktop\Maxent_samples\Compare_NEC_EC_150m_311_WGS_84_m.csv
environmentallayers: C:\Users\Bill\Desktop\Maxent Layers\maxent.cache
randomseed: true
randomtestpoints: 25
replicates: 10
replicatetype: subsample
